# Does the Act of Copulation per se, without Considering Seminal Deposition, Change the Expression of Genes in the Porcine Female Genital Tract?

**DOI:** 10.3390/ijms21155477

**Published:** 2020-07-31

**Authors:** Manuel Alvarez-Rodriguez, Cristina A. Martinez, Dominic Wright, Heriberto Rodriguez-Martinez

**Affiliations:** 1 Department of Biomedical & Clinical Sciences (BKV), BKH/Obstetrics & Gynaecology, Faculty of Medicine and Health Sciences, Linköping University, SE-58185 Linköping, Sweden; manuel.alvarez-rodriguez@liu.se (M.A.-R.); cristina.martinez-serrano@liu.se (C.A.M.); 2 Department of Physics, Chemistry and Biology, Faculty of Science and Engineering, Linköping University, SE-58183 Linköping, Sweden; dominic.wright@liu.se

**Keywords:** natural mating, semen-free transcriptomics, semen-free gene expression, sow

## Abstract

Semen—through its specific sperm and seminal plasma (SP) constituents—induces changes of gene expression in the internal genital tract of pigs, particularly in the functional sperm reservoir at the utero-tubal junction (UTJ). Although seminal effects are similarly elicited by artificial insemination (AI), major changes in gene expression are registered after natural mating, a fact suggesting the act of copulation induces per se changes in genes that AI does not affect. The present study explored which pathways were solely influenced by copulation, affecting the differential expression of genes (DEGs) of the pre/peri-ovulatory genital tract (cervix, distal uterus, proximal uterus and UTJ) of estrus sows, 24 h after various procedures were performed to compare natural mating with AI of semen (control 1), sperm-free SP harvested from the sperm-peak fraction (control 2), sperm-free SP harvested from the whole ejaculate (control 3) or saline-extender BTS (control 4), using a microarray chip (GeneChip^®^ porcine gene 1.0 st array). Genes related to neuroendocrine responses (*ADRA1*, *ADRA2*, *GABRB2*, *CACNB2*), smooth muscle contractility (*WNT7A*), angiogenesis and vascular remodeling (*poFUT1*, *NTN4*) were, among others, overrepresented with distal and proximal uterine segments exhibiting the highest number of DEGs. The findings provide novel evidence that relevant transcriptomic changes in the porcine female reproductive tract occur in direct response to the specific act of copulation, being semen-independent.

## 1. Introduction

Copulation between a male and a female is definitory for sexual reproduction among species with internal fertilization [1]. Copulation implies a species-specific deposition of semen, from the placement of a spermatotheca in insects [2,3] to the ejaculation of a fluid built by the concerted secretion of different accessory sexual glands, the seminal plasma (SP) (in which epidydimal spermatozoa are suspended [4,5,6,7,8]) into different compartments of the internal genital tract (vagina to uterus). Decades of using in vitro fertilization demonstrated that the fertilization event is a matter for two gametes to interact at cell level [8]. However, semen has, by its specific sperm- and SP constituents proven able to induce major changes in the expression of genes in tissues of the internal genital tract alongside fertilization, in animal classes as disparate as insects or pigs [9]. Such changes clearly indicate pathways governing sperm transport [10], ovulation [11], sperm storage [4,6,12], sperm capacitation [12,13] and gamete encounter [14], processes that apparently continue during early embryo development and—for Eutheria—with placental development and pregnancy [11,15]. To complicate matters, while fertilization can be achieved without the intervention of immune responses, immunity modulation is apparently crucial to tolerate the presence of a foreign cell (the spermatozoon) and the proteins it and the accompanying SP carries in a female whose innate and adaptative immune system is prompt in eliminating both pathogens and foreign antigens. Such a conserved mechanism is overruled in internal fertilization by the attainment of a mechanism of maternal tolerance towards spermatozoa/semen [4,5,16,17] which is prolonged in Eutheria for the entire pregnancy, with embryos and placentae considered hemi-allogenic [15,18]. Semen elicit changes in gene expression changes in the female both after natural mating and artificial insemination (AI). However, the major changes in gene expression are elicited by natural mating [10]. This fact suggests that copulation acts per se differ from AI, no matter how similar it may seem to mating [19,20].

This study aimed to determine if the act of copulation in sows affected the differential expression of genes in the pre/peri-ovulatory genital tract (endocervix, the endometrium and the UTJ), without considering effects of semen or sperm-free SP. The study focused on DEGs not affected by AI with semen (control 1), sperm-free SP harvested from the sperm-peak fraction (control 2), sperm-free SP harvested from the whole ejaculate (control 3) or saline-extender BTS (control 4), 24 h past the procedures, using a microarray chip (GeneChip^®^ porcine gene 1.0 st array, Thermo Fisher Scientific) containing 25,000 gene level probe sets, followed by bioinformatics for enriched analysis of functional categories (GO terms) and restrictive bioinformatic analysis

## 2. Results

### 2.1. Total DEGs in the Peri-Ovulatory Uterine Tract

**Figure 1:**
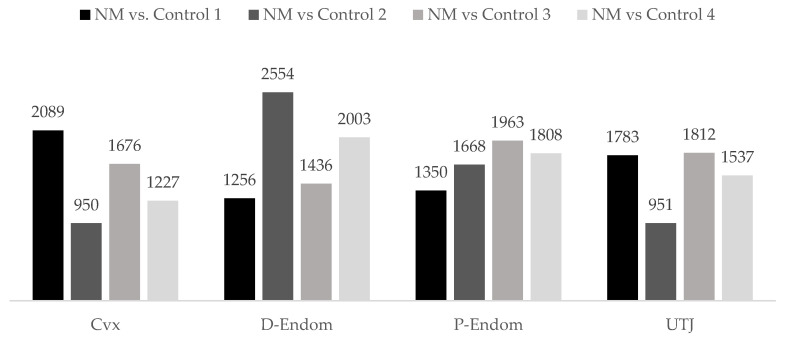
Differential expression of annotated genes in the mucosa of the uterus (endocervix (Cvx), endometrium (distal: D-Endom or proximal: P-Endom) and the utero-tubal junction (UTJ)) of sows induced by natural mating (NM) when compared to cervical AI of different contents; control 1: semen, control 2: seminal plasma from the sperm-peak fraction, control 3: seminal plasma from the entire ejaculate, control 4: sham–AI with BTS extender; negative control. The numbers represent the number of differentially expressed genes (*p*-value < 0.05).

The microarray analyses showed that natural mating (NM) altered the expression of annotated genes (950–2554) in the different anatomic uterine mucosal segments (Cvx-UTJ) when compared to the different contents of cervical AIs (controls 1–4), 24 h after exposure (Figure 1).

### 2.2. DEGs in the Peri-Ovulatory Uterine Tract, Only Affected by Copulation

**Figure 2:**
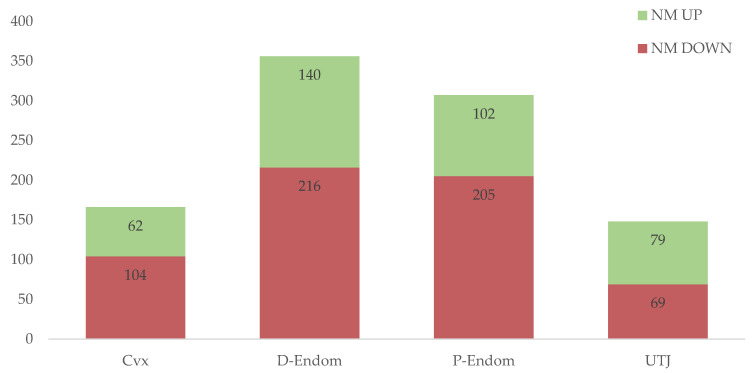
Differential expression (up- and downregulation) of annotated genes solely affected by copulation in the mucosa of the sow uterus (endocervix (Cvx), endometrium (distal: D-Endom or proximal: P-Endom) and the utero-tubal junction (UTJ)). The numbers represent the number of differentially expressed genes (*p*-value < 0.05).

Figure 2 summarizes the numbers of up- and downregulated annotated DEGs solely affected by copulation in the mucosa of the sow uterus, distributed per segments; endocervix (Cvx), endometrium (distal: D-Endom or proximal: P-Endom) and the utero-tubal junction (UTJ). The highest number of transcriptomic changes induced by copulation was registered in the Distal part of the endometrium (D-Endom; DEGs = 356), followed by the Proximal part (P-Endom; DEGs = 307), endocervix (Cvx; DEGs = 166) and the UTJ (DEGs = 148) (Figure 2). A complete list of these copulation-affected up- and downregulated DEGs in the different mucosal segments appears in Supplementary Tables S1–S4.

### 2.3. Copulation Altered Transcripts Common to All Uterine Segments (Cvx to UTJ)

**Figure 3:**
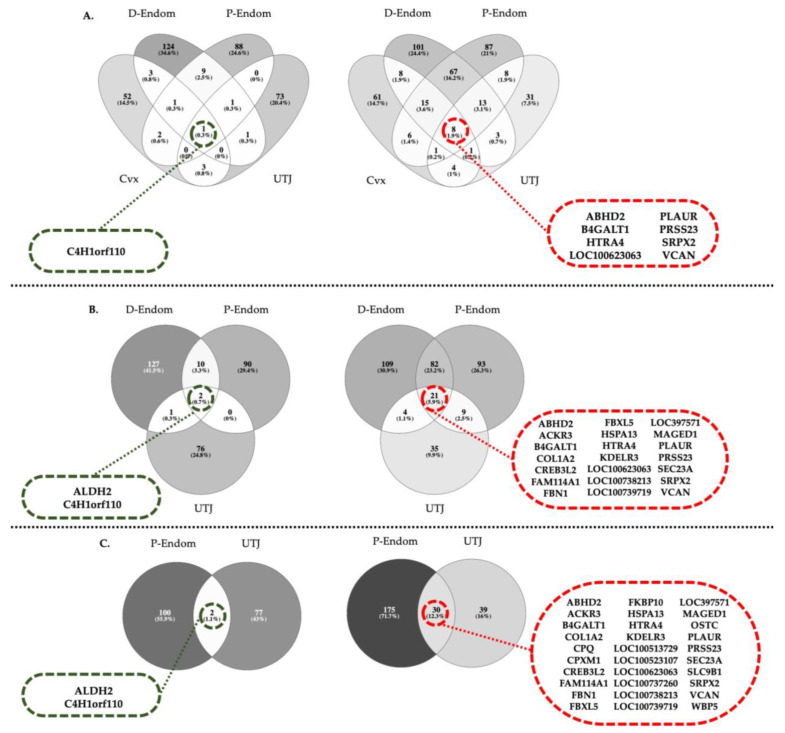
(**A**–**C**) Venn diagrams depicting in discontinuous rings the numbers and acronyms of copulation-induced, co-expressed DEGs shared by the different mucosal segments of the sow uterus (endocervix (Cvx), endometrium (distal: D-Endom or proximal: P-Endom) and the utero-tubal junction (UTJ), as listed in Supplementary Tables S1–S4. The diagrams include (**A**) comparisons of all four segments, (**B**) when endocervix was excluded or (**C**) when excluding endocervix and distal endometrium. Upregulated genes in green and downregulated in red.

The Venn diagrams in Figure 3A–C depict the numbers of copulation-induced, co-expressed DEGs shared among uterine segments listed in Supplementary Tables S1–S4, including all four segments (Figure 3A), excluding endocervix (Figure 3B) or excluding endocervix and distal endometrium (Figure 3C). Only one DEG transcript was commonly upregulated while 8 were downregulated among all four mucosal segments examined (Figure 3A). Excluding the endocervix, the number of upregulated and downregulated transcripts were 2 and 21, respectively (Figure 3B). Lastly, comparing the most proximal segments (P-Endom and UTJ), 2 genes were upregulated and 30 genes were downregulated (Figure 3C).

### 2.4. Biologic Meaning of Differentially Expressed Genes Induced by Copulation

**Figure 4:**
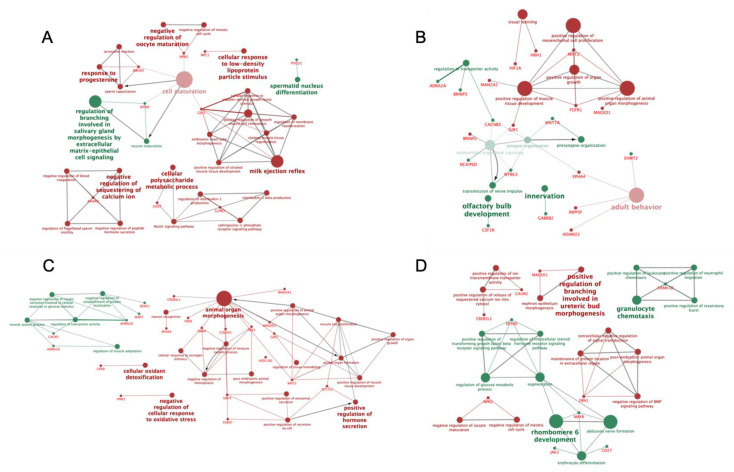
Schematic representation of biologic terms and pathways of selected DEGs, among different mucosal segments of the sow endocervix (Cvx; (**A**)), endometrium (distal: D-Endom (**B**) or proximal: P-Endom (**C**)) and the utero-tubal junction (UTJ; (**D**) 24 h past copulation. The analysis of overrepresented functional categories was performed using the Cytoscape v3.0.0 application ClueGo v2.0.3. Terms are functionally grouped based on shared genes (kappa score) and are shown in different colors (green: up-regulated; red: down-regulated). The size of the nodes indicates the degree of significance, where the biggest nodes correspond to highest significance and the intensity of the nodes indicates the amount of genes involved in that specific term, where the more intense correspond to the highest number of genes found. The following ClueGo parameters were used: GO tree levels, 1–3 (first level = 0); minimum number of genes, 1; *p*-value correction, Benjamini–Hochberg, terms with *p* < 0.05, GO term connection restriction (kappa score), 0.4; GO term grouping, initial group size of 1. The resulting network was modified; that is, some redundant and non-informative terms were deleted and the network manually rearranged.

From the general set of DEGs solely induced by copulation, listed in Supplementary Tables S1–S4, we selected 92 DEGs significantly involved in different pathways, biologic and molecular processes, with potential roles in reproductive processes, including neuroendocrine responses, hormone regulation, uterine contractility, angiogenesis and vascular remodeling, among others. The gene acronyms of this subset are listed in Table 1 while the biologic terms and pathways in which those genes are involved are represented in Figure 4. The complete list of all copulation-induced DEGs, KEGG pathway-categorized by Biologic processes and Molecular function are listed in Supplementary Tables S5–S7.

## 3. Discussion

The present study explored the exclusive action of the act of copulation on gene expression of different mucosal uterine segments (endocervix, distal or proximal endometrium and the sperm reservoir UTJ) in pre-ovulatory estrous sows 24 h post mating. The total DEG-database compared natural mating with a boar with each of the experimental control AI-procedures including cervical deposition of semen, SP or the saline extender (BTS), in order to discriminate the effect of each of these cells/fluids from the actual influence of act of copulation. Subtracting all genes present in each control from the NM, resulted in a DEG-database including transcripts solely related to copulation.

After a 24 h period, copulation per *se* induced an overrepresentation of transcripts related to neuroendocrine responses in the different segments of the uterus and the UTJ mucosae. The highest amount of DEGs was found in the endocervix and the distal and proximal parts of the uterine horn (D-Endom and P-Endom). These findings are not surprising since the uterus is widely known to act as a sensory transducer, converting the mechanical exteroceptive stimulus of copulation into neural electrical signals through the pelvic nerve [21]. To date, the literature points to vagino-cervical stimulation—which also occurs during AI—as the main agent responsible for generating electrical signals ascending from the cervix and reaching much of the limbic system and the hypothalamus, where several areas containing neurons receptive to vagino-cervical input have been identified [22]. Moreover, pigs are reproductively known for the boar delivering a large ejaculate (150–300 mL), which distends the lumen of the long (up to one meter each) uterine horns, stimulating the pressure receptors present in the uterine sub-mucosa and between the layers of the myometrium [23]. In swine, spontaneous uterine activity in sows increases naturally around estrus, with estrogens being key triggers for increased frequency of contractions [24], as well as playing a role in the transport and distribution of semen through the female genital tract [25,26].

Moreover, copulation induces, via the presence of the boar (pheromones) and the mechanical stimuli during mating, a release in oxytocin in the sow that increases uterine activity [24]. The levels of hypothalamic adiponectin and its receptors, present in the porcine hypothalamus in areas responsible for GnRH production and secretion, are highly dependent on the endocrine status during the various phases of the estrus cycle [27]. Steroid hormones play an important role in the regulation of cyclic changes in the uterus and preparation of intrauterine environment, inhibition of uterine contractions, cell proliferation and apoptosis and modulation of secretory activity of the uterus, among other events [28], including the action of glucocorticoids regulating intrauterine events [29].

We hereby report that the act of copulation in pigs, which involves the presence and mounting by the boar maintaining the standing reflex displayed by the sow, but also vagino-cervical stimulation and mechanical distention of the lumen, has the potential of inducing relevant neuro-endocrine molecular responses that contributes to successful fertilization by favoring uterine contractions that control sperm transport to the sperm reservoir at the UTJ.

Interestingly, a common upregulation of the α2-adrenergic receptor (*ADRA2A*) was found in D-Endom and P-Endom in response to mating, while α1-adrenergic receptor (*ADRA1A*) was upregulated just in P-Endom. *ADRA1A* and *ADRA2A* encode G protein-coupled receptors (GPCRs) that inhibit the activity of membrane-associated adenylyl cyclase (mACs) and their production of cAMP [30]. Besides being relevant during estrous, the number of α1&2-adrenoreceptors increases thereafter, under progesterone dominance in pigs [31] and several other species [32,33,34,35].

Another neuroendocrine mechanism controlling uterine contractility is the γ-Amino-butyric acid (*GABA*) signaling pathway. *GABA*, an inhibitory neurotransmitter in the mammalian brain, is alongside GABA G protein-coupled receptors (GPCRs), playing relevant roles in ovaries [36,37,38,39], oviducts [38], oocytes [39], the uterus [40] and the placenta [41]. Stimulation of GABAB receptors, which control Ca2+ and/or K+ channels [42], have been reported to tonically enchase contractions of uterine strips [43,44]. In the present study, GABA receptor subunit β2 gene (*GABRB2*) was upregulated in D-Endom after copulation, indicating relevance in porcine.

Moreover, the calcium channel, voltage-dependent, β2 subunit (*CACNB2*), a neuroendocrine-related gene, was also upregulated in the D-Endom tissue in response to copulation. *CACNB2* is one of the four homologous genes coding for the auxiliary Cavβ subunits, which are important modulators of the Ca(2+) channel activity, implicated in smooth muscle contraction [45]. Their upregulation suggests that 24 h after copulation, mechanisms related to sperm transport to the oviduct or even cleansing of surplus semen are activated, mostly in the distal region of the pig uterus. Other genes involved in uterine contractility were found overexpressed in response to mating, e.g., *WNT7A* (upregulated in D-Endom) a ligand of the WNT family that plays a critical role in the development and morphogenesis of uterine smooth muscle and mucosal glands [46] during the crucial sperm transport, respectively blastocyst nurture and implantation in the adult uterus [47]. *WNT7A* is also responsible for changes in the levels of sex steroid hormones in the female reproductive tract [48,49]. Lower myometrial proliferation is associated with a downregulation of *WNT7A* [46] and total ablation of *WNT7A* in the uterus has been shown to alter uterine development [50,51]. For instance, adult *WNT7A*-null mice are viable, but infertile and exhibit malformations in the female reproductive tract, including shortened and uncoiled oviducts, hypoplastic uterine horns and a vaginal septum tract [50].

Smooth muscle contractility is not the only relevant mechanism to be addressed. Protein fucosylation, which is a type of protein glycosylation, is one of the most common and important post-translational modifications involved in uterine angiogenesis and vascular remodeling, major during the receptive metestrus period, when embryos are transported to the uterine cavity. Impaired angiogenesis results in pregnancy pathologies or pregnancy failure [52,53]. Fucosylation is incorporated as two major forms: N-fucosylation and O-fucosylation, which are catalyzed by fucosyltransferases (FUTs) and protein O-fucosyltransferases (poFUTs), respectively. The accumulated evidence shows that FUTs participate in sperm–oocyte recognition, uterine receptivity formation and trophoblast invasion at the fetal–maternal interface [54]. The gene encoding protein O-fucosyltransferase 1 (poFUT1) presented a higher expression in response to copulation in the D-Endom region of the uterus. poFUT1 has been closely related with endometrial angiogenesis and vascular remodeling [55]. Previous studies showed that, the expression of poFUT1 was higher in the endometrium of women during the secretory compared to the proliferative phase and in the endometrium of early pregnant women than in that of miscarriage patients [55,56]. An elevated level of poFUT1 was also observed in impregnated uteri compared to the non-impregnated uteri in mice [55,56]. Previous studies also demonstrated that poFUT1 expression was decreased in placental villi from miscarriage patients and silencing poFUT1 suppressed the proliferation and invasion of JAR cells through inactivating MAPK and PI3K/Akt-signaling pathways [57,58,59]. Thus, the upregulation of poFUT1 registered in the present study may relate to an increased uterine capillary irrigation of the uterine lamina propria in order to increase uterine blood flow under the epithelial lining, which will ultimately be delivering oxygen and nutrients to the epitheliochorial pig placenta [60].

Furthermore, Netrin 4 (NTN4), which was found to be upregulated in the Cvx after mating, is a member of the heterogeneous family of laminin-related proteins that also participates in the regulation of angiogenesis in several tissues including embryos [61,62]. In addition, Netrins functions include essential contributions to regulating cell–cell and cell-matrix adhesion, tissue morphogenesis and the maintenance of appropriate cell–cell interactions [63] all of which are implicated in gamete interaction and early embryo development [64,65]. All these findings pose the idea that, although fertility after AI is comparable to that achieved by natural mating, the latter appears to induce physiological mechanisms of endometrial receptiveness more efficiently, not necessarily through semen influence, but even initiated by copulation stimulus.

A significant downregulation of *ABHD2* was found in all tissues examined (Cvx-UTJ) in relation to the sole act of mating. ABHD2 is a serine hydrolase that belongs to the subgroup of the α,β-hydrolase fold-containing proteins involved in the control of the sperm hyperactivation via progesterone and CatSper Ca2+ channels [13,66,67,68,69,70,71,72]. The fact that this sperm-hyperactivating gene was downregulated along the entire uterus suggests that these molecular signals are essential for reproduction in a spatiotemporal specific manner, being partially regulated by the endometrium to perhaps avoid premature sperm capacitation.

Another gene found downregulated in response to copulation was the erythropoietin-producing hepatocellular receptor A4 (*EPHA1*), downregulated in D-Endom and P-Endom tissues. *EPHA1* is expressed by endometrial epithelial cells and blastocysts, playing an important role facilitating embryo implantation [73]. In domestic animals, expression of *EPHA1* increases in the endometrium during the peri-implantation period of pregnancy [74,75], and it seems hereby affected by the act of copulation; alongside Colony-stimulating factor-1 receptor gene (*CSF1R*) which on the other hand appeared upregulated in D-Endom. The gene also appears to play key roles in cell-to-cell interactions, gamete receptivity and embryo implantation [76]. Although it seems reasonable that mechanisms ruling embryo implantation are kept downstream as early as 24 h post insemination, we found that copulation (rather that only the entry of semen or SP in the female genital tract), is capable of eliciting a robust response regarding the physiological mechanisms during the reproductive process.

Copulation also downregulated the expression of *FGFR1* gene in D-Endom. The secretion of gonadotropin-releasing hormone (GnRH), which regulates the synthesis and release of the gonadotropin hormones (LH and FSH) from the pituitary, depends upon multiple signaling mechanisms, including sex-steroid feedback regulation and fibroblast growth factor (FGF)-signaling [77]. *FGFR1* encodes for type 1 FGF receptor, which is a cell surface receptor of the tyrosine kinase family, expressed in several reproductive tissues [78]. Transgenic mice with specific gain-of function mutations in *FGFR1* exhibit delayed puberty and decreased number of GnRH neurons in the hypothalamus [79]. These findings suggest that pregnancies not achieved by natural mating may be prone to long-term impairment in reproductive performance. Longitudinal comparisons of sows being repeatedly bred via natural mating vs conventional AI over many pregnancies are thus required to disclose whether the above comment can be true. Unfortunately, most pigs today are only bred via AI, with the exception of the extensive, field managed pigs (i.e., Iberian pigs) or wild boar, which display high fertility.

There was an interesting co-expression of several transcripts within different segments of the genital tract. For instance, *ALDH2* (Aldehyde dehydrogenase) was upregulated in D-Endom, P-Endom and UTJ. ALDH is a detoxifying enzyme that plays a fundamental role in determining sperm longevity and motility [80]. ALDH2 has been determined as the most important isoform of this family responsible for reducing reactive-oxygen species (ROS) generation by sperm mitochondria, thus reducing cell apoptosis [81,82,83]. Its presence has been reported in several cells/tissues as spermatozoa, placenta or cervix [84,85,86], but, to the best of our knowledge, this is the first report to demonstrate *ALDH2* gene expression in the entire endometrium and UTJ of pigs and its activation in response to the act of copulation, indicating that a reduction of ROS-generation induced by an upregulation of *ALDH2* could be triggered as early as 24 h after copulation independently of the presence of the well-known antioxidant seminal plasma [87].

A general downregulation of *ACKR3*, *COL1A2* (D-Endom-UTJ), *B4GAT1* and *VCAN* (all segments) was found in response to mating. The expression of atypical chemokine receptors (ACKRs) has been reported at the maternal–fetal interface in humans and mice [88,89] and in the bovine and porcine endometrium during the estrus cycle and early pregnancy [90,91], specifically displaying high expression during the estrous cycle to be dramatically downregulated during early pregnancy [91]. It is thus logical to suggest, based on the results of the present study, that uterine *ACKR3* may be responsible of reducing chemokine activity in the pig endometrium towards the establishment of a state of maternal sperm tolerance [92]. By the same line of reasoning, a downregulation of collagen alpha-2 (I) chain (*COL1A2*) may contribute to prepare the endometrium to the presence of hemi-allogeneic embryos, as it occurs in other species [93,94]. The fact that copulation downregulated β-1,4-galactosyltransferase 1 (*B4GALT1*) during this period should not be surprising. Although detected in uterine epithelial cells [95], *B4GALT1* is upregulated in relation to cell adhesion [96], including pre-implantation embryos [97] or ectoplacental cone cells [98], events occurring far later than the period explored in the present study. A downregulated Versican (*VCAN*) would reduce, probably under the action of steroid hormones during estrus [99], the display of inflammatory responses post mating by a generic alteration of the expression of cytokines and chemokines [100], a downregulation that could be favoring maternal sperm tolerance.

## 4. Materials and Methods

### 4.1. Ethics Statement

Animal handling was performed in compliance with the European Community (Directive 2010/63/EU) and current Swedish legislation (SJVFS 2017:40). It followed the reduction principle of the 3Rs on animal experimentation (replacement, reduction and refinement) while maintaining enough numbers of biologic replicates from distinct animals, reliable estimates of variation among samples within procedures that could distinguish true differences between conditions. The experiments were approved in advance by the “Regional committee for ethical approval of animal experiments” (Linköpings Djurförsöksetiska nämnd) in Linköping, Sweden (permits no. 75–12, no. ID1400 and Dnr 03416-2020 (26/03/2020)).

### 4.2. Experimental Design

**Figure 5:**
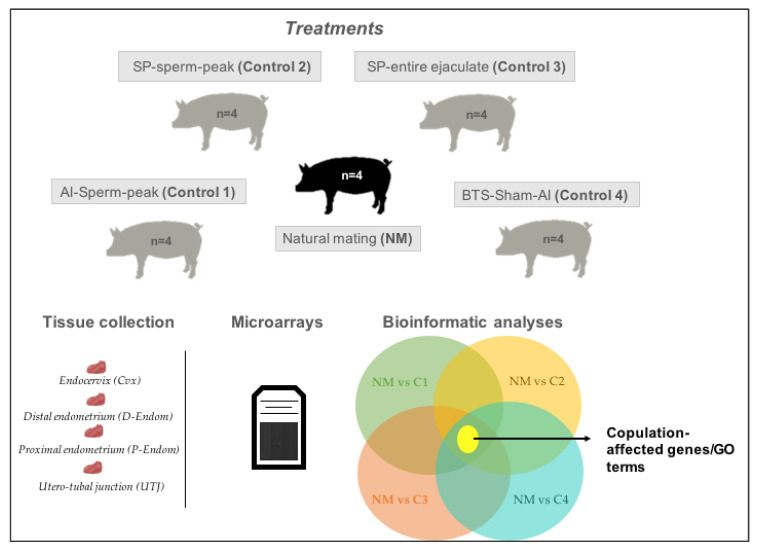
Graphical illustration of the experimental design. Experimental sow groups compared natural mating (NM) with cervical artificial insemination (AI) of semen of the sperm-peak fraction (control 1), of sperm-free SP from the sperm-peak fraction (control 2), of sperm-free SP from the whole ejaculate (control 3) or of saline BTS semen extender (control 4, sham–AI). Mucosal samples from the endocervix (Cvx), endometrium (distal: D-Endom or proximal: P-Endom) and the utero-tubal junction (UTJ) were surgically retrieved 24 h later and analyzed. NM was first compared to all experimental controls to isolate those DEGs not affected by semen, SP or AI-vagino-cervical stimulation, e.g., those being exclusively affected by copulation.

This study aimed to investigate the molecular patterns of the pre/peri-ovulatory porcine mucosa of the uterus (endo-cervix, endometrium and UTJ) in response to the act of copulation per se, e.g., isolated from the effects of semen or sperm-free SP, using microarray technology (Figure 5). Multiparous sows displaying standing estrus in the presence of a boar (*n* = 20), were randomly subjected to: Natural mating (NM; *n* = 4) or selective cervical AIs; of the sperm-peak ejaculate fraction (control 1; *n* = 4, 50 mL), of sperm-free SP from the sperm-peak ejaculate fraction (control 2; *n* = 4, 50 mL), of sperm-free SP from the entire ejaculate (control 3; *n* = 4, 50 mL) or of Beltsville thawing solution; BTS (control 4; *n* = 4, 50 mL, sham–AI). Mucosal samples of specific segments of the uterus: endocervix (Cvx), distal endometrium (D-Endom), proximal endometrium (P-Endom) and the utero-tubal junction (UTJ), were retrieved during surgery under general anesthesia 24 h after each procedure, snap-frozen in liquid nitrogen (LN_2_) and stored at −80 °C until further analysis [4,9,16].

### 4.3. Animal Management

Weaned sows (parity 1–3) and young mature boars (9–11 months) of proven fertility, were recruited from a controlled breeding farm (Swedish Landrace breed). Throughout all experiments, animals were handled carefully to avoid any unnecessary stress. The animals were individually kept in separate pens at the translational medicine center (TMC/CBR-3) of Linköping University under controlled temperature and light regimes (12 h: 12 h light/dark cycle). Pigs were fed with commercial feedstuff (Lantmännen, Stockholm, Sweden) according to national standards provided with water ad libitum and receiving the same management. Detection of estrus of the sows was performed twice daily, beginning one day after weaning. Sows were tested by experienced personnel for standing estrus reflex by applying back-pressure while sows had snout-to-snout contact with adjacent located mature boars. When sows showed standing estrus reflex they were considered to be on the first day of behavioral estrus and then used in this study. Boar ejaculates and the specific sperm-peak ejaculate fraction (the first 10 mL of the sperm-rich fraction), were both collected using the gloved-hand method. Only semen with at least 70% motile and 75% morphologically normal-looking spermatozoa immediately after collection was used.

### 4.4. Natural Mating and Artificial Insemination of Semen, Sperm-Free SP and BTS-Extender

Sows were at the onset of estrus and randomly allowed to be mounted by an individual boar or cervically inseminated/infused with the sperm-peak ejaculate fraction (containing 25% of the total spermatozoa of the ejaculate, control 1) or SP-harvested after double centrifugation at 1500× *g* for 10 min and microscopically checked for absence of spermatozoa, either from the sperm-peak fraction (control 2) or from the whole ejaculate (control 3). Cervical infusions of BTS-extender constituted control 4 (sham–AI).

### 4.5. Collection of Samples of the Internal Genital Tract

All sows were subjected to mid-ventral laparotomies to collect mucosal tissue samples 24 h after the procedures (pre/peri-ovulation period), as previously described [101]. Briefly, sows were sedated by the i.m. administration of a mixture of 5 mg dexmedetomidine (dexdomitor, Orion Pharma Animal Health, Sollentuna, Sweden) and 100 mg tiletamine hydrochloride/zolazepam hydrochloride (Zoletil vet, Virbac A/S, Kolding, Denmark) followed by anesthesia induced IV with sodium thiopental (Abbot Scandinavia AB, Solna, Sweden, 7 mg/kg BW) and maintained with isoflurane (3.5%–5%, Baxter Medical AB, Kista, Sweden) administered via a tracheal cuffed tube by a close-circuit PVC-ventilator (Servo ventilator 900 D, Siemens-Elema AB, Solna, Sweden). Peripheral blood plasma was analyzed (ELISA) for progesterone (P_4_) and estradiol 17ß (E_2_) contents, confirming the sows were in peri-ovulatory estrus (P_4_ = 0.77 ± 0.35 pg/mL; E_2_ ranging 294.2–376.50 ± 27.76 pg/mL, *p* > 0.05 among sows/groups). Mucosal samples were immediately retrieved from the endocervix (Cvx), distal uterine horn (D-Endom), proximal uterine horn (P-Endom) and the utero-tubal junction (UTJ), plunged in LN_2_ and stored at −80 °C in RNAlater (Ambion, Thermo Fisher Scientific Baltics UAB, Vilnius, Lithuania) until analyzed.

### 4.6. Total Transcriptome Analysis

Total RNA was isolated from tissue samples using Trizol reagent (Invitrogen, Carlsbad, CA, USA) and quality assessment was performed using an Agilent 2100 Bioanalyzer (Agilent Technologies, Santa Clara, CA, USA) according to the manufacturer’s instructions. The RNA integrity number (RIN) values obtained were in the range of 8 to 10, which guarantied the homogeneity and high quality of the samples. Equal amounts of total RNA (250 ng) from each sample were used to make cDNA using GeneChip^®^ whole transcript plus reagent kit (Affymetrix, Santa Clara, CA, USA, 25,000 probes) following the manufacturer protocol. cDNA was then hybridized and loaded on the array chip (GeneChip^®^ porcine gene 1.0 st array, Affymetrix, Inc., 3420 Central Expressway, Santa Clara, CA 95051, USA), incubated at 45 °C under 60 rotations per min, for 16 h. The hybridized cartridge array chip was then unloaded and subjected to washing and staining using a GeneChip^®^ Fluidics Station 450 (Affymetrix), to be finally scanned using the Affymetrix GeneChip^®^ scanner GCS3000 [101].

### 4.7. Microarray Data and Enrichment Analyses of the Degs Unrelated to Semen or SP Influence

The array data were examined using Partek Genomics Suite 7.0 (Partek, St. Louis, MO, USA), following normalization using the robust multichip average RMA method [102]. Differentially expressed genes (DEGs) for NM and control groups (1–4) were compared in between by a one-way ANOVA, setting fold changes (FC) >1 or <−1 and *p*-value < 0.05 to identify those DEGs exclusively affected by copulation and not affected semen, SP or vaginal–cervical stimulation. To obtain a biologically meaningful overview of the significantly modified transcripts, an enrichment analysis was performed. Analysis of overrepresented gene ontology (GO) terms and pathways were performed with the DAVID (database for annotation, visualization and integrated discover) and KEGG (Kyoto encyclopedia of genes and genomes) databases. Graphic illustration of overrepresented GO terms was produced with the Cytoscape v3.0.0 application CluePedia v2.0.3 [103].

## 5. Conclusions

Altogether, the present findings point out that copulation enhances, per se—and even when isolated from the known effects of semen influence—reproductive processes related to sperm transport, but also to sperm tolerance, uterine receptivity and fertilization success. Most relevant is the finding that these influences may be different from those triggered by the simple deposition of semen or seminal plasma as mimicked by cervical artificial insemination.

## Figures and Tables

**Table 1:** Subset of selected differentially expressed genes (DEGs, *p* < 0.05, up- or downregulated) that copulation in sows induced 24 h later in the endocervix (Cvx), the endometrium (distal: D-Endom or proximal: P-Endom) and the utero-tubal junction (UTJ), classified by relevant biologic terms and pathways according to DAVID database.

Cvx		D-Endom			P-Endom			UTJ	
UP	DOWN	UP	DOWN		UP	DOWN		UP	DOWN
*FAM107A*	*ABHD2*	*ADRA2A*	*ABDH2*	*HSD11B1*	*ADRA1A*	*ABDH2*	*OLFM3*	*CAMK1D*	*ABDH2*
*HDAC9*	*ANXA5*	*BRINP3*	*ADAM22*	*HTR2A*	*ADRA2A*	*ADAM22*	*P2RX7*	*CD27*	*CALM2*
*MYLIP*	*BRINP2*	*CACNB2*	*AGTR1*	*INPP5 F*	*ASGR2*	*AGTR1*	*RARA*	*EP300*	*CREB3 L2*
*NTN4*	*DLGAP2*	*CSF1R*	*ALCAM*	*MAGED1*	*CALM1*	*BRINP2*	*SDC2*	*JAK2*	*FBN1*
*PYGO1*	*GJA1*	*EHMT2*	*BRINP2*	*MAN2A1*	*DEAF1*	*COL3A1*	*SDC4*	*MAFB*	*MAGED1*
	*IL6ST*	*GABRB2*	*COL3A1*	*ME1*	*MAPT*	*CREB3L1*	*TAC3*	*MYLIP*	*MAPK10*
	*LRP12*	*NCKIPSD*	*COL4A1*	*NPR2*	*PACSIN3*	*CREB3 L2*	*TCF7 L2*	*ZNF217*	*NPR2*
	*MAN2A1*	*NIPSNAP1*	*CREB3 L2*	*OLFM3*	*RXFP4*	*EPHA4*	*VCAN*		*TP23*
	*NPC1*	*NTRK3*	*EPHA4*	*PTGFR*	*VIPR1*	*FBN1*	*VNN1*		*VCAN*
	*NPR2*	*POFUT1*	*FBN1*	*RCAN2*		*FRZB*	*WNT2*		
	*NPY2R*	*RARG*	*FGFR1*	*RPS6KA6*		*GJA1*			
	*S1PR3*	*TBL1X*	*FRZB*	*S1PR3*		*GPX8*			
	*VCAN*	*TENM2*	*GJA1*	*SDC2*		*HSD11B1*			
		*WNT7A*	*GPX8*	*TCF7 L2*		*IRAK4*			
			*HIF1A*	*VCAN*		*MAGED1*			
			*HRH1*	*WNT2*		*MAN2A1*			

## Supplementary Materials

Supplementary materials can be found at https://www.mdpi.com/1422-0067/21/15/5477/s1.
