# Supplementary material for: Does the Act of Copulation per se, without Considering Seminal Deposition, Change the Expression of Genes in the Porcine Female Genital Tract?
Source: Int J Mol Sci. 2020 Jul 31;21(15):5477. doi: 10.3390/ijms21155477 (PMC7432858; doi:10.3390/ijms21155477)
Supplement: Supplementary file 1 [file ijms-21-05477-s001.pdf]

Supplementary Table 1. List (akronym and description) of differentially expressed genes (P < 0.05) solely related to copulation, in in pig endocervix (Cvx) up- or down-regulated.

UP	Description	*DOWN*	
AGT	angiotensinogen (serpin peptidase inhibitor, clade A, member 8)	*ABHD2*	abhydrolase domain containing 2(ABHD2)
AKAP13	A kinase (PRKA) anchor protein 13; A-kinase anchor protein 13-like	*AGPAT2*	1-acylglycerol-3-phosphate O-acyltransferase 2(AGPAT2)
AKAP9	A kinase (PRKA) anchor protein 9; A-kinase anchor protein 9-like	*ANXA5*	annexin A5(ANXA5)
ANKRD33B	ankyrin repeat domain 33B; ankyrin repeat domain-containing protein 33B-like	*ATP6V0C*	ATPase H+ transporting V0 subunit c(ATP6V0C)
C2H5orf45	chromosome 2 open reading frame, human C5orf45	*B3GALT2*	beta-1,3-galactosyltransferase 2(B3GALT2)
C4H1orf110	chromosome 4 open reading frame, human C1orf110	*B4GALT1*	beta-1,4-galactosyltransferase 1(B4GALT1)
CCDC130	coiled-coil domain containing 130(CCDC130)	*BRINP2*	BMP/retinoic acid inducible neural specific 2(BRINP2)
CCDC92	coiled-coil domain containing 92(CCDC92)	*C7H14orf119*	chromosome 7 open reading frame, human C14orf119(C7H14orf119)
CDKAL1	CDK5 regulatory subunit associated protein 1 like 1(CDKAL1)	*C8H4orf32*	chromosome 8 open reading frame, human C4orf32
COLCA2	colorectal cancer associated 2(COLCA2)	*CD47*	CD47 molecule
CPD	carboxypeptidase D(CPD)	*CDH7*	cadherin 7(CDH7)
CRTC1	CREB regulated transcription coactivator 1(CRTC1)	*CMPK2*	cytidine/uridine monophosphate kinase 2(CMPK2)
DACH1	dachshund family transcription factor 1	*CMTR1*	cap methyltransferase 1; cap-specific mRNA (nucleoside-2-O-)-methyltransferase 1-like; uncharacterized LOC102158493 (CMTR1; LOC106507681; LOC102158493)
DAZAP2	DAZ associated protein 2(DAZAP2)	*CPXM1*	carboxypeptidase X, M14 family member 1(CPXM1)
DDX17	DEAD-box helicase 17(DDX17)	*CXCL10*	C-X-C motif chemokine ligand 10(CXCL10)
DECR2	2,4-dienoyl-CoA reductase 2, peroxisomal(DECR2)	*CYP51*	cytochrome P450, family 51, subfamily A, polypeptide 1(CYP51)
ENPP5	ectonucleotide pyrophosphatase/phosphodiesterase 5 (putative)(ENPP5)	*DCBLD1*	discoidin, CUB and LCCL domain containing 1(DCBLD1)
FAM107A	family with sequence similarity 107 member A(FAM107A)	*DLGAP2*	DLG associated protein 2(DLGAP2)
FMO1	flavin containing monooxygenase 1(FMO1)	*DPT*	dermatopontin(DPT)
FUNDC2	FUN14 domain containing 2(FUNDC2)	*DRAM1*	DNA damage regulated autophagy modulator 1(DRAM1)
GGA2	golgi associated, gamma adaptin ear containing, ARF binding protein 2(GGA2)	*ENPEP*	glutamyl aminopeptidase(ENPEP)
HDAC9	histone deacetylase 9(HDAC9)	*FAM111A*	family with sequence similarity 111 member A(FAM111A)
IGF1R	insulin like growth factor 1 receptor(IGF1R)	*FDPS*	farnesyl diphosphate synthase(FDPS)
ING1	inhibitor of growth family, member 1	*FGFBP1*	fibroblast growth factor binding protein 1(FGFBP1)
KCNQ4	potassium voltage-gated channel subfamily Q member 4(KCNQ4)	*GARS*	glycyl-tRNA synthetase(GARS)
LOC100153094	glutathione S-transferase theta-1(LOC100153094)	*GCNT3*	glucosaminyl (N-acetyl) transferase 3, mucin type(GCNT3)
LOC100153559	leucine rich adaptor protein 1-like	*GJA1*	gap junction protein alpha 1(GJA1)
LOC100520903	zinc finger protein 300-like(LOC100520903)	*HMGCR*	3-hydroxy-3-methylglutaryl-CoA reductase(HMGCR)
LOC100521052	mitochondrial inner membrane protease subunit 2(LOC100521052)	*HMGCS1*	3-hydroxy-3-methylglutaryl-CoA synthase 1(HMGCS1)
LOC100523068	CUB and sushi domain-containing protein 2-like; uncharacterized LOC102159420	*HTRA4*	HtrA serine peptidase 4(HTRA4)
LOC100627616	calcium-activated potassium channel subunit alpha-1-like; potassium channel, calcium activated large conductance subfamily M alpha, member 1	*IER3*	immediate early response 3(IER3)
LOC100738970	CXXC-type zinc finger protein 4(LOC100738970)	*IFI44*	interferon induced protein 44(IFI44)
LOC102159576	trinucleotide repeat-containing gene 6B protein-like; uncharacterized LOC106510264; uncharacterized LOC100516974	*IFRD1*	interferon related developmental regulator 1(IFRD1)
LOC102166095	nuclear receptor coactivator 2-like(LOC102166095)	*IL6ST*	interleukin 6 signal transducer(IL6ST)
LOC102166102	methylcytosine dioxygenase TET2-like; methylcytosine dioxygenase TET2	*INSIG1*	insulin induced gene 1(INSIG1)
MIR181B-1	microRNA 181b-1(MIR181B-1)	*ISG12(A)*	putative ISG12(a) protein(ISG12(A))
MTURN	myosin regulatory light chain interacting protein(MYLIP)	*KSR1*	kinase suppressor of ras 1(KSR1)
MYLIP	myosin regulatory light chain interacting protein	*LOC100152428*	multidrug resistance-associated protein 1-like(LOC100152428)
NCOR2	nuclear receptor corepressor 2(NCOR2)	*LOC100153163*	MHC class I polypeptide-related sequence B-like
NDRG4	NDRG family member 4(NDRG4)	*LOC100154959*	olfactory receptor 4K3(LOC100154959)
NTN4	netrin 4(NTN4)	*LOC100157647*	pleckstrin homology domain-containing family A member 1; pleckstrin homology domain-containing family A member 1-like

PCBD1	pterin-4 alpha-carbinolamine dehydratase 1(PCBD1)	*LOC100158037*	probable ATP-dependent RNA helicase DDX60(LOC100158037)
PDZRN4	PDZ domain containing ring finger 4(PDZRN4)	*LOC100511841*	UDP-glucuronosyltransferase 1-10(LOC100511841)
PNISR	PNN interacting serine and arginine rich protein(PNISR)	*LOC100517053*	integrin alpha-3(LOC100517053)
PRKCQ	protein kinase C theta(PRKCQ)	*LOC100517940*	apolipoprotein L3-like
PYGO1	pygopus family PHD finger 1(PYGO1)	*LOC100518083*	E3 ISG15--protein ligase HERC5(LOC100518083)
RETSAT	retinol saturase(RETSAT)	*LOC100523138*	transmembrane protein 88-like(LOC100523138)
RFXAP	regulatory factor X associated protein(RFXAP)	*LOC100525346*	poliovirus receptor homolog(LOC100525346)
RNF149	ring finger protein 149(RNF149)	*LOC100623063*	protein FAM69A-like; family with sequence similarity 69, member A (LOC100623063; FAM69A)
SESN3	sestrin 3(SESN3)	*LOC100625846*	histone deacetylase 8-like
SLC9A3R2	SLC9A3 regulator 2(SLC9A3R2)	*LOC100737852*	peptidyl-prolyl cis-trans isomerase C-like
SPHKAP	SPHK1 interactor, AKAP domain containing	*LOC100739412*	histidine decarboxylase-like
SREK1	splicing regulatory glutamine/lysine-rich protein 1	*LOC100739844*	semaphorin-3A-like(LOC100739844)
THBS3	thrombospondin 3(THBS3)	*LOC102161647*	trichohyalin
TMEM131	transmembrane protein 131(TMEM131)	*LOC102165708*	uncharacterized LOC102165708
TMEM141	transmembrane protein 141(TMEM141)	*LOC106504198*	protein S100-A5-like; protein S100-A5
TMEM213	transmembrane protein 213(TMEM213)	*LRP12*	LDL receptor related protein 12(LRP12)
TSHZ2	teashirt zinc finger homeobox 2(TSHZ2)	*LRP8*	LDL receptor related protein 8(LRP8)
VAMP2	vesicle associated membrane protein 2(VAMP2)	*MAN2A1*	mannosidase alpha class 2A member 1(MAN2A1)
ZBTB44	zinc finger and BTB domain containing 44(ZBTB44)	*MAPK6*	mitogen-activated protein kinase 6(MAPK6)
ZNF239	zinc finger protein 239	*MFSD2A*	major facilitator superfamily domain containing 2A(MFSD2A)
ZNF318	zinc finger protein 318	*MIR671*	microRNA mir-671; chondroitin polymerizing factor 2 (MIR671; CHPF2)
		MMP3	matrix metallopeptidase 3 (stromelysin 1, progelatinase)(MMP3)
		MTMR11	myotubularin related protein 11(MTMR11)
		MX1	myxovirus (influenza virus) resistance 1, interferon-inducible protein p78 (mouse)(MX1)
		NCOA4	nuclear receptor coactivator 4(NCOA4)
		NPC1	NPC intracellular cholesterol transporter 1(NPC1)
		NPR2	natriuretic peptide receptor B/guanylate cyclase B (atrionatriuretic peptide receptor B)(NPR2)
		NPY2R	neuropeptide Y receptor Y2(NPY2R)
		NT5C2	5'-nucleotidase, cytosolic II(NT5C2)
		OLFML3	olfactomedin like 3(OLFML3)
		PALM2	paralemmin 2(PALM2)
		PANK3	pantothenate kinase 3(PANK3)
		PAPSS2	3'-phosphoadenosine 5'-phosphosulfate synthase 2(PAPSS2)
		PGD	phosphogluconate dehydrogenase(PGD)
		PLAT	plasminogen activator, tissue type(PLAT)
		PLAUR	plasminogen activator, urokinase receptor(PLAUR)
		PLOD2	procollagen-lysine,2-oxoglutarate 5-dioxygenase 2(PLOD2)
		PRSS23	protease, serine 23(PRSS23)
		RASD1	ras related dexamethasone induced 1(RASD1)
		REC8	REC8 meiotic recombination protein(REC8)
		RIPK3	receptor interacting serine/threonine kinase 3(RIPK3)
		RNF24	ring finger protein 24
		RSAD2	radical S-adenosyl methionine domain containing 2(RSAD2)
		S1PR3	sphingosine-1-phosphate receptor 3(S1PR3)
		SEPHS2	selenophosphate synthetase 2(SEPHS2)
		SGPP2	sphingosine-1-phosphate phosphatase 2(SGPP2)
		SLA-7	MHC class I antigen 7(SLA-7)
		SLC25A17	solute carrier family 25 member 17(SLC25A17)
		SLC30A7	solute carrier family 30 member 7(SLC30A7)
		SNX31	sorting nexin 31(SNX31)
		SQLE	squalene epoxidase(SQLE)
		SRPX2	sushi repeat containing protein, X-linked 2(SRPX2)
		STARD4	StAR related lipid transfer domain containing 4(STARD4)
		TAPBP	TAP binding protein(TAPBP)
		TDG	thymine-DNA glycosylase(TDG)

		THBS1	thrombospondin 1(THBS1)
		TM4SF1	transmembrane 4 L six family member 1(TM4SF1)
		TRIB2	tribbles pseudokinase 2
		UBA7	ubiquitin like modifier activating enzyme 7(UBA7)
		UBASH3B	ubiquitin associated and SH3 domain containing B(UBASH3B)
		VCAN	versican(VCAN)
		XPNPEP1	X-prolyl aminopeptidase 1(XPNPEP1)
		ZBP1	Z-DNA binding protein 1(ZBP1)

Supplementary Table 2. List (akronym and description) of differentially expressed genes (P < 0.05) solely related to copulation, in pig distal endometrium (DistUt) up- or down-regulated.

UP		*DOWN*	
AAR2	AAR2 splicing factor homolog(AAR2)	*ABCB1*	ATP-binding cassette, sub-family B (MDR/TAP), member 1(ABCB1)
ABCA9	ATP binding cassette subfamily A member 9(ABCA9)	*ABHD2*	abhydrolase domain containing 2(ABHD2)
ADCK2	aarF domain containing kinase 2(ADCK2)	*ACKR3*	atypical chemokine receptor 3(ACKR3)
ADRA2A	adrenoceptor alpha 2A(ADRA2A)	*ADAM22*	ADAM metallopeptidase domain 22(ADAM22)
ALDH2	aldehyde dehydrogenase 2 family (mitochondrial)(ALDH2)	*ADAMTS3*	ADAM metallopeptidase with thrombospondin type 1 motif, 3
ALDH6A1	aldehyde dehydrogenase 6 family member A1(ALDH6A1)	*ADAMTS5*	ADAM metallopeptidase with thrombospondin type 1 motif 5(ADAMTS5)
ANXA9	annexin A9(ANXA9)	*AFF1*	AF4/FMR2 family member 1(AFF1)
AQP9	aquaporin 9(AQP9)	*AFF2*	AF4/FMR2 family member 2(AFF2)
ARRB2	arrestin beta 2(ARRB2)	*AGR2*	anterior gradient 2, protein disulphide isomerase family member(AGR2)
ATP2A3	ATPase sarcoplasmic/endoplasmic reticulum Ca2+ transporting 3(ATP2A3)	*AGTR1*	angiotensin II receptor type 1(AGTR1)
ATP2C2	ATPase secretory pathway Ca2+ transporting 2(ATP2C2)	*ALCAM*	activated leukocyte cell adhesion molecule(ALCAM)
ATP5E	ATP synthase, H+ transporting, mitochondrial F1 complex, epsilon subunit(ATP5E)	*ANXA5*	annexin A5(ANXA5)
BRINP3	BMP/retinoic acid inducible neural specific 3(BRINP3)	*ARF4*	ADP ribosylation factor 4(ARF4)
C4H1orf110	chromosome 4 open reading frame, human C1orf110	*ARHGAP28*	Rho GTPase activating protein 28(ARHGAP28)
CACNB2	calcium voltage-gated channel auxiliary subunit beta 2(CACNB2)	*ARL6IP5*	ADP ribosylation factor like GTPase 6 interacting protein 5(ARL6IP5)
CAP2	CAP, adenylate cyclase-associated protein, 2 (yeast)(CAP2)	*ARMCX3*	armadillo repeat containing, X-linked 3(ARMCX3)
CAPN15	calpain 15(CAPN15)	*ATP8B2*	ATPase phospholipid transporting 8B2(ATP8B2)
CAPN5	calpain 5(CAPN5)	*B3GALT2*	beta-1,3-galactosyltransferase 2(B3GALT2)
CASP2	caspase 2, apoptosis-related cysteine peptidase	*B4GALT1*	beta-1,4-galactosyltransferase 1(B4GALT1)
CBX8	chromobox 8(CBX8)	*BICC1*	BicC family RNA binding protein 1(BICC1)
CCDC124	coiled-coil domain containing 124(CCDC124)	*BRINP2*	BMP/retinoic acid inducible neural specific 2(BRINP2)
CDC25B	cell division cycle 25B(CDC25B)	*CALU*	calumenin(CALU)
CDC25C	cell division cycle 25C(CDC25C)	*CC2D2B*	coiled-coil and C2 domain containing 2B(CC2D2B)
CEP68	centrosomal protein 68(CEP68)	*CD248*	CD248 molecule(CD248)
CHKA	choline kinase alpha(CHKA)	*CDH11*	cadherin 11(CDH11)
CNOT1	CCR4-NOT transcription complex subunit 1(CNOT1)	*CFL2*	cofilin 2(CFL2)
CNOT2	CCR4-NOT transcription complex subunit 2(CNOT2)	*CHSY3*	chondroitin sulfate synthase 3(CHSY3)
COQ4	coenzyme Q4(COQ4)	*CLDN11*	claudin 11(CLDN11)
CSF1R	colony stimulating factor 1 receptor(CSF1R)	*CLINT1*	clathrin interactor 1(CLINT1)
CUL4A	cullin 4A(CUL4A)	*COL1A2*	collagen type I alpha 2 chain(COL1A2)
CYFIP2	cytoplasmic FMR1 interacting protein 2(CYFIP2)	*COL3A1*	collagen type III alpha 1 chain(COL3A1)
DGAT1	diacylglycerol O-acyltransferase 1(DGAT1)	*COL4A1*	collagen type IV alpha 1 chain(COL4A1)
DGUOK	deoxyguanosine kinase(DGUOK)	*COL5A2*	collagen type V alpha 2 chain(COL5A2)
DHRS3	dehydrogenase/reductase 3(DHRS3)	*COL6A3*	collagen type VI alpha 3 chain(COL6A3)
DHX32	DEAH-box helicase 32 (putative)(DHX32)	*CREB3L2*	cAMP responsive element binding protein 3 like 2(CREB3L2)
DOPEY2	dopey family member 2(DOPEY2)	*CRISPLD2*	cysteine rich secretory protein LCCL domain containing 2(CRISPLD2)
EHMT2	euchromatic histone lysine methyltransferase 2(EHMT2)	*CRYGS*	crystallin gamma S(CRYGS)
FAH	fumarylacetoacetate hydrolase(FAH)	*DCLK1*	doublecortin-like kinase 1
FAM149A	family with sequence similarity 149 member A(FAM149A)	*DCN*	decorin(DCN)
FAM84A	family with sequence similarity 84 member A(FAM84A)	*DDX26B*	DEAD/H (Asp-Glu-Ala-Asp/His) box polypeptide 26B
FCGR1A	Fc fragment of IgG, high affinity Ia, receptor (CD64)(FCGR1A)	*DMP1*	dentin matrix acidic phosphoprotein 1(DMP1)

FMO2	flavin containing monooxygenase 2(FMO2)	*DNAJB9*	DnaJ heat shock protein family (Hsp40) member B9(DNAJB9)
GABRB2	gamma-aminobutyric acid type A receptor beta2 subunit(GABRB2)	*EDEM3*	ER degradation enhancing alpha-mannosidase like protein 3(EDEM3)
GARNL3	GTPase activating Rap/RanGAP domain like 3(GARNL3)	*EIF2AK3*	eukaryotic translation initiation factor 2 alpha kinase 3(EIF2AK3)
GMPR	guanosine monophosphate reductase(GMPR)	*ELL2*	elongation factor for RNA polymerase II 2(ELL2)
GNMT	glycine N-methyltransferase(GNMT)	*ENPEP*	glutamyl aminopeptidase(ENPEP)
GPA33	glycoprotein A33(GPA33)	*EPHA4*	EPH receptor A4(EPHA4)
GSTK1	glutathione S-transferase kappa 1(GSTK1)	*ERLEC1*	endoplasmic reticulum lectin 1(ERLEC1)
HADH	hydroxyacyl-CoA dehydrogenase(HADH)	*ERO1A*	endoplasmic reticulum oxidoreductase 1 alpha(ERO1A)
HSP90AB1	heat shock protein 90 alpha family class B member 1(HSP90AB1)	*EXOC5*	exocyst complex component 5(EXOC5)
HSPE1	heat shock protein family E (Hsp10) member 1(HSPE1)	*FAM114A1*	family with sequence similarity 114 member A1(FAM114A1)
IDH3G	isocitrate dehydrogenase 3 (NAD(+)) gamma(IDH3G)	*FAM171B*	family with sequence similarity 171 member B(FAM171B)
IGSF8	immunoglobulin superfamily member 8(IGSF8)	*FAM213A*	family with sequence similarity 213 member A(FAM213A)
IL1RAPL2	interleukin 1 receptor accessory protein like 2(IL1RAPL2)	*FAP*	fibroblast activation protein alpha(FAP)
ING1	inhibitor of growth family, member 1	*FBN1*	fibrillin 1(FBN1)
IQCE	IQ motif containing E(IQCE)	*FBXL5*	F-box and leucine rich repeat protein 5(FBXL5)
KBTBD4	kelch repeat and BTB domain containing 4(KBTBD4)	*FBXO28*	F-box protein 28(FBXO28)
KCNMB4	potassium large conductance calcium-activated channel, subfamily M, beta member 4(KCNMB4)	*FGF2*	fibroblast growth factor 2(FGF2)
KCTD1	potassium channel tetramerization domain containing 1(KCTD1)	*FGFR1*	fibroblast growth factor receptor 1(FGFR1)
KCTD12	potassium channel tetramerization domain containing 12(KCTD12)	*FKBP7*	FK506 binding protein 7(FKBP7)
KRT19	keratin 19(KRT19)	*FOXP2*	forkhead box P2(FOXP2)
LOC100152489	olfactory receptor 13C8(LOC100152489)	*FRZB*	frizzled-related protein(FRZB)
LOC100153966	activator of 90 kDa heat shock protein ATPase homolog 1(LOC100153966)	*GCLM*	glutamate-cysteine ligase modifier subunit(GCLM)
LOC100155148	mitochondrial intermediate peptidase	*GCNT3*	glucosaminyl (N-acetyl) transferase 3, mucin type(GCNT3)
LOC100513474	platelet-activating factor acetylhydrolase IB subunit gamma(LOC100513474)	*GJA1*	gap junction protein alpha 1(GJA1)
LOC100513647	uncharacterized LOC100513647	*GNAI1*	G protein subunit alpha i1(GNAI1)
LOC100514456	E3 ubiquitin-protein ligase RNF144A-like	*GORAB*	golgin, RAB6-interacting
LOC100520426	leucine carboxyl methyltransferase 1 (LOC100520426; LCMT1)	*GPX8*	glutathione peroxidase 8 (putative)(GPX8)
LOC100521052	mitochondrial inner membrane protease subunit 2(LOC100521052)	*GREB1*	growth regulation by estrogen in breast cancer 1
LOC100521189	potassium channel subfamily K member 12	*GUCY1B3*	guanylate cyclase 1, soluble, beta 3(GUCY1B3)
LOC100521617	myelin and lymphocyte protein(LOC100521617)	*HCFC2*	host cell factor C2(HCFC2)
LOC100624445	gamma-glutamylcyclotransferase-like(LOC100624445)	*HELZ*	helicase with zinc finger(HELZ)
LOC100626307	DNA primase large subunit-like(LOC100626307)	*HIF1A*	hypoxia inducible factor 1, alpha subunit (basic helix-loop-helix transcription factor)(HIF1A)
LOC100736752	palmitoyltransferase ZDHHC13-like	*HOXA10*	homeobox A10(HOXA10)
LOC100737730	integrator complex subunit 4-like	*HRH1*	histamine receptor H1(HRH1)
LOC100738939	nuclear envelope integral membrane protein 1(LOC100738939)	*HSBP1L1*	heat shock factor binding protein 1 like 1(HSBP1L1)
LOC100738957	B9 domain-containing protein 2-like	*HSD11B1*	hydroxysteroid 11-beta dehydrogenase 1(HSD11B1)
LOC102160697	ADAMTS-like protein 1	*HSD17B11*	hydroxysteroid 17-beta dehydrogenase 11(HSD17B11)
LOC106504056	two pore calcium channel protein 1-like; two pore calcium channel protein 1 (LOC106504056; LOC100151806; LOC102167997)	*HSPA13*	heat shock protein family A (Hsp70) member 13(HSPA13)
LOC106504128	R3H domain-containing protein 1-like(LOC106504128)	*HTR2A*	5-hydroxytryptamine receptor 2A(HTR2A)
LRRC1	leucine rich repeat containing 1(LRRC1)	*HTRA4*	HtrA serine peptidase 4(HTRA4)
MAP4K2	mitogen-activated protein kinase kinase kinase kinase 2(MAP4K2)	*IFRD1*	interferon related developmental regulator 1(IFRD1)
MPP1	membrane palmitoylated protein 1(MPP1)	*IKBIP*	IKBKB interacting protein(IKBIP)
MSLN	mesothelin(MSLN)	*INPP5F*	inositol polyphosphate-5-phosphatase F(INPP5F)

MTMR7	myotubularin related protein 7(MTMR7)	*ISG12(A)*	putative ISG12(a) protein(ISG12(A))
NCKIPSD	NCK interacting protein with SH3 domain(NCKIPSD)	*KCTD9*	potassium channel tetramerization domain containing 9(KCTD9)
NCOR2	nuclear receptor corepressor 2(NCOR2)	*KDELR3*	KDEL endoplasmic reticulum protein retention receptor 3(KDELR3)
NDUFA10	NADH:ubiquinone oxidoreductase subunit A10(NDUFA10)	*KIFAP3*	kinesin associated protein 3(KIFAP3)
NDUFV1	NADH:ubiquinone oxidoreductase core subunit V1(NDUFV1)	*LOC100152428*	multidrug resistance-associated protein 1-like(LOC100152428)
NFIX	nuclear factor I X(NFIX)	*LOC100153395*	fibronectin type III domain-containing protein 3B; fibronectin type III domain-containing protein 3B-like; uncharacterized LOC106504256 (LOC100153395; LOC100519977; LOC100625867; LOC106504256)
NIPSNAP1	nipsnap homolog 1 (C. elegans)(NIPSNAP1)	*LOC100157647*	pleckstrin homology domain-containing family A member 1; pleckstrin homology domain-containing family A member 1-like (LOC100157647; LOC100625949)
NIPSNAP3A	nipsnap homolog 3A(NIPSNAP3A)	*LOC100157704*	olfactory receptor 1L8-like(LOC100157704)
NTRK3	neurotrophic receptor tyrosine kinase 3(NTRK3)	*LOC100511013*	protein GREB1
NUDT14	nudix hydrolase 14(NUDT14)	*LOC100512644*	uncharacterized LOC100512644(LOC100512644)
OLFML2A	olfactomedin like 2A(OLFML2A)	*LOC100515061*	probable glutathione peroxidase 8; glutathione peroxidase 8 (putative) (LOC100515061; GPX8)
PCBD1	pterin-4 alpha-carbinolamine dehydratase 1(PCBD1)	*LOC100515833*	FYVE, RhoGEF and PH domain-containing protein 6-like; FYVE, RhoGEF and PH domain-containing protein 6 (LOC100515833; LOC100517825)
PDF	peptide deformylase (mitochondrial)(PDF)	*LOC100516665*	protocadherin gamma-C3; protocadherin gamma-A11; protocadherin gamma-A2-like; protocadherin gamma-B5-like (100736974; LOC106509588; LOC100737660)
PDGFD	platelet derived growth factor D(PDGFD)	*LOC100517015*	cAMP-dependent protein kinase inhibitor beta-like(LOC100517015)
PLD2	phospholipase D2(PLD2)	*LOC100517735*	chondroitin sulfate N-acetylgalactosaminyltransferase 1(LOC100517735)
PLXDC1	plexin domain containing 1(PLXDC1)	*LOC100517820*	proteasome activator complex subunit 4(LOC100517820)
POFUT1	protein O-fucosyltransferase 1(POFUT1)	*LOC100517940*	apolipoprotein L3-like
POLR1A	RNA polymerase I subunit A(POLR1A)	*LOC100518433*	tetratricopeptide repeat protein 39A
POLR1D	RNA polymerase I subunit D(POLR1D)	*LOC100518541*	A disintegrin and metalloproteinase with thrombospondin motifs 19-like; A disintegrin and metalloproteinase with thrombospondin motifs 19 (LOC100518541; LOC100518181)
PRPSAP1	phosphoribosyl pyrophosphate synthetase associated protein 1(PRPSAP1)	*LOC100518720*	chondroitin sulfate synthase 3(LOC100518720)
PRR3	proline rich 3(PRR3)	*LOC100520066*	geranylgeranyl transferase type-1 subunit beta
RAB3IP	RAB3A interacting protein(RAB3IP)	*LOC100520265*	protocadherin-9(LOC100520265)
RARG	retinoic acid receptor gamma(RARG)	*LOC100525491*	rho GTPase-activating protein 7
RNF167	ring finger protein 167(RNF167)	*LOC100525519*	anoctamin-4
RNF4	ring finger protein 4(RNF4)	*LOC100623063*	protein FAM69A-like; family with sequence similarity 69, member A (LOC100623063; FAM69A)
RREB1	ras responsive element binding protein 1(RREB1)	*LOC100736857*	microphthalmia-associated transcription factor-like(LOC100736857)
RUFY1	RUN and FYVE domain containing 1(RUFY1)	*LOC100737666*	collagen alpha-1(XVI) chain(LOC100737666)
SAMD1	sterile alpha motif domain containing 1	*LOC100737852*	peptidyl-prolyl cis-trans isomerase C-like
SLC15A1	solute carrier family 15 member 1(SLC15A1)	*LOC100738213*	collagen alpha-1(I) chain
SLC35F1	solute carrier family 35 member F1(SLC35F1)	*LOC100738373*	epidermal growth factor receptor kinase substrate 8-like(LOC100738373)
SLC44A1	solute carrier family 44 member 1(SLC44A1)	*LOC100738400*	ubiquitin-conjugating enzyme E2 E2
SLC7A7	solute carrier family 7 member 7(SLC7A7)	*LOC100739719*	tetraspanin-6(LOC100739719)
SPR	sepiapterin reductase (7,8-dihydrobiopterin:NADP+ oxidoreductase)(SPR)	*LOC100739763*	LOC100739763
SRCAP	Snf2 related CREBBP activator protein(SRCAP)	*LOC100739844*	semaphorin-3A-like(LOC100739844)
STARD10	StAR related lipid transfer domain containing 10(STARD10)	*LOC102157535*	40S ribosomal protein S6 pseudogene
STRIP2	striatin interacting protein 2(STRIP2)	*LOC102161149*	melanoma inhibitory activity protein 3-like (LOC102161149; LOC100520445)
STX10	syntaxin 10(STX10)	*LOC102161405*	uncharacterized LOC102161405
TBL1X	transducin beta like 1X-linked(TBL1X)	*LOC102162328*	uncharacterized LOC102162328
TDP1	tyrosyl-DNA phosphodiesterase 1(TDP1)	*LOC102163342*	A disintegrin and metalloproteinase with thrombospondin motifs 19-like
TENM2	teneurin transmembrane protein 2(TENM2)	*LOC102164530*	collagen alpha-1(XV) chain
TET3	tet methylcytosine dioxygenase 3(TET3)	*LOC102164566*	uncharacterized LOC102164566
TMEM164	transmembrane protein 164	*LOC102164569*	pro-neuregulin-1, membrane-bound isoform-like; pro-neuregulin-3, membrane-bound isoform-like (LOC102164569; LOC100620584)
TRAP1	TNF receptor associated protein 1(TRAP1)	*LOC102165708*	uncharacterized LOC102165708
TRIM9	tripartite motif containing 9(TRIM9)	*LOC102165740*	uncharacterized LOC102165740

UBP1	upstream binding protein 1 (LBP-1a)(UBP1)	*LOC106504075*	proton myo-inositol cotransporter; uncharacterized LOC100520492 (LOC106504075; LOC100520492)
UBQLN4	ubiquilin 4(UBQLN4)	*LOC106504251*	multidrug resistance protein 1-like (LOC106504251; LOC100522455)
UNC13D	unc-13 homolog D(UNC13D)	*LOC106508947*	collagen alpha-1(XII) chain-like; collagen alpha-1(XII) chain (LOC106508947; LOC106508948; LOC100156689)
USP2	ubiquitin specific peptidase 2(USP2)	*LOC397380*	uncharacterized LOC397380
VEGFB	vascular endothelial growth factor B(VEGFB)	*LOC397571*	collagen alpha-1(I) chain
WNT7A	Wnt family member 7A(WNT7A)	*LRRC17*	leucine rich repeat containing 17; leucine-rich repeat-containing protein 17-like; leucine-rich repeat-containing protein 17 (LRRC17; LOC100513808; LOC100513611)
WRAP73	WD repeat containing, antisense to TP73(WRAP73)	*LYSMD3*	LysM domain containing 3(LYSMD3)
ZBTB7C	zinc finger and BTB domain containing 7C(ZBTB7C)	*MAGED1*	MAGE family member D1(MAGED1)
ZCCHC14	zinc finger CCHC-type containing 14(ZCCHC14)	*MAN2A1*	mannosidase alpha class 2A member 1(MAN2A1)
ZMYM3	zinc finger MYM-type containing 3(ZMYM3)	*MARCH-5*	membrane-associated ring finger (C3HC4) 5
ZNF185	zinc finger protein 185 (LIM domain); zinc finger protein 185-like (ZNF185; LOC100737297)	*MARCKS*	myristoylated alanine rich protein kinase C substrate(MARCKS)
ZSWIM1	zinc finger SWIM-type containing 1(ZSWIM1)	*ME1*	malic enzyme 1(ME1)
		MFAP2	microfibrillar associated protein 2(MFAP2)
		MGAT4D	MGAT4 family member D(MGAT4D)
		MIR671	microRNA mir-671; chondroitin polymerizing factor 2 (MIR671; CHPF2)
		MMP1	matrix metallopeptidase 1(MMP1)
		MMP3	matrix metallopeptidase 3 (stromelysin 1, progelatinase)(MMP3)
		MON2	MON2 homolog, regulator of endosome-to-Golgi trafficking(MON2)
		MORC3	MORC family CW-type zinc finger 3(MORC3)
		MOSPD1	motile sperm domain containing 1(MOSPD1)
		MRC2	mannose receptor C type 2(MRC2)
		MXRA5	matrix remodeling associated 5(MXRA5)
		NCEH1	neutral cholesterol ester hydrolase 1(NCEH1)
		NPR2	natriuretic peptide receptor B/guanylate cyclase B (atrionatriuretic peptide receptor B)(NPR2)
		OLFM3	olfactomedin 3(OLFM3)
		OMD	osteomodulin(OMD)
		PDIA3	protein disulfide isomerase family A member 3(PDIA3)
		PDP1	pyruvate dehyrogenase phosphatase catalytic subunit 1(PDP1)
		PELO	pelota homolog (Drosophila)(PELO)
		PLAU	plasminogen activator, urokinase(PLAU)
		PLAUR	plasminogen activator, urokinase receptor(PLAUR)
		PLEKHA8	pleckstrin homology domain containing A8(PLEKHA8)
		PLOD2	procollagen-lysine,2-oxoglutarate 5-dioxygenase 2(PLOD2)
		PMAIP1	phorbol-12-myristate-13-acetate-induced protein 1(PMAIP1)
		PPAP2A	protein phosphatase 3 catalytic subunit gamma(PPP3CC)
		PPP3CC	protein phosphatase 4 regulatory subunit 4(PPP4R4)
		PPP4R4	PR/SET domain 1(PRDM1)
		PRDM1	prion protein(PRNP)
		PRNP	protease, serine 23(PRSS23)
		PRSS23	prostaglandin F receptor(PTGFR)
		PTGFR	RAS like family 12(RASL12)
		RASL12	regulator of calcineurin 2(RCAN2)
		RCAN2	RNA exonuclease 2(REXO2)
		REXO2	Rho family GTPase 3(RND3)
		RND3	ring finger protein 11(RNF11)
		RNF11	ribosomal protein S6 kinase A6(RPS6KA6)
		RNF24	sphingosine-1-phosphate receptor 3(S1PR3)
		RPS6KA6	syndecan 2(SDC2)
		S1PR3	Sec23 homolog A, coat complex II component(SEC23A)
		SDC2	Sec23 homolog B, coat complex II component(SEC23B)
		SEC23A	SEC63 homolog, protein translocation regulator(SEC63)
		SEC23B	solute carrier family 30 member 7(SLC30A7)
		SEC63	solute carrier family 38 member 1(SLC38A1)

		SELK	solute carrier family 38 member 2(SLC38A2)
		SLC30A7	solute carrier family 39 member 7(SLC39A7)
		SLC38A1	solute carrier family 41 member 2(SLC41A2)
		SLC38A2	solute carrier organic anion transporter family, member 2A1(SLCO2A1)
		SLC39A7	secreted protein, acidic, cysteine-rich (osteonectin)(SPARC)
		SLC41A2	sushi repeat containing protein, X-linked 2(SRPX2)
		SLCO2A1	STEAP2 metalloreductase(STEAP2)
		SPARC	secreted protein, acidic, cysteine-rich (osteonectin); SPARC (SPARC; LOC100737020; LOC100514136)
		SRPX2	sushi-repeat containing protein, X-linked 2
		STEAP2	STEAP family member 2, metalloreductase; metalloreductase STEAP2 (STEAP2; LOC100624711)
		SVEP1	sushi, von Willebrand factor type A, EGF and pentraxin domain containing 1(SVEP1)
		TAC3	tachykinin 3(TAC3)
		TCF7L2	transcription factor 7 like 2(TCF7L2)
		TDG	thymine-DNA glycosylase(TDG)
		TGM2	transglutaminase 2(TGM2)
		TMEM170B	transmembrane protein 170B(TMEM170B)
		TMEM263	transmembrane protein 263(TMEM263)
		TMEM35	transmembrane protein 35
		TRIB2	tribbles pseudokinase 2
		TSPAN9	tetraspanin 9(TSPAN9)
		TTC39B	tetratricopeptide repeat domain 39B(TTC39B)
		TVP23B	trans-golgi network vesicle protein 23 homolog B(TVP23B)
		UBL3	ubiquitin like 3(UBL3)
		UCAL-P19	uterocalin P19(UCAL-P19)
		UGGT2	UDP-glucose glycoprotein glucosyltransferase 2(UGGT2)
		VCAN	versican(VCAN)
		VGLL3	vestigial like family member 3(VGLL3)
		VSTM4	V-set and transmembrane domain containing 4(VSTM4)
		WDR72	WD repeat domain 72
		WFDC9	WAP four-disulfide core domain 9(WFDC9)
		WIPI1	WD repeat domain, phosphoinositide interacting 1(WIPI1)
		WNT2	Wnt family member 2(WNT2)
		ZBTB38	zinc finger and BTB domain containing 38(ZBTB38)
		ZFAND5	zinc finger AN1-type containing 5(ZFAND5)
		ZNF516	zinc finger protein 516(ZNF516)

Supplementary Table 3. List (akronym and description) of differentially expressed genes (P < 0.05) solely related to copulation, in pig proximal endometrium (ProxUt) up- or down-regulated.

UP			*DOWN*	
ADHFE1	alcohol dehydrogenase, iron containing 1(ADHFE1)		*ABCB1*	ATP-binding cassette, sub-family B (MDR/TAP), member 1(ABCB1)
ADRA1A	adrenoceptor alpha 1A(ADRA1A)		*ABHD2*	abhydrolase domain containing 2(ABHD2)
ADRA2A	adrenoceptor alpha 2A(ADRA2A)		*ACKR3*	atypical chemokine receptor 3(ACKR3)
ALDH2	aldehyde dehydrogenase 2 family (mitochondrial)(ALDH2)		*ADAM12*	ADAM metallopeptidase domain 12(ADAM12)
ALOXE3	arachidonate lipoxygenase 3		*ADAM22*	ADAM metallopeptidase domain 22(ADAM22)
ANKRD23	ankyrin repeat domain 23(ANKRD23)		*ADAMTS9*	ADAM metallopeptidase with thrombospondin type 1 motif 9(ADAMTS9)
ASGR2	asialoglycoprotein receptor 2(ASGR2)		*ADD3*	adducin 3(ADD3)
C15H2orf72	chromosome 15 open reading frame, human C2orf72		*AFF2*	AF4/FMR2 family member 2(AFF2)
C1H15orf52	chromosome 1 open reading frame, human C15orf52		*AGR2*	anterior gradient 2, protein disulphide isomerase family member(AGR2)
C4H1orf110	chromosome 4 open reading frame, human C1orf110		*AGTR1*	angiotensin II receptor type 1(AGTR1)
CALM1	calmodulin 1(CALM1)		*AMMECR1*	Alport syndrome, mental retardation, midface hypoplasia and elliptocytosis chromosomal region gene 1(AMMECR1)
CD207	CD207 molecule, langerin(CD207)		*ATAD2B*	beta-1,3-galactosyltransferase 2(B3GALT2)
CELF4	CUGBP, Elav-like family member 4(CELF4)		*B3GALT2*	UDP-GlcNAc:betaGal beta-1,3-N-acetylglucosaminyltransferase 2(B3GNT2)
CEP250	centrosome-associated protein CEP250-like; centrosomal protein 250kDa (LOC100521166; CEP250)		*B3GNT2*	beta-1,4-galactosyltransferase 1(B4GALT1)
CHCHD10	coiled-coil-helix-coiled-coil-helix domain containing 10(CHCHD10)		*B4GALT1*	bone morphogenetic protein 1(BMP1)
CHST4	carbohydrate sulfotransferase 4(CHST4)		*BMP1*	BMP/retinoic acid inducible neural specific 2(BRINP2)
CNTROB	centrobin, centriole duplication and spindle assembly protein(CNTROB)		*BRINP2*	bone morphogenetic protein/retinoic acid inducible neural-specific 2
CRACR2A	calcium release activated channel regulator 2A		*C7H14orf119*	chromosome 7 open reading frame, human C14orf119(C7H14orf119)
CRYL1	crystallin lambda 1(CRYL1)		*CCDC160*	coiled-coil domain containing 160(CCDC160)
CTXN3	cortexin 3(CTXN3)		*CDH11*	cadherin 11(CDH11)
DDB1	damage specific DNA binding protein 1(DDB1)		*CHIC2*	cysteine rich hydrophobic domain 2(CHIC2)
DEAF1	DEAF1, transcription factor(DEAF1)		*CHSY3*	chondroitin sulfate synthase 3(CHSY3)
DGAT1	diacylglycerol O-acyltransferase 1(DGAT1)		*CLDN11*	claudin 11(CLDN11)
FAM117A	family with sequence similarity 117 member A(FAM117A)		*CLDND1*	claudin domain containing 1(CLDND1)
FIGF	c-fos induced growth factor (vascular endothelial growth factor D)		*CLIC2*	chloride intracellular channel 2(CLIC2)
GALK1	galactokinase 1(GALK1)		*CLINT1*	clathrin interactor 1(CLINT1)
GCM2	glial cells missing homolog 2 (Drosophila)		*COL1A2*	collagen type I alpha 2 chain(COL1A2)
GFRA1	GDNF family receptor alpha 1		*COL3A1*	collagen type III alpha 1 chain(COL3A1)
GPR26	G protein-coupled receptor 26(GPR26)		*COL7A1*	collagen type VII alpha 1 chain(COL7A1)
GRK1	G protein-coupled receptor kinase 1(GRK1)		*CPQ*	carboxypeptidase Q; carboxypeptidase Q-like (CPQ; LOC106508327; LOC102163803)
HOXD12	homeobox D12(HOXD12)		*CPXM1*	carboxypeptidase X, M14 family member 1(CPXM1)
HSF1	heat shock transcription factor 1(HSF1)		*CREB3L1*	cAMP responsive element binding protein 3 like 1(CREB3L1)
HSPB1	heat shock protein family B (small) member 1(HSPB1)		*CREB3L2*	cAMP responsive element binding protein 3 like 2(CREB3L2)
IL17D	interleukin 17D(IL17D)		*CRISPLD2*	cysteine rich secretory protein LCCL domain containing 2(CRISPLD2)
JADE3	jade family PHD finger 3(JADE3)		*CRYGS*	crystallin gamma S(CRYGS)
JAZF1	JAZF zinc finger 1(JAZF1)		*DAP*	death-associated protein
KCNQ4	potassium voltage-gated channel subfamily Q member 4(KCNQ4)		*DCLRE1A*	DNA cross-link repair 1A(DCLRE1A)
KHK	ketohexokinase(KHK)		*DCTD*	dCMP deaminase(DCTD)
LCN10	lipocalin 10(LCN10)		*DMP1*	dentin matrix acidic phosphoprotein 1(DMP1)
LIM2	lens intrinsic membrane protein 2(LIM2)		*DRAM1*	DNA damage regulated autophagy modulator 1(DRAM1)
LOC100152736	zinc finger protein 142(LOC100152736)		*ECE1*	endothelin converting enzyme 1(ECE1)
LOC100155148	mitochondrial intermediate peptidase		*ELF4*	E74 like ETS transcription factor 4(ELF4)
LOC100511353	A disintegrin and metalloproteinase with thrombospondin motifs 12		*ENPEP*	glutamyl aminopeptidase(ENPEP)
LOC100511598	WD repeat-containing protein 17-like		*ENTPD5*	ectonucleoside triphosphate diphosphohydrolase 5(ENTPD5)
LOC100513291	butyrophilin, subfamily 1, member; butyrophilin subfamily 3 member A2-like (LOC100513291; LOC106504090)		*EPAS1*	endothelial PAS domain protein 1(EPAS1)

LOC100513769	taste receptor type 2 member 16		*EPHA4*	EPH receptor A4(EPHA4)
LOC100515137	5-oxoprolinase-like(LOC100515137)		*ERCC6*	ERCC excision repair 6, chromatin remodeling factor(ERCC6)
LOC100515312	C2 calcium-dependent domain-containing protein 4D-like(LOC100515312)		*ERLEC1*	endoplasmic reticulum lectin 1(ERLEC1)
LOC100516511	olfactory receptor 1A1(LOC100516511)		*ERP44*	endoplasmic reticulum protein 44(ERP44)
LOC100516539	splicing factor 3A subunit 2(LOC100516539)		*EXOC5*	exocyst complex component 5(EXOC5)
LOC100517804	putative olfactory receptor 5AK3(LOC100517804)		*FAM111A*	family with sequence similarity 111 member A(FAM111A)
LOC100521466	olfactory receptor 52L1(LOC100521466)		*FAM114A1*	family with sequence similarity 114 member A1(FAM114A1)
LOC100524975	olfactory receptor 10H1(LOC100524975)		*FAM171B*	family with sequence similarity 171 member B(FAM171B)
LOC100626192	sodium-dependent proline transporter-like		*FAP*	fibroblast activation protein alpha(FAP)
LOC100627836	uncharacterized LOC100627836		*FBLN1*	fibulin 1(FBLN1)
LOC100737030	keratin, type I cytoskeletal 42		*FBN1*	fibrillin 1(FBN1)
LOC100737805	SUN domain-containing protein 1-like(LOC100737805)		*FBXL5*	F-box and leucine rich repeat protein 5(FBXL5)
LOC100737809	ras-specific guanine nucleotide-releasing factor RalGPS2-like; Ral GEF with PH domain and SH3 binding motif 2 (LOC100737809; RALGPS2)		*FGFBP1*	fibroblast growth factor binding protein 1(FGFBP1)
LOC100738970	CXXC-type zinc finger protein 4(LOC100738970)		*FGFR1OP2*	FGFR1 oncogene partner 2(FGFR1OP2)
LOC100739011	putative lipoyltransferase 2, mitochondrial(LOC100739011)		*FKBP10*	FK506 binding protein 10, 65 kDa
LOC102160697	ADAMTS-like protein 1		*FKBP7*	FK506 binding protein 7(FKBP7)
LOC102161977	ral guanine nucleotide dissociation stimulator-like (LOC102161977; LOC106504274)		*FRZB*	frizzled-related protein(FRZB)
LOC102162013	calcium-binding mitochondrial carrier protein SCaMC-3-like; solute carrier family 25 (mitochondrial carrier; phosphate carrier), member 23 (LOC102162013; SLC25A23)		*GCNT3*	glucosaminyl (N-acetyl) transferase 3, mucin type(GCNT3)
LOC102164149	uncharacterized LOC102164149		*GJA1*	gap junction protein alpha 1(GJA1)
LOC102164150	neuronal PAS domain-containing protein 2-like; neuronal PAS domain protein 2 (LOC102164150; NPAS2)		*GLB1L*	galactosidase beta 1 like(GLB1L)
LOC106506271	sodium channel protein type 1 subunit alpha-like		*GMNN*	geminin, DNA replication inhibitor(GMNN)
LOC106506828	uncharacterized LOC106506828; WAS/WASL interacting protein family, member 3 (LOC106506828; WIPF3)		*GNAI1*	G protein subunit alpha i1(GNAI1)
MAPT	microtubule associated protein tau(MAPT)		*GOLGA5*	golgin A5(GOLGA5)
MIR193A	microRNA mir-193a		*GORAB*	golgin, RAB6-interacting
MIR30A	microRNA mir-30a		*GPD2*	glycerol-3-phosphate dehydrogenase 2(GPD2)
MIR99B	microRNA mir-99b		*GPX8*	glutathione peroxidase 8 (putative)(GPX8)
MLXIP	MLX interacting protein(MLXIP)		*HOXA10*	homeobox A10(HOXA10)
MST1	macrophage stimulating 1(MST1)		*HSD11B1*	hydroxysteroid 11-beta dehydrogenase 1(HSD11B1)
MTMR7	myotubularin related protein 7(MTMR7)		*HSPA13*	heat shock protein family A (Hsp70) member 13(HSPA13)
OTOGL	otogelin like(OTOGL)		*HTRA4*	HtrA serine peptidase 4(HTRA4)
PACSIN3	protein kinase C and casein kinase substrate in neurons 3(PACSIN3)		*IER3*	immediate early response 3(IER3)
PCBD1	pterin-4 alpha-carbinolamine dehydratase 1(PCBD1)		*IFI27L2*	interferon, alpha-inducible protein 27-like 2
PGLYRP1	peptidoglycan recognition protein 1(PGLYRP1)		*IRAK4*	interleukin 1 receptor associated kinase 4(IRAK4)
PPAPDC1A	phosphatidic acid phosphatase type 2 domain containing 1A		*ISG12(A)*	putative ISG12(a) protein(ISG12(A))
RHBDL1	rhomboid like 1(RHBDL1)		*KDELR3*	KDEL endoplasmic reticulum protein retention receptor 3(KDELR3)
RHCG	Rh family C glycoprotein(RHCG)		*KDR*	kinase insert domain receptor(KDR)
RNF152	ring finger protein 152(RNF152)		*LMAN2*	lectin, mannose binding 2(LMAN2)
RREB1	ras responsive element binding protein 1(RREB1)		*LOC100152428*	multidrug resistance-associated protein 1-like(LOC100152428)
RTN4RL1	reticulon 4 receptor like 1(RTN4RL1)		*LOC100153395*	fibronectin type III domain-containing protein 3B; fibronectin type III domain-containing protein 3B-like; uncharacterized LOC106504256 (LOC100153395; LOC100519977; LOC100625867; LOC106504256)
RXFP4	relaxin/insulin like family peptide receptor 4(RXFP4)		*LOC100157647*	pleckstrin homology domain-containing family A member 1; pleckstrin homology domain-containing family A member 1-like (LOC100157647; LOC100625949)
SDR16C5	short chain dehydrogenase/reductase family 16C, member 5(SDR16C5)		*LOC100512381*	brain-specific angiogenesis inhibitor 3
SERINC5	serine incorporator 5(SERINC5)		*LOC100512644*	uncharacterized LOC100512644(LOC100512644)
SERTM1	serine rich and transmembrane domain containing 1(SERTM1)		*LOC100512873*	antileukoproteinase(LOC100512873)
SLC35F1	solute carrier family 35 member F1(SLC35F1)		*LOC100513074*	serine protease inhibitor Kazal-type 9(LOC100513074)
SP7	Sp7 transcription factor(SP7)		*LOC100513729*	hippocampus abundant transcript 1 protein

SPR	sepiapterin reductase (7,8-dihydrobiopterin:NADP+ oxidoreductase)(SPR)		*LOC100515061*	glutathione peroxidase 8 (putative); probable glutathione peroxidase 8 (GPX8; LOC100515061)
SSMEM1	serine rich single-pass membrane protein 1(SSMEM1)		*LOC100516193*	uncharacterized LOC100516193; spermatogenesis associated, serine-rich 2 (LOC100516193; SPATS2)
STK11	serine/threonine kinase 11(STK11)		*LOC100517015*	cAMP-dependent protein kinase inhibitor beta-like(LOC100517015)
THAP10	THAP domain containing 11(THAP11)		*LOC100517569*	lysine-specific demethylase 4C-like (LOC100622077; LOC100517569)
THAP11	thymosin beta 4, X-linked(TMSB4X)		*LOC100518433*	tetratricopeptide repeat protein 39A
TMSB4X	thymosin beta 4, X-linked(TMSB4X)		*LOC100518720*	chondroitin sulfate synthase 3(LOC100518720)
VIPR1	vasoactive intestinal peptide receptor 1(VIPR1)		*LOC100520265*	protocadherin-9(LOC100520265)
XAB2	XPA binding protein 2(XAB2)		*LOC100523107*	melanoma-associated antigen D4(LOC100523107)
XK	X-linked Kx blood group(XK)		*LOC100523528*	protein CASC4-like(LOC100523528)
ZNF576	zinc finger protein 576(ZNF576)		*LOC100525121*	neuron navigator 1(LOC100525121)
ZNF697	zinc finger protein 697(ZNF697)		*LOC100620725*	piezo-type mechanosensitive ion channel component 2
ZSWIM1	zinc finger SWIM-type containing 1(ZSWIM1)		*LOC100623063*	protein FAM69A-like; family with sequence similarity 69, member A (LOC100623063; FAM69A)
			LOC100623707	phosphatidylinositol 3-kinase regulatory subunit alpha-like(LOC100623707)
			LOC100623976	neuron navigator 1
			LOC100736973	ras-related protein Rap-1b(LOC100736973)
			LOC100737260	astrotactin-1-like
			LOC100737666	collagen alpha-1(XVI) chain(LOC100737666)
			LOC100737681	contactin-associated protein-like 2(LOC100737681)
			LOC100737852	peptidyl-prolyl cis-trans isomerase C-like
			LOC100738023	zinc finger protein 705A-like (LOC100738023; LOC100525433)
			LOC100738213	collagen alpha-1(I) chain
			LOC100738595	fibulin-2
			LOC100738704	TNFAIP3-interacting protein 3; TNFAIP3-interacting protein 3-like (LOC100738704; LOC106504099)
			LOC100739719	tetraspanin-6(LOC100739719)
			LOC100739844	semaphorin-3A-like(LOC100739844)
			LOC102157535	40S ribosomal protein S6 pseudogene
			LOC102161405	uncharacterized LOC102161405
			LOC102161446	ras GTPase-activating protein 2-like; ras GTPase-activating protein 2 (LOC102161446; LOC100515414)
			LOC102164530	collagen alpha-1(XV) chain
			LOC102164566	uncharacterized LOC102164566
			LOC102164569	pro-neuregulin-1, membrane-bound isoform-like (LOC100739280; LOC102164569)
			LOC102165708	uncharacterized LOC102165708
			LOC102165740	uncharacterized LOC102165740
			LOC102168155	succinate--hydroxymethylglutarate CoA-transferase; succinate--hydroxymethylglutarate CoA-transferase pseudogene (LOC102168155; LOC106508407)
			LOC106505778	protein HEG homolog 1-like; HEG homolog 1 (zebrafish) (LOC106505778; HEG1)
			LOC106508945	uncharacterized LOC106508945; transmembrane protein 30A; uncharacterized LOC100737076 (LOC106508945; TMEM30A; LOC100737076)
			LOC397380	uncharacterized LOC397380
			LOC397571	collagen alpha-1(I) chain
			LRRC17	leucine rich repeat containing 17; leucine-rich repeat-containing protein 17-like; leucine-rich repeat-containing protein 17 (LRRC17; LOC100513808; LOC100513611)
			LUM	lumican(LUM)
			MAGED1	MAGE family member D1(MAGED1)
			MAN1A1	mannosidase alpha class 1A member 1(MAN1A1)
			MAN2A1	mannosidase alpha class 2A member 1(MAN2A1)
			MARCKS	myristoylated alanine rich protein kinase C substrate(MARCKS)
			MDFIC	MyoD family inhibitor domain containing(MDFIC)
			MFAP2	microfibrillar associated protein 2(MFAP2)
			MFGE8	milk fat globule-EGF factor 8 protein(MFGE8)
			MGAT4D	MGAT4 family member D(MGAT4D)

			MIER1	matrix metallopeptidase 1(MMP1)
			MMP1	MORC family CW-type zinc finger 3(MORC3)
			MORC3	mannose receptor C type 2(MRC2)
			MRC2	mannose receptor, C type 2
			NCEH1	neutral cholesterol ester hydrolase 1(NCEH1)
			NOX4	NADPH oxidase 4(NOX4)
			NSF	N-ethylmaleimide sensitive factor, vesicle fusing ATPase(NSF)
			OLFM3	olfactomedin 3(OLFM3)
			OLFML3	olfactomedin like 3(OLFML3)
			OMD	osteomodulin(OMD)
			OSTC	oligosaccharyltransferase complex non-catalytic subunit(OSTC)
			P2RX7	purinergic receptor P2X 7(P2RX7)
			PDE7B	phosphodiesterase 7B(PDE7B)
			PDP1	pyruvate dehyrogenase phosphatase catalytic subunit 1(PDP1)
			PLAU	plasminogen activator, urokinase(PLAU)
			PLAUR	plasminogen activator, urokinase receptor(PLAUR)
			PLEKHG2	pleckstrin homology and RhoGEF domain containing G2(PLEKHG2)
			PLOD2	procollagen-lysine,2-oxoglutarate 5-dioxygenase 2(PLOD2)
			PMAIP1	phorbol-12-myristate-13-acetate-induced protein 1(PMAIP1)
			PPP3CC	protein phosphatase 3 catalytic subunit gamma(PPP3CC)
			PPP4R4	protein phosphatase 4 regulatory subunit 4(PPP4R4)
			PRDM1	PR/SET domain 1(PRDM1)
			PRSS23	protease, serine 23(PRSS23)
			PTPN1	protein tyrosine phosphatase, non-receptor type 1(PTPN1)
			RAB32	RAB32, member RAS oncogene family(RAB32)
			RAB8B	RAB8B, member RAS oncogene family(RAB8B)
			RAP2C	RAP2C, member of RAS oncogene family(RAP2C)
			RARA	retinoic acid receptor alpha(RARA)
			RNF24	ring finger protein 24
			ROBO2	roundabout guidance receptor 2(ROBO2)
			SDC2	syndecan 2(SDC2)
			SDC4	syndecan 4(SDC4)
			SEC22B	SEC22 homolog B, vesicle trafficking protein(SEC22B)
			SEC23A	Sec23 homolog A, coat complex II component(SEC23A)
			SELL	selectin L(SELL)
			SERPINH1	signal induced proliferation associated 1 like 1(SIPA1L1)
			SIPA1L1	solute carrier family 12 member 2(SLC12A2)
			SLC12A2	solute carrier family 38 member 2(SLC38A2)
			SLC38A2	solute carrier family 9 member B1(SLC9B1)
			SLC9B1	snail family transcriptional repressor 2(SNAI2)
			SNAI2	snail homolog 2 (Drosophila)(SNAI2)
			SPARC	secreted protein, acidic, cysteine-rich (osteonectin)(SPARC)
			SRP54	signal recognition particle 54(SRP54)
			SRPX2	sushi repeat containing protein, X-linked 2(SRPX2)
			STAT2	signal transducer and activator of transcription 2(STAT2)
			STEAP2	STEAP2 metalloreductase(STEAP2)
			TAC3	tachykinin 3(TAC3)
			TBC1D15	TBC1 domain family member 15(TBC1D15)
			TCEAL8	transcription elongation factor A like 8(TCEAL8)
			TCF7L2	transcription factor 7 like 2(TCF7L2)
			TDG	thymine-DNA glycosylase(TDG)
			TMEM263	transmembrane protein 263(TMEM263)
			TMEM39A	transmembrane protein 39A(TMEM39A)
			TMEM74	transmembrane protein 74(TMEM74)
			TXNDC5	thioredoxin domain containing 5(TXNDC5)
			UACA	uveal autoantigen with coiled-coil domains and ankyrin repeats(UACA)
			UCAL-P19	uterocalin P19(UCAL-P19)
			USP15	ubiquitin specific peptidase 15(USP15)

			VCAN	versican(VCAN)
			VNN1	vanin 1(VNN1)
			VWA5A	von Willebrand factor A domain containing 5A(VWA5A)
			WBP5	WW domain binding protein 5
			WDR72	WD repeat domain 72
			WNT2	Wnt family member 2(WNT2)
			XBP1	X-box binding protein 1(XBP1)
			ZBTB1	zinc finger and BTB domain containing 1(ZBTB1)
			ZNF516	zinc finger protein 516(ZNF516)

Supplementary Table 4. List (akronym and description) of differentially expressed genes ($P < 0.05$) solely related to copulation, in pig utero-tubal junction mucosa (UTJ) up- or down-regulated.

UP			*DOWN*	
ADAP2	ArfGAP with dual PH domains 2(ADAP2)		*ABHD2*	abhydrolase domain containing 2(ABHD2)
ALDH2	aldehyde dehydrogenase 2 family (mitochondrial)(ALDH2)		*ACKR3*	atypical chemokine receptor 3(ACKR3)
ANKZF1	ankyrin repeat and zinc finger domain containing 1(ANKZF1)		*ALDH18A1*	aldehyde dehydrogenase 18 family member A1(ALDH18A1)
ARHGAP20	Rho GTPase activating protein 20(ARHGAP20)		*ATP8B2*	ATPase phospholipid transporting 8B2(ATP8B2)
ARRB1	arrestin beta 1(ARRB1)		*B4GALT1*	beta-1,4-galactosyltransferase 1(B4GALT1)
BMPER	BMP binding endothelial regulator(BMPER)		*C13H3orf14*	chromosome 13 open reading frame, human C3orf14(C13H3orf14)
C18H7orf49	chromosome 18 open reading frame, human C7orf49(C18H7orf49)		*C6H1orf123*	chromosome 6 open reading frame, human C1orf123(C6H1orf123)
C1H9orf171	chromosome 1 open reading frame, human C9orf171		*CALM2*	calmodulin 2(CALM2)
C1QC	complement C1q C chain(C1QC)		*CCNO*	cyclin O(CCNO)
C4H1orf110	chromosome 4 open reading frame, human C1orf110		*CD248*	CD248 molecule(CD248)
CA9	carbonic anhydrase 9(CA9)		*CD276*	CD276 molecule(CD276)
CACNA1D	calcium voltage-gated channel subunit alpha1 D(CACNA1D)		*CD63*	CD63 molecule(CD63)
CAMK1D	calcium/calmodulin dependent protein kinase ID(CAMK1D)		*COL1A2*	collagen type I alpha 2 chain(COL1A2)
CCDC37	coiled-coil domain containing 37; coiled-coil domain-containing protein 37-like (CCDC37; LOC100624365)		*COPG1*	coatomer protein complex, subunit gamma 1
CCDC81	coiled-coil domain containing 81(CCDC81)		*CPQ*	carboxypeptidase Q; carboxypeptidase Q-like (CPQ; LOC106508327; LOC102163803)
CD27	CD27 molecule(CD27)		*CPXM1*	carboxypeptidase X, M14 family member 1(CPXM1)
CDC14A	cell division cycle 14A(CDC14A)		*CREB3L2*	cAMP responsive element binding protein 3 like 2(CREB3L2)
CHDH	choline dehydrogenase(CHDH)		*CYP51*	cytochrome P450, family 51, subfamily A, polypeptide 1(CYP51)
CHKA	choline kinase alpha(CHKA)		*DPF2*	double PHD fingers 2(DPF2)
CHN2	chimerin 2(CHN2)		*EEF1G*	eukaryotic translation elongation factor 1 gamma(EEF1G)
COLCA2	colorectal cancer associated 2(COLCA2)		*FAM114A1*	family with sequence similarity 114 member A1(FAM114A1)
CTDSPL	CTD small phosphatase like(CTDSPL)		*FAM167B*	family with sequence similarity 167 member B(FAM167B)
CXHXorf21	chromosome X open reading frame, human CXorf21(CXHXorf21)		*FAM19A4*	family with sequence similarity 19 member A4, C-C motif chemokine like(FAM19A4)
DLEC1	deleted in lung and esophageal cancer 1(DLEC1)		*FAM98A*	family with sequence similarity 98 member A(FAM98A)
DYRK1A	dual specificity tyrosine phosphorylation regulated kinase 1A(DYRK1A)		*FBN1*	fibrillin 1(FBN1)
ECT2L	epithelial cell transforming 2 like(ECT2L)		*FBXL5*	F-box and leucine rich repeat protein 5(FBXL5)
EFCAB6	EF-hand calcium binding domain 6(EFCAB6)		*FKBP10*	FK506 binding protein 10, 65 kDa
ELMO1	engulfment and cell motility 1(ELMO1)		*GAPDH*	glyceraldehyde-3-phosphate dehydrogenase(GAPDH)
EP300	E1A binding protein p300(EP300)		*GDI2*	GDP dissociation inhibitor 2(GDI2)
EPB41L1	erythrocyte membrane protein band 4.1 like 1(EPB41L1)		*GNG2*	G protein subunit gamma 2(GNG2)
EPS15	epidermal growth factor receptor pathway substrate 15(EPS15)		*HMGCS1*	3-hydroxy-3-methylglutaryl-CoA synthase 1(HMGCS1)
FHAD1	forkhead-associated (FHA) phosphopeptide binding domain 1		*HSPA13*	heat shock protein family A (Hsp70) member 13(HSPA13)
GAS2L2	growth arrest specific 2 like 2(GAS2L2)		*HTRA4*	HtrA serine peptidase 4(HTRA4)
HAVCR2	hepatitis A virus cellular receptor 2(HAVCR2)		*KDELR3*	KDEL endoplasmic reticulum protein retention receptor 3(KDELR3)
HIPK3	homeodomain interacting protein kinase 3(HIPK3)		*LOC100513729*	hippocampus abundant transcript 1 protein
HPGD	hydroxyprostaglandin dehydrogenase 15-(NAD)(HPGD)		*LOC100523107*	melanoma-associated antigen D4(LOC100523107)
IRF2	interferon regulatory factor 2(IRF2)		*LOC100524585*	ephrin type-B receptor 1(LOC100524585)
JADE1	jade family PHD finger 1(JADE1)		*LOC100623063*	protein FAM69A-like; family with sequence similarity 69, member A (LOC100623063; FAM69A)
JAK2	Janus kinase 2(JAK2)		*LOC100737260*	astrotactin-1-like
KCNRG	potassium channel regulator(KCNRG)		*LOC100738034*	citrate synthase, mitochondrial-like; citrate synthase (LOC100738034; CS)
KIAA1147	KIAA1147 ortholog(KIAA1147)		*LOC100738213*	collagen alpha-1(I) chain
KIAA1551	KIAA1551 ortholog(KIAA1551)		*LOC100739719*	tetraspanin-6(LOC100739719)

KIAA2018	KIAA2018 ortholog		*LOC102161647*	trichohyalin
LOC100154396	sister chromatid cohesion protein PDS5 homolog B(LOC100154396)		*LOC397571*	collagen alpha-1(I) chain
LOC100157427	nesprin-1-like		*MAGED1*	MAGE family member D1(MAGED1)
LOC100514181	coiled-coil domain-containing protein 40		*MIR218B*	microRNA mir-218b
LOC100514701	MAP kinase-activating death domain protein		*MMP19*	matrix metallopeptidase 19(MMP19)
LOC100518397	dynein heavy chain 9, axonemal		*NPR2*	natriuretic peptide receptor B/guanylate cyclase B (atrionatriuretic peptide receptor B)(NPR2)
LOC100519735	dynein heavy chain 9, axonemal-like		*OSTC*	oligosaccharyltransferase complex non-catalytic subunit(OSTC)
LOC100525061	B-cell scaffold protein with ankyrin repeats(LOC100525061)		*PGK1*	phosphoglycerate kinase 1(PGK1)
LOC100624977	uncharacterized protein C9orf117-like; chromosome 1 open reading frame, human C9orf117 (LOC100624977; C1H9orf117)		*PLAUR*	plasminogen activator, urokinase receptor(PLAUR)
LOC100627422	ligand-dependent corepressor(LOC100627422)		*PPM1J*	protein phosphatase, Mg2+/Mn2+ dependent, 1J
LOC102164742	uncharacterized LOC102164742		*PRSS23*	protease, serine 23(PRSS23)
LOC106504646	intraflagellar transport protein 88 homolog; uncharacterized LOC106504652 (LOC106504646; LOC106504652)		*PTGFRN*	prostaglandin F2 receptor inhibitor(PTGFRN)
MAFB	MAF bZIP transcription factor B(MAFB)		*RABAC1*	Rab acceptor 1(RABAC1)
MAPK10	mitogen-activated protein kinase 10(MAPK10)		*REXO2*	RNA exonuclease 2(REXO2)
MGA	MAX gene associated		*RHOC*	ras homolog family member C(RHOC)
MIR425	microRNA mir-425		*SEC23A*	Sec23 homolog A, coat complex II component(SEC23A)
MTR	5-methyltetrahydrofolate-homocysteine methyltransferase(MTR)		*SEC61G*	Sec61 translocon gamma subunit(SEC61G)
MYLIP	myosin regulatory light chain interacting protein(MYLIP)		*SEPN1*	selenoprotein N, 1
PNRC1	proline-rich nuclear receptor coactivator 1		*SHAS2*	hyaluronan synthase 2(SHAS2)
RAB11FIP1	RAB11 family interacting protein 1(RAB11FIP1)		*SLC9B1*	solute carrier family 9 member B1(SLC9B1)
RDH10	retinol dehydrogenase 10 (all-trans)(RDH10)		*SQLE*	squalene epoxidase(SQLE)
RENBP	renin binding protein(RENBP)		*SRPX2*	sushi repeat containing protein, X-linked 2(SRPX2)
RNF128	ring finger protein 128, E3 ubiquitin protein ligase(RNF128)		*TMEM183A*	transmembrane protein 183A(TMEM183A)
SLC2A12	solute carrier family 2 member 12(SLC2A12)		*TMEM45A*	transmembrane protein 45A(TMEM45A)
STMND1	stathmin domain containing 1(STMND1)		*TP23*	basic proline-rich protein(TP23)
TCTE1	t-complex-associated-testis-expressed 1(TCTE1)		*VCAN*	versican(VCAN)
TEKT2	tektin 2(TEKT2)		*WBP5*	WW domain binding protein 5
TSHZ2	teashirt zinc finger homeobox 2(TSHZ2)			
TSNAXIP1	translin associated factor X interacting protein 1(TSNAXIP1)			
TTC39C	tetratricopeptide repeat domain 39C(TTC39C)			
TTLL10	tubulin tyrosine ligase-like family member 10			
TTLL6	tubulin tyrosine ligase like 6(TTLL6)			
UAP1	UDP-N-acetylglucosamine pyrophosphorylase 1(UAP1)			
ZNF12	zinc finger protein 12			
ZNF202	zinc finger protein 202			
ZNF217	zinc finger protein 217(ZNF217)			
ZXDB	zinc finger, X-linked, duplicated B(ZXDB)			

Table 5. List of biological **KEGG-pathways** (**DAVID database,** P< 0.05) of differentially-expressed genes (DEGs, ID and name) solely related to copulation, in pig uterine mucosae (endocervix (Cvx), endometrium (distal: D-Endom or proximal: P-Endom) and utero-tubal junction (UTJ)), up- or down-regulated.

**Tissue**	**Regulation**	**ID**	**Gene Name**	**KEGG_PATHWAY**
**Cvx**	**UP**	LOC100153094	glutathione S-transferase theta-1(LOC100153094)	ssc00480:Glutathione metabolism,ssc00980:Metabolism of xenobiotics by cytochrome P450,ssc00982:Drug metabolism - cytochrome P450,ssc05204:Chemical carcinogenesis,
**Cvx**	**UP**	RETSAT	retinol saturase(RETSAT)	ssc00830:Retinol metabolism,
**Cvx**	**UP**	FMO1	flavin containing monooxygenase 1(FMO1)	ssc00982:Drug metabolism - cytochrome P450,
**Cvx**	**UP**	LOC100521052	mitochondrial inner membrane protease subunit 2(LOC100521052)	ssc03060:Protein export,\
**Cvx**	**UP**	IGF1R	insulin like growth factor 1 receptor(IGF1R)	ssc04014:Ras signaling pathway,ssc04015:Rap1 signaling pathway,ssc04066:HIF-1 signaling pathway,ssc04068:FoxO signaling pathway,ssc04114:Oocyte meiosis,ssc04144:Endocytosis,ssc04151:PI3K-Akt signaling pathway,ssc04152:AMPK signaling pathway,ssc04510:Focal adhesion,ssc04520:Adherens junction,ssc04550:Signaling pathways regulating pluripotency of stem cells,ssc04730:Long-term

				depression,ssc04913:Ovarian steroidogenesis,ssc04914:Progesterone-mediated oocyte maturation,ssc05200:Pathways in cancer,ssc05202:Transcriptional misregulation in cancer,ssc05205:Proteoglycans in cancer,ssc05214:Glioma,ssc05215:Prostate cancer,ssc05218:Melanoma,
**Cvx**	**UP**	PRKCQ	protein kinase C theta(PRKCQ)	ssc04064:NF-kappa B signaling pathway,ssc04270:Vascular smooth muscle contraction,ssc04660:T cell receptor signaling pathway,ssc04750:Inflammatory mediator regulation of TRP channels,ssc04920:Adipocytokine signaling pathway,ssc04931:Insulin resistance,ssc05162:Measles,
**Cvx**	**UP**	SESN3	sestrin 3(SESN3)	ssc04115:p53 signaling pathway,
**Cvx**	**UP**	VAMP2	vesicle associated membrane protein 2(VAMP2)	ssc04130:SNARE interactions in vesicular transport,ssc04721:Synaptic vesicle cycle,ssc04911:Insulin secretion,ssc04962:Vasopressin-regulated water reabsorption,ssc04970:Salivary secretion,
**Cvx**	**UP**	GGA2	golgi associated, gamma adaptin ear containing, ARF binding protein 2(GGA2)	ssc04142:Lysosome,
**Cvx**	**UP**	THBS3	thrombospondin 3(THBS3)	ssc04145:Phagosome,ssc04151:PI3K-Akt signaling pathway,ssc04510:Focal adhesion,ssc04512:ECM-receptor interaction,ssc05144:Malaria,
**Cvx**	**UP**	DECR2	2,4-dienoyl-CoA reductase 2, peroxisomal(DECR2)	ssc04146:Peroxisome,
**Cvx**	**UP**	LOC100738970	CXXC-type zinc finger protein 4(LOC100738970)	ssc04310:Wnt signaling pathway,\
**Cvx**	**UP**	NCOR2	nuclear receptor corepressor 2(NCOR2)	ssc04330:Notch signaling pathway,ssc05169:Epstein-Barr virus infection,
**Cvx**	**UP**	NTN4	netrin 4(NTN4)	ssc04360:Axon guidance,
**Cvx**	**UP**	RFXAP	regulatory factor X associated protein(RFXAP)	ssc04612:Antigen processing and presentation,ssc05152:Tuberculosis,ssc05340:Primary immunodeficiency,
**Cvx**	**UP**	AGT	angiotensinogen(AGT)	ssc04614:Renin-angiotensin system,ssc04924:Renin secretion,\
**Cvx**	**UP**	KCNQ4	potassium voltage-gated channel subfamily Q member 4(KCNQ4)	ssc04725:Cholinergic synapse,
**Cvx**	**UP**	SLC9A3R2	SLC9A3 regulator 2(SLC9A3R2)	ssc04960:Aldosterone-regulated sodium reabsorption,
**Cvx**	**UP**	HDAC9	histone deacetylase 9(HDAC9)	ssc05034:Alcoholism,ssc05203:Viral carcinogenesis,
**Cvx**	**UP**	CRTC1	CREB regulated transcription coactivator 1(CRTC1)	ssc05166:HTLV-I infection,\
**Cvx**	**UP**	MIR181B-1	microRNA 181b-1(MIR181B-1)	ssc05206:MicroRNAs in cancer,\
**Cvx**	**DOWN**	PGD	phosphogluconate dehydrogenase(PGD)	ssc00030:Pentose phosphate pathway,ssc00480:Glutathione metabolism,ssc01100:Metabolic pathways,ssc01130:Biosynthesis of antibiotics,ssc01200:Carbon metabolism,
**Cvx**	**DOWN**	LOC100511841	UDP-glucuronosyltransferase 1-10(LOC100511841)	ssc00040:Pentose and glucuronate interconversions,ssc00053:Ascorbate and aldarate metabolism,ssc00140:Steroid hormone biosynthesis,ssc00830:Retinol metabolism,ssc00860:Porphyrin and chlorophyll metabolism,ssc00980:Metabolism of xenobiotics by cytochrome P450,ssc00982:Drug metabolism - cytochrome P450,ssc00983:Drug metabolism - other enzymes,ssc01100:Metabolic pathways,ssc05204:Chemical carcinogenesis,
**Cvx**	**DOWN**	B4GALT1	beta-1,4-galactosyltransferase 1(B4GALT1)	ssc00052:Galactose metabolism,ssc00510:N-Glycan biosynthesis,ssc00514:Other types of O-glycan biosynthesis,ssc00533:Glycosaminoglycan biosynthesis - keratan sulfate,ssc00601:Glycosphingolipid biosynthesis - lacto and neolacto series,ssc01100:Metabolic pathways,
**Cvx**	**DOWN**	HMGCS1	3-hydroxy-3-methylglutaryl-CoA synthase 1(HMGCS1)	ssc00072:Synthesis and degradation of ketone bodies,ssc00280:Valine, leucine and isoleucine degradation,ssc00650:Butanoate metabolism,ssc00900:Terpenoid backbone biosynthesis,ssc01100:Metabolic pathways,ssc01130:Biosynthesis of antibiotics,
**Cvx**	**DOWN**	CYP51	cytochrome P450, family 51, subfamily A, polypeptide 1(CYP51)	ssc00100:Steroid biosynthesis,ssc01100:Metabolic pathways,ssc01130:Biosynthesis of antibiotics,
**Cvx**	**DOWN**	SQLE	squalene epoxidase(SQLE)	ssc00100:Steroid biosynthesis,ssc01100:Metabolic pathways,ssc01130:Biosynthesis of antibiotics,
**Cvx**	**DOWN**	ATP6V0C	ATPase H+ transporting V0 subunit c(ATP6V0C)	ssc00190:Oxidative phosphorylation,ssc01100:Metabolic pathways,ssc04142:Lysosome,ssc04145:Phagosome,ssc04721:Synaptic vesicle cycle,ssc04966:Collecting duct acid secretion,ssc05152:Tuberculosis,ssc05323:Rheumatoid arthritis,
**Cvx**	**DOWN**	NT5C2	5'-nucleotidase, cytosolic II(NT5C2)	ssc00230:Purine metabolism,ssc00240:Pyrimidine metabolism,ssc00760:Nicotinate and nicotinamide metabolism,ssc01100:Metabolic pathways,
**Cvx**	**DOWN**	PAPSS2	3'-phosphoadenosine 5'-phosphosulfate synthase 2(PAPSS2)	ssc00230:Purine metabolism,ssc00450:Selenocompound metabolism,ssc00920:Sulfur metabolism,ssc01100:Metabolic pathways,ssc01130:Biosynthesis of antibiotics,
**Cvx**	**DOWN**	NPR2	natriuretic peptide receptor B/guanylate cyclase B (atrionatriuretic peptide receptor B)(NPR2)	ssc00230:Purine metabolism,ssc04022:cGMP-PKG signaling pathway,ssc04270:Vascular smooth muscle contraction,ssc04921:Oxytocin signaling pathway,
**Cvx**	**DOWN**	CMPK2	cytidine/uridine monophosphate kinase 2(CMPK2)	ssc00240:Pyrimidine metabolism,ssc01100:Metabolic pathways,
**Cvx**	**DOWN**	PLOD2	procollagen-lysine,2-oxoglutarate 5-dioxygenase 2(PLOD2)	ssc00310:Lysine degradation,
**Cvx**	**DOWN**	SEPHS2	selenophosphate synthetase 2(SEPHS2)	ssc00450:Selenocompound metabolism,ssc01100:Metabolic pathways,
**Cvx**	**DOWN**	MAN2A1	mannosidase alpha class 2A member 1(MAN2A1)	ssc00510:N-Glycan biosynthesis,ssc01100:Metabolic pathways,
**Cvx**	**DOWN**	GCNT3	glucosaminyl (N-acetyl) transferase 3, mucin type(GCNT3)	ssc00512:Mucin type O-Glycan biosynthesis,ssc01100:Metabolic pathways,
**Cvx**	**DOWN**	AGPAT2	1-acylglycerol-3-phosphate O-acyltransferase 2(AGPAT2)	ssc00561:Glycerolipid metabolism,ssc00564:Glycerophospholipid metabolism,ssc01100:Metabolic pathways,ssc04975:Fat digestion and absorption,
**Cvx**	**DOWN**	SGPP2	sphingosine-1-phosphate phosphatase 2(SGPP2)	ssc00600:Sphingolipid metabolism,ssc04071:Sphingolipid signaling pathway,
**Cvx**	**DOWN**	B3GALT2	beta-1,3-galactosyltransferase 2(B3GALT2)	ssc00601:Glycosphingolipid biosynthesis - lacto and neolacto series,ssc01100:Metabolic pathways,
**Cvx**	**DOWN**	PANK3	pantothenate kinase 3(PANK3)	ssc00770:Pantothenate and CoA biosynthesis,ssc01100:Metabolic pathways,
**Cvx**	**DOWN**	HMGCR	3-hydroxy-3-methylglutaryl-CoA reductase(HMGCR)	ssc00900:Terpenoid backbone biosynthesis,ssc01100:Metabolic pathways,ssc01130:Biosynthesis of antibiotics,ssc04152:AMPK signaling pathway,ssc04976:Bile secretion,
**Cvx**	**DOWN**	FDPS	farnesyl diphosphate synthase(FDPS)	ssc00900:Terpenoid backbone biosynthesis,ssc01100:Metabolic pathways,ssc01130:Biosynthesis of antibiotics,ssc05164:Influenza A,ssc05166:HTLV-I infection,
**Cvx**	**DOWN**	GARS	glycyl-tRNA synthetase(GARS)	ssc00970:Aminoacyl-tRNA biosynthesis,
**Cvx**	**DOWN**	KSR1	kinase suppressor of ras 1(KSR1)	ssc04014:Ras signaling pathway,ssc05152:Tuberculosis,
**Cvx**	**DOWN**	THBS1	thrombospondin 1(THBS1)	ssc04015:Rap1 signaling pathway,ssc04115:p53 signaling pathway,ssc04145:Phagosome,ssc04151:PI3K-Akt signaling pathway,ssc04350:TGF-beta signaling pathway,ssc04510:Focal adhesion,ssc04512:ECM-receptor interaction,ssc05144:Malaria,ssc05205:Proteoglycans in cancer,ssc05206:MicroRNAs in cancer,ssc05219:Bladder cancer,
**Cvx**	**DOWN**	CXCL10	C-X-C motif chemokine ligand 10(CXCL10)	ssc04060:Cytokine-cytokine receptor interaction,ssc04062:Chemokine signaling pathway,ssc04620:Toll-like receptor signaling pathway,ssc04622:RIG-I-like receptor signaling pathway,ssc04623:Cytosolic DNA-sensing pathway,ssc04668:TNF signaling pathway,ssc05164:Influenza A,
**Cvx**	**DOWN**	IL6ST	interleukin 6 signal transducer(IL6ST)	ssc04060:Cytokine-cytokine receptor interaction,ssc04550:Signaling pathways regulating pluripotency of stem cells,ssc04630:Jak-STAT signaling pathway,ssc05203:Viral carcinogenesis,
**Cvx**	**DOWN**	S1PR3	sphingosine-1-phosphate receptor 3(S1PR3)	ssc04071:Sphingolipid signaling pathway,ssc04080:Neuroactive ligand-receptor interaction,
**Cvx**	**DOWN**	NPY2R	neuropeptide Y receptor Y2(NPY2R)	ssc04080:Neuroactive ligand-receptor interaction,
**Cvx**	**DOWN**	REC8	REC8 meiotic recombination protein(REC8)	ssc04114:Oocyte meiosis,\

**Cvx**	**DOWN**	UBA7	ubiquitin like modifier activating enzyme 7(UBA7)	ssc04120:Ubiquitin mediated proteolysis,ssc05012:Parkinson's disease,
**Cvx**	**DOWN**	NPC1	NPC intracellular cholesterol transporter 1(NPC1)	ssc04142:Lysosome,
**Cvx**	**DOWN**	SLA-7	MHC class I antigen 7(SLA-7)	ssc04144:Endocytosis,ssc04145:Phagosome,ssc04514:Cell adhesion molecules (CAMs),ssc04612:Antigen processing and presentation,ssc04940:Type I diabetes mellitus,ssc05166:HTLV-I infection,ssc05168:Herpes simplex infection,ssc05169:Epstein-Barr virus infection,ssc05203:Viral carcinogenesis,ssc05320:Autoimmune thyroid disease,ssc05330:Allograft rejection,ssc05332:Graft-versus-host disease,ssc05416:Viral myocarditis,
**Cvx**	**DOWN**	SLC25A17	solute carrier family 25 member 17(SLC25A17)	ssc04146:Peroxisome,
**Cvx**	**DOWN**	LOC100517053	integrin alpha-3(LOC100517053)	ssc04151:PI3K-Akt signaling pathway,ssc04510:Focal adhesion,ssc04512:ECM-receptor interaction,ssc04640:Hematopoietic cell lineage,ssc04810:Regulation of actin cytoskeleton,ssc05200:Pathways in cancer,ssc05222:Small cell lung cancer,ssc05410:Hypertrophic cardiomyopathy (HCM),ssc05412:Arrhythmogenic right ventricular cardiomyopathy (ARVC),ssc05414:Dilated cardiomyopathy, \
**Cvx**	**DOWN**	CD47	CD47 molecule(CD47)	ssc04512:ECM-receptor interaction,
**Cvx**	**DOWN**	LOC100525346	poliovirus receptor homolog(LOC100525346)	ssc04514:Cell adhesion molecules (CAMs),
**Cvx**	**DOWN**	VCAN	versican(VCAN)	ssc04514:Cell adhesion molecules (CAMs),
**Cvx**	**DOWN**	GJA1	gap junction protein alpha 1(GJA1)	ssc04540:Gap junction,ssc05412:Arrhythmogenic right ventricular cardiomyopathy (ARVC),
**Cvx**	**DOWN**	PLAT	plasminogen activator, tissue type(PLAT)	ssc04610:Complement and coagulation cascades,ssc05202:Transcriptional misregulation in cancer,
**Cvx**	**DOWN**	PLAUR	plasminogen activator, urokinase receptor(PLAUR)	ssc04610:Complement and coagulation cascades,ssc05205:Proteoglycans in cancer,
**Cvx**	**DOWN**	TAPBP	TAP binding protein(TAPBP)	ssc04612:Antigen processing and presentation,
**Cvx**	**DOWN**	ENPEP	glutamyl aminopeptidase(ENPEP)	ssc04614:Renin-angiotensin system,
**Cvx**	**DOWN**	ZBP1	Z-DNA binding protein 1(ZBP1)	ssc04623:Cytosolic DNA-sensing pathway,
**Cvx**	**DOWN**	RIPK3	receptor interacting serine/threonine kinase 3(RIPK3)	ssc04623:Cytosolic DNA-sensing pathway,ssc04668:TNF signaling pathway,
**Cvx**	**DOWN**	MMP3	matrix metallopeptidase 3 (stromelysin 1, progelatinase)(MMP3)	ssc04668:TNF signaling pathway,ssc05202:Transcriptional misregulation in cancer,ssc05323:Rheumatoid arthritis,
**Cvx**	**DOWN**	RASD1	ras related dexamethasone induced 1(RASD1)	ssc04713:Circadian entrainment,
**Cvx**	**DOWN**	LOC100154959	olfactory receptor 4K3(LOC100154959)	ssc04740:Olfactory transduction,
**Cvx**	**DOWN**	MX1	myxovirus (influenza virus) resistance 1, interferon-inducible protein p78 (mouse)(MX1)	ssc05162:Measles,ssc05164:Influenza A,
**Cvx**	**DOWN**	RSAD2	radical S-adenosyl methionine domain containing 2(RSAD2)	ssc05164:Influenza A,
**Cvx**	**DOWN**	NCOA4	nuclear receptor coactivator 4(NCOA4)	ssc05200:Pathways in cancer,ssc05216:Thyroid cancer,
**DistUt**	**UP**	ALDH2	aldehyde dehydrogenase 2 family (mitochondrial)(ALDH2)	ssc00010:Glycolysis / Gluconeogenesis,ssc00053:Ascorbate and aldarate metabolism,ssc00071:Fatty acid degradation,ssc00280:Valine, leucine and isoleucine degradation,ssc00310:Lysine degradation,ssc00330:Arginine and proline metabolism,ssc00340:Histidine metabolism,ssc00380:Tryptophan metabolism,ssc00410:beta-Alanine metabolism,ssc00561:Glycerolipid metabolism,ssc00620:Pyruvate metabolism,ssc01100:Metabolic pathways,ssc01130:Biosynthesis of antibiotics,
**DistUt**	**UP**	IDH3G	isocitrate dehydrogenase 3 (NAD(+)) gamma(IDH3G)	ssc00020:Citrate cycle (TCA cycle),ssc01100:Metabolic pathways,ssc01130:Biosynthesis of antibiotics,ssc01200:Carbon metabolism,ssc01210:2-Oxocarboxylic acid metabolism,ssc01230:Biosynthesis of amino acids,
**DistUt**	**UP**	HADH	hydroxyacyl-CoA dehydrogenase(HADH)	ssc00062:Fatty acid elongation,ssc00071:Fatty acid degradation,ssc00280:Valine, leucine and isoleucine degradation,ssc00310:Lysine degradation,ssc00380:Tryptophan metabolism,ssc00650:Butanoate metabolism,ssc01100:Metabolic pathways,ssc01130:Biosynthesis of antibiotics,ssc01212:Fatty acid metabolism,
**DistUt**	**UP**	NDUFV1	NADH:ubiquinone oxidoreductase core subunit V1(NDUFV1)	ssc00190:Oxidative phosphorylation,ssc01100:Metabolic pathways,ssc04932:Non-alcoholic fatty liver disease (NAFLD),ssc05010:Alzheimer's disease,ssc05012:Parkinson's disease,ssc05016:Huntington's disease,
**DistUt**	**UP**	NDUFA10	NADH:ubiquinone oxidoreductase subunit A10(NDUFA10)	ssc00190:Oxidative phosphorylation,ssc01100:Metabolic pathways,ssc04932:Non-alcoholic fatty liver disease (NAFLD),ssc05010:Alzheimer's disease,ssc05012:Parkinson's disease,ssc05016:Huntington's disease,
**DistUt**	**UP**	ATP5E	ATP synthase, H+ transporting, mitochondrial F1 complex, epsilon subunit(ATP5E)	ssc00190:Oxidative phosphorylation,ssc01100:Metabolic pathways,ssc05010:Alzheimer's disease,ssc05012:Parkinson's disease,ssc05016:Huntington's disease,
**DistUt**	**UP**	GMPR	guanosine monophosphate reductase(GMPR)	ssc00230:Purine metabolism,
**DistUt**	**UP**	POLR1A	RNA polymerase I subunit A(POLR1A)	ssc00230:Purine metabolism,ssc00240:Pyrimidine metabolism,ssc01100:Metabolic pathways,ssc03020:RNA polymerase,
**DistUt**	**UP**	POLR1D	RNA polymerase I subunit D(POLR1D)	ssc00230:Purine metabolism,ssc00240:Pyrimidine metabolism,ssc01100:Metabolic pathways,ssc03020:RNA polymerase,ssc04623:Cytosolic DNA-sensing pathway,
**DistUt**	**UP**	DGUOK	deoxyguanosine kinase(DGUOK)	ssc00230:Purine metabolism,ssc01100:Metabolic pathways,
**DistUt**	**UP**	GNMT	glycine N-methyltransferase(GNMT)	ssc00260:Glycine, serine and threonine metabolism,
**DistUt**	**UP**	ALDH6A1	aldehyde dehydrogenase 6 family member A1(ALDH6A1)	ssc00280:Valine, leucine and isoleucine degradation,ssc00410:beta-Alanine metabolism,ssc00562:Inositol phosphate metabolism,ssc00640:Propanoate metabolism,ssc01100:Metabolic pathways,ssc01200:Carbon metabolism,
**DistUt**	**UP**	EHMT2	euchromatic histone lysine methyltransferase 2(EHMT2)	ssc00310:Lysine degradation,
**DistUt**	**UP**	FAH	fumarylacetoacetate hydrolase(FAH)	ssc00350:Tyrosine metabolism,ssc01100:Metabolic pathways,
**DistUt**	**UP**	LOC100624445	gamma-glutamylcyclotransferase-like(LOC100624445)	ssc00480:Glutathione metabolism,
**DistUt**	**UP**	GSTK1	glutathione S-transferase kappa 1(GSTK1)	ssc00480:Glutathione metabolism,ssc00980:Metabolism of xenobiotics by cytochrome P450,ssc00982:Drug metabolism - cytochrome P450,ssc04146:Peroxisome,ssc05204:Chemical carcinogenesis,
**DistUt**	**UP**	POFUT1	protein O-fucosyltransferase 1(POFUT1)	ssc00514:Other types of O-glycan biosynthesis,
**DistUt**	**UP**	DGAT1	diacylglycerol O-acyltransferase 1(DGAT1)	ssc00561:Glycerolipid metabolism,ssc00830:Retinol metabolism,ssc01100:Metabolic pathways,ssc04975:Fat digestion and absorption,
**DistUt**	**UP**	MTMR7	myotubularin related protein 7(MTMR7)	ssc00562:Inositol phosphate metabolism,ssc01100:Metabolic pathways,ssc04070:Phosphatidylinositol signaling system,
**DistUt**	**UP**	PLD2	phospholipase D2(PLD2)	ssc00564:Glycerophospholipid metabolism,ssc00565:Ether lipid metabolism,ssc01100:Metabolic pathways,ssc04014:Ras signaling pathway,ssc04024:cAMP signaling pathway,ssc04071:Sphingolipid signaling pathway,ssc04144:Endocytosis,ssc04666:Fc gamma R-mediated phagocytosis,ssc04724:Glutamatergic synapse,ssc04912:GnRH signaling pathway,ssc05231:Choline metabolism in cancer,
**DistUt**	**UP**	CHKA	choline kinase alpha(CHKA)	ssc00564:Glycerophospholipid metabolism,ssc01100:Metabolic pathways,ssc05231:Choline metabolism in cancer,
**DistUt**	**UP**	LOC100513474	platelet-activating factor acetylhydrolase IB subunit gamma(LOC100513474)	ssc00565:Ether lipid metabolism,ssc01100:Metabolic pathways,

**DistUt**	**UP**	SPR	sepiapterin reductase (7,8-dihydrobiopterin:NADP+ oxidoreductase)(SPR)	ssc00790:Folate biosynthesis,ssc01100:Metabolic pathways,
**DistUt**	**UP**	DHRS3	dehydrogenase/reductase 3(DHRS3)	ssc00830:Retinol metabolism,ssc01100:Metabolic pathways,
**DistUt**	**UP**	FMO2	flavin containing monooxygenase 2(FMO2)	ssc00982:Drug metabolism - cytochrome P450,
**DistUt**	**UP**	ABCA9	ATP binding cassette subfamily A member 9(ABCA9)	ssc02010:ABC transporters,
**DistUt**	**UP**	CYFIP2	cytoplasmic FMR1 interacting protein 2(CYFIP2)	ssc03013:RNA transport,ssc04810:Regulation of actin cytoskeleton,
**DistUt**	**UP**	CNOT1	CCR4-NOT transcription complex subunit 1(CNOT1)	ssc03018:RNA degradation,
**DistUt**	**UP**	CNOT2	CCR4-NOT transcription complex subunit 2(CNOT2)	ssc03018:RNA degradation,
**DistUt**	**UP**	LOC100521052	mitochondrial inner membrane protease subunit 2(LOC100521052)	ssc03060:Protein export, \
**DistUt**	**UP**	CUL4A	cullin 4A(CUL4A)	ssc03420:Nucleotide excision repair,ssc04120:Ubiquitin mediated proteolysis,
**DistUt**	**UP**	MAP4K2	mitogen-activated protein kinase kinase kinase kinase 2(MAP4K2)	ssc04010:MAPK signaling pathway,
**DistUt**	**UP**	ARRB2	arrestin beta 2(ARRB2)	ssc04010:MAPK signaling pathway,ssc04062:Chemokine signaling pathway,ssc04144:Endocytosis,ssc05032:Morphine addiction,
**DistUt**	**UP**	CDC25B	cell division cycle 25B(CDC25B)	ssc04010:MAPK signaling pathway,ssc04110:Cell cycle,ssc04914:Progesterone-mediated oocyte maturation,ssc05206:MicroRNAs in cancer,
**DistUt**	**UP**	CACNB2	calcium voltage-gated channel auxiliary subunit beta 2(CACNB2)	ssc04010:MAPK signaling pathway,ssc04260:Cardiac muscle contraction,ssc04261:Adrenergic signaling in cardiomyocytes,ssc04921:Oxytocin signaling pathway,ssc05410:Hypertrophic cardiomyopathy (HCM),ssc05412:Arrhythmogenic right ventricular cardiomyopathy (ARVC),ssc05414:Dilated cardiomyopathy,
**DistUt**	**UP**	CSF1R	colony stimulating factor 1 receptor(CSF1R)	ssc04014:Ras signaling pathway,ssc04015:Rap1 signaling pathway,ssc04060:Cytokine-cytokine receptor interaction,ssc04151:PI3K-Akt signaling pathway,ssc04380:Osteoclast differentiation,ssc04640:Hematopoietic cell lineage,ssc05200:Pathways in cancer,ssc05202:Transcriptional misregulation in cancer,
**DistUt**	**UP**	PDGFD	platelet derived growth factor D(PDGFD)	ssc04014:Ras signaling pathway,ssc04015:Rap1 signaling pathway,ssc04151:PI3K-Akt signaling pathway,ssc04510:Focal adhesion,ssc04540:Gap junction,ssc04810:Regulation of actin cytoskeleton,ssc05215:Prostate cancer,ssc05218:Melanoma,ssc05231:Choline metabolism in cancer, \
**DistUt**	**UP**	VEGFB	vascular endothelial growth factor B(VEGFB)	ssc04014:Ras signaling pathway,ssc04015:Rap1 signaling pathway,ssc04151:PI3K-Akt signaling pathway,ssc04510:Focal adhesion,ssc05200:Pathways in cancer,
**DistUt**	**UP**	ADRA2A	adrenoceptor alpha 2A(ADRA2A)	ssc04022:cGMP-PKG signaling pathway,ssc04080:Neuroactive ligand-receptor interaction,
**DistUt**	**UP**	GABRB2	gamma-aminobutyric acid type A receptor beta2 subunit(GABRB2)	ssc04080:Neuroactive ligand-receptor interaction,ssc04723:Retrograde endocannabinoid signaling,ssc04726:Serotonergic synapse,ssc04727:GABAergic synapse,ssc05032:Morphine addiction,ssc05033:Nicotine addiction,
**DistUt**	**UP**	CDC25C	cell division cycle 25C(CDC25C)	ssc04110:Cell cycle,ssc04114:Oocyte meiosis,ssc04914:Progesterone-mediated oocyte maturation,ssc05206:MicroRNAs in cancer,
**DistUt**	**UP**	STX10	syntaxin 10(STX10)	ssc04130:SNARE interactions in vesicular transport,
**DistUt**	**UP**	UBQLN4	ubiquilin 4(UBQLN4)	ssc04141:Protein processing in endoplasmic reticulum,
**DistUt**	**UP**	HSP90AB1	heat shock protein 90 alpha family class B member 1(HSP90AB1)	ssc04141:Protein processing in endoplasmic reticulum,ssc04151:PI3K-Akt signaling pathway,ssc04612:Antigen processing and presentation,ssc04621:NOD-like receptor signaling pathway,ssc04914:Progesterone-mediated oocyte maturation,ssc04915:Estrogen signaling pathway,ssc05200:Pathways in cancer,ssc05215:Prostate cancer,
**DistUt**	**UP**	RUFY1	RUN and FYVE domain containing 1(RUFY1)	ssc04144:Endocytosis,
**DistUt**	**UP**	FCGR1A	Fc fragment of IgG, high affinity Ia, receptor (CD64)(FCGR1A)	ssc04145:Phagosome,ssc04380:Osteoclast differentiation,ssc04640:Hematopoietic cell lineage,ssc04666:Fc gamma R-mediated phagocytosis,ssc05140:Leishmaniasis,ssc05150:Staphylococcus aureus infection,ssc05152:Tuberculosis,ssc05202:Transcriptional misregulation in cancer,ssc05322:Systemic lupus erythematosus,
**DistUt**	**UP**	TBL1X	transducin beta like 1X-linked(TBL1X)	ssc04310:Wnt signaling pathway,
**DistUt**	**UP**	WNT7A	Wnt family member 7A(WNT7A)	ssc04310:Wnt signaling pathway,ssc04390:Hippo signaling pathway,ssc04550:Signaling pathways regulating pluripotency of stem cells,ssc04916:Melanogenesis,ssc05166:HTLV-I infection,ssc05200:Pathways in cancer,ssc05205:Proteoglycans in cancer,ssc05217:Basal cell carcinoma,
**DistUt**	**UP**	NCOR2	nuclear receptor corepressor 2(NCOR2)	ssc04330:Notch signaling pathway,ssc05169:Epstein-Barr virus infection,
**DistUt**	**UP**	NTRK3	neurotrophic receptor tyrosine kinase 3(NTRK3)	ssc04722:Neurotrophin signaling pathway,ssc05230:Central carbon metabolism in cancer,
**DistUt**	**UP**	LOC100152489	olfactory receptor 13C8(LOC100152489)	ssc04740:Olfactory transduction,
**DistUt**	**UP**	SLC15A1	solute carrier family 15 member 1(SLC15A1)	ssc04974:Protein digestion and absorption,
**DistUt**	**UP**	SLC7A7	solute carrier family 7 member 7(SLC7A7)	ssc04974:Protein digestion and absorption,
**DistUt**	**UP**	AQP9	aquaporin 9(AQP9)	ssc04976:Bile secretion,
**DistUt**	**UP**	SLC44A1	solute carrier family 44 member 1(SLC44A1)	ssc05231:Choline metabolism in cancer,
**DistUt**	**DOWN**	B4GALT1	beta-1,4-galactosyltransferase 1(B4GALT1)	ssc00052:Galactose metabolism,ssc00510:N-Glycan biosynthesis,ssc00514:Other types of O-glycan biosynthesis,ssc00533:Glycosaminoglycan biosynthesis - keratan sulfate,ssc00601:Glycosphingolipid biosynthesis - lacto and neolacto series,ssc01100:Metabolic pathways,
**DistUt**	**DOWN**	HSD11B1	hydroxysteroid 11-beta dehydrogenase 1(HSD11B1)	ssc00140:Steroid hormone biosynthesis,ssc00980:Metabolism of xenobiotics by cytochrome P450,ssc01100:Metabolic pathways,ssc05204:Chemical carcinogenesis,
**DistUt**	**DOWN**	GUCY1B3	guanylate cyclase 1, soluble, beta 3(GUCY1B3)	ssc00230:Purine metabolism,ssc04022:cGMP-PKG signaling pathway,ssc04270:Vascular smooth muscle contraction,ssc04540:Gap junction,ssc04611:Platelet activation,ssc04713:Circadian entrainment,ssc04730:Long-term depression,ssc04921:Oxytocin signaling pathway,ssc04924:Renin secretion,ssc04970:Salivary secretion,
**DistUt**	**DOWN**	NPR2	natriuretic peptide receptor B/guanylate cyclase B (atrionatriuretic peptide receptor B)(NPR2)	ssc00230:Purine metabolism,ssc04022:cGMP-PKG signaling pathway,ssc04270:Vascular smooth muscle contraction,ssc04921:Oxytocin signaling pathway,
**DistUt**	**DOWN**	PLOD2	procollagen-lysine,2-oxoglutarate 5-dioxygenase 2(PLOD2)	ssc00310:Lysine degradation,
**DistUt**	**DOWN**	GPX8	glutathione peroxidase 8 (putative)(GPX8)	ssc00480:Glutathione metabolism,ssc00590:Arachidonic acid metabolism,ssc04918:Thyroid hormone synthesis,
**DistUt**	**DOWN**	GCLM	glutamate-cysteine ligase modifier subunit(GCLM)	ssc00480:Glutathione metabolism,ssc01100:Metabolic pathways,
**DistUt**	**DOWN**	MAN2A1	mannosidase alpha class 2A member 1(MAN2A1)	ssc00510:N-Glycan biosynthesis,ssc01100:Metabolic pathways,
**DistUt**	**DOWN**	MGAT4D	MGAT4 family member D(MGAT4D)	ssc00510:N-Glycan biosynthesis,ssc01100:Metabolic pathways, \
**DistUt**	**DOWN**	GCNT3	glucosaminyl (N-acetyl) transferase 3, mucin type(GCNT3)	ssc00512:Mucin type O-Glycan biosynthesis,ssc01100:Metabolic pathways,
**DistUt**	**DOWN**	LOC100517735	chondroitin sulfate N-acetylgalactosaminyltransferase 1(LOC100517735)	ssc00532:Glycosaminoglycan biosynthesis - chondroitin sulfate / dermatan sulfate,ssc01100:Metabolic pathways, \
**DistUt**	**DOWN**	CHSY3	chondroitin sulfate synthase 3(CHSY3)	ssc00532:Glycosaminoglycan biosynthesis - chondroitin sulfate / dermatan sulfate,ssc01100:Metabolic pathways, \

**DistUt**	**DOWN**	LOC100518720	chondroitin sulfate synthase 3(LOC100518720)	ssc00532:Glycosaminoglycan biosynthesis - chondroitin sulfate / dermatan sulfate,ssc01100:Metabolic pathways,\
**DistUt**	**DOWN**	B3GALT2	beta-1,3-galactosyltransferase 2(B3GALT2)	ssc00601:Glycosphingolipid biosynthesis - lacto and neolacto series,ssc01100:Metabolic pathways,
**DistUt**	**DOWN**	ME1	malic enzyme 1(ME1)	ssc00620:Pyruvate metabolism,ssc01100:Metabolic pathways,ssc01200:Carbon metabolism,
**DistUt**	**DOWN**	ABCB1	ATP-binding cassette, sub-family B (MDR/TAP), member 1(ABCB1)	ssc02010:ABC transporters,ssc04976:Bile secretion,ssc05206:MicroRNAs in cancer,
**DistUt**	**DOWN**	REXO2	RNA exonuclease 2(REXO2)	ssc03008:Ribosome biogenesis in eukaryotes,\
**DistUt**	**DOWN**	PELO	pelota homolog (Drosophila)(PELO)	ssc03015:mRNA surveillance pathway,
**DistUt**	**DOWN**	LOC100517820	proteasome activator complex subunit 4(LOC100517820)	ssc03050:Proteasome,\
**DistUt**	**DOWN**	SEC63	SEC63 homolog, protein translocation regulator(SEC63)	ssc03060:Protein export,ssc04141:Protein processing in endoplasmic reticulum,
**DistUt**	**DOWN**	MMP1	matrix metallopeptidase 1(MMP1)	ssc03320:PPAR signaling pathway,ssc05200:Pathways in cancer,ssc05219:Bladder cancer,ssc05323:Rheumatoid arthritis,
**DistUt**	**DOWN**	FGFR1	fibroblast growth factor receptor 1(FGFR1)	ssc04010:MAPK signaling pathway,ssc04014:Ras signaling pathway,ssc04015:Rap1 signaling pathway,ssc04151:PI3K-Akt signaling pathway,ssc04520:Adherens junction,ssc04550:Signaling pathways regulating pluripotency of stem cells,ssc04810:Regulation of actin cytoskeleton,ssc05200:Pathways in cancer,ssc05205:Proteoglycans in cancer,ssc05215:Prostate cancer,ssc05218:Melanoma,ssc05230:Central carbon metabolism in cancer,
**DistUt**	**DOWN**	FGF2	fibroblast growth factor 2(FGF2)	ssc04010:MAPK signaling pathway,ssc04014:Ras signaling pathway,ssc04015:Rap1 signaling pathway,ssc04151:PI3K-Akt signaling pathway,ssc04550:Signaling pathways regulating pluripotency of stem cells,ssc04810:Regulation of actin cytoskeleton,ssc05200:Pathways in cancer,ssc05205:Proteoglycans in cancer,ssc05218:Melanoma,
**DistUt**	**DOWN**	PPP3CC	protein phosphatase 3 catalytic subunit gamma(PPP3CC)	ssc04010:MAPK signaling pathway,ssc04020:Calcium signaling pathway,ssc04022:cGMP-PKG signaling pathway,ssc04114:Oocyte meiosis,ssc04310:Wnt signaling pathway,ssc04360:Axon guidance,ssc04370:VEGF signaling pathway,ssc04380:Osteoclast differentiation,ssc04650:Natural killer cell mediated cytotoxicity,ssc04660:T cell receptor signaling pathway,ssc04662:B cell receptor signaling pathway,ssc04720:Long-term potentiation,ssc04724:Glutamatergic synapse,ssc04728:Dopaminergic synapse,ssc04921:Oxytocin signaling pathway,ssc04922:Glucagon signaling pathway,ssc04924:Renin secretion,ssc05010:Alzheimer's disease,ssc05014:Amyotrophic lateral sclerosis (ALS),ssc05031:Amphetamine addiction,ssc05152:Tuberculosis,ssc05166:HTLV-I infection,\
**DistUt**	**DOWN**	RPS6KA6	ribosomal protein S6 kinase A6(RPS6KA6)	ssc04010:MAPK signaling pathway,ssc04114:Oocyte meiosis,ssc04150:mTOR signaling pathway,ssc04720:Long-term potentiation,ssc04722:Neurotrophin signaling pathway,ssc04914:Progesterone-mediated oocyte maturation,ssc04931:Insulin resistance,
**DistUt**	**DOWN**	GNAI1	G protein subunit alpha i1(GNAI1)	ssc04015:Rap1 signaling pathway,ssc04022:cGMP-PKG signaling pathway,ssc04024:cAMP signaling pathway,ssc04062:Chemokine signaling pathway,ssc04071:Sphingolipid signaling pathway,ssc04261:Adrenergic signaling in cardiomyocytes,ssc04360:Axon guidance,ssc04540:Gap junction,ssc04611:Platelet activation,ssc04670:Leukocyte transendothelial migration,ssc04713:Circadian entrainment,ssc04723:Retrograde endocannabinoid signaling,ssc04724:Glutamatergic synapse,ssc04725:Cholinergic synapse,ssc04726:Serotonergic synapse,ssc04727:GABAergic synapse,ssc04728:Dopaminergic synapse,ssc04730:Long-term depression,ssc04914:Progesterone-mediated oocyte maturation,ssc04915:Estrogen signaling pathway,ssc04916:Melanogenesis,ssc04921:Oxytocin signaling pathway,ssc04923:Regulation of lipolysis in adipocytes,ssc04924:Renin secretion,ssc04971:Gastric acid secretion,ssc05012:Parkinson's disease,ssc05030:Cocaine addiction,ssc05032:Morphine addiction,ssc05034:Alcoholism,ssc05133:Pertussis,ssc05142:Chagas disease (American trypanosomiasis),ssc05145:Toxoplasmosis,ssc05200:Pathways in cancer,
**DistUt**	**DOWN**	AGTR1	angiotensin II receptor type 1(AGTR1)	ssc04020:Calcium signaling pathway,ssc04022:cGMP-PKG signaling pathway,ssc04080:Neuroactive ligand-receptor interaction,ssc04261:Adrenergic signaling in cardiomyocytes,ssc04270:Vascular smooth muscle contraction,ssc04614:Renin-angiotensin system,ssc04924:Renin secretion,ssc04925:Aldosterone synthesis and secretion,ssc05200:Pathways in cancer,
**DistUt**	**DOWN**	PTGFR	prostaglandin F receptor(PTGFR)	ssc04020:Calcium signaling pathway,ssc04080:Neuroactive ligand-receptor interaction,
**DistUt**	**DOWN**	HTR2A	5-hydroxytryptamine receptor 2A(HTR2A)	ssc04020:Calcium signaling pathway,ssc04080:Neuroactive ligand-receptor interaction,ssc04540:Gap junction,ssc04726:Serotonergic synapse,ssc04750:Inflammatory mediator regulation of TRP channels,
**DistUt**	**DOWN**	HRH1	histamine receptor H1(HRH1)	ssc04020:Calcium signaling pathway,ssc04080:Neuroactive ligand-receptor interaction,ssc04750:Inflammatory mediator regulation of TRP channels,
**DistUt**	**DOWN**	CREB3L2	cAMP responsive element binding protein 3 like 2(CREB3L2)	ssc04022:cGMP-PKG signaling pathway,ssc04024:cAMP signaling pathway,ssc04151:PI3K-Akt signaling pathway,ssc04152:AMPK signaling pathway,ssc04261:Adrenergic signaling in cardiomyocytes,ssc04668:TNF signaling pathway,ssc04725:Cholinergic synapse,ssc04728:Dopaminergic synapse,ssc04911:Insulin secretion,ssc04915:Estrogen signaling pathway,ssc04916:Melanogenesis,ssc04918:Thyroid hormone synthesis,ssc04922:Glucagon signaling pathway,ssc04925:Aldosterone synthesis and secretion,ssc04931:Insulin resistance,ssc04962:Vasopressin-regulated water reabsorption,ssc05016:Huntington's disease,ssc05030:Cocaine addiction,ssc05031:Amphetamine addiction,ssc05034:Alcoholism,ssc05161:Hepatitis B,ssc05203:Viral carcinogenesis,ssc05215:Prostate cancer,
**DistUt**	**DOWN**	ACKR3	atypical chemokine receptor 3(ACKR3)	ssc04060:Cytokine-cytokine receptor interaction,
**DistUt**	**DOWN**	PLAU	plasminogen activator, urokinase(PLAU)	ssc04064:NF-kappa B signaling pathway,ssc04610:Complement and coagulation cascades,ssc05202:Transcriptional misregulation in cancer,ssc05205:Proteoglycans in cancer,ssc05206:MicroRNAs in cancer,
**DistUt**	**DOWN**	HIF1A	hypoxia inducible factor 1, alpha subunit (basic helix-loop-helix transcription factor)(HIF1A)	ssc04066:HIF-1 signaling pathway,ssc04919:Thyroid hormone signaling pathway,ssc05200:Pathways in cancer,ssc05205:Proteoglycans in cancer,ssc05211:Renal cell carcinoma,ssc05230:Central carbon metabolism in cancer,ssc05231:Choline metabolism in cancer,
**DistUt**	**DOWN**	S1PR3	sphingosine-1-phosphate receptor 3(S1PR3)	ssc04071:Sphingolipid signaling pathway,ssc04080:Neuroactive ligand-receptor interaction,
**DistUt**	**DOWN**	PMAIP1	phorbol-12-myristate-13-acetate-induced protein 1(PMAIP1)	ssc04115:p53 signaling pathway,ssc05203:Viral carcinogenesis,\
**DistUt**	**DOWN**	EDEM3	ER degradation enhancing alpha-mannosidase like protein 3(EDEM3)	ssc04141:Protein processing in endoplasmic reticulum,
**DistUt**	**DOWN**	SEC23A	Sec23 homolog A, coat complex II component(SEC23A)	ssc04141:Protein processing in endoplasmic reticulum,
**DistUt**	**DOWN**	SEC23B	Sec23 homolog B, coat complex II component(SEC23B)	ssc04141:Protein processing in endoplasmic reticulum,
**DistUt**	**DOWN**	ERLEC1	endoplasmic reticulum lectin 1(ERLEC1)	ssc04141:Protein processing in endoplasmic reticulum,
**DistUt**	**DOWN**	ERO1A	endoplasmic reticulum oxidoreductase 1 alpha(ERO1A)	ssc04141:Protein processing in endoplasmic reticulum,
**DistUt**	**DOWN**	UGGT2	UDP-glucose glycoprotein glucosyltransferase 2(UGGT2)	ssc04141:Protein processing in endoplasmic reticulum,\
**DistUt**	**DOWN**	PDIA3	protein disulfide isomerase family A member 3(PDIA3)	ssc04141:Protein processing in endoplasmic reticulum,ssc04612:Antigen processing and presentation,
**DistUt**	**DOWN**	EIF2AK3	eukaryotic translation initiation factor 2 alpha kinase 3(EIF2AK3)	ssc04141:Protein processing in endoplasmic reticulum,ssc04932:Non-alcoholic fatty liver disease (NAFLD),ssc05010:Alzheimer's disease,ssc05160:Hepatitis C,ssc05162:Measles,ssc05164:Influenza A,ssc05168:Herpes simplex infection,
**DistUt**	**DOWN**	MRC2	mannose receptor C type 2(MRC2)	ssc04145:Phagosome,ssc05152:Tuberculosis,
**DistUt**	**DOWN**	COL1A2	collagen type I alpha 2 chain(COL1A2)	ssc04151:PI3K-Akt signaling pathway,ssc04510:Focal adhesion,ssc04512:ECM-receptor interaction,ssc04611:Platelet activation,ssc04974:Protein digestion and absorption,ssc05146:Amoebiasis,
**DistUt**	**DOWN**	COL3A1	collagen type III alpha 1 chain(COL3A1)	ssc04151:PI3K-Akt signaling pathway,ssc04510:Focal adhesion,ssc04512:ECM-receptor interaction,ssc04611:Platelet activation,ssc04974:Protein digestion and absorption,ssc05146:Amoebiasis,
**DistUt**	**DOWN**	COL5A2	collagen type V alpha 2 chain(COL5A2)	ssc04151:PI3K-Akt signaling pathway,ssc04510:Focal adhesion,ssc04512:ECM-receptor interaction,ssc04611:Platelet activation,ssc04974:Protein digestion and absorption,ssc05146:Amoebiasis,
**DistUt**	**DOWN**	COL6A3	collagen type VI alpha 3 chain(COL6A3)	ssc04151:PI3K-Akt signaling pathway,ssc04510:Focal adhesion,ssc04512:ECM-receptor interaction,ssc04974:Protein digestion and absorption,
**DistUt**	**DOWN**	COL4A1	collagen type IV alpha 1 chain(COL4A1)	ssc04151:PI3K-Akt signaling pathway,ssc04510:Focal adhesion,ssc04512:ECM-receptor interaction,ssc04974:Protein digestion and absorption,ssc05146:Amoebiasis,ssc05200:Pathways in cancer,ssc05222:Small cell lung cancer,

**DistUt**	**DOWN**	TCF7L2	transcription factor 7 like 2(TCF7L2)	ssc04310:Wnt signaling pathway,ssc04390:Hippo signaling pathway,ssc04520:Adherens junction,ssc04916:Melanogenesis,ssc05200:Pathways in cancer,ssc05210:Colorectal cancer,ssc05213:Endometrial cancer,ssc05215:Prostate cancer,ssc05216:Thyroid cancer,ssc05217:Basal cell carcinoma,ssc05221:Acute myeloid leukemia,ssc05412:Arrhythmogenic right ventricular cardiomyopathy (ARVC),
**DistUt**	**DOWN**	WNT2	Wnt family member 2(WNT2)	ssc04310:Wnt signaling pathway,ssc04390:Hippo signaling pathway,ssc04550:Signaling pathways regulating pluripotency of stem cells,ssc04916:Melanogenesis,ssc05166:HTLV-I infection,ssc05200:Pathways in cancer,ssc05205:Proteoglycans in cancer,ssc05217:Basal cell carcinoma,
**DistUt**	**DOWN**	DCN	decorin(DCN)	ssc04350:TGF-beta signaling pathway,ssc05205:Proteoglycans in cancer,
**DistUt**	**DOWN**	EPHA4	EPH receptor A4(EPHA4)	ssc04360:Axon guidance,
**DistUt**	**DOWN**	CFL2	cofilin 2(CFL2)	ssc04360:Axon guidance,ssc04666:Fc gamma R-mediated phagocytosis,ssc04810:Regulation of actin cytoskeleton,ssc05133:Pertussis,
**DistUt**	**DOWN**	ALCAM	activated leukocyte cell adhesion molecule(ALCAM)	ssc04514:Cell adhesion molecules (CAMs),
**DistUt**	**DOWN**	VCAN	versican(VCAN)	ssc04514:Cell adhesion molecules (CAMs),
**DistUt**	**DOWN**	CLDN11	claudin 11(CLDN11)	ssc04514:Cell adhesion molecules (CAMs),ssc04530:Tight junction,ssc04670:Leukocyte transendothelial migration,ssc05160:Hepatitis C,
**DistUt**	**DOWN**	SDC2	syndecan 2(SDC2)	ssc04514:Cell adhesion molecules (CAMs),ssc05144:Malaria,ssc05205:Proteoglycans in cancer,
**DistUt**	**DOWN**	GJA1	gap junction protein alpha 1(GJA1)	ssc04540:Gap junction,ssc05412:Arrhythmogenic right ventricular cardiomyopathy (ARVC),
**DistUt**	**DOWN**	PLAUR	plasminogen activator, urokinase receptor(PLAUR)	ssc04610:Complement and coagulation cascades,ssc05205:Proteoglycans in cancer,
**DistUt**	**DOWN**	ENPEP	glutamyl aminopeptidase(ENPEP)	ssc04614:Renin-angiotensin system,
**DistUt**	**DOWN**	MARCKS	myristoylated alanine rich protein kinase C substrate(MARCKS)	ssc04666:Fc gamma R-mediated phagocytosis,ssc05206:MicroRNAs in cancer, \
**DistUt**	**DOWN**	MMP3	matrix metallopeptidase 3 (stromelysin 1, progelatinase)(MMP3)	ssc04668:TNF signaling pathway,ssc05202:Transcriptional misregulation in cancer,ssc05323:Rheumatoid arthritis,
**DistUt**	**DOWN**	MAGED1	MAGE family member D1(MAGED1)	ssc04722:Neurotrophin signaling pathway,
**DistUt**	**DOWN**	SLC38A1	solute carrier family 38 member 1(SLC38A1)	ssc04724:Glutamatergic synapse,ssc04727:GABAergic synapse,
**DistUt**	**DOWN**	SLC38A2	solute carrier family 38 member 2(SLC38A2)	ssc04724:Glutamatergic synapse,ssc04727:GABAergic synapse,ssc04974:Protein digestion and absorption,
**DistUt**	**DOWN**	LOC100157704	olfactory receptor 1L8-like(LOC100157704)	ssc04740:Olfactory transduction,
**DistUt**	**DOWN**	RCAN2	regulator of calcineurin 2(RCAN2)	ssc04919:Thyroid hormone signaling pathway,
**DistUt**	**DOWN**	LOC100512644	uncharacterized LOC100512644(LOC100512644)	ssc04974:Protein digestion and absorption, \
**DistUt**	**DOWN**	NCEH1	neutral cholesterol ester hydrolase 1(NCEH1)	ssc04976:Bile secretion,
**DistUt**	**DOWN**	STEAP2	STEAP2 metalloreductase(STEAP2)	ssc04978:Mineral absorption,
**DistUt**	**DOWN**	TGM2	transglutaminase 2(TGM2)	ssc05016:Huntington's disease,
**DistUt**	**DOWN**	PRNP	prion protein(PRNP)	ssc05020:Prion diseases,
**DistUt**	**DOWN**	HCFC2	host cell factor C2(HCFC2)	ssc05168:Herpes simplex infection,
**DistUt**	**DOWN**	AFF1	AF4/FMR2 family member 1(AFF1)	ssc05202:Transcriptional misregulation in cancer, \
**ProxUt**	**UP**	ALDH2	aldehyde dehydrogenase 2 family (mitochondrial)(ALDH2)	ssc00010:Glycolysis / Gluconeogenesis,ssc00053:Ascorbate and aldarate metabolism,ssc00071:Fatty acid degradation,ssc00280:Valine, leucine and isoleucine degradation,ssc00310:Lysine degradation,ssc00330:Arginine and proline metabolism,ssc00340:Histidine metabolism,ssc00380:Tryptophan metabolism,ssc00410:beta-Alanine metabolism,ssc00561:Glycerolipid metabolism,ssc00620:Pyruvate metabolism,ssc01100:Metabolic pathways,ssc01130:Biosynthesis of antibiotics,
**ProxUt**	**UP**	CRYL1	crystallin lambda 1(CRYL1)	ssc00040:Pentose and glucuronate interconversions,ssc01100:Metabolic pathways,
**ProxUt**	**UP**	KHK	ketohexokinase(KHK)	ssc00051:Fructose and mannose metabolism,ssc01100:Metabolic pathways,
**ProxUt**	**UP**	GALK1	galactokinase 1(GALK1)	ssc00052:Galactose metabolism,ssc00520:Amino sugar and nucleotide sugar metabolism,ssc01100:Metabolic pathways,
**ProxUt**	**UP**	LOC100515137	5-oxoprolinase-like(LOC100515137)	ssc00480:Glutathione metabolism, \
**ProxUt**	**UP**	CHST4	carbohydrate sulfotransferase 4(CHST4)	ssc00533:Glycosaminoglycan biosynthesis - keratan sulfate,
**ProxUt**	**UP**	DGAT1	diacylglycerol O-acyltransferase 1(DGAT1)	ssc00561:Glycerolipid metabolism,ssc00830:Retinol metabolism,ssc01100:Metabolic pathways,ssc04975:Fat digestion and absorption,
**ProxUt**	**UP**	MTMR7	myotubularin related protein 7(MTMR7)	ssc00562:Inositol phosphate metabolism,ssc01100:Metabolic pathways,ssc04070:Phosphatidylinositol signaling system,
**ProxUt**	**UP**	LOC100739011	putative lipoyltransferase 2, mitochondrial(LOC100739011)	ssc00785:Lipoic acid metabolism,ssc01100:Metabolic pathways,
**ProxUt**	**UP**	SPR	sepiapterin reductase (7,8-dihydrobiopterin:NADP+ oxidoreductase)(SPR)	ssc00790:Folate biosynthesis,ssc01100:Metabolic pathways,
**ProxUt**	**UP**	SDR16C5	short chain dehydrogenase/reductase family 16C, member 5(SDR16C5)	ssc00830:Retinol metabolism,
**ProxUt**	**UP**	XAB2	XPA binding protein 2(XAB2)	ssc03040:Spliceosome,
**ProxUt**	**UP**	LOC100516539	splicing factor 3A subunit 2(LOC100516539)	ssc03040:Spliceosome, \
**ProxUt**	**UP**	DDB1	damage specific DNA binding protein 1(DDB1)	ssc03420:Nucleotide excision repair,ssc04120:Ubiquitin mediated proteolysis,ssc05161:Hepatitis B,ssc05203:Viral carcinogenesis,
**ProxUt**	**UP**	HSPB1	heat shock protein family B (small) member 1(HSPB1)	ssc04010:MAPK signaling pathway,ssc04370:VEGF signaling pathway,ssc05146:Amoebiasis,
**ProxUt**	**UP**	MAPT	microtubule associated protein tau(MAPT)	ssc04010:MAPK signaling pathway,ssc05010:Alzheimer's disease,
**ProxUt**	**UP**	CALM1	calmodulin 1(CALM1)	ssc04014:Ras signaling pathway,ssc04015:Rap1 signaling pathway,ssc04020:Calcium signaling pathway,ssc04022:cGMP-PKG signaling pathway,ssc04024:cAMP signaling pathway,ssc04070:Phosphatidylinositol signaling system,ssc04114:Oocyte meiosis,ssc04261:Adrenergic signaling in cardiomyocytes,ssc04270:Vascular smooth muscle contraction,ssc04713:Circadian entrainment,ssc04720:Long-term potentiation,ssc04722:Neurotrophin signaling pathway,ssc04728:Dopaminergic synapse,ssc04740:Olfactory transduction,ssc04744:Phototransduction,ssc04750:Inflammatory mediator regulation of TRP channels,ssc04910:Insulin signaling pathway,ssc04912:GnRH signaling pathway,ssc04915:Estrogen signaling pathway,ssc04916:Melanogenesis,ssc04921:Oxytocin signaling pathway,ssc04922:Glucagon signaling pathway,ssc04924:Renin secretion,ssc04925:Aldosterone synthesis and secretion,ssc04970:Salivary secretion,ssc04971:Gastric acid secretion,ssc05010:Alzheimer's disease,ssc05031:Amphetamine addiction,ssc05034:Alcoholism,ssc05133:Pertussis,ssc05152:Tuberculosis,ssc05214:Glioma,
**ProxUt**	**UP**	ADRA1A	adrenoceptor alpha 1A(ADRA1A)	ssc04020:Calcium signaling pathway,ssc04022:cGMP-PKG signaling pathway,ssc04080:Neuroactive ligand-receptor interaction,ssc04152:AMPK signaling pathway,ssc04261:Adrenergic signaling in cardiomyocytes,ssc04270:Vascular smooth muscle contraction,ssc04970:Salivary secretion,
**ProxUt**	**UP**	ADRA2A	adrenoceptor alpha 2A(ADRA2A)	ssc04022:cGMP-PKG signaling pathway,ssc04080:Neuroactive ligand-receptor interaction,

**ProxUt**	**UP**	GRK1	G protein-coupled receptor kinase 1(GRK1)	ssc04062:Chemokine signaling pathway,ssc04144:Endocytosis,ssc04744:Phototransduction,
**ProxUt**	**UP**	STK11	serine/threonine kinase 11(STK11)	ssc04068:FoxO signaling pathway,ssc04150:mTOR signaling pathway,ssc04151:PI3K-Akt signaling pathway,ssc04152:AMPK signaling pathway,ssc04920:Adipocytokine signaling pathway,
**ProxUt**	**UP**	RXFP4	relaxin/insulin like family peptide receptor 4(RXFP4)	ssc04080:Neuroactive ligand-receptor interaction,
**ProxUt**	**UP**	VIPR1	vasoactive intestinal peptide receptor 1(VIPR1)	ssc04080:Neuroactive ligand-receptor interaction,
**ProxUt**	**UP**	LOC100738970	CXXC-type zinc finger protein 4(LOC100738970)	ssc04310:Wnt signaling pathway,\
**ProxUt**	**UP**	KCNQ4	potassium voltage-gated channel subfamily Q member 4(KCNQ4)	ssc04725:Cholinergic synapse,
**ProxUt**	**UP**	LOC100524975	olfactory receptor 10H1(LOC100524975)	ssc04740:Olfactory transduction,
**ProxUt**	**UP**	LOC100516511	olfactory receptor 1A1(LOC100516511)	ssc04740:Olfactory transduction,
**ProxUt**	**UP**	LOC100521466	olfactory receptor 52L1(LOC100521466)	ssc04740:Olfactory transduction,\
**ProxUt**	**UP**	LOC100517804	putative olfactory receptor 5AK3(LOC100517804)	ssc04740:Olfactory transduction,\
**ProxUt**	**UP**	TMSB4X	thymosin beta 4, X-linked(TMSB4X)	ssc04810:Regulation of actin cytoskeleton,
**ProxUt**	**UP**	ASGR2	asialoglycoprotein receptor 2(ASGR2)	ssc04918:Thyroid hormone synthesis,
**ProxUt**	**UP**	MLXIP	MLX interacting protein(MLXIP)	ssc04931:Insulin resistance,ssc04932:Non-alcoholic fatty liver disease (NAFLD),
**ProxUt**	**UP**	HSF1	heat shock transcription factor 1(HSF1)	ssc05134:Legionellosis,
**ProxUt**	**DOWN**	B4GALT1	beta-1,4-galactosyltransferase 1(B4GALT1)	ssc00052:Galactose metabolism,ssc00510:N-Glycan biosynthesis,ssc00514:Other types of O-glycan biosynthesis,ssc00533:Glycosaminoglycan biosynthesis - keratan sulfate,ssc00601:Glycosphingolipid biosynthesis - lacto and neolacto series,ssc01100:Metabolic pathways,
**ProxUt**	**DOWN**	HSD11B1	hydroxysteroid 11-beta dehydrogenase 1(HSD11B1)	ssc00140:Steroid hormone biosynthesis,ssc00980:Metabolism of xenobiotics by cytochrome P450,ssc01100:Metabolic pathways,ssc05204:Chemical carcinogenesis,
**ProxUt**	**DOWN**	ENTPD5	ectonucleoside triphosphate diphosphohydrolase 5(ENTPD5)	ssc00230:Purine metabolism,ssc00240:Pyrimidine metabolism,
**ProxUt**	**DOWN**	PDE7B	phosphodiesterase 7B(PDE7B)	ssc00230:Purine metabolism,ssc05032:Morphine addiction,
**ProxUt**	**DOWN**	DCTD	dCMP deaminase(DCTD)	ssc00240:Pyrimidine metabolism,ssc01100:Metabolic pathways,
**ProxUt**	**DOWN**	PLOD2	procollagen-lysine,2-oxoglutarate 5-dioxygenase 2(PLOD2)	ssc00310:Lysine degradation,
**ProxUt**	**DOWN**	GPX8	glutathione peroxidase 8 (putative)(GPX8)	ssc00480:Glutathione metabolism,ssc00590:Arachidonic acid metabolism,ssc04918:Thyroid hormone synthesis,
**ProxUt**	**DOWN**	MAN2A1	mannosidase alpha class 2A member 1(MAN2A1)	ssc00510:N-Glycan biosynthesis,ssc01100:Metabolic pathways,
**ProxUt**	**DOWN**	MGAT4D	MGAT4 family member D(MGAT4D)	ssc00510:N-Glycan biosynthesis,ssc01100:Metabolic pathways,\
**ProxUt**	**DOWN**	MAN1A1	mannosidase alpha class 1A member 1(MAN1A1)	ssc00510:N-Glycan biosynthesis,ssc01100:Metabolic pathways,ssc04141:Protein processing in endoplasmic reticulum,
**ProxUt**	**DOWN**	GCNT3	glucosaminyl (N-acetyl) transferase 3, mucin type(GCNT3)	ssc00512:Mucin type O-Glycan biosynthesis,ssc01100:Metabolic pathways,
**ProxUt**	**DOWN**	CHSY3	chondroitin sulfate synthase 3(CHSY3)	ssc00532:Glycosaminoglycan biosynthesis - chondroitin sulfate / dermatan sulfate,ssc01100:Metabolic pathways,\
**ProxUt**	**DOWN**	LOC100518720	chondroitin sulfate synthase 3(LOC100518720)	ssc00532:Glycosaminoglycan biosynthesis - chondroitin sulfate / dermatan sulfate,ssc01100:Metabolic pathways,\
**ProxUt**	**DOWN**	B3GNT2	UDP-GlcNAc:betaGal beta-1,3-N-acetylglucosaminyltransferase 2(B3GNT2)	ssc00533:Glycosaminoglycan biosynthesis - keratan sulfate,ssc00601:Glycosphingolipid biosynthesis - lacto and neolacto series,ssc01100:Metabolic pathways,
**ProxUt**	**DOWN**	GPD2	glycerol-3-phosphate dehydrogenase 2(GPD2)	ssc00564:Glycerophospholipid metabolism,
**ProxUt**	**DOWN**	B3GALT2	beta-1,3-galactosyltransferase 2(B3GALT2)	ssc00601:Glycosphingolipid biosynthesis - lacto and neolacto series,ssc01100:Metabolic pathways,
**ProxUt**	**DOWN**	VNN1	vanin 1(VNN1)	ssc00770:Pantothenate and CoA biosynthesis,
**ProxUt**	**DOWN**	ABCB1	ATP-binding cassette, sub-family B (MDR/TAP), member 1(ABCB1)	ssc02010:ABC transporters,ssc04976:Bile secretion,ssc05206:MicroRNAs in cancer,
**ProxUt**	**DOWN**	SRP54	signal recognition particle 54(SRP54)	ssc03060:Protein export,
**ProxUt**	**DOWN**	MMP1	matrix metallopeptidase 1(MMP1)	ssc03320:PPAR signaling pathway,ssc05200:Pathways in cancer,ssc05219:Bladder cancer,ssc05323:Rheumatoid arthritis,
**ProxUt**	**DOWN**	LOC100736973	ras-related protein Rap-1b(LOC100736973)	ssc04010:MAPK signaling pathway,ssc04014:Ras signaling pathway,ssc04015:Rap1 signaling pathway,ssc04024:cAMP signaling pathway,ssc04062:Chemokine signaling pathway,ssc04510:Focal adhesion,ssc04611:Platelet activation,ssc04670:Leukocyte transendothelial migration,ssc04720:Long-term potentiation,ssc04722:Neurotrophin signaling pathway,ssc04972:Pancreatic secretion,ssc05211:Renal cell carcinoma,
**ProxUt**	**DOWN**	PPP3CC	protein phosphatase 3 catalytic subunit gamma(PPP3CC)	ssc04010:MAPK signaling pathway,ssc04020:Calcium signaling pathway,ssc04022:cGMP-PKG signaling pathway,ssc04114:Oocyte meiosis,ssc04310:Wnt signaling pathway,ssc04360:Axon guidance,ssc04370:VEGF signaling pathway,ssc04380:Osteoclast differentiation,ssc04650:Natural killer cell mediated cytotoxicity,ssc04660:T cell receptor signaling pathway,ssc04662:B cell receptor signaling pathway,ssc04720:Long-term potentiation,ssc04724:Glutamatergic synapse,ssc04728:Dopaminergic synapse,ssc04921:Oxytocin signaling pathway,ssc04922:Glucagon signaling pathway,ssc04924:Renin secretion,ssc05010:Alzheimer's disease,ssc05014:Amyotrophic lateral sclerosis (ALS),ssc05031:Amphetamine addiction,ssc05152:Tuberculosis,ssc05166:HTLV-I infection,\
**ProxUt**	**DOWN**	LOC100623707	phosphatidylinositol 3-kinase regulatory subunit alpha-like(LOC100623707)	ssc04012:ErbB signaling pathway,ssc04014:Ras signaling pathway,ssc04015:Rap1 signaling pathway,ssc04024:cAMP signaling pathway,ssc04062:Chemokine signaling pathway,ssc04066:HIF-1 signaling pathway,ssc04068:FoxO signaling pathway,ssc04070:Phosphatidylinositol signaling system,ssc04071:Sphingolipid signaling pathway,ssc04150:mTOR signaling pathway,ssc04151:PI3K-Akt signaling pathway,ssc04152:AMPK signaling pathway,ssc04210:Apoptosis,ssc04370:VEGF signaling pathway,ssc04380:Osteoclast differentiation,ssc04510:Focal adhesion,ssc04550:Signaling pathways regulating pluripotency of stem cells,ssc04611:Platelet activation,ssc04620:Toll-like receptor signaling pathway,ssc04630:Jak-STAT signaling pathway,ssc04650:Natural killer cell mediated cytotoxicity,ssc04660:T cell receptor signaling pathway,ssc04662:B cell receptor signaling pathway,ssc04664:Fc epsilon RI signaling pathway,ssc04666:Fc gamma R-mediated phagocytosis,ssc04668:TNF signaling pathway,ssc04670:Leukocyte transendothelial migration,ssc04722:Neurotrophin signaling pathway,ssc04725:Cholinergic synapse,ssc04750:Inflammatory mediator regulation of TRP channels,ssc04810:Regulation of actin cytoskeleton,ssc04910:Insulin signaling pathway,ssc04914:Progesterone-mediated oocyte maturation,ssc04915:Estrogen signaling pathway,ssc04917:Prolactin signaling pathway,ssc04919:Thyroid hormone signaling pathway,ssc04923:Regulation of lipolysis in adipocytes,ssc04930:Type II diabetes mellitus,ssc04931:Insulin resistance,ssc04932:Non-alcoholic fatty liver disease (NAFLD),ssc04960:Aldosterone-regulated sodium reabsorption,ssc04973:Carbohydrate digestion and absorption,ssc05100:Bacterial invasion of epithelial cells,ssc05142:Chagas disease (American trypanosomiasis),ssc05146:Amoebiasis,ssc05160:Hepatitis C,ssc05161:Hepatitis B,ssc05162:Measles,ssc05164:Influenza A,ssc05166:HTLV-I infection,ssc05169:Epstein-Barr virus infection,ssc05200:Pathways in cancer,ssc05203:Viral carcinogenesis,ssc05205:Proteoglycans in cancer,ssc05210:Colorectal cancer,ssc05211:Renal cell carcinoma,ssc05212:Pancreatic cancer,ssc05213:Endometrial cancer,ssc05214:Glioma,ssc05215:Prostate cancer,ssc05218:Melanoma,ssc05220:Chronic myeloid leukemia,ssc05221:Acute myeloid leukemia,ssc05222:Small cell lung cancer,ssc05223:Non-small cell lung cancer,ssc05230:Central carbon metabolism in cancer,ssc05231:Choline metabolism in cancer,
**ProxUt**	**DOWN**	KDR	kinase insert domain receptor(KDR)	ssc04014:Ras signaling pathway,ssc04015:Rap1 signaling pathway,ssc04151:PI3K-Akt signaling pathway,ssc04370:VEGF signaling pathway,ssc04510:Focal adhesion,ssc05205:Proteoglycans in cancer,

**ProxUt**	**DOWN**	SIPA1L1	signal induced proliferation associated 1 like 1(SIPA1L1)	ssc04015:Rap1 signaling pathway,
**ProxUt**	**DOWN**	GNAI1	G protein subunit alpha i1(GNAI1)	ssc04015:Rap1 signaling pathway,ssc04022:cGMP-PKG signaling pathway,ssc04024:cAMP signaling pathway,ssc04062:Chemokine signaling pathway,ssc04071:Sphingolipid signaling pathway,ssc04261:Adrenergic signaling in cardiomyocytes,ssc04360:Axon guidance,ssc04540:Gap junction,ssc04611:Platelet activation,ssc04670:Leukocyte transendothelial migration,ssc04713:Circadian entrainment,ssc04723:Retrograde endocannabinoid signaling,ssc04724:Glutamatergic synapse,ssc04725:Cholinergic synapse,ssc04726:Serotonergic synapse,ssc04727:GABAergic synapse,ssc04728:Dopaminergic synapse,ssc04730:Long-term depression,ssc04914:Progesterone-mediated oocyte maturation,ssc04915:Estrogen signaling pathway,ssc04916:Melanogenesis,ssc04921:Oxytocin signaling pathway,ssc04923:Regulation of lipolysis in adipocytes,ssc04924:Renin secretion,ssc04971:Gastric acid secretion,ssc05012:Parkinson's disease,ssc05030:Cocaine addiction,ssc05032:Morphine addiction,ssc05034:Alcoholism,ssc05133:Pertussis,ssc05142:Chagas disease (American trypanosomiasis),ssc05145:Toxoplasmosis,ssc05200:Pathways in cancer,
**ProxUt**	**DOWN**	AGTR1	angiotensin II receptor type 1(AGTR1)	ssc04020:Calcium signaling pathway,ssc04022:cGMP-PKG signaling pathway,ssc04080:Neuroactive ligand-receptor interaction,ssc04261:Adrenergic signaling in cardiomyocytes,ssc04270:Vascular smooth muscle contraction,ssc04614:Renin-angiotensin system,ssc04924:Renin secretion,ssc04925:Aldosterone synthesis and secretion,ssc05200:Pathways in cancer,
**ProxUt**	**DOWN**	P2RX7	purinergic receptor P2X 7(P2RX7)	ssc04020:Calcium signaling pathway,ssc04080:Neuroactive ligand-receptor interaction,
**ProxUt**	**DOWN**	CREB3L1	cAMP responsive element binding protein 3 like 1(CREB3L1)	ssc04022:cGMP-PKG signaling pathway,ssc04024:cAMP signaling pathway,ssc04151:PI3K-Akt signaling pathway,ssc04152:AMPK signaling pathway,ssc04261:Adrenergic signaling in cardiomyocytes,ssc04668:TNF signaling pathway,ssc04725:Cholinergic synapse,ssc04728:Dopaminergic synapse,ssc04911:Insulin secretion,ssc04915:Estrogen signaling pathway,ssc04916:Melanogenesis,ssc04918:Thyroid hormone synthesis,ssc04922:Glucagon signaling pathway,ssc04925:Aldosterone synthesis and secretion,ssc04931:Insulin resistance,ssc04962:Vasopressin-regulated water reabsorption,ssc05016:Huntington's disease,ssc05030:Cocaine addiction,ssc05031:Amphetamine addiction,ssc05034:Alcoholism,ssc05161:Hepatitis B,ssc05203:Viral carcinogenesis,ssc05215:Prostate cancer,
**ProxUt**	**DOWN**	CREB3L2	cAMP responsive element binding protein 3 like 2(CREB3L2)	ssc04022:cGMP-PKG signaling pathway,ssc04024:cAMP signaling pathway,ssc04151:PI3K-Akt signaling pathway,ssc04152:AMPK signaling pathway,ssc04261:Adrenergic signaling in cardiomyocytes,ssc04668:TNF signaling pathway,ssc04725:Cholinergic synapse,ssc04728:Dopaminergic synapse,ssc04911:Insulin secretion,ssc04915:Estrogen signaling pathway,ssc04916:Melanogenesis,ssc04918:Thyroid hormone synthesis,ssc04922:Glucagon signaling pathway,ssc04925:Aldosterone synthesis and secretion,ssc04931:Insulin resistance,ssc04962:Vasopressin-regulated water reabsorption,ssc05016:Huntington's disease,ssc05030:Cocaine addiction,ssc05031:Amphetamine addiction,ssc05034:Alcoholism,ssc05161:Hepatitis B,ssc05203:Viral carcinogenesis,ssc05215:Prostate cancer,
**ProxUt**	**DOWN**	ACKR3	atypical chemokine receptor 3(ACKR3)	ssc04060:Cytokine-cytokine receptor interaction,
**ProxUt**	**DOWN**	STAT2	signal transducer and activator of transcription 2(STAT2)	ssc04062:Chemokine signaling pathway,ssc04380:Osteoclast differentiation,ssc04630:Jak-STAT signaling pathway,ssc05160:Hepatitis C,ssc05161:Hepatitis B,ssc05162:Measles,ssc05164:Influenza A,ssc05168:Herpes simplex infection,
**ProxUt**	**DOWN**	PLAU	plasminogen activator, urokinase(PLAU)	ssc04064:NF-kappa B signaling pathway,ssc04610:Complement and coagulation cascades,ssc05202:Transcriptional misregulation in cancer,ssc05205:Proteoglycans in cancer,ssc05206:MicroRNAs in cancer,
**ProxUt**	**DOWN**	IRAK4	interleukin 1 receptor associated kinase 4(IRAK4)	ssc04064:NF-kappa B signaling pathway,ssc04620:Toll-like receptor signaling pathway,ssc04722:Neurotrophin signaling pathway,ssc05133:Pertussis,ssc05140:Leishmaniasis,ssc05142:Chagas disease (American trypanosomiasis),ssc05145:Toxoplasmosis,ssc05152:Tuberculosis,ssc05162:Measles,ssc05164:Influenza A,
**ProxUt**	**DOWN**	PMAIP1	phorbol-12-myristate-13-acetate-induced protein 1(PMAIP1)	ssc04115:p53 signaling pathway,ssc05203:Viral carcinogenesis, \
**ProxUt**	**DOWN**	SEC22B	SEC22 homolog B, vesicle trafficking protein(SEC22B)	ssc04130:SNARE interactions in vesicular transport,ssc04145:Phagosome,ssc05134:Legionellosis,
**ProxUt**	**DOWN**	SEC23A	Sec23 homolog A, coat complex II component(SEC23A)	ssc04141:Protein processing in endoplasmic reticulum,
**ProxUt**	**DOWN**	ERLEC1	endoplasmic reticulum lectin 1(ERLEC1)	ssc04141:Protein processing in endoplasmic reticulum,
**ProxUt**	**DOWN**	LMAN2	lectin, mannose binding 2(LMAN2)	ssc04141:Protein processing in endoplasmic reticulum,
**ProxUt**	**DOWN**	TXNDC5	thioredoxin domain containing 5(TXNDC5)	ssc04141:Protein processing in endoplasmic reticulum, \
**ProxUt**	**DOWN**	XBP1	X-box binding protein 1(XBP1)	ssc04141:Protein processing in endoplasmic reticulum,ssc04932:Non-alcoholic fatty liver disease (NAFLD),ssc05166:HTLV-I infection,
**ProxUt**	**DOWN**	MRC2	mannose receptor C type 2(MRC2)	ssc04145:Phagosome,ssc05152:Tuberculosis,
**ProxUt**	**DOWN**	COL1A2	collagen type I alpha 2 chain(COL1A2)	ssc04151:PI3K-Akt signaling pathway,ssc04510:Focal adhesion,ssc04512:ECM-receptor interaction,ssc04611:Platelet activation,ssc04974:Protein digestion and absorption,ssc05146:Amoebiasis,
**ProxUt**	**DOWN**	COL3A1	collagen type III alpha 1 chain(COL3A1)	ssc04151:PI3K-Akt signaling pathway,ssc04510:Focal adhesion,ssc04512:ECM-receptor interaction,ssc04611:Platelet activation,ssc04974:Protein digestion and absorption,ssc05146:Amoebiasis,
**ProxUt**	**DOWN**	TCF7L2	transcription factor 7 like 2(TCF7L2)	ssc04310:Wnt signaling pathway,ssc04390:Hippo signaling pathway,ssc04520:Adherens junction,ssc04916:Melanogenesis,ssc05200:Pathways in cancer,ssc05210:Colorectal cancer,ssc05213:Endometrial cancer,ssc05215:Prostate cancer,ssc05216:Thyroid cancer,ssc05217:Basal cell carcinoma,ssc05221:Acute myeloid leukemia,ssc05412:Arrhythmogenic right ventricular cardiomyopathy (ARVC),
**ProxUt**	**DOWN**	WNT2	Wnt family member 2(WNT2)	ssc04310:Wnt signaling pathway,ssc04390:Hippo signaling pathway,ssc04550:Signaling pathways regulating pluripotency of stem cells,ssc04916:Melanogenesis,ssc05166:HTLV-I infection,ssc05200:Pathways in cancer,ssc05205:Proteoglycans in cancer,ssc05217:Basal cell carcinoma,
**ProxUt**	**DOWN**	EPHA4	EPH receptor A4(EPHA4)	ssc04360:Axon guidance,
**ProxUt**	**DOWN**	ROBO2	roundabout guidance receptor 2(ROBO2)	ssc04360:Axon guidance, \
**ProxUt**	**DOWN**	SNAI2	snail homolog 2 (Drosophila)(SNAI2)	ssc04390:Hippo signaling pathway,ssc04520:Adherens junction,
**ProxUt**	**DOWN**	SDC4	syndecan 4(SDC4)	ssc04512:ECM-receptor interaction,ssc04514:Cell adhesion molecules (CAMs),ssc05205:Proteoglycans in cancer,
**ProxUt**	**DOWN**	SELL	selectin L(SELL)	ssc04514:Cell adhesion molecules (CAMs),
**ProxUt**	**DOWN**	VCAN	versican(VCAN)	ssc04514:Cell adhesion molecules (CAMs),
**ProxUt**	**DOWN**	LOC100737681	contactin-associated protein-like 2(LOC100737681)	ssc04514:Cell adhesion molecules (CAMs), \
**ProxUt**	**DOWN**	CLDN11	claudin 11(CLDN11)	ssc04514:Cell adhesion molecules (CAMs),ssc04530:Tight junction,ssc04670:Leukocyte transendothelial migration,ssc05160:Hepatitis C,
**ProxUt**	**DOWN**	SDC2	syndecan 2(SDC2)	ssc04514:Cell adhesion molecules (CAMs),ssc05144:Malaria,ssc05205:Proteoglycans in cancer,
**ProxUt**	**DOWN**	PTPN1	protein tyrosine phosphatase, non-receptor type 1(PTPN1)	ssc04520:Adherens junction,ssc04910:Insulin signaling pathway,ssc04931:Insulin resistance,
**ProxUt**	**DOWN**	GJA1	gap junction protein alpha 1(GJA1)	ssc04540:Gap junction,ssc05412:Arrhythmogenic right ventricular cardiomyopathy (ARVC),
**ProxUt**	**DOWN**	PLAUR	plasminogen activator, urokinase receptor(PLAUR)	ssc04610:Complement and coagulation cascades,ssc05205:Proteoglycans in cancer,
**ProxUt**	**DOWN**	ENPEP	glutamyl aminopeptidase(ENPEP)	ssc04614:Renin-angiotensin system,
**ProxUt**	**DOWN**	MARCKS	myristoylated alanine rich protein kinase C substrate(MARCKS)	ssc04666:Fc gamma R-mediated phagocytosis,ssc05206:MicroRNAs in cancer, \
**ProxUt**	**DOWN**	NSF	N-ethylmaleimide sensitive factor, vesicle fusing ATPase(NSF)	ssc04721:Synaptic vesicle cycle,ssc04727:GABAergic synapse,ssc04962:Vasopressin-regulated water reabsorption,
**ProxUt**	**DOWN**	MAGED1	MAGE family member D1(MAGED1)	ssc04722:Neurotrophin signaling pathway,
**ProxUt**	**DOWN**	SLC38A2	solute carrier family 38 member 2(SLC38A2)	ssc04724:Glutamatergic synapse,ssc04727:GABAergic synapse,ssc04974:Protein digestion and absorption,

**ProxUt**	**DOWN**	SLC12A2	solute carrier family 12 member 2(SLC12A2)	ssc04970:Salivary secretion,ssc04972:Pancreatic secretion,
**ProxUt**	**DOWN**	COL7A1	collagen type VII alpha 1 chain(COL7A1)	ssc04974:Protein digestion and absorption,
**ProxUt**	**DOWN**	LOC100512644	uncharacterized LOC100512644(LOC100512644)	ssc04974:Protein digestion and absorption, \
**ProxUt**	**DOWN**	NCEH1	neutral cholesterol ester hydrolase 1(NCEH1)	ssc04976:Bile secretion,
**ProxUt**	**DOWN**	STEAP2	STEAP2 metalloreductase(STEAP2)	ssc04978:Mineral absorption,
**ProxUt**	**DOWN**	RARA	retinoic acid receptor alpha(RARA)	ssc05200:Pathways in cancer,ssc05202:Transcriptional misregulation in cancer,ssc05221:Acute myeloid leukemia,
**ProxUt**	**DOWN**	EPAS1	endothelial PAS domain protein 1(EPAS1)	ssc05200:Pathways in cancer,ssc05211:Renal cell carcinoma,
**ProxUt**	**DOWN**	LUM	lumican(LUM)	ssc05205:Proteoglycans in cancer,
**UTJ**	**UP**	ALDH2	aldehyde dehydrogenase 2 family (mitochondrial)(ALDH2)	ssc00010:Glycolysis / Gluconeogenesis,ssc00053:Ascorbate and aldarate metabolism,ssc00071:Fatty acid degradation,ssc00280:Valine, leucine and isoleucine degradation,ssc00310:Lysine degradation,ssc00330:Arginine and proline metabolism,ssc00340:Histidine metabolism,ssc00380:Tryptophan metabolism,ssc00410:beta-Alanine metabolism,ssc00561:Glycerolipid metabolism,ssc00620:Pyruvate metabolism,ssc01100:Metabolic pathways,ssc01130:Biosynthesis of antibiotics,
**UTJ**	**UP**	CHDH	choline dehydrogenase(CHDH)	ssc00260:Glycine, serine and threonine metabolism,ssc01100:Metabolic pathways,
**UTJ**	**UP**	MTR	5-methyltetrahydrofolate-homocysteine methyltransferase(MTR)	ssc00270:Cysteine and methionine metabolism,ssc00450:Selenocompound metabolism,ssc00670:One carbon pool by folate,ssc01100:Metabolic pathways,ssc01230:Biosynthesis of amino acids,
**UTJ**	**UP**	RENBP	renin binding protein(RENBP)	ssc00520:Amino sugar and nucleotide sugar metabolism,
**UTJ**	**UP**	UAP1	UDP-N-acetylglucosamine pyrophosphorylase 1(UAP1)	ssc00520:Amino sugar and nucleotide sugar metabolism,ssc01100:Metabolic pathways,ssc01130:Biosynthesis of antibiotics,
**UTJ**	**UP**	CHKA	choline kinase alpha(CHKA)	ssc00564:Glycerophospholipid metabolism,ssc01100:Metabolic pathways,ssc05231:Choline metabolism in cancer,
**UTJ**	**UP**	RDH10	retinol dehydrogenase 10 (all-trans)(RDH10)	ssc00830:Retinol metabolism,ssc01100:Metabolic pathways, \
**UTJ**	**UP**	CA9	carbonic anhydrase 9(CA9)	ssc00910:Nitrogen metabolism,
**UTJ**	**UP**	MAPK10	mitogen-activated protein kinase 10(MAPK10)	ssc04010:MAPK signaling pathway,ssc04012:ErbB signaling pathway,ssc04014:Ras signaling pathway,ssc04024:cAMP signaling pathway,ssc04068:FoxO signaling pathway,ssc04071:Sphingolipid signaling pathway,ssc04141:Protein processing in endoplasmic reticulum,ssc04310:Wnt signaling pathway,ssc04380:Osteoclast differentiation,ssc04510:Focal adhesion,ssc04620:Toll-like receptor signaling pathway,ssc04621:NOD-like receptor signaling pathway,ssc04622:RIG-I-like receptor signaling pathway,ssc04664:Fc epsilon RI signaling pathway,ssc04668:TNF signaling pathway,ssc04722:Neurotrophin signaling pathway,ssc04723:Retrograde endocannabinoid signaling,ssc04728:Dopaminergic synapse,ssc04750:Inflammatory mediator regulation of TRP channels,ssc04910:Insulin signaling pathway,ssc04912:GnRH signaling pathway,ssc04914:Progesterone-mediated oocyte maturation,ssc04917:Prolactin signaling pathway,ssc04920:Adipocytokine signaling pathway,ssc04930:Type II diabetes mellitus,ssc04931:Insulin resistance,ssc04932:Non-alcoholic fatty liver disease (NAFLD),ssc05132:Salmonella infection,ssc05133:Pertussis,ssc05142:Chagas disease (American trypanosomiasis),ssc05145:Toxoplasmosis,ssc05152:Tuberculosis,ssc05160:Hepatitis C,ssc05161:Hepatitis B,ssc05164:Influenza A,ssc05168:Herpes simplex infection,ssc05169:Epstein-Barr virus infection,ssc05200:Pathways in cancer,ssc05210:Colorectal cancer,ssc05212:Pancreatic cancer,ssc05231:Choline metabolism in cancer,
**UTJ**	**UP**	CACNA1D	calcium voltage-gated channel subunit alpha1 D(CACNA1D)	ssc04010:MAPK signaling pathway,ssc04020:Calcium signaling pathway,ssc04022:cGMP-PKG signaling pathway,ssc04024:cAMP signaling pathway,ssc04260:Cardiac muscle contraction,ssc04261:Adrenergic signaling in cardiomyocytes,ssc04270:Vascular smooth muscle contraction,ssc04713:Circadian entrainment,ssc04723:Retrograde endocannabinoid signaling,ssc04724:Glutamatergic synapse,ssc04725:Cholinergic synapse,ssc04726:Serotonergic synapse,ssc04727:GABAergic synapse,ssc04728:Dopaminergic synapse,ssc04911:Insulin secretion,ssc04912:GnRH signaling pathway,ssc04921:Oxytocin signaling pathway,ssc04924:Renin secretion,ssc04925:Aldosterone synthesis and secretion,ssc04930:Type II diabetes mellitus,ssc04973:Carbohydrate digestion and absorption,ssc05010:Alzheimer's disease,ssc05031:Amphetamine addiction,ssc05410:Hypertrophic cardiomyopathy (HCM),ssc05412:Arrhythmogenic right ventricular cardiomyopathy (ARVC),ssc05414:Dilated cardiomyopathy, \
**UTJ**	**UP**	ARRB1	arrestin beta 1(ARRB1)	ssc04010:MAPK signaling pathway,ssc04062:Chemokine signaling pathway,ssc04144:Endocytosis,ssc05032:Morphine addiction,
**UTJ**	**UP**	EP300	E1A binding protein p300(EP300)	ssc04024:cAMP signaling pathway,ssc04066:HIF-1 signaling pathway,ssc04068:FoxO signaling pathway,ssc04110:Cell cycle,ssc04310:Wnt signaling pathway,ssc04330:Notch signaling pathway,ssc04350:TGF-beta signaling pathway,ssc04520:Adherens junction,ssc04630:Jak-STAT signaling pathway,ssc04720:Long-term potentiation,ssc04916:Melanogenesis,ssc04919:Thyroid hormone signaling pathway,ssc04922:Glucagon signaling pathway,ssc05016:Huntington's disease,ssc05152:Tuberculosis,ssc05161:Hepatitis B,ssc05164:Influenza A,ssc05166:HTLV-I infection,ssc05168:Herpes simplex infection,ssc05200:Pathways in cancer,ssc05203:Viral carcinogenesis,ssc05206:MicroRNAs in cancer,ssc05211:Renal cell carcinoma,ssc05215:Prostate cancer,
**UTJ**	**UP**	CD27	CD27 molecule(CD27)	ssc04060:Cytokine-cytokine receptor interaction,
**UTJ**	**UP**	JAK2	Janus kinase 2(JAK2)	ssc04062:Chemokine signaling pathway,ssc04151:PI3K-Akt signaling pathway,ssc04550:Signaling pathways regulating pluripotency of stem cells,ssc04630:Jak-STAT signaling pathway,ssc04725:Cholinergic synapse,ssc04917:Prolactin signaling pathway,ssc04920:Adipocytokine signaling pathway,ssc05140:Leishmaniasis,ssc05145:Toxoplasmosis,ssc05152:Tuberculosis,ssc05162:Measles,ssc05164:Influenza A,ssc05168:Herpes simplex infection,
**UTJ**	**UP**	ELMO1	engulfment and cell motility 1(ELMO1)	ssc04062:Chemokine signaling pathway,ssc05100:Bacterial invasion of epithelial cells,
**UTJ**	**UP**	CDC14A	cell division cycle 14A(CDC14A)	ssc04110:Cell cycle,
**UTJ**	**UP**	EPS15	epidermal growth factor receptor pathway substrate 15(EPS15)	ssc04144:Endocytosis,
**UTJ**	**UP**	RAB11FIP1	RAB11 family interacting protein 1(RAB11FIP1)	ssc04144:Endocytosis, \
**UTJ**	**UP**	C1QC	complement C1q C chain(C1QC)	ssc04610:Complement and coagulation cascades,ssc05020:Prion diseases,ssc05133:Pertussis,ssc05142:Chagas disease (American trypanosomiasis),ssc05150:Staphylococcus aureus infection,ssc05322:Systemic lupus erythematosus,
**UTJ**	**UP**	CAMK1D	calcium/calmodulin dependent protein kinase ID(CAMK1D)	ssc04921:Oxytocin signaling pathway,ssc04925:Aldosterone synthesis and secretion,
**UTJ**	**UP**	HPGD	hydroxyprostaglandin dehydrogenase 15-(NAD)(HPGD)	ssc05202:Transcriptional misregulation in cancer, \
**UTJ**	**DOWN**	PGK1	phosphoglycerate kinase 1(PGK1)	ssc00010:Glycolysis / Gluconeogenesis,ssc01100:Metabolic pathways,ssc01130:Biosynthesis of antibiotics,ssc01200:Carbon metabolism,ssc01230:Biosynthesis of amino acids,
**UTJ**	**DOWN**	GAPDH	glyceraldehyde-3-phosphate dehydrogenase(GAPDH)	ssc00010:Glycolysis / Gluconeogenesis,ssc01100:Metabolic pathways,ssc01130:Biosynthesis of antibiotics,ssc01200:Carbon metabolism,ssc01230:Biosynthesis of amino acids,ssc04066:HIF-1 signaling pathway,ssc05010:Alzheimer's disease,
**UTJ**	**DOWN**	B4GALT1	beta-1,4-galactosyltransferase 1(B4GALT1)	ssc00052:Galactose metabolism,ssc00510:N-Glycan biosynthesis,ssc00514:Other types of O-glycan biosynthesis,ssc00533:Glycosaminoglycan biosynthesis - keratan sulfate,ssc00601:Glycosphingolipid biosynthesis - lacto and neolacto series,ssc01100:Metabolic pathways,
**UTJ**	**DOWN**	HMGCS1	3-hydroxy-3-methylglutaryl-CoA synthase 1(HMGCS1)	ssc00072:Synthesis and degradation of ketone bodies,ssc00280:Valine, leucine and isoleucine degradation,ssc00650:Butanoate metabolism,ssc00900:Terpenoid backbone biosynthesis,ssc01100:Metabolic pathways,ssc01130:Biosynthesis of antibiotics,
**UTJ**	**DOWN**	CYP51	cytochrome P450, family 51, subfamily A, polypeptide 1(CYP51)	ssc00100:Steroid biosynthesis,ssc01100:Metabolic pathways,ssc01130:Biosynthesis of antibiotics,

**UTJ**	**DOWN**	SQLE	squalene epoxidase(SQLE)	ssc00100:Steroid biosynthesis,ssc01100:Metabolic pathways,ssc01130:Biosynthesis of antibiotics,
**UTJ**	**DOWN**	NPR2	natriuretic peptide receptor B/guanylate cyclase B (atrionatriuretic peptide receptor B)(NPR2)	ssc00230:Purine metabolism,ssc04022:cGMP-PKG signaling pathway,ssc04270:Vascular smooth muscle contraction,ssc04921:Oxytocin signaling pathway,
**UTJ**	**DOWN**	ALDH18A1	aldehyde dehydrogenase 18 family member A1(ALDH18A1)	ssc00330:Arginine and proline metabolism,ssc01100:Metabolic pathways,ssc01230:Biosynthesis of amino acids,
**UTJ**	**DOWN**	REXO2	RNA exonuclease 2(REXO2)	ssc03008:Ribosome biogenesis in eukaryotes,\
**UTJ**	**DOWN**	SEC61G	Sec61 translocon gamma subunit(SEC61G)	ssc03060:Protein export,ssc04141:Protein processing in endoplasmic reticulum,ssc04145:Phagosome,
**UTJ**	**DOWN**	CALM2	calmodulin 2(CALM2)	ssc04014:Ras signaling pathway,ssc04015:Rap1 signaling pathway,ssc04020:Calcium signaling pathway,ssc04022:cGMP-PKG signaling pathway,ssc04024:cAMP signaling pathway,ssc04070:Phosphatidylinositol signaling system,ssc04114:Oocyte meiosis,ssc04261:Adrenergic signaling in cardiomyocytes,ssc04270:Vascular smooth muscle contraction,ssc04713:Circadian entrainment,ssc04720:Long-term potentiation,ssc04722:Neurotrophin signaling pathway,ssc04728:Dopaminergic synapse,ssc04740:Olfactory transduction,ssc04744:Phototransduction,ssc04750:Inflammatory mediator regulation of TRP channels,ssc04910:Insulin signaling pathway,ssc04912:GnRH signaling pathway,ssc04915:Estrogen signaling pathway,ssc04916:Melanogenesis,ssc04921:Oxytocin signaling pathway,ssc04922:Glucagon signaling pathway,ssc04924:Renin secretion,ssc04925:Aldosterone synthesis and secretion,ssc04970:Salivary secretion,ssc04971:Gastric acid secretion,ssc05010:Alzheimer's disease,ssc05031:Amphetamine addiction,ssc05034:Alcoholism,ssc05133:Pertussis,ssc05152:Tuberculosis,ssc05214:Glioma,
**UTJ**	**DOWN**	GNG2	G protein subunit gamma 2(GNG2)	ssc04014:Ras signaling pathway,ssc04062:Chemokine signaling pathway,ssc04151:PI3K-Akt signaling pathway,ssc04713:Circadian entrainment,ssc04723:Retrograde endocannabinoid signaling,ssc04724:Glutamatergic synapse,ssc04725:Cholinergic synapse,ssc04726:Serotonergic synapse,ssc04727:GABAergic synapse,ssc04728:Dopaminergic synapse,ssc05032:Morphine addiction,ssc05034:Alcoholism,ssc05200:Pathways in cancer,
**UTJ**	**DOWN**	CREB3L2	cAMP responsive element binding protein 3 like 2(CREB3L2)	ssc04022:cGMP-PKG signaling pathway,ssc04024:cAMP signaling pathway,ssc04151:PI3K-Akt signaling pathway,ssc04152:AMPK signaling pathway,ssc04261:Adrenergic signaling in cardiomyocytes,ssc04668:TNF signaling pathway,ssc04725:Cholinergic synapse,ssc04728:Dopaminergic synapse,ssc04911:Insulin secretion,ssc04915:Estrogen signaling pathway,ssc04916:Melanogenesis,ssc04918:Thyroid hormone synthesis,ssc04922:Glucagon signaling pathway,ssc04925:Aldosterone synthesis and secretion,ssc04931:Insulin resistance,ssc04962:Vasopressin-regulated water reabsorption,ssc05016:Huntington's disease,ssc05030:Cocaine addiction,ssc05031:Amphetamine addiction,ssc05034:Alcoholism,ssc05161:Hepatitis B,ssc05203:Viral carcinogenesis,ssc05215:Prostate cancer,
**UTJ**	**DOWN**	ACKR3	atypical chemokine receptor 3(ACKR3)	ssc04060:Cytokine-cytokine receptor interaction,
**UTJ**	**DOWN**	SEC23A	Sec23 homolog A, coat complex II component(SEC23A)	ssc04141:Protein processing in endoplasmic reticulum,
**UTJ**	**DOWN**	CD63	CD63 molecule(CD63)	ssc04142:Lysosome,ssc05205:Proteoglycans in cancer,
**UTJ**	**DOWN**	COL1A2	collagen type I alpha 2 chain(COL1A2)	ssc04151:PI3K-Akt signaling pathway,ssc04510:Focal adhesion,ssc04512:ECM-receptor interaction,ssc04611:Platelet activation,ssc04974:Protein digestion and absorption,ssc05146:Amoebiasis,
**UTJ**	**DOWN**	LOC100524585	ephrin type-B receptor 1(LOC100524585)	ssc04360:Axon guidance,\
**UTJ**	**DOWN**	CD276	CD276 molecule(CD276)	ssc04514:Cell adhesion molecules (CAMs),
**UTJ**	**DOWN**	VCAN	versican(VCAN)	ssc04514:Cell adhesion molecules (CAMs),
**UTJ**	**DOWN**	PLAUR	plasminogen activator, urokinase receptor(PLAUR)	ssc04610:Complement and coagulation cascades,ssc05205:Proteoglycans in cancer,
**UTJ**	**DOWN**	MAGED1	MAGE family member D1(MAGED1)	ssc04722:Neurotrophin signaling pathway,
**UTJ**	**DOWN**	EEF1G	eukaryotic translation elongation factor 1 gamma(EEF1G)	ssc05134:Legionellosis,

Table 6. List of pathways classified according to biological processes (**DAVID database,** P< 0.05) of differentially-expressed genes (DEGs, ID and name), solely related to copulation, in pig uterine mucosae (endocervix (Cvx), endometrium (distal: D-Endom or proximal: P-Endom) and utero-tubal junction (UTJ)), up- or down-regulated.

Tissue	Regulation	ID	Gene Name	GOTERM_BP_DIRECT
Cvx	UP	NCOR2	nuclear receptor corepressor 2(NCOR2)	GO:0000122~negative regulation of transcription from RNA polymerase II promoter,
Cvx	UP	TSHZ2	teashirt zinc finger homeobox 2(TSHZ2)	GO:0000122~negative regulation of transcription from RNA polymerase II promoter,GO:0006351~transcription, DNA-templated,GO:0006357~regulation of transcription from RNA polymerase II promoter,GO:0007275~multicellular organism development,GO:0010468~regulation of gene expression,
Cvx	UP	FUNDC2	FUN14 domain containing 2(FUNDC2)	GO:0000422~mitophagy,
Cvx	UP	FAM107A	family with sequence similarity 107 member A(FAM107A)	GO:0001558~regulation of cell growth,
Cvx	UP	PYGO1	pygopus family PHD finger 1(PYGO1)	GO:0001822~kidney development,GO:0002244~hematopoietic progenitor cell differentiation,GO:0007289~spermatid nucleus differentiation,GO:0009791~post-embryonic development,GO:0016055~Wnt signaling pathway,GO:0034504~protein localization to nucleus,GO:0045944~positive regulation of transcription from RNA polymerase II promoter,
Cvx	UP	TMEM141	transmembrane protein 141(TMEM141)	GO:0003341~cilium movement,GO:0036158~outer dynein arm assembly,
Cvx	UP	THBS3	thrombospondin 3(THBS3)	GO:0003417~growth plate cartilage development,GO:0007155~cell adhesion,GO:0043931~ossification involved in bone maturation,GO:0060346~bone trabecula formation,
Cvx	UP	FMO1	flavin containing monooxygenase 1(FMO1)	GO:0006082~organic acid metabolic process,GO:0009404~toxin metabolic process,GO:0017144~drug metabolic process,GO:0070995~NADPH oxidation,
Cvx	UP	LOC100520903	zinc finger protein 300-like(LOC100520903)	GO:0006355~regulation of transcription, DNA-templated,
Cvx	UP	PRKCQ	protein kinase C theta(PRKCQ)	GO:0006509~membrane protein ectodomain proteolysis,GO:0018105~peptidyl-serine phosphorylation,GO:0032212~positive regulation of telomere maintenance via telomerase,GO:0032740~positive regulation of interleukin-17 production,GO:0032753~positive regulation of interleukin-4 production,GO:0035556~intracellular signal transduction,GO:0042102~positive regulation of T cell proliferation,GO:0045086~positive regulation of interleukin-2 biosynthetic process,GO:0046627~negative regulation of insulin receptor signaling pathway,GO:0051092~positive regulation of NF-kappaB transcription factor activity,GO:0051973~positive regulation of telomerase activity,GO:0060326~cell chemotaxis,GO:0070233~negative regulation of T cell apoptotic process,GO:0090330~regulation of platelet aggregation,GO:1904355~positive regulation of telomere capping,GO:2000318~positive regulation of T-helper 17 type immune response,GO:2000570~positive regulation of T-helper 2 cell activation,
Cvx	UP	CPD	carboxypeptidase D(CPD)	GO:0006518~peptide metabolic process,GO:0016485~protein processing,
Cvx	UP	DECR2	2,4-dienoyl-CoA reductase 2, peroxisomal(DECR2)	GO:0006636~unsaturated fatty acid biosynthetic process,
Cvx	UP	PCBD1	pterin-4 alpha-carbinolamine dehydratase 1(PCBD1)	GO:0006729~tetrahydrobiopterin biosynthetic process,GO:0051289~protein homotetramerization,GO:0051291~protein heterooligomerization,
Cvx	UP	LOC100153094	glutathione S-transferase theta-1(LOC100153094)	GO:0006749~glutathione metabolic process,
Cvx	UP	GGA2	golgi associated, gamma adaptin ear containing, ARF binding protein 2(GGA2)	GO:0006886~intracellular protein transport,GO:0016192~vesicle-mediated transport,
Cvx	UP	VAMP2	vesicle associated membrane protein 2(VAMP2)	GO:0006887~exocytosis,GO:0006906~vesicle fusion,GO:0016079~synaptic vesicle exocytosis,GO:0017157~regulation of exocytosis,GO:0032869~cellular response to insulin stimulus,GO:0043001~Golgi to plasma membrane protein transport,GO:0060291~long-term synaptic potentiation,GO:0090316~positive regulation of intracellular protein transport,
Cvx	UP	AKAP9	A-kinase anchoring protein 9(AKAP9)	GO:0007165~signal transduction,
Cvx	UP	NDRG4	NDRG family member 4(NDRG4)	GO:0007165~signal transduction,GO:0007420~brain development,GO:0010642~negative regulation of platelet-derived growth factor receptor signaling pathway,GO:0014912~negative regulation of smooth muscle cell migration,GO:0048662~negative regulation of smooth muscle cell proliferation,GO:0070374~positive regulation of ERK1 and ERK2 cascade,
Cvx	UP	IGF1R	insulin like growth factor 1 receptor(IGF1R)	GO:0007169~transmembrane receptor protein tyrosine kinase signaling pathway,GO:0018108~peptidyl-tyrosine phosphorylation,GO:0043066~negative regulation of apoptotic process,GO:0046328~regulation of JNK cascade,GO:0046777~protein autophosphorylation,GO:0051389~inactivation of MAPKK activity,
Cvx	UP	MYLIP	myosin regulatory light chain interacting protein(MYLIP)	GO:0007399~nervous system development,GO:0010989~negative regulation of low-density lipoprotein particle clearance,GO:0031648~protein destabilization,GO:0032803~regulation of low-density lipoprotein particle receptor catabolic process,GO:0042632~cholesterol homeostasis,GO:0042787~protein ubiquitination involved in ubiquitin-dependent protein catabolic process,GO:0045732~positive regulation of protein catabolic process,
Cvx	UP	KCNQ4	potassium voltage-gated channel subfamily Q member 4(KCNQ4)	GO:0007605~sensory perception of sound,GO:0042472~inner ear morphogenesis,
Cvx	UP	ENPP5	ectonucleotide pyrophosphatase/phosphodiesterase 5 (putative)(ENPP5)	GO:0008152~metabolic process,
Cvx	UP	CCDC130	coiled-coil domain containing 130(CCDC130)	GO:0009615~response to virus,
Cvx	UP	DDX17	DEAD-box helicase 17(DDX17)	GO:0010501~RNA secondary structure unwinding,GO:0045893~positive regulation of transcription, DNA-templated,
Cvx	UP	NTN4	netrin 4(NTN4)	GO:0016322~neuron remodeling,GO:0060668~regulation of branching involved in salivary gland morphogenesis by extracellular matrix-epithelial cell signaling,
Cvx	UP	LOC102166095	nuclear receptor coactivator 2-like(LOC102166095)	GO:0030522~intracellular receptor signaling pathway,GO:0032870~cellular response to hormone stimulus,GO:0045944~positive regulation of transcription from RNA polymerase II promoter,
Cvx	UP	RNF149	ring finger protein 149(RNF149)	GO:0031647~regulation of protein stability,GO:0035690~cellular response to drug,GO:0043409~negative regulation of MAPK cascade,
Cvx	UP	SESN3	sestrin 3(SESN3)	GO:0032868~response to insulin,GO:0042593~glucose homeostasis,GO:0051896~regulation of protein kinase B signaling,GO:0071230~cellular response to amino acid stimulus,GO:1901031~regulation of response to reactive oxygen species,GO:1904262~negative regulation of TORC1 signaling,
Cvx	UP	HDAC9	histone deacetylase 9(HDAC9)	GO:0040029~regulation of gene expression, epigenetic,
Cvx	UP	CDKAL1	CDK5 regulatory subunit associated protein 1 like 1(CDKAL1)	GO:1990145~maintenance of translational fidelity,
Cvx	DOWN	PAPSS2	3'-phosphoadenosine 5'-phosphosulfate synthase 2(PAPSS2)	GO:0000103~sulfate assimilation,GO:0007596~blood coagulation,GO:0050428~3'-phosphoadenosine 5'-phosphosulfate biosynthetic process,GO:0060348~bone development,GO:0070814~hydrogen sulfide biosynthetic process,
Cvx	DOWN	TDG	thymine-DNA glycosylase(TDG)	GO:0000122~negative regulation of transcription from RNA polymerase II promoter,GO:0006285~base-excision repair, AP site formation,GO:0006298~mismatch repair,GO:0009790~embryo development,GO:0032091~negative regulation of protein binding,GO:0035562~negative regulation of chromatin binding,GO:0040029~regulation of gene expression, epigenetic,GO:0080111~DNA demethylation,

**Cvx**	**DOWN**	THBS1	thrombospondin 1(THBS1)	GO:0000187~activation of MAPK activity,GO:0001937~negative regulation of endothelial cell proliferation,GO:0001953~negative regulation of cell-matrix adhesion,GO:0002040~sprouting angiogenesis,GO:0002544~chronic inflammatory response,GO:0002581~negative regulation of antigen processing and presentation of peptide or polysaccharide antigen via MHC class II,GO:0002605~negative regulation of dendritic cell antigen processing and presentation,GO:0006954~inflammatory response,GO:0006955~immune response,GO:0007050~cell cycle arrest,GO:0007155~cell adhesion,GO:0009749~response to glucose,GO:0010748~negative regulation of plasma membrane long-chain fatty acid transport,GO:0010751~negative regulation of nitric oxide mediated signal transduction,GO:0010754~negative regulation of cGMP-mediated signaling,GO:0010757~negative regulation of plasminogen activation,GO:0010763~positive regulation of fibroblast migration,GO:0016477~cell migration,GO:0016525~negative regulation of angiogenesis,GO:0018149~peptide cross-linking,GO:0030511~positive regulation of transforming growth factor beta receptor signaling pathway,GO:0030823~regulation of cGMP metabolic process,GO:0032026~response to magnesium ion,GO:0032695~negative regulation of interleukin-12 production,GO:0040037~negative regulation of fibroblast growth factor receptor signaling pathway,GO:0042493~response to drug,GO:0042535~positive regulation of tumor necrosis factor biosynthetic process,GO:0043032~positive regulation of macrophage activation,GO:0043154~negative regulation of cysteine-type endopeptidase activity involved in apoptotic process,GO:0043536~positive regulation of blood vessel endothelial cell migration,GO:0043537~negative regulation of blood vessel endothelial cell migration,GO:0043652~engulfment of apoptotic cell,GO:0045727~positive regulation of translation,GO:0045766~positive regulation of angiogenesis,GO:0050921~positive regulation of chemotaxis,GO:0051592~response to calcium ion,GO:0051897~positive regulation of protein kinase B signaling,GO:0051918~negative regulation of fibrinolysis,GO:1902043~positive regulation of extrinsic apoptotic signaling pathway via death domain receptors,GO:2000353~positive regulation of endothelial cell apoptotic process,GO:2000379~positive regulation of reactive oxygen species metabolic process,GO:2001027~negative regulation of endothelial cell chemotaxis,
**Cvx**	**DOWN**	VCAN	versican(VCAN)	GO:0001501~skeletal system development,GO:0001649~osteoblast differentiation,GO:0007155~cell adhesion,GO:0007417~central nervous system development,
**Cvx**	**DOWN**	ENPEP	glutamyl aminopeptidase(ENPEP)	GO:0001525~angiogenesis,GO:0002003~angiotensin maturation,GO:0003081~regulation of systemic arterial blood pressure by renin-angiotensin,GO:0006508~proteolysis,GO:0008217~regulation of blood pressure,GO:0008283~cell proliferation,GO:0016477~cell migration,GO:0032835~glomerulus development,GO:0043171~peptide catabolic process,
**Cvx**	**DOWN**	SRPX2	sushi repeat containing protein, X-linked 2(SRPX2)	GO:0001525~angiogenesis,GO:0016337~single organismal cell-cell adhesion,GO:0042325~regulation of phosphorylation,GO:0048870~cell motility,GO:0051965~positive regulation of synapse assembly,GO:0071625~vocalization behavior,GO:0090050~positive regulation of cell migration involved in sprouting angiogenesis,
**Cvx**	**DOWN**	GJA1	gap junction protein alpha 1(GJA1)	GO:0001649~osteoblast differentiation,GO:0001701~in utero embryonic development,GO:0001764~neuron migration,GO:0001947~heart looping,GO:0002070~epithelial cell maturation,GO:0002088~lens development in camera-type eye,GO:0002931~response to ischemia,GO:0003294~atrial ventricular junction remodeling,GO:0007154~cell communication,GO:0007267~cell-cell signaling,GO:0007507~heart development,GO:0007512~adult heart development,GO:0009611~response to wounding,GO:0010628~positive regulation of gene expression,GO:0010629~negative regulation of gene expression,GO:0010643~cell communication by chemical coupling,GO:0030308~negative regulation of cell growth,GO:0030500~regulation of bone mineralization,GO:0036120~cellular response to platelet-derived growth factor stimulus,GO:0042733~embryonic digit morphogenesis,GO:0043123~positive regulation of I-kappaB kinase/NF-kappaB signaling,GO:0043403~skeletal muscle tissue regeneration,GO:0045216~cell-cell junction organization,GO:0045669~positive regulation of osteoblast differentiation,GO:0045844~positive regulation of striated muscle tissue development,GO:0046850~regulation of bone remodeling,GO:0048514~blood vessel morphogenesis,GO:0055085~transmembrane transport,GO:0060156~milk ejection,GO:0060307~regulation of ventricular cardiac muscle cell membrane repolarization,GO:0060371~regulation of atrial cardiac muscle cell membrane depolarization,GO:0060373~regulation of ventricular cardiac muscle cell membrane depolarization,GO:0061337~cardiac conduction,GO:0071673~positive regulation of smooth muscle cell chemotaxis,
**Cvx**	**DOWN**	PLAT	plasminogen activator, tissue type(PLAT)	GO:0001666~response to hypoxia,GO:0014909~smooth muscle cell migration,GO:0031639~plasminogen activation,GO:0045861~negative regulation of proteolysis,GO:0048008~platelet-derived growth factor receptor signaling pathway,
**Cvx**	**DOWN**	PLOD2	procollagen-lysine,2-oxoglutarate 5-dioxygenase 2(PLOD2)	GO:0001666~response to hypoxia,GO:0098779~mitophagy in response to mitochondrial depolarization,
**Cvx**	**DOWN**	MAN2A1	mannosidase alpha class 2A member 1(MAN2A1)	GO:0001701~in utero embryonic development,GO:0001889~liver development,GO:0006013~mannose metabolic process,GO:0006491~N-glycan processing,GO:0006517~protein deglycosylation,GO:0007005~mitochondrion organization,GO:0007033~vacuole organization,GO:0007585~respiratory gaseous exchange,GO:0048286~lung alveolus development,GO:0050769~positive regulation of neurogenesis,GO:0060042~retina morphogenesis in camera-type eye,
**Cvx**	**DOWN**	LOC100739844	semaphorin-3A-like(LOC100739844)	GO:0001755~neural crest cell migration,GO:0021772~olfactory bulb development,GO:0048843~negative regulation of axon extension involved in axon guidance,GO:0050919~negative chemotaxis,GO:0071526~semaphorin-plexin signaling pathway,GO:2001224~positive regulation of neuron migration,
**Cvx**	**DOWN**	S1PR3	sphingosine-1-phosphate receptor 3(S1PR3)	GO:0001816~cytokine production,GO:0006954~inflammatory response,GO:0007193~adenylate cyclase-inhibiting G-protein coupled receptor signaling pathway,GO:0007219~Notch signaling pathway,GO:0032651~regulation of interleukin-1 beta production,GO:1903141~negative regulation of establishment of endothelial barrier,
**Cvx**	**DOWN**	AGPAT2	1-acylglycerol-3-phosphate O-acyltransferase 2(AGPAT2)	GO:0001819~positive regulation of cytokine production,GO:0006654~phosphatidic acid biosynthetic process,GO:0008654~phospholipid biosynthetic process,GO:0046474~glycerophospholipid biosynthetic process,
**Cvx**	**DOWN**	SEPHS2	selenophosphate synthetase 2(SEPHS2)	GO:0001887~selenium compound metabolic process,GO:0016260~selenocysteine biosynthetic process,GO:0016310~phosphorylation,
**Cvx**	**DOWN**	RIPK3	receptor interacting serine/threonine kinase 3(RIPK3)	GO:0001914~regulation of T cell mediated cytotoxicity,GO:0007249~I-kappaB kinase/NF-kappaB signaling,GO:0010922~positive regulation of phosphatase activity,GO:0032147~activation of protein kinase activity,GO:0032649~regulation of interferon-gamma production,GO:0033077~T cell differentiation in thymus,GO:0043029~T cell homeostasis,GO:0046006~regulation of activated T cell proliferation,GO:0046777~protein autophosphorylation,GO:0048535~lymph node development,GO:0048536~spleen development,GO:0048538~thymus development,GO:0051092~positive regulation of NF-kappaB transcription factor activity,GO:0051260~protein homooligomerization,GO:0051291~protein heterooligomerization,GO:0051351~positive regulation of ligase activity,GO:0051353~positive regulation of oxidoreductase activity,GO:0060545~positive regulation of necroptotic process,GO:0070235~regulation of activation-induced cell death of T cells,GO:0070266~necroptotic process,GO:0090312~positive regulation of protein deacetylation,GO:1990000~amyloid fibril formation,GO:2000379~positive regulation of reactive oxygen species metabolic process,GO:2000452~regulation of CD8-positive, alpha-beta cytotoxic T cell extravasation,GO:2001244~positive regulation of intrinsic apoptotic signaling pathway,
**Cvx**	**DOWN**	PLAUR	plasminogen activator, urokinase receptor(PLAUR)	GO:0001934~positive regulation of protein phosphorylation,GO:0030162~regulation of proteolysis,GO:0038195~urokinase plasminogen activator signaling pathway,GO:0043388~positive regulation of DNA binding,GO:0045742~positive regulation of epidermal growth factor receptor signaling pathway,GO:0090200~positive regulation of release of cytochrome c from mitochondria,GO:2001243~negative regulation of intrinsic apoptotic signaling pathway,GO:2001268~negative regulation of cysteine-type endopeptidase activity involved in apoptotic signaling pathway,
**Cvx**	**DOWN**	B4GALT1	beta-1,4-galactosyltransferase 1(B4GALT1)	GO:0002064~epithelial cell development,GO:0002526~acute inflammatory response,GO:0005989~lactose biosynthetic process,GO:0006012~galactose metabolic process,GO:0006487~protein N-linked glycosylation,GO:0007155~cell adhesion,GO:0007339~binding of sperm to zona pellucida,GO:0007341~penetration of zona pellucida,GO:0008285~negative regulation of cell proliferation,GO:0030198~extracellular matrix organization,GO:0030879~mammary gland development,GO:0045136~development of secondary sexual characteristics,GO:0048754~branching morphogenesis of an epithelial tube,GO:0050900~leukocyte migration,GO:0051270~regulation of cellular component movement,GO:0060046~regulation of acrosome reaction,GO:0060054~positive regulation of epithelial cell proliferation involved in wound healing,GO:0060055~angiogenesis involved in wound healing,GO:0060058~positive regulation of apoptotic process involved in mammary gland involution,
**Cvx**	**DOWN**	FGFBP1	fibroblast growth factor binding protein 1(FGFBP1)	GO:0002230~positive regulation of defense response to virus by host,GO:0008284~positive regulation of cell proliferation,GO:0045743~positive regulation of fibroblast growth factor receptor signaling pathway,GO:0098779~mitophagy in response to mitochondrial depolarization,GO:0098792~xenophagy,
**Cvx**	**DOWN**	ANXA5	annexin A5(ANXA5)	GO:0002230~positive regulation of defense response to virus by host,GO:0010033~response to organic substance,GO:0050819~negative regulation of coagulation,GO:0098779~mitophagy in response to mitochondrial depolarization,GO:0098792~xenophagy,
**Cvx**	**DOWN**	GCNT3	glucosaminyl (N-acetyl) transferase 3, mucin type(GCNT3)	GO:0002426~immunoglobulin production in mucosal tissue,GO:0048729~tissue morphogenesis,GO:0050892~intestinal absorption,GO:0060993~kidney morphogenesis,
**Cvx**	**DOWN**	CXCL10	C-X-C motif chemokine ligand 10(CXCL10)	GO:0002690~positive regulation of leukocyte chemotaxis,GO:0006954~inflammatory response,GO:0006955~immune response,GO:0007186~G-protein coupled receptor signaling pathway,GO:0008284~positive regulation of cell proliferation,GO:0010818~T cell chemotaxis,GO:0010819~regulation of T cell chemotaxis,GO:0030816~positive regulation of cAMP metabolic process,GO:0032496~response to lipopolysaccharide,GO:0042118~endothelial cell activation,GO:0042127~regulation of cell proliferation,GO:0043950~positive regulation of cAMP-mediated signaling,GO:0045662~negative regulation of myoblast differentiation,GO:0051281~positive regulation of release of sequestered calcium ion into cytosol,GO:0051607~defense response to virus,GO:0060326~cell chemotaxis,GO:0070098~chemokine-mediated signaling

				pathway,GO:0071222~cellular response to lipopolysaccharide,GO:0090026~positive regulation of monocyte chemotaxis,GO:1901509~regulation of endothelial tube morphogenesis,GO:1901740~negative regulation of myoblast fusion,GO:2000406~positive regulation of T cell migration,
**Cvx**	**DOWN**	NPY2R	neuropeptide Y receptor Y2(NPY2R)	GO:0003151~outflow tract morphogenesis,GO:0003214~cardiac left ventricle morphogenesis,GO:0007166~cell surface receptor signaling pathway,GO:0007193~adenylate cyclase-inhibiting G-protein coupled receptor signaling pathway,GO:0007218~neuropeptide signaling pathway,GO:0007268~chemical synaptic transmission,GO:0007631~feeding behavior,
**Cvx**	**DOWN**	IL6ST	interleukin 6 signal transducer(IL6ST)	GO:0005977~glycogen metabolic process,GO:0008593~regulation of Notch signaling pathway,GO:0042102~positive regulation of T cell proliferation,GO:0042511~positive regulation of tyrosine phosphorylation of Stat1 protein,GO:0042517~positive regulation of tyrosine phosphorylation of Stat3 protein,GO:0045669~positive regulation of osteoblast differentiation,GO:0048711~positive regulation of astrocyte differentiation,GO:0070106~interleukin-27-mediated signaling pathway,
**Cvx**	**DOWN**	PGD	phosphogluconate dehydrogenase(PGD)	GO:0006098~pentose-phosphate shunt,GO:0009051~pentose-phosphate shunt, oxidative branch,GO:0019322~pentose biosynthetic process,GO:0019521~D-gluconate metabolic process,GO:0055114~oxidation-reduction process,
**Cvx**	**DOWN**	NPR2	natriuretic peptide receptor B/guanylate cyclase B (atrionatriuretic peptide receptor B)(NPR2)	GO:0006182~cGMP biosynthetic process,GO:0007168~receptor guanylyl cyclase signaling pathway,GO:0035556~intracellular signal transduction,GO:0044702~single organism reproductive process,GO:0051447~negative regulation of meiotic cell cycle,GO:0060348~bone development,GO:0097011~cellular response to granulocyte macrophage colony-stimulating factor stimulus,GO:1900194~negative regulation of oocyte maturation,
**Cvx**	**DOWN**	CMPK2	cytidine/uridine monophosphate kinase 2(CMPK2)	GO:0006227~dUDP biosynthetic process,GO:0006233~dTDP biosynthetic process,GO:0006235~dTTP biosynthetic process,GO:0071222~cellular response to lipopolysaccharide,
**Cvx**	**DOWN**	FAM111A	family with sequence similarity 111 member A(FAM111A)	GO:0006260~DNA replication,GO:0045071~negative regulation of viral genome replication,
**Cvx**	**DOWN**	SLC25A17	solute carrier family 25 member 17(SLC25A17)	GO:0006412~translation,GO:0006635~fatty acid beta-oxidation,GO:0015908~fatty acid transport,
**Cvx**	**DOWN**	GARS	glycyl-tRNA synthetase(GARS)	GO:0006426~glycyl-tRNA aminoacylation,GO:0015966~diadenosine tetraphosphate biosynthetic process,
**Cvx**	**DOWN**	UBA7	ubiquitin like modifier activating enzyme 7(UBA7)	GO:0006464~cellular protein modification process,GO:0019941~modification-dependent protein catabolic process,GO:0032020~ISG15-protein conjugation,
**Cvx**	**DOWN**	UBASH3B	ubiquitin associated and SH3 domain containing B(UBASH3B)	GO:0006469~negative regulation of protein kinase activity,GO:0009968~negative regulation of signal transduction,GO:0045671~negative regulation of osteoclast differentiation,GO:0045779~negative regulation of bone resorption,GO:0051279~regulation of release of sequestered calcium ion into cytosol,GO:0090331~negative regulation of platelet aggregation,
**Cvx**	**DOWN**	NPC1	NPC intracellular cholesterol transporter 1(NPC1)	GO:0006486~protein glycosylation,GO:0006897~endocytosis,GO:0006914~autophagy,GO:0007041~lysosomal transport,GO:0007628~adult walking behavior,GO:0008203~cholesterol metabolic process,GO:0008206~bile acid metabolic process,GO:0016242~negative regulation of macroautophagy,GO:0030301~cholesterol transport,GO:0031579~membrane raft organization,GO:0033344~cholesterol efflux,GO:0042493~response to drug,GO:0042632~cholesterol homeostasis,GO:0046718~viral entry into host cell,GO:0071383~cellular response to steroid hormone stimulus,GO:0071404~cellular response to low-density lipoprotein particle stimulus,GO:0090150~establishment of protein localization to membrane,GO:2000189~positive regulation of cholesterol homeostasis,
**Cvx**	**DOWN**	B3GALT2	beta-1,3-galactosyltransferase 2(B3GALT2)	GO:0006486~protein glycosylation,GO:0009312~oligosaccharide biosynthetic process,
**Cvx**	**DOWN**	CPXM1	carboxypeptidase X, M14 family member 1(CPXM1)	GO:0006508~proteolysis,
**Cvx**	**DOWN**	INSIG1	insulin induced gene 1(INSIG1)	GO:0006641~triglyceride metabolic process,GO:0006695~cholesterol biosynthetic process,GO:0010894~negative regulation of steroid biosynthetic process,GO:0032933~SREBP signaling pathway,GO:0042472~inner ear morphogenesis,GO:0042474~middle ear morphogenesis,GO:0045599~negative regulation of fat cell differentiation,GO:0045717~negative regulation of fatty acid biosynthetic process,GO:0060021~palate development,GO:0060363~cranial suture morphogenesis,GO:1901303~negative regulation of cargo loading into COPII-coated vesicle,
**Cvx**	**DOWN**	SGPP2	sphingosine-1-phosphate phosphatase 2(SGPP2)	GO:0006670~sphingosine metabolic process,
**Cvx**	**DOWN**	HMGCR	3-hydroxy-3-methylglutaryl-CoA reductase(HMGCR)	GO:0006695~cholesterol biosynthetic process,GO:0008299~isoprenoid biosynthetic process,GO:0008542~visual learning,GO:0015936~coenzyme A metabolic process,GO:0016126~sterol biosynthetic process,GO:0043407~negative regulation of MAP kinase activity,GO:0051262~protein tetramerization,GO:0055114~oxidation-reduction process,
**Cvx**	**DOWN**	SNX31	sorting nexin 31(SNX31)	GO:0006886~intracellular protein transport,GO:1990126~retrograde transport, endosome to plasma membrane,
**Cvx**	**DOWN**	ATP6V0C	ATPase H+ transporting V0 subunit c(ATP6V0C)	GO:0007042~lysosomal lumen acidification,GO:0015991~ATP hydrolysis coupled proton transport,GO:0030177~positive regulation of Wnt signaling pathway,
**Cvx**	**DOWN**	CD47	CD47 molecule(CD47)	GO:0007155~cell adhesion,GO:0008228~opsonization,GO:0008284~positive regulation of cell proliferation,GO:0009617~response to bacterium,GO:0050729~positive regulation of inflammatory response,GO:0050766~positive regulation of phagocytosis,GO:0050870~positive regulation of T cell activation,
**Cvx**	**DOWN**	DPT	dermatopontin(DPT)	GO:0007155~cell adhesion,GO:0008285~negative regulation of cell proliferation,GO:0030199~collagen fibril organization,
**Cvx**	**DOWN**	CDH7	cadherin 7(CDH7)	GO:0007156~homophilic cell adhesion via plasma membrane adhesion molecules,
**Cvx**	**DOWN**	LOC100154959	olfactory receptor 4K3(LOC100154959)	GO:0007186~G-protein coupled receptor signaling pathway,GO:0050907~detection of chemical stimulus involved in sensory perception,
**Cvx**	**DOWN**	RASD1	ras related dexamethasone induced 1(RASD1)	GO:0007264~small GTPase mediated signal transduction,
**Cvx**	**DOWN**	KSR1	kinase suppressor of ras 1(KSR1)	GO:0007265~Ras protein signal transduction,GO:0019933~cAMP-mediated signaling,GO:0043410~positive regulation of MAPK cascade,
**Cvx**	**DOWN**	DLGAP2	DLG associated protein 2(DLGAP2)	GO:0007270~neuron-neuron synaptic transmission,
**Cvx**	**DOWN**	ABHD2	abhydrolase domain containing 2(ABHD2)	GO:0007340~acrosome reaction,GO:0009611~response to wounding,GO:0030336~negative regulation of cell migration,GO:0032570~response to progesterone,GO:0046464~acylglycerol catabolic process,GO:0048240~sperm capacitation,
**Cvx**	**DOWN**	LOC100511841	UDP-glucuronosyltransferase 1-10(LOC100511841)	GO:0008152~metabolic process,
**Cvx**	**DOWN**	HMGCS1	3-hydroxy-3-methylglutaryl-CoA synthase 1(HMGCS1)	GO:0008299~isoprenoid biosynthetic process,
**Cvx**	**DOWN**	PALM2	paralemmin 2(PALM2)	GO:0008360~regulation of cell shape,
**Cvx**	**DOWN**	RSAD2	radical S-adenosyl methionine domain containing 2(RSAD2)	GO:0009615~response to virus,GO:0034157~positive regulation of toll-like receptor 7 signaling pathway,GO:0034165~positive regulation of toll-like receptor 9 signaling pathway,GO:0035710~CD4-positive, alpha-beta T cell activation,GO:0043367~CD4-positive, alpha-beta T cell differentiation,GO:0045071~negative regulation of viral genome replication,GO:0045087~innate immune response,GO:0050709~negative regulation of protein secretion,GO:0051607~defense response to virus,GO:2000553~positive regulation of T-helper 2 cell cytokine production,
**Cvx**	**DOWN**	SLC30A7	solute carrier family 30 member 7(SLC30A7)	GO:0010043~response to zinc ion,GO:0032119~sequestering of zinc ion,GO:0061088~regulation of sequestering of zinc ion,
**Cvx**	**DOWN**	DRAM1	DNA damage regulated autophagy modulator 1(DRAM1)	GO:0010506~regulation of autophagy,
**Cvx**	**DOWN**	MMP3	matrix metallopeptidase 3 (stromelysin 1, progelatinase)(MMP3)	GO:0010727~negative regulation of hydrogen peroxide metabolic process,GO:0032461~positive regulation of protein oligomerization,
**Cvx**	**DOWN**	XPNPEP1	X-prolyl aminopeptidase 1(XPNPEP1)	GO:0010815~bradykinin catabolic process,
**Cvx**	**DOWN**	IER3	immediate early response 3(IER3)	GO:0010941~regulation of cell death,GO:0043066~negative regulation of apoptotic process,GO:2001020~regulation of response to DNA damage stimulus,
**Cvx**	**DOWN**	IFRD1	interferon related developmental regulator 1(IFRD1)	GO:0014706~striated muscle tissue development,GO:0030517~negative regulation of axon extension,GO:0042692~muscle cell differentiation,GO:0043403~skeletal muscle tissue regeneration,GO:0048671~negative regulation of collateral sprouting,
**Cvx**	**DOWN**	MFSD2A	major facilitator superfamily domain containing 2A(MFSD2A)	GO:0015908~fatty acid transport,GO:0045056~transcytosis,GO:0060856~establishment of blood-brain barrier,GO:1990379~lipid transport across blood brain barrier,
**Cvx**	**DOWN**	PANK3	pantothenate kinase 3(PANK3)	GO:0015937~coenzyme A biosynthetic process,

Cvx	DOWN	SLA-7	MHC class I antigen 7(SLA-7)	GO:0019882~antigen processing and presentation,
Cvx	DOWN	TAPBP	TAP binding protein(TAPBP)	GO:0019885~antigen processing and presentation of endogenous peptide antigen via MHC class I,
Cvx	DOWN	MX1	myxovirus (influenza virus) resistance 1, interferon-inducible protein p78 (mouse)(MX1)	GO:0032439~endosome localization,GO:0045071~negative regulation of viral genome replication,GO:0048285~organelle fission,GO:0051607~defense response to virus,GO:0071357~cellular response to type I interferon,GO:1901253~negative regulation of intracellular transport of viral material,
Cvx	DOWN	CYP51	cytochrome P450, family 51, subfamily A, polypeptide 1(CYP51)	GO:0033488~cholesterol biosynthetic process via 24,25-dihydrolanosterol,GO:0055114~oxidation-reduction process,GO:0070988~demethylation,
Cvx	DOWN	LOC100518083	E3 ISG15--protein ligase HERC5(LOC100518083)	GO:0042787~protein ubiquitination involved in ubiquitin-dependent protein catabolic process,
Cvx	DOWN	FDPS	farnesyl diphosphate synthase(FDPS)	GO:0045337~farnesyl diphosphate biosynthetic process,
Cvx	DOWN	BRINP2	BMP/retinoic acid inducible neural specific 2(BRINP2)	GO:0045666~positive regulation of neuron differentiation,GO:0045930~negative regulation of mitotic cell cycle,GO:0071300~cellular response to retinoic acid,
Cvx	DOWN	NT5C2	5'-nucleotidase, cytosolic II(NT5C2)	GO:0046040~IMP metabolic process,GO:0046085~adenosine metabolic process,
Cvx	DOWN	MAPK6	mitogen-activated protein kinase 6(MAPK6)	GO:0060999~positive regulation of dendritic spine development,
Cvx	DOWN	LOC100523138	transmembrane protein 88-like(LOC100523138)	GO:0090090~negative regulation of canonical Wnt signaling pathway,
DistUt	UP	CDC25C	cell division cycle 25C(CDC25C)	GO:0000086~G2/M transition of mitotic cell cycle,GO:0007067~mitotic nuclear division,GO:0035335~peptidyl-tyrosine dephosphorylation,GO:0051301~cell division,
DistUt	UP	NCOR2	nuclear receptor corepressor 2(NCOR2)	GO:0000122~negative regulation of transcription from RNA polymerase II promoter,
DistUt	UP	CNOT2	CCR4-NOT transcription complex subunit 2(CNOT2)	GO:0000122~negative regulation of transcription from RNA polymerase II promoter,GO:0000288~nuclear-transcribed mRNA catabolic process, deadenylation-dependent decay,GO:0001829~trophectodermal cell differentiation,GO:0010606~positive regulation of cytoplasmic mRNA processing body assembly,GO:0017148~negative regulation of translation,GO:0033147~negative regulation of intracellular estrogen receptor signaling pathway,GO:2000036~regulation of stem cell population maintenance,
DistUt	UP	CNOT1	CCR4-NOT transcription complex subunit 1(CNOT1)	GO:0000122~negative regulation of transcription from RNA polymerase II promoter,GO:0000288~nuclear-transcribed mRNA catabolic process, deadenylation-dependent decay,GO:0010606~positive regulation of cytoplasmic mRNA processing body assembly,GO:0017148~negative regulation of translation,GO:0033147~negative regulation of intracellular estrogen receptor signaling pathway,GO:0035195~gene silencing by miRNA,GO:0048387~negative regulation of retinoic acid receptor signaling pathway,GO:0060213~positive regulation of nuclear-transcribed mRNA poly(A) tail shortening,GO:2000036~regulation of stem cell population maintenance,
DistUt	UP	RARG	retinoic acid receptor gamma(RARG)	GO:0000122~negative regulation of transcription from RNA polymerase II promoter,GO:0001843~neural tube closure,GO:0002068~glandular epithelial cell development,GO:0003406~retinal pigment epithelium development,GO:0003430~growth plate cartilage chondrocyte growth,GO:0006351~transcription, DNA-templated,GO:0008284~positive regulation of cell proliferation,GO:0008285~negative regulation of cell proliferation,GO:0009952~anterior/posterior pattern specification,GO:0031076~embryonic camera-type eye development,GO:0032331~negative regulation of chondrocyte differentiation,GO:0035116~embryonic hindlimb morphogenesis,GO:0035264~multicellular organism growth,GO:0043065~positive regulation of apoptotic process,GO:0043066~negative regulation of apoptotic process,GO:0045637~regulation of myeloid cell differentiation,GO:0045944~positive regulation of transcription from RNA polymerase II promoter,GO:0048048~embryonic eye morphogenesis,GO:0060324~face development,GO:0060534~trachea cartilage development,GO:0060740~prostate gland epithelium morphogenesis,GO:0070384~Harderian gland development,GO:0071300~cellular response to retinoic acid,
DistUt	UP	NFIX	nuclear factor I X(NFIX)	GO:0000122~negative regulation of transcription from RNA polymerase II promoter,GO:0006260~DNA replication,GO:0006351~transcription, DNA-templated,GO:0045944~positive regulation of transcription from RNA polymerase II promoter,
DistUt	UP	EHMT2	euchromatic histone lysine methyltransferase 2(EHMT2)	GO:0000122~negative regulation of transcription from RNA polymerase II promoter,GO:0006275~regulation of DNA replication,GO:0007130~synaptonemal complex assembly,GO:0007286~spermatid development,GO:0009566~fertilization,GO:0010424~DNA methylation on cytosine within a CG sequence,GO:0035265~organ growth,GO:0036166~phenotypic switching,GO:0048148~behavioral response to cocaine,GO:0048665~neuron fate specification,GO:0071314~cellular response to cocaine,GO:1902902~negative regulation of autophagosome assembly,
DistUt	UP	RREB1	ras responsive element binding protein 1(RREB1)	GO:0000122~negative regulation of transcription from RNA polymerase II promoter,GO:0006355~regulation of transcription, DNA-templated,GO:0006366~transcription from RNA polymerase II promoter,GO:0007165~signal transduction,GO:0007275~multicellular organism development,GO:0010634~positive regulation of epithelial cell migration,GO:0033601~positive regulation of mammary gland epithelial cell proliferation,GO:1900026~positive regulation of substrate adhesion-dependent cell spreading,GO:1903691~positive regulation of wound healing, spreading of epidermal cells,GO:2000394~positive regulation of lamellipodium morphogenesis,
DistUt	UP	USP2	ubiquitin specific peptidase 2(USP2)	GO:0000122~negative regulation of transcription from RNA polymerase II promoter,GO:0006511~ubiquitin-dependent protein catabolic process,GO:0016579~protein deubiquitination,GO:0032922~circadian regulation of gene expression,GO:0043153~entrainment of circadian clock by photoperiod,GO:0045475~locomotor rhythm,GO:0045931~positive regulation of mitotic cell cycle,GO:0050821~protein stabilization,
DistUt	UP	CBX8	chromobox 8(CBX8)	GO:0000122~negative regulation of transcription from RNA polymerase II promoter,GO:0016574~histone ubiquitination,
DistUt	UP	NTRK3	neurotrophic receptor tyrosine kinase 3(NTRK3)	GO:0000187~activation of MAPK activity,GO:0001933~negative regulation of protein phosphorylation,GO:0007169~transmembrane receptor protein tyrosine kinase signaling pathway,GO:0007399~nervous system development,GO:0007507~heart development,GO:0007623~circadian rhythm,GO:0008284~positive regulation of cell proliferation,GO:0010628~positive regulation of gene expression,GO:0018108~peptidyl-tyrosine phosphorylation,GO:0030154~cell differentiation,GO:0030335~positive regulation of cell migration,GO:0032148~activation of protein kinase B activity,GO:0033138~positive regulation of peptidyl-serine phosphorylation,GO:0038179~neurotrophin signaling pathway,GO:0042490~mechanoreceptor differentiation,GO:0043065~positive regulation of apoptotic process,GO:0048691~positive regulation of axon extension involved in regeneration,GO:0050927~positive regulation of positive chemotaxis,GO:0051965~positive regulation of synapse assembly,GO:0070306~lens fiber cell differentiation,GO:0090630~activation of GTPase activity,GO:2000251~positive regulation of actin cytoskeleton reorganization,
DistUt	UP	RNF167	ring finger protein 167(RNF167)	GO:0000209~protein polyubiquitination,GO:0045786~negative regulation of cell cycle,
DistUt	UP	AAR2	AAR2 splicing factor homolog(AAR2)	GO:0000244~spliceosomal tri-snRNP complex assembly,
DistUt	UP	DHX32	DEAH-box helicase 32 (putative)(DHX32)	GO:0000398~mRNA splicing, via spliceosome,
DistUt	UP	WNT7A	Wnt family member 7A(WNT7A)	GO:0000578~embryonic axis specification,GO:0001502~cartilage condensation,GO:0001525~angiogenesis,GO:0002062~chondrocyte differentiation,GO:0007269~neurotransmitter secretion,GO:0008105~asymmetric protein localization,GO:0009953~dorsal/ventral pattern formation,GO:0010595~positive regulation of endothelial cell migration,GO:0014719~skeletal muscle satellite cell activation,GO:0014834~skeletal muscle satellite cell maintenance involved in skeletal muscle regeneration,GO:0016055~Wnt signaling pathway,GO:0021707~cerebellar granule cell differentiation,GO:0021846~cell proliferation in forebrain,GO:0022009~central nervous system vasculogenesis,GO:0030010~establishment of cell polarity,GO:0030182~neuron differentiation,GO:0031133~regulation of axon diameter,GO:0035019~somatic stem cell population maintenance,GO:0035115~embryonic forelimb morphogenesis,GO:0035116~embryonic hindlimb morphogenesis,GO:0035567~non-canonical Wnt signaling pathway,GO:0035659~Wnt signaling pathway involved in wound healing, spreading of epidermal cells,GO:0042733~embryonic digit morphogenesis,GO:0043066~negative regulation of apoptotic process,GO:0043627~response to estrogen,GO:0045165~cell fate commitment,GO:0045944~positive regulation of transcription from RNA polymerase II promoter,GO:0048103~somatic stem cell division,GO:0048864~stem cell development,GO:0050768~negative regulation of neurogenesis,GO:0050808~synapse organization,GO:0060021~palate development,GO:0060054~positive regulation of epithelial cell proliferation involved in wound healing,GO:0060066~oviduct development,GO:0060070~canonical Wnt signaling pathway,GO:0060997~dendritic spine morphogenesis,GO:0061038~uterus morphogenesis,GO:0071560~cellular response to transforming growth factor beta stimulus,GO:0090263~positive regulation of canonical Wnt signaling pathway,GO:1904891~positive regulation of excitatory synapse assembly,GO:2000463~positive regulation of excitatory postsynaptic potential,
DistUt	UP	SLC7A7	solute carrier family 7 member 7(SLC7A7)	GO:0000821~regulation of arginine metabolic process,

**DistUt**	**UP**	TENM2	teneurin transmembrane protein 2(TENM2)	GO:0000902~cell morphogenesis,GO:0007157~heterophilic cell-cell adhesion via plasma membrane cell adhesion molecules,GO:0007165~signal transduction,GO:0048666~neuron development,GO:0097264~self proteolysis,
**DistUt**	**UP**	CAP2	CAP, adenylate cyclase-associated protein, 2 (yeast)(CAP2)	GO:0000902~cell morphogenesis,GO:0007163~establishment or maintenance of cell polarity,GO:0007165~signal transduction,GO:0008154~actin polymerization or depolymerization,GO:0045761~regulation of adenylate cyclase activity,
**DistUt**	**UP**	PLXDC1	plexin domain containing 1(PLXDC1)	GO:0001525~angiogenesis,
**DistUt**	**UP**	VEGFB	vascular endothelial growth factor B(VEGFB)	GO:0001525~angiogenesis,GO:0001666~response to hypoxia,GO:0001938~positive regulation of endothelial cell proliferation,GO:0006493~protein O-linked glycosylation,GO:0045766~positive regulation of angiogenesis,GO:0048010~vascular endothelial growth factor receptor signaling pathway,GO:0050930~induction of positive chemotaxis,GO:0060048~cardiac muscle contraction,GO:0060754~positive regulation of mast cell chemotaxis,GO:0060976~coronary vasculature development,
**DistUt**	**UP**	POFUT1	protein O-fucosyltransferase 1(POFUT1)	GO:0001525~angiogenesis,GO:0001756~somitogenesis,GO:0006493~protein O-linked glycosylation,GO:0007219~Notch signaling pathway,GO:0007399~nervous system development,GO:0007507~heart development,
**DistUt**	**UP**	UBP1	upstream binding protein 1 (LBP-1a)(UBP1)	GO:0001525~angiogenesis,GO:0006357~regulation of transcription from RNA polymerase II promoter,
**DistUt**	**UP**	CDC25B	cell division cycle 25B(CDC25B)	GO:0001556~oocyte maturation,GO:0006468~protein phosphorylation,GO:0007144~female meiosis I,GO:0032467~positive regulation of cytokinesis,GO:0045860~positive regulation of protein kinase activity,GO:0045931~positive regulation of mitotic cell cycle,GO:0051301~cell division,
**DistUt**	**UP**	CUL4A	cullin 4A(CUL4A)	GO:0001701~in utero embryonic development,GO:0006974~cellular response to DNA damage stimulus,GO:0008284~positive regulation of cell proliferation,GO:0030097~hemopoiesis,GO:0030853~negative regulation of granulocyte differentiation,GO:0035019~somatic stem cell population maintenance,GO:0042787~protein ubiquitination involved in ubiquitin-dependent protein catabolic process,GO:0043161~proteasome-mediated ubiquitin-dependent protein catabolic process,GO:0051246~regulation of protein metabolic process,GO:1900087~positive regulation of G1/S transition of mitotic cell cycle,GO:2000001~regulation of DNA damage checkpoint,GO:2000819~regulation of nucleotide-excision repair,
**DistUt**	**UP**	FCGR1A	Fc fragment of IgG, high affinity Ia, receptor (CD64)(FCGR1A)	GO:0001788~antibody-dependent cellular cytotoxicity,GO:0001798~positive regulation of type IIa hypersensitivity,GO:0001805~positive regulation of type III hypersensitivity,GO:0006898~receptor-mediated endocytosis,GO:0006910~phagocytosis, recognition,GO:0006911~phagocytosis, engulfment,GO:0042590~antigen processing and presentation of exogenous peptide antigen via MHC class I,GO:0042742~defense response to bacterium,GO:0050766~positive regulation of phagocytosis,
**DistUt**	**UP**	ADRA2A	adrenoceptor alpha 2A(ADRA2A)	GO:0001819~positive regulation of cytokine production,GO:0006940~regulation of smooth muscle contraction,GO:0007194~negative regulation of adenylate cyclase activity,GO:0007267~cell-cell signaling,GO:0019229~regulation of vasoconstriction,GO:0030168~platelet activation,GO:0030335~positive regulation of cell migration,GO:0030818~negative regulation of cAMP biosynthetic process,GO:0032148~activation of protein kinase B activity,GO:0032870~cellular response to hormone stimulus,GO:0035625~epidermal growth factor-activated receptor transactivation by G-protein coupled receptor signaling pathway,GO:0042593~glucose homeostasis,GO:0043268~positive regulation of potassium ion transport,GO:0050995~negative regulation of lipid catabolic process,GO:0051044~positive regulation of membrane protein ectodomain proteolysis,GO:0051926~negative regulation of calcium ion transport,GO:0061179~negative regulation of insulin secretion involved in cellular response to glucose stimulus,GO:0071878~negative regulation of adrenergic receptor signaling pathway,GO:0071880~adenylate cyclase-activating adrenergic receptor signaling pathway,GO:0071881~adenylate cyclase-inhibiting adrenergic receptor signaling pathway,GO:0071882~phospholipase C-activating adrenergic receptor signaling pathway,GO:0071883~activation of MAPK activity by adrenergic receptor signaling pathway,GO:0090303~positive regulation of wound healing,GO:1901020~negative regulation of calcium ion transmembrane transporter activity,
**DistUt**	**UP**	HSP90AB1	heat shock protein 90 alpha family class B member 1(HSP90AB1)	GO:0001890~placenta development,GO:0006457~protein folding,GO:0006950~response to stress,GO:0019062~virion attachment to host cell,GO:0031396~regulation of protein ubiquitination,GO:0032435~negative regulation of proteasomal ubiquitin-dependent protein catabolic process,GO:0043066~negative regulation of apoptotic process,GO:0060334~regulation of interferon-gamma-mediated signaling pathway,GO:0060338~regulation of type I interferon-mediated signaling pathway,GO:0071353~cellular response to interleukin-4,GO:0097435~fibril organization,
**DistUt**	**UP**	ARRB2	arrestin beta 2(ARRB2)	GO:0002031~G-protein coupled receptor internalization,GO:0002032~desensitization of G-protein coupled receptor protein signaling pathway by arrestin,GO:0002092~positive regulation of receptor internalization,GO:0006366~transcription from RNA polymerase II promoter,GO:0007179~transforming growth factor beta receptor signaling pathway,GO:0007628~adult walking behavior,GO:0016567~protein ubiquitination,GO:0031397~negative regulation of protein ubiquitination,GO:0031398~positive regulation of protein ubiquitination,GO:0032088~negative regulation of NF-kappaB transcription factor activity,GO:0032226~positive regulation of synaptic transmission, dopaminergic,GO:0032691~negative regulation of interleukin-1 beta production,GO:0032695~negative regulation of interleukin-12 production,GO:0032715~negative regulation of interleukin-6 production,GO:0032720~negative regulation of tumor necrosis factor production,GO:0034122~negative regulation of toll-like receptor signaling pathway,GO:0043161~proteasome-mediated ubiquitin-dependent protein catabolic process,GO:0045953~negative regulation of natural killer cell mediated cytotoxicity,GO:0051897~positive regulation of protein kinase B signaling,GO:0060326~cell chemotaxis,GO:0060765~regulation of androgen receptor signaling pathway,GO:0070374~positive regulation of ERK1 and ERK2 cascade,
**DistUt**	**UP**	UNC13D	unc-13 homolog D(UNC13D)	GO:0002432~granuloma formation,GO:0002467~germinal center formation,GO:0006909~phagocytosis,GO:0043304~regulation of mast cell degranulation,GO:0043320~natural killer cell degranulation,GO:0051607~defense response to virus,GO:1900026~positive regulation of substrate adhesion-dependent cell spreading,GO:1903307~positive regulation of regulated secretory pathway,
**DistUt**	**UP**	DHRS3	dehydrogenase/reductase 3(DHRS3)	GO:0003151~outflow tract morphogenesis,GO:0030278~regulation of ossification,GO:0042572~retinol metabolic process,GO:0048387~negative regulation of retinoic acid receptor signaling pathway,GO:0060021~palate development,GO:0060349~bone morphogenesis,GO:0060411~cardiac septum morphogenesis,
**DistUt**	**UP**	GNMT	glycine N-methyltransferase(GNMT)	GO:0005977~glycogen metabolic process,GO:0006111~regulation of gluconeogenesis,GO:0006555~methionine metabolic process,GO:0006730~one-carbon metabolic process,GO:0032259~methylation,GO:0046500~S-adenosylmethionine metabolic process,GO:0051289~protein homotetramerization,
**DistUt**	**UP**	ALDH2	aldehyde dehydrogenase 2 family (mitochondrial)(ALDH2)	GO:0006068~ethanol catabolic process,GO:0055114~oxidation-reduction process,
**DistUt**	**UP**	FMO2	flavin containing monooxygenase 2(FMO2)	GO:0006082~organic acid metabolic process,GO:0006805~xenobiotic metabolic process,GO:0009404~toxin metabolic process,GO:0017144~drug metabolic process,GO:0055114~oxidation-reduction process,GO:0070995~NADPH oxidation,GO:0072592~oxygen metabolic process,
**DistUt**	**UP**	IDH3G	isocitrate dehydrogenase 3 (NAD(+)) gamma(IDH3G)	GO:0006099~tricarboxylic acid cycle,GO:0006102~isocitrate metabolic process,
**DistUt**	**UP**	NDUFA10	NADH:ubiquinone oxidoreductase subunit A10(NDUFA10)	GO:0006120~mitochondrial electron transport, NADH to ubiquinone,
**DistUt**	**UP**	GMPR	guanosine monophosphate reductase(GMPR)	GO:0006144~purine nucleobase metabolic process,GO:0015951~purine ribonucleotide interconversion,
**DistUt**	**UP**	ALDH6A1	aldehyde dehydrogenase 6 family member A1(ALDH6A1)	GO:0006210~thymine catabolic process,GO:0006574~valine catabolic process,GO:0050873~brown fat cell differentiation,
**DistUt**	**UP**	TET3	tet methylcytosine dioxygenase 3(TET3)	GO:0006211~5-methylcytosine catabolic process,GO:0006493~protein O-linked glycosylation,GO:0044727~DNA demethylation of male pronucleus,GO:0045944~positive regulation of transcription from RNA polymerase II promoter,GO:0070989~oxidative demethylation,GO:0080111~DNA demethylation,GO:0080182~histone H3-K4 trimethylation,
**DistUt**	**UP**	TDP1	tyrosyl-DNA phosphodiesterase 1(TDP1)	GO:0006281~DNA repair,
**DistUt**	**UP**	POLR1D	RNA polymerase I subunit D(POLR1D)	GO:0006351~transcription, DNA-templated,GO:0006360~transcription from RNA polymerase I promoter,GO:0006383~transcription from RNA polymerase III promoter,
**DistUt**	**UP**	TBL1X	transducin beta like 1X-linked(TBL1X)	GO:0006357~regulation of transcription from RNA polymerase II promoter,GO:0016575~histone deacetylation,GO:0043161~proteasome-mediated ubiquitin-dependent protein catabolic process,GO:0043627~response to estrogen,GO:0045444~fat cell differentiation,GO:0045892~negative regulation of transcription, DNA-templated,GO:0045944~positive regulation of transcription from RNA polymerase II promoter,GO:0048545~response to steroid hormone,
**DistUt**	**UP**	POLR1A	RNA polymerase I subunit A(POLR1A)	GO:0006360~transcription from RNA polymerase I promoter,
**DistUt**	**UP**	PDF	peptide deformylase (mitochondrial)(PDF)	GO:0006412~translation,GO:0008284~positive regulation of cell proliferation,GO:0018206~peptidyl-methionine modification,GO:0031365~N-terminal protein amino acid modification,
**DistUt**	**UP**	TRAP1	TNF receptor associated protein 1(TRAP1)	GO:0006457~protein folding,GO:0006950~response to stress,GO:0009386~translational attenuation,GO:1901856~negative regulation of cellular respiration,

**DistUt**	**UP**	HSPE1	heat shock protein family E (Hsp10) member 1(HSPE1)	GO:0006457~protein folding,GO:0006986~response to unfolded protein,GO:0051085~chaperone mediated protein folding requiring cofactor,
**DistUt**	**UP**	DGUOK	deoxyguanosine kinase(DGUOK)	GO:0006468~protein phosphorylation,GO:0046070~dGTP metabolic process,GO:0046122~purine deoxyribonucleoside metabolic process,
**DistUt**	**UP**	CAPN15	calpain 15(CAPN15)	GO:0006508~proteolysis,
**DistUt**	**UP**	CAPN5	calpain 5(CAPN5)	GO:0006508~proteolysis,
**DistUt**	**UP**	FAH	fumarylacetoacetate hydrolase(FAH)	GO:0006527~arginine catabolic process,GO:0009072~aromatic amino acid family metabolic process,
**DistUt**	**UP**	HADH	hydroxyacyl-CoA dehydrogenase(HADH)	GO:0006635~fatty acid beta-oxidation,
**DistUt**	**UP**	CHKA	choline kinase alpha(CHKA)	GO:0006646~phosphatidylethanolamine biosynthetic process,
**DistUt**	**UP**	PLD2	phospholipase D2(PLD2)	GO:0006654~phosphatidic acid biosynthetic process,GO:0036465~synaptic vesicle recycling,GO:0048017~inositol lipid-mediated signaling,GO:0048870~cell motility,
**DistUt**	**UP**	SPR	sepiapterin reductase (7,8-dihydrobiopterin:NADP+ oxidoreductase)(SPR)	GO:0006729~tetrahydrobiopterin biosynthetic process,GO:0006809~nitric oxide biosynthetic process,
**DistUt**	**UP**	PCBD1	pterin-4 alpha-carbinolamine dehydratase 1(PCBD1)	GO:0006729~tetrahydrobiopterin biosynthetic process,GO:0051289~protein homotetramerization,GO:0051291~protein heterooligomerization,
**DistUt**	**UP**	COQ4	coenzyme Q4(COQ4)	GO:0006744~ubiquinone biosynthetic process,
**DistUt**	**UP**	GSTK1	glutathione S-transferase kappa 1(GSTK1)	GO:0006749~glutathione metabolic process,GO:0030855~epithelial cell differentiation,
**DistUt**	**UP**	LOC100624445	gamma-glutamylcyclotransferase-like(LOC100624445)	GO:0006750~glutathione biosynthetic process,
**DistUt**	**UP**	SLC35F1	solute carrier family 35 member F1(SLC35F1)	GO:0006810~transport,
**DistUt**	**UP**	ABCA9	ATP binding cassette subfamily A member 9(ABCA9)	GO:0006869~lipid transport,
**DistUt**	**UP**	ATP2C2	ATPase secretory pathway Ca2+ transporting 2(ATP2C2)	GO:0006874~cellular calcium ion homeostasis,GO:0061180~mammary gland epithelium development,GO:0072661~protein targeting to plasma membrane,GO:0090280~positive regulation of calcium ion import,
**DistUt**	**UP**	STX10	syntaxin 10(STX10)	GO:0006886~intracellular protein transport,GO:0006906~vesicle fusion,GO:0032880~regulation of protein localization,GO:0042147~retrograde transport, endosome to Golgi,GO:0048193~Golgi vesicle transport,GO:0048278~vesicle docking,
**DistUt**	**UP**	MAP4K2	mitogen-activated protein kinase kinase kinase kinase 2(MAP4K2)	GO:0006903~vesicle targeting,GO:0007257~activation of JUN kinase activity,
**DistUt**	**UP**	CYFIP2	cytoplasmic FMR1 interacting protein 2(CYFIP2)	GO:0006915~apoptotic process,GO:0016337~single organismal cell-cell adhesion,GO:0097202~activation of cysteine-type endopeptidase activity,
**DistUt**	**UP**	LOC100153966	activator of 90 kDa heat shock protein ATPase homolog 1(LOC100153966)	GO:0006950~response to stress,
**DistUt**	**UP**	STRIP2	striatin interacting protein 2(STRIP2)	GO:0007010~cytoskeleton organization,GO:0008360~regulation of cell shape,GO:0016477~cell migration,
**DistUt**	**UP**	MSLN	mesothelin(MSLN)	GO:0007160~cell-matrix adhesion,
**DistUt**	**UP**	LRRC1	leucine rich repeat containing 1(LRRC1)	GO:0007165~signal transduction,GO:0032088~negative regulation of NF-kappaB transcription factor activity,
**DistUt**	**UP**	CSF1R	colony stimulating factor 1 receptor(CSF1R)	GO:0007169~transmembrane receptor protein tyrosine kinase signaling pathway,GO:0007411~axon guidance,GO:0008283~cell proliferation,GO:0008284~positive regulation of cell proliferation,GO:0008285~negative regulation of cell proliferation,GO:0008360~regulation of cell shape,GO:0019221~cytokine-mediated signaling pathway,GO:0021772~olfactory bulb development,GO:0021879~forebrain neuron differentiation,GO:0030316~osteoclast differentiation,GO:0030335~positive regulation of cell migration,GO:0031529~ruffle organization,GO:0042517~positive regulation of tyrosine phosphorylation of Stat3 protein,GO:0043066~negative regulation of apoptotic process,GO:0045217~cell-cell junction maintenance,GO:0046488~phosphatidylinositol metabolic process,GO:0046777~protein autophosphorylation,GO:0048015~phosphatidylinositol-mediated signaling,GO:0061098~positive regulation of protein tyrosine kinase activity,GO:0070374~positive regulation of ERK1 and ERK2 cascade,GO:0071902~positive regulation of protein serine/threonine kinase activity,GO:0090197~positive regulation of chemokine secretion,GO:2000249~regulation of actin cytoskeleton reorganization,
**DistUt**	**UP**	LOC100152489	olfactory receptor 13C8(LOC100152489)	GO:0007186~G-protein coupled receptor signaling pathway,GO:0007608~sensory perception of smell,
**DistUt**	**UP**	KRT19	keratin 19(KRT19)	GO:0007219~Notch signaling pathway,GO:0043627~response to estrogen,GO:0045214~sarcomere organization,GO:0060706~cell differentiation involved in embryonic placenta development,
**DistUt**	**UP**	CACNB2	calcium voltage-gated channel auxiliary subunit beta 2(CACNB2)	GO:0007268~chemical synaptic transmission,GO:0007528~neuromuscular junction development,GO:0007601~visual perception,GO:0051928~positive regulation of calcium ion transport,GO:0070509~calcium ion import,GO:0086091~regulation of heart rate by cardiac conduction,GO:0098912~membrane depolarization during atrial cardiac muscle cell action potential,GO:1901385~regulation of voltage-gated calcium channel activity,
**DistUt**	**UP**	LOC100513474	platelet-activating factor acetylhydrolase IB subunit gamma(LOC100513474)	GO:0007420~brain development,
**DistUt**	**UP**	GABRB2	gamma-aminobutyric acid type A receptor beta2 subunit(GABRB2)	GO:0007605~sensory perception of sound,GO:0043524~negative regulation of neuron apoptotic process,GO:0060119~inner ear receptor cell development,GO:0060384~innervation,GO:0090102~cochlea development,
**DistUt**	**UP**	NDUFV1	NADH:ubiquinone oxidoreductase core subunit V1(NDUFV1)	GO:0009060~aerobic respiration,
**DistUt**	**UP**	PRPSAP1	phosphoribosyl pyrophosphate synthetase associated protein 1(PRPSAP1)	GO:0009116~nucleoside metabolic process,GO:0009165~nucleotide biosynthetic process,
**DistUt**	**UP**	AQP9	aquaporin 9(AQP9)	GO:0009992~cellular water homeostasis,GO:0015837~amine transport,GO:0034220~ion transmembrane transport,GO:0046689~response to mercury ion,GO:0071320~cellular response to cAMP,GO:0071918~urea transmembrane transport,
**DistUt**	**UP**	CEP68	centrosomal protein 68(CEP68)	GO:0010457~centriole-centriole cohesion,GO:0033365~protein localization to organelle,
**DistUt**	**UP**	NCKIPSD	NCK interacting protein with SH3 domain(NCKIPSD)	GO:0010976~positive regulation of neuron projection development,
**DistUt**	**UP**	STARD10	StAR related lipid transfer domain containing 10(STARD10)	GO:0015914~phospholipid transport,GO:0032782~bile acid secretion,GO:0035360~positive regulation of peroxisome proliferator activated receptor signaling pathway,
**DistUt**	**UP**	ATP5E	ATP synthase, H+ transporting, mitochondrial F1 complex, epsilon subunit(ATP5E)	GO:0015986~ATP synthesis coupled proton transport,
**DistUt**	**UP**	ANXA9	annexin A9(ANXA9)	GO:0016337~single organismal cell-cell adhesion,
**DistUt**	**UP**	GPA33	glycoprotein A33(GPA33)	GO:0016337~single organismal cell-cell adhesion,
**DistUt**	**UP**	RNF4	ring finger protein 4(RNF4)	GO:0016567~protein ubiquitination,GO:0033234~negative regulation of protein sumoylation,GO:0043161~proteasome-mediated ubiquitin-dependent protein catabolic process,GO:0045944~positive regulation of transcription from RNA polymerase II promoter,GO:0046685~response to arsenic-containing substance,GO:0090169~regulation of spindle assembly,GO:0090234~regulation of kinetochore assembly,
**DistUt**	**UP**	NIPSNAP1	nipsnap homolog 1 (C. elegans)(NIPSNAP1)	GO:0019233~sensory perception of pain,

**DistUt**	**UP**	DGAT1	diacylglycerol O-acyltransferase 1(DGAT1)	GO:0019432~triglyceride biosynthetic process,GO:0019915~lipid storage,GO:0034379~very-low-density lipoprotein particle assembly,GO:0035336~long-chain fatty-acyl-CoA metabolic process,GO:0046339~diacylglycerol metabolic process,GO:0055089~fatty acid homeostasis,
**DistUt**	**UP**	RUFY1	RUN and FYVE domain containing 1(RUFY1)	GO:0030100~regulation of endocytosis,
**DistUt**	**UP**	KBTBD4	kelch repeat and BTB domain containing 4(KBTBD4)	GO:0030162~regulation of proteolysis,GO:0042787~protein ubiquitination involved in ubiquitin-dependent protein catabolic process,GO:0043161~proteasome-mediated ubiquitin-dependent protein catabolic process,
**DistUt**	**UP**	OLFML2A	olfactomedin like 2A(OLFML2A)	GO:0030198~extracellular matrix organization,
**DistUt**	**UP**	UBQLN4	ubiquilin 4(UBQLN4)	GO:0032434~regulation of proteasomal ubiquitin-dependent protein catabolic process,GO:1901097~negative regulation of autophagosome maturation,
**DistUt**	**UP**	TRIM9	tripartite motif containing 9(TRIM9)	GO:0043161~proteasome-mediated ubiquitin-dependent protein catabolic process,
**DistUt**	**UP**	ZBTB7C	zinc finger and BTB domain containing 7C(ZBTB7C)	GO:0045600~positive regulation of fat cell differentiation,GO:0045944~positive regulation of transcription from RNA polymerase II promoter,GO:1903025~regulation of RNA polymerase II regulatory region sequence-specific DNA binding,
**DistUt**	**UP**	BRINP3	BMP/retinoic acid inducible neural specific 3(BRINP3)	GO:0045666~positive regulation of neuron differentiation,GO:0045930~negative regulation of mitotic cell cycle,GO:0071300~cellular response to retinoic acid,
**DistUt**	**UP**	KCTD1	potassium channel tetramerization domain containing 1(KCTD1)	GO:0045892~negative regulation of transcription, DNA-templated,GO:0051260~protein homooligomerization,
**DistUt**	**UP**	MTMR7	myotubularin related protein 7(MTMR7)	GO:0046855~inositol phosphate dephosphorylation,GO:0046856~phosphatidylinositol dephosphorylation,
**DistUt**	**UP**	DOPEY2	dopey family member 2(DOPEY2)	GO:0050890~cognition,
**DistUt**	**UP**	GARNL3	GTPase activating Rap/RanGAP domain like 3(GARNL3)	GO:0051056~regulation of small GTPase mediated signal transduction,
**DistUt**	**UP**	KCTD12	potassium channel tetramerization domain containing 12(KCTD12)	GO:0051260~protein homooligomerization,
**DistUt**	**UP**	ATP2A3	ATPase sarcoplasmic/endoplasmic reticulum Ca2+ transporting 3(ATP2A3)	GO:0070588~calcium ion transmembrane transport,
**DistUt**	**UP**	MPP1	membrane palmitoylated protein 1(MPP1)	GO:0090022~regulation of neutrophil chemotaxis,
**DistUt**	**UP**	WRAP73	WD repeat containing, antisense to TP73(WRAP73)	GO:0090307~mitotic spindle assembly,GO:1902857~positive regulation of nonmotile primary cilium assembly,
**DistUt**	**UP**	LOC100521617	myelin and lymphocyte protein(LOC100521617)	GO:1902043~positive regulation of extrinsic apoptotic signaling pathway via death domain receptors,
**DistUt**	**DOWN**	WIPI1	WD repeat domain, phosphoinositide interacting 1(WIPI1)	GO:0000045~autophagosome assembly,GO:0000422~mitophagy,GO:0006497~protein lipidation,GO:0034497~protein localization to pre-autophagosomal structure,GO:0044804~nucleophagy,GO:0048203~vesicle targeting, trans-Golgi to endosome,
**DistUt**	**DOWN**	FGFR1	fibroblast growth factor receptor 1(FGFR1)	GO:0000122~negative regulation of transcription from RNA polymerase II promoter,GO:0001525~angiogenesis,GO:0001657~ureteric bud development,GO:0001701~in utero embryonic development,GO:0001759~organ induction,GO:0002053~positive regulation of mesenchymal cell proliferation,GO:0002062~chondrocyte differentiation,GO:0007037~vacuolar phosphate transport,GO:0007605~sensory perception of sound,GO:0008284~positive regulation of cell proliferation,GO:0010863~positive regulation of phospholipase C activity,GO:0010976~positive regulation of neuron projection development,GO:0021847~ventricular zone neuroblast division,GO:0030326~embryonic limb morphogenesis,GO:0030901~midbrain development,GO:0035607~fibroblast growth factor receptor signaling pathway involved in orbitofrontal cortex development,GO:0042472~inner ear morphogenesis,GO:0042473~outer ear morphogenesis,GO:0042474~middle ear morphogenesis,GO:0043406~positive regulation of MAP kinase activity,GO:0045787~positive regulation of cell cycle,GO:0046777~protein autophosphorylation,GO:0048339~paraxial mesoderm development,GO:0048378~regulation of lateral mesodermal cell fate specification,GO:0048469~cell maturation,GO:0048762~mesenchymal cell differentiation,GO:0060045~positive regulation of cardiac muscle cell proliferation,GO:0060117~auditory receptor cell development,GO:0060445~branching involved in salivary gland morphogenesis,GO:0060484~lung-associated mesenchyme development,GO:0060665~regulation of branching involved in salivary gland morphogenesis by mesenchymal-epithelial signaling,GO:0070640~vitamin D3 metabolic process,GO:0090080~positive regulation of MAPKKK cascade by fibroblast growth factor receptor signaling pathway,GO:0090272~negative regulation of fibroblast growth factor production,GO:2000546~positive regulation of endothelial cell chemotaxis to fibroblast growth factor,GO:2000830~positive regulation of parathyroid hormone secretion,GO:2001239~regulation of extrinsic apoptotic signaling pathway in absence of ligand,
**DistUt**	**DOWN**	TCF7L2	transcription factor 7 like 2(TCF7L2)	GO:0000122~negative regulation of transcription from RNA polymerase II promoter,GO:0001568~blood vessel development,GO:0006357~regulation of transcription from RNA polymerase II promoter,GO:0007050~cell cycle arrest,GO:0008283~cell proliferation,GO:0010909~positive regulation of heparan sulfate proteoglycan biosynthetic process,GO:0032024~positive regulation of insulin secretion,GO:0032092~positive regulation of protein binding,GO:0032350~regulation of hormone metabolic process,GO:0042593~glucose homeostasis,GO:0043433~negative regulation of sequence-specific DNA binding transcription factor activity,GO:0043570~maintenance of DNA repeat elements,GO:0044334~canonical Wnt signaling pathway involved in positive regulation of epithelial to mesenchymal transition,GO:0045444~fat cell differentiation,GO:0045944~positive regulation of transcription from RNA polymerase II promoter,GO:0046827~positive regulation of protein export from nucleus,GO:0048625~myoblast fate commitment,GO:0048660~regulation of smooth muscle cell proliferation,GO:0051897~positive regulation of protein kinase B signaling,GO:0060070~canonical Wnt signaling pathway,GO:0090090~negative regulation of canonical Wnt signaling pathway,GO:2000675~negative regulation of type B pancreatic cell apoptotic process,GO:2001237~negative regulation of extrinsic apoptotic signaling pathway,
**DistUt**	**DOWN**	TDG	thymine-DNA glycosylase(TDG)	GO:0000122~negative regulation of transcription from RNA polymerase II promoter,GO:0006285~base-excision repair, AP site formation,GO:0006298~mismatch repair,GO:0009790~embryo development,GO:0032091~negative regulation of protein binding,GO:0035562~negative regulation of chromatin binding,GO:0040029~regulation of gene expression, epigenetic,GO:0080111~DNA demethylation,
**DistUt**	**DOWN**	LOC100736857	microphthalmia-associated transcription factor-like(LOC100736857)	GO:0000122~negative regulation of transcription from RNA polymerase II promoter,GO:0006461~protein complex assembly,GO:0030318~melanocyte differentiation,GO:0042981~regulation of apoptotic process,GO:0045670~regulation of osteoclast differentiation,GO:0097531~mast cell migration,GO:2000144~positive regulation of DNA-templated transcription, initiation,
**DistUt**	**DOWN**	MOSPD1	motile sperm domain containing 1(MOSPD1)	GO:0000122~negative regulation of transcription from RNA polymerase II promoter,GO:0045944~positive regulation of transcription from RNA polymerase II promoter,
**DistUt**	**DOWN**	PRDM1	PR/SET domain 1(PRDM1)	GO:0000122~negative regulation of transcription from RNA polymerase II promoter,GO:0048869~cellular developmental process,
**DistUt**	**DOWN**	VCAN	versican(VCAN)	GO:0001501~skeletal system development,GO:0001649~osteoblast differentiation,GO:0007155~cell adhesion,GO:0007417~central nervous system development,
**DistUt**	**DOWN**	FBN1	fibrillin 1(FBN1)	GO:0001501~skeletal system development,GO:0006006~glucose metabolic process,GO:0007507~heart development,GO:0009653~anatomical structure morphogenesis,GO:0010737~protein kinase A signaling,GO:0034199~activation of protein kinase A activity,GO:0042593~glucose homeostasis,GO:0043010~camera-type eye development,GO:0048048~embryonic eye morphogenesis,GO:0048050~post-embryonic eye morphogenesis,GO:0090287~regulation of cellular response to growth factor stimulus,
**DistUt**	**DOWN**	COL5A2	collagen type V alpha 2 chain(COL5A2)	GO:0001501~skeletal system development,GO:0030199~collagen fibril organization,GO:0043588~skin development,GO:0048592~eye morphogenesis,GO:0071230~cellular response to amino acid stimulus,GO:1903225~negative regulation of endodermal cell differentiation,
**DistUt**	**DOWN**	EIF2AK3	eukaryotic translation initiation factor 2 alpha kinase 3(EIF2AK3)	GO:0001503~ossification,GO:0001525~angiogenesis,GO:0006983~ER overload response,GO:0010575~positive regulation of vascular endothelial growth factor production,GO:0030968~endoplasmic reticulum unfolded protein response,GO:0031018~endocrine pancreas development,GO:0034198~cellular response to amino acid starvation,GO:0036492~eiF2alpha phosphorylation in response to endoplasmic reticulum stress,GO:0042149~cellular response to glucose starvation,GO:0045943~positive regulation of transcription from RNA polymerase I promoter,GO:0046777~protein autophosphorylation,GO:0051260~protein homooligomerization,GO:0070417~cellular response to cold,GO:1902235~regulation of endoplasmic reticulum stress-induced intrinsic apoptotic signaling pathway,
**DistUt**	**DOWN**	DMP1	dentin matrix acidic phosphoprotein 1(DMP1)	GO:0001503~ossification,GO:0010811~positive regulation of cell-substrate adhesion,GO:0030198~extracellular matrix organization,GO:0070173~regulation of enamel mineralization,

**DistUt**	**DOWN**	ACKR3	atypical chemokine receptor 3(ACKR3)	GO:0001525~angiogenesis,GO:0001570~vasculogenesis,GO:0002230~positive regulation of defense response to virus by host,GO:0006935~chemotaxis,GO:0031623~receptor internalization,GO:0070374~positive regulation of ERK1 and ERK2 cascade,GO:0098792~xenophagy,GO:1902230~negative regulation of intrinsic apoptotic signaling pathway in response to DNA damage,
**DistUt**	**DOWN**	HIF1A	hypoxia inducible factor 1, alpha subunit (basic helix-loop-helix transcription factor)(HIF1A)	GO:0001525~angiogenesis,GO:0001755~neural crest cell migration,GO:0001837~epithelial to mesenchymal transition,GO:0001892~embryonic placenta development,GO:0001922~B-1 B cell homeostasis,GO:0001947~heart looping,GO:0002052~positive regulation of neuroblast proliferation,GO:0002248~connective tissue replacement involved in inflammatory response wound healing,GO:0003151~outflow tract morphogenesis,GO:0003208~cardiac ventricle morphogenesis,GO:0006089~lactate metabolic process,GO:0006110~regulation of glycolytic process,GO:0006351~transcription, DNA-templated,GO:0006879~cellular iron ion homeostasis,GO:0007595~lactation,GO:0008542~visual learning,GO:0010573~vascular endothelial growth factor production,GO:0010575~positive regulation of vascular endothelial growth factor production,GO:0010634~positive regulation of epithelial cell migration,GO:0010870~positive regulation of receptor biosynthetic process,GO:0014850~response to muscle activity,GO:0016239~positive regulation of macroautophagy,GO:0019896~axonal transport of mitochondrion,GO:0021502~neural fold elevation formation,GO:0021987~cerebral cortex development,GO:0030502~negative regulation of bone mineralization,GO:0030949~positive regulation of vascular endothelial growth factor receptor signaling pathway,GO:0032007~negative regulation of TOR signaling,GO:0032364~oxygen homeostasis,GO:0032909~regulation of transforming growth factor beta2 production,GO:0032963~collagen metabolic process,GO:0035162~embryonic hemopoiesis,GO:0035774~positive regulation of insulin secretion involved in cellular response to glucose stimulus,GO:0042541~hemoglobin biosynthetic process,GO:0042593~glucose homeostasis,GO:0043619~regulation of transcription from RNA polymerase II promoter in response to oxidative stress,GO:0045648~positive regulation of erythrocyte differentiation,GO:0045766~positive regulation of angiogenesis,GO:0045926~negative regulation of growth,GO:0045944~positive regulation of transcription from RNA polymerase II promoter,GO:0046716~muscle cell cellular homeostasis,GO:0046886~positive regulation of hormone biosynthetic process,GO:0048546~digestive tract morphogenesis,GO:0050790~regulation of catalytic activity,GO:0051216~cartilage development,GO:0051541~elastin metabolic process,GO:0060574~intestinal epithelial cell maturation,GO:0061030~epithelial cell differentiation involved in mammary gland alveolus development,GO:0061072~iris morphogenesis,GO:0061298~retina vasculature development in camera-type eye,GO:0061419~positive regulation of transcription from RNA polymerase II promoter in response to hypoxia,GO:0070244~negative regulation of thymocyte apoptotic process,GO:0071347~cellular response to interleukin-1,GO:0071542~dopaminergic neuron differentiation,GO:0097411~hypoxia-inducible factor-1alpha signaling pathway,GO:1902895~positive regulation of pri-miRNA transcription from RNA polymerase II promoter,GO:1903377~negative regulation of oxidative stress-induced neuron intrinsic apoptotic signaling pathway,GO:1903599~positive regulation of mitophagy,GO:1903715~regulation of aerobic respiration,GO:2000378~negative regulation of reactive oxygen species metabolic process,GO:2001054~negative regulation of mesenchymal cell apoptotic process,
**DistUt**	**DOWN**	ENPEP	glutamyl aminopeptidase(ENPEP)	GO:0001525~angiogenesis,GO:0002003~angiotensin maturation,GO:0003081~regulation of systemic arterial blood pressure by renin-angiotensin,GO:0006508~proteolysis,GO:0008217~regulation of blood pressure,GO:0008283~cell proliferation,GO:0016477~cell migration,GO:0032835~glomerulus development,GO:0043171~peptide catabolic process,
**DistUt**	**DOWN**	SRPX2	sushi repeat containing protein, X-linked 2(SRPX2)	GO:0001525~angiogenesis,GO:0016337~single organismal cell-cell adhesion,GO:0042325~regulation of phosphorylation,GO:0048870~cell motility,GO:0051965~positive regulation of synapse assembly,GO:0071625~vocalization behavior,GO:0090050~positive regulation of cell migration involved in sprouting angiogenesis,
**DistUt**	**DOWN**	COL4A1	collagen type IV alpha 1 chain(COL4A1)	GO:0001569~patterning of blood vessels,GO:0007420~brain development,GO:0007528~neuromuscular junction development,GO:0061304~retinal blood vessel morphogenesis,GO:0061333~renal tubule morphogenesis,GO:0071230~cellular response to amino acid stimulus,GO:0071711~basement membrane organization,
**DistUt**	**DOWN**	GJA1	gap junction protein alpha 1(GJA1)	GO:0001649~osteoblast differentiation,GO:0001701~in utero embryonic development,GO:0001764~neuron migration,GO:0001947~heart looping,GO:0002070~epithelial cell maturation,GO:0002088~lens development in camera-type eye,GO:0002931~response to ischemia,GO:0003294~atrial ventricular junction remodeling,GO:0007154~cell communication,GO:0007267~cell-cell signaling,GO:0007507~heart development,GO:0007512~adult heart development,GO:0009611~response to wounding,GO:0010628~positive regulation of gene expression,GO:0010629~negative regulation of gene expression,GO:0010643~cell communication by chemical coupling,GO:0030308~negative regulation of cell growth,GO:0030500~regulation of bone mineralization,GO:0036120~cellular response to platelet-derived growth factor stimulus,GO:0042733~embryonic digit morphogenesis,GO:0043123~positive regulation of I-kappaB kinase/NF-kappaB signaling,GO:0043403~skeletal muscle tissue regeneration,GO:0045216~cell-cell junction organization,GO:0045669~positive regulation of osteoblast differentiation,GO:0045844~positive regulation of striated muscle tissue development,GO:0046850~regulation of bone remodeling,GO:0048514~blood vessel morphogenesis,GO:0055085~transmembrane transport,GO:0060156~milk ejection,GO:0060307~regulation of ventricular cardiac muscle cell membrane repolarization,GO:0060371~regulation of atrial cardiac muscle cell membrane depolarization,GO:0060373~regulation of ventricular cardiac muscle cell membrane depolarization,GO:0061337~cardiac conduction,GO:0071673~positive regulation of smooth muscle cell chemotaxis,
**DistUt**	**DOWN**	MRC2	mannose receptor C type 2(MRC2)	GO:0001649~osteoblast differentiation,GO:0030574~collagen catabolic process,
**DistUt**	**DOWN**	PLAU	plasminogen activator, urokinase(PLAU)	GO:0001666~response to hypoxia,GO:0006508~proteolysis,GO:0010469~regulation of receptor activity,GO:0014909~smooth muscle cell migration,GO:0014910~regulation of smooth muscle cell migration,GO:0030335~positive regulation of cell migration,GO:0033628~regulation of cell adhesion mediated by integrin,GO:0042127~regulation of cell proliferation,GO:0042730~fibrinolysis,GO:2000097~regulation of smooth muscle cell-matrix adhesion,
**DistUt**	**DOWN**	PLOD2	procollagen-lysine,2-oxoglutarate 5-dioxygenase 2(PLOD2)	GO:0001666~response to hypoxia,GO:0098779~mitophagy in response to mitochondrial depolarization,
**DistUt**	**DOWN**	MAN2A1	mannosidase alpha class 2A member 1(MAN2A1)	GO:0001701~in utero embryonic development,GO:0001889~liver development,GO:0006013~mannose metabolic process,GO:0006491~N-glycan processing,GO:0006517~protein deglycosylation,GO:0007005~mitochondrion organization,GO:0007033~vacuole organization,GO:0007585~respiratory gaseous exchange,GO:0048286~lung alveolus development,GO:0050769~positive regulation of neurogenesis,GO:0060042~retina morphogenesis in camera-type eye,
**DistUt**	**DOWN**	LOC100739844	semaphorin-3A-like(LOC100739844)	GO:0001755~neural crest cell migration,GO:0021772~olfactory bulb development,GO:0048843~negative regulation of axon extension involved in axon guidance,GO:0050919~negative chemotaxis,GO:0071526~semaphorin-plexin signaling pathway,GO:2001224~positive regulation of neuron migration,
**DistUt**	**DOWN**	S1PR3	sphingosine-1-phosphate receptor 3(S1PR3)	GO:0001816~cytokine production,GO:0006954~inflammatory response,GO:0007193~adenylate cyclase-inhibiting G-protein coupled receptor signaling pathway,GO:0007219~Notch signaling pathway,GO:0032651~regulation of interleukin-1 beta production,GO:1903141~negative regulation of establishment of endothelial barrier,
**DistUt**	**DOWN**	AGTR1	angiotensin II receptor type 1(AGTR1)	GO:0001822~kidney development,GO:0007266~Rho protein signal transduction,GO:0010873~positive regulation of cholesterol esterification,GO:0019229~regulation of vasoconstriction,GO:0019722~calcium-mediated signaling,GO:0032270~positive regulation of cellular protein metabolic process,GO:0032430~positive regulation of phospholipase A2 activity,GO:0051482~positive regulation of cytosolic calcium ion concentration involved in phospholipase C-activating G-protein coupled signaling pathway,GO:0060326~cell chemotaxis,GO:0086097~phospholipase C-activating angiotensin-activated signaling pathway,
**DistUt**	**DOWN**	SEC63	SEC63 homolog, protein translocation regulator(SEC63)	GO:0001889~liver development,GO:0006614~SRP-dependent cotranslational protein targeting to membrane,GO:0006620~posttranslational protein targeting to membrane,GO:0006807~nitrogen compound metabolic process,GO:0010259~multicellular organism aging,GO:0072001~renal system development,
**DistUt**	**DOWN**	INPP5F	inositol polyphosphate-5-phosphatase F(INPP5F)	GO:0001921~positive regulation of receptor recycling,GO:0008344~adult locomotory behavior,GO:0014898~cardiac muscle hypertrophy in response to stress,GO:0031161~phosphatidylinositol catabolic process,GO:0042518~negative regulation of tyrosine phosphorylation of Stat3 protein,GO:0046856~phosphatidylinositol dephosphorylation,GO:0048015~phosphatidylinositol-mediated signaling,GO:0048681~negative regulation of axon regeneration,GO:0072583~clathrin-mediated endocytosis,GO:2000145~regulation of cell motility,GO:2001135~regulation of endocytic recycling,
**DistUt**	**DOWN**	PRNP	prion protein(PRNP)	GO:0001933~negative regulation of protein phosphorylation,GO:0006979~response to oxidative stress,GO:0032689~negative regulation of interferon-gamma production,GO:0032700~negative regulation of interleukin-17 production,GO:0032703~negative regulation of interleukin-2 production,GO:0032880~regulation of protein localization,GO:0035690~cellular response to drug,GO:0043066~negative regulation of apoptotic process,GO:0043433~negative regulation of sequence-specific DNA binding transcription factor activity,GO:0046007~negative regulation of activated T cell proliferation,GO:0050860~negative regulation of T cell receptor signaling pathway,GO:0051260~protein homooligomerization,GO:0070885~negative regulation of calcineurin-NFAT signaling cascade,GO:0071280~cellular response to copper ion,GO:1901379~regulation of potassium ion transmembrane transport,
**DistUt**	**DOWN**	PLAUR	plasminogen activator, urokinase receptor(PLAUR)	GO:0001934~positive regulation of protein phosphorylation,GO:0030162~regulation of proteolysis,GO:0038195~urokinase plasminogen activator signaling pathway,GO:0043388~positive regulation of DNA binding,GO:0045742~positive regulation of epidermal growth factor receptor signaling pathway,GO:0090200~positive regulation of release of cytochrome c from mitochondria,GO:2001243~negative regulation of intrinsic apoptotic signaling pathway,GO:2001268~negative regulation of cysteine-type endopeptidase activity involved in apoptotic signaling pathway,

**DistUt**	**DOWN**	CRYGS	crystallin gamma S(CRYGS)	GO:0002009~morphogenesis of an epithelium,GO:0002088~lens development in camera-type eye,
**DistUt**	**DOWN**	ARL6IP5	ADP ribosylation factor like GTPase 6 interacting protein 5(ARL6IP5)	GO:0002037~negative regulation of L-glutamate transport,GO:0008631~intrinsic apoptotic signaling pathway in response to oxidative stress,GO:0010917~negative regulation of mitochondrial membrane potential,GO:0015813~L-glutamate transport,GO:0032874~positive regulation of stress-activated MAPK cascade,GO:0043280~positive regulation of cysteine-type endopeptidase activity involved in apoptotic process,
**DistUt**	**DOWN**	FOXP2	forkhead box P2(FOXP2)	GO:0002053~positive regulation of mesenchymal cell proliferation,GO:0006351~transcription, DNA-templated,GO:0007519~skeletal muscle tissue development,GO:0009791~post-embryonic development,GO:0021549~cerebellum development,GO:0021757~caudate nucleus development,GO:0021758~putamen development,GO:0021987~cerebral cortex development,GO:0040007~growth,GO:0042297~vocal learning,GO:0043010~camera-type eye development,GO:0048286~lung alveolus development,GO:0048745~smooth muscle tissue development,GO:0060013~righting reflex,GO:0060501~positive regulation of epithelial cell proliferation involved in lung morphogenesis,
**DistUt**	**DOWN**	WNT2	Wnt family member 2(WNT2)	GO:0002053~positive regulation of mesenchymal cell proliferation,GO:0016055~Wnt signaling pathway,GO:0030182~neuron differentiation,GO:0033278~cell proliferation in midbrain,GO:0045165~cell fate commitment,GO:0045944~positive regulation of transcription from RNA polymerase II promoter,GO:0048146~positive regulation of fibroblast proliferation,GO:0050769~positive regulation of neurogenesis,GO:0051091~positive regulation of sequence-specific DNA binding transcription factor activity,GO:0055009~atrial cardiac muscle tissue morphogenesis,GO:0060045~positive regulation of cardiac muscle cell proliferation,GO:0060070~canonical Wnt signaling pathway,GO:0060317~cardiac epithelial to mesenchymal transition,GO:0060492~lung induction,GO:0060501~positive regulation of epithelial cell proliferation involved in lung morphogenesis,GO:0060716~labyrinthine layer blood vessel development,GO:0061180~mammary gland epithelium development,GO:0071560~cellular response to transforming growth factor beta stimulus,GO:0090263~positive regulation of canonical Wnt signaling pathway,GO:1904948~midbrain dopaminergic neuron differentiation,
**DistUt**	**DOWN**	CREB3L2	cAMP responsive element binding protein 3 like 2(CREB3L2)	GO:0002062~chondrocyte differentiation,GO:0006888~ER to Golgi vesicle-mediated transport,GO:0030968~endoplasmic reticulum unfolded protein response,
**DistUt**	**DOWN**	B4GALT1	beta-1,4-galactosyltransferase 1(B4GALT1)	GO:0002064~epithelial cell development,GO:0002526~acute inflammatory response,GO:0005989~lactose biosynthetic process,GO:0006012~galactose metabolic process,GO:0006487~protein N-linked glycosylation,GO:0007155~cell adhesion,GO:0007339~binding of sperm to zona pellucida,GO:0007341~penetration of zona pellucida,GO:0008285~negative regulation of cell proliferation,GO:0030198~extracellular matrix organization,GO:0030879~mammary gland development,GO:0045136~development of secondary sexual characteristics,GO:0048754~branching morphogenesis of an epithelial tube,GO:0050900~leukocyte migration,GO:0051270~regulation of cellular component movement,GO:0060046~regulation of acrosome reaction,GO:0060054~positive regulation of epithelial cell proliferation involved in wound healing,GO:0060055~angiogenesis involved in wound healing,GO:0060058~positive regulation of apoptotic process involved in mammary gland involution,
**DistUt**	**DOWN**	ANXA5	annexin A5(ANXA5)	GO:0002230~positive regulation of defense response to virus by host,GO:0010033~response to organic substance,GO:0050819~negative regulation of coagulation,GO:0098779~mitophagy in response to mitochondrial depolarization,GO:0098792~xenophagy,
**DistUt**	**DOWN**	GCNT3	glucosaminyl (N-acetyl) transferase 3, mucin type(GCNT3)	GO:0002426~immunoglobulin production in mucosal tissue,GO:0048729~tissue morphogenesis,GO:0050892~intestinal absorption,GO:0060993~kidney morphogenesis,
**DistUt**	**DOWN**	ABCB1	ATP-binding cassette, sub-family B (MDR/TAP), member 1(ABCB1)	GO:0002481~antigen processing and presentation of exogenous protein antigen via MHC class Ib, TAP-dependent,GO:0002485~antigen processing and presentation of endogenous peptide antigen via MHC class I via ER pathway, TAP-dependent,GO:0002489~antigen processing and presentation of endogenous peptide antigen via MHC class Ib via ER pathway, TAP-dependent,GO:0002591~positive regulation of antigen processing and presentation of peptide antigen via MHC class I,GO:0055085~transmembrane transport,
**DistUt**	**DOWN**	ME1	malic enzyme 1(ME1)	GO:0006090~pyruvate metabolic process,GO:0006108~malate metabolic process,GO:0009725~response to hormone,GO:0051262~protein tetramerization,GO:0055114~oxidation-reduction process,GO:1902031~regulation of NADP metabolic process,
**DistUt**	**DOWN**	NPR2	natriuretic peptide receptor B/guanylate cyclase B (atrionatriuretic peptide receptor B)(NPR2)	GO:0006182~cGMP biosynthetic process,GO:0007168~receptor guanylyl cyclase signaling pathway,GO:0035556~intracellular signal transduction,GO:0044702~single organism reproductive process,GO:0051447~negative regulation of meiotic cell cycle,GO:0060348~bone development,GO:0097011~cellular response to granulocyte macrophage colony-stimulating factor stimulus,GO:1900194~negative regulation of oocyte maturation,
**DistUt**	**DOWN**	HOXA10	homeobox A10(HOXA10)	GO:0006355~regulation of transcription, DNA-templated,
**DistUt**	**DOWN**	HOXA10	homeobox A10(HOXA10)	GO:0006355~regulation of transcription, DNA-templated,
**DistUt**	**DOWN**	VGLL3	vestigial like family member 3(VGLL3)	GO:0006355~regulation of transcription, DNA-templated,
**DistUt**	**DOWN**	MAGED1	MAGE family member D1(MAGED1)	GO:0006357~regulation of transcription from RNA polymerase II promoter,GO:0032922~circadian regulation of gene expression,GO:0042752~regulation of circadian rhythm,GO:0042981~regulation of apoptotic process,GO:0043406~positive regulation of MAP kinase activity,GO:0045892~negative regulation of transcription, DNA-templated,GO:0045893~positive regulation of transcription, DNA-templated,GO:0050680~negative regulation of epithelial cell proliferation,GO:0090190~positive regulation of branching involved in ureteric bud morphogenesis,GO:2001235~positive regulation of apoptotic signaling pathway,
**DistUt**	**DOWN**	ZNF516	zinc finger protein 516(ZNF516)	GO:0006366~transcription from RNA polymerase II promoter,GO:0007165~signal transduction,GO:0007275~multicellular organism development,GO:0009409~response to cold,GO:0045893~positive regulation of transcription, DNA-templated,GO:0050873~brown fat cell differentiation,GO:0060612~adipose tissue development,
**DistUt**	**DOWN**	ELL2	elongation factor for RNA polymerase II 2(ELL2)	GO:0006368~transcription elongation from RNA polymerase II promoter,GO:0042795~snRNA transcription from RNA polymerase II promoter,
**DistUt**	**DOWN**	PDIA3	protein disulfide isomerase family A member 3(PDIA3)	GO:0006457~protein folding,GO:0034976~response to endoplasmic reticulum stress,GO:0045454~cell redox homeostasis,GO:2001238~positive regulation of extrinsic apoptotic signaling pathway,
**DistUt**	**DOWN**	ERO1A	endoplasmic reticulum oxidoreductase 1 alpha(ERO1A)	GO:0006464~cellular protein modification process,GO:0019471~4-hydroxyproline metabolic process,GO:0022417~protein maturation by protein folding,GO:0030198~extracellular matrix organization,GO:0030968~endoplasmic reticulum unfolded protein response,GO:0034975~protein folding in endoplasmic reticulum,GO:0034976~response to endoplasmic reticulum stress,GO:0045454~cell redox homeostasis,GO:0050873~brown fat cell differentiation,GO:0051085~chaperone mediated protein folding requiring cofactor,GO:0051209~release of sequestered calcium ion into cytosol,GO:0055114~oxidation-reduction process,GO:0070059~intrinsic apoptotic signaling pathway in response to endoplasmic reticulum stress,
**DistUt**	**DOWN**	DCN	decorin(DCN)	GO:0006469~negative regulation of protein kinase activity,GO:0010596~negative regulation of endothelial cell migration,GO:0014068~positive regulation of phosphatidylinositol 3-kinase signaling,GO:0016239~positive regulation of macroautophagy,GO:0016525~negative regulation of angiogenesis,GO:0019221~cytokine-mediated signaling pathway,GO:0019800~peptide cross-linking via chondroitin 4-sulfate glycosaminoglycan,GO:0045944~positive regulation of transcription from RNA polymerase II promoter,GO:0046426~negative regulation of JAK-STAT cascade,GO:0051901~positive regulation of mitochondrial depolarization,GO:0090141~positive regulation of mitochondrial fission,GO:1900747~negative regulation of vascular endothelial growth factor signaling pathway,
**DistUt**	**DOWN**	NCEH1	neutral cholesterol ester hydrolase 1(NCEH1)	GO:0006470~protein dephosphorylation,GO:0006805~xenobiotic metabolic process,GO:0009056~catabolic process,GO:0060395~SMAD protein signal transduction,
**DistUt**	**DOWN**	ARF4	ADP ribosylation factor 4(ARF4)	GO:0006471~protein ADP-ribosylation,GO:0006890~retrograde vesicle-mediated transport, Golgi to ER,GO:0007264~small GTPase mediated signal transduction,GO:0007612~learning,GO:0016477~cell migration,GO:0031584~activation of phospholipase D activity,GO:0043066~negative regulation of apoptotic process,GO:0045176~apical protein localization,GO:0045197~establishment or maintenance of epithelial cell apical/basal polarity,GO:0045944~positive regulation of transcription from RNA polymerase II promoter,GO:0060996~dendritic spine development,GO:0061512~protein localization to cilium,GO:2000377~regulation of reactive oxygen species metabolic process,
**DistUt**	**DOWN**	B3GALT2	beta-1,3-galactosyltransferase 2(B3GALT2)	GO:0006486~protein glycosylation,GO:0009312~oligosaccharide biosynthetic process,
**DistUt**	**DOWN**	EDEM3	ER degradation enhancing alpha-mannosidase like protein 3(EDEM3)	GO:0006491~N-glycan processing,GO:0008152~metabolic process,GO:0030433~ER-associated ubiquitin-dependent protein catabolic process,GO:0030968~endoplasmic reticulum unfolded protein response,GO:0097466~glycoprotein ERAD pathway,GO:1904382~mannose trimming involved in glycoprotein ERAD pathway,
**DistUt**	**DOWN**	MMP1	matrix metallopeptidase 1(MMP1)	GO:0006508~proteolysis,GO:0030574~collagen catabolic process,GO:0032461~positive regulation of protein oligomerization,
**DistUt**	**DOWN**	GCLM	glutamate-cysteine ligase modifier subunit(GCLM)	GO:0006534~cysteine metabolic process,GO:0006536~glutamate metabolic process,GO:0006750~glutathione biosynthetic process,GO:0006979~response to oxidative stress,GO:0008637~apoptotic mitochondrial changes,GO:0035229~positive regulation of glutamate-cysteine ligase activity,GO:0042493~response to drug,GO:0050880~regulation of blood vessel size,GO:0051900~regulation of mitochondrial depolarization,GO:2001237~negative regulation of extrinsic apoptotic signaling pathway,

**DistUt**	**DOWN**	KDELR3	KDEL endoplasmic reticulum protein retention receptor 3(KDELR3)	GO:0006621~protein retention in ER lumen,GO:0015031~protein transport,
**DistUt**	**DOWN**	HSD17B11	hydroxysteroid 17-beta dehydrogenase 11(HSD17B11)	GO:0006710~androgen catabolic process,
**DistUt**	**DOWN**	SLCO2A1	solute carrier organic anion transporter family, member 2A1(SLCO2A1)	GO:0006811~ion transport,
**DistUt**	**DOWN**	SLC39A7	solute carrier family 39 member 7(SLC39A7)	GO:0006829~zinc II ion transport,GO:0055085~transmembrane transport,
**DistUt**	**DOWN**	SLC38A1	solute carrier family 38 member 1(SLC38A1)	GO:0006867~asparagine transport,GO:0006868~glutamine transport,GO:0015817~histidine transport,
**DistUt**	**DOWN**	SEC23A	Sec23 homolog A, coat complex II component(SEC23A)	GO:0006886~intracellular protein transport,GO:0006888~ER to Golgi vesicle-mediated transport,
**DistUt**	**DOWN**	SEC23B	Sec23 homolog B, coat complex II component(SEC23B)	GO:0006886~intracellular protein transport,GO:0006888~ER to Golgi vesicle-mediated transport,
**DistUt**	**DOWN**	EXOC5	exocyst complex component 5(EXOC5)	GO:0006887~exocytosis,GO:0042384~cilium assembly,GO:0048278~vesicle docking,
**DistUt**	**DOWN**	HTR2A	5-hydroxytryptamine receptor 2A(HTR2A)	GO:0006939~smooth muscle contraction,GO:0007193~adenylate cyclase-inhibiting G-protein coupled receptor signaling pathway,GO:0007200~phospholipase C-activating G-protein coupled receptor signaling pathway,GO:0007202~activation of phospholipase C activity,GO:0007210~serotonin receptor signaling pathway,GO:0007268~chemical synaptic transmission,GO:0007610~behavior,GO:0010513~positive regulation of phosphatidylinositol biosynthetic process,GO:0014065~phosphatidylinositol 3-kinase signaling,GO:0033674~positive regulation of kinase activity,GO:0042493~response to drug,GO:0044380~protein localization to cytoskeleton,GO:0045600~positive regulation of fat cell differentiation,GO:0045821~positive regulation of glycolytic process,GO:0046883~regulation of hormone secretion,GO:0050731~positive regulation of peptidyl-tyrosine phosphorylation,GO:0050795~regulation of behavior,GO:0051209~release of sequestered calcium ion into cytosol,GO:0070374~positive regulation of ERK1 and ERK2 cascade,
**DistUt**	**DOWN**	PTGFR	prostaglandin F receptor(PTGFR)	GO:0006954~inflammatory response,GO:0007189~adenylate cyclase-activating G-protein coupled receptor signaling pathway,GO:0007204~positive regulation of cytosolic calcium ion concentration,GO:0008284~positive regulation of cell proliferation,GO:0010628~positive regulation of gene expression,GO:0032355~response to estradiol,GO:0032496~response to lipopolysaccharide,GO:0035584~calcium-mediated signaling using intracellular calcium source,GO:0043066~negative regulation of apoptotic process,GO:0071799~cellular response to prostaglandin D stimulus,
**DistUt**	**DOWN**	HRH1	histamine receptor H1(HRH1)	GO:0006954~inflammatory response,GO:0007200~phospholipase C-activating G-protein coupled receptor signaling pathway,GO:0007613~memory,GO:0008542~visual learning,GO:0019229~regulation of vasoconstriction,GO:0043114~regulation of vascular permeability,GO:0045907~positive regulation of vasoconstriction,GO:0048167~regulation of synaptic plasticity,GO:0048245~eosinophil chemotaxis,GO:0050804~modulation of synaptic transmission,
**DistUt**	**DOWN**	RPS6KA6	ribosomal protein S6 kinase A6(RPS6KA6)	GO:0006978~DNA damage response, signal transduction by p53 class mediator resulting in transcription of p21 class mediator,GO:0035556~intracellular signal transduction,
**DistUt**	**DOWN**	GPX8	glutathione peroxidase 8 (putative)(GPX8)	GO:0006979~response to oxidative stress,
**DistUt**	**DOWN**	KIFAP3	kinesin associated protein 3(KIFAP3)	GO:0007017~microtubule-based process,GO:0008104~protein localization,GO:0008285~negative regulation of cell proliferation,GO:0043066~negative regulation of apoptotic process,GO:0046587~positive regulation of calcium-dependent cell-cell adhesion,
**DistUt**	**DOWN**	ATP8B2	ATPase phospholipid transporting 8B2(ATP8B2)	GO:0007030~Golgi organization,
**DistUt**	**DOWN**	CLDN11	claudin 11(CLDN11)	GO:0007155~cell adhesion,GO:0007283~spermatogenesis,GO:0008366~axon ensheathment,
**DistUt**	**DOWN**	LOC100520265	protocadherin-9(LOC100520265)	GO:0007156~homophilic cell adhesion via plasma membrane adhesion molecules,
**DistUt**	**DOWN**	CDH11	cadherin 11(CDH11)	GO:0007156~homophilic cell adhesion via plasma membrane adhesion molecules,GO:0021957~corticospinal tract morphogenesis,
**DistUt**	**DOWN**	ALCAM	activated leukocyte cell adhesion molecule(ALCAM)	GO:0007157~heterophilic cell-cell adhesion via plasma membrane cell adhesion molecules,GO:0008045~motor neuron axon guidance,
**DistUt**	**DOWN**	COL3A1	collagen type III alpha 1 chain(COL3A1)	GO:0007160~cell-matrix adhesion,GO:0007179~transforming growth factor beta receptor signaling pathway,GO:0007229~integrin-mediated signaling pathway,GO:0007507~heart development,GO:0009314~response to radiation,GO:0018149~peptide cross-linking,GO:0021987~cerebral cortex development,GO:0030199~collagen fibril organization,GO:0034097~response to cytokine,GO:0035025~positive regulation of Rho protein signal transduction,GO:0042060~wound healing,GO:0043206~extracellular fibril organization,GO:0043588~skin development,GO:0048565~digestive tract development,GO:0050777~negative regulation of immune response,GO:0060414~aorta smooth muscle tissue morphogenesis,GO:0071230~cellular response to amino acid stimulus,GO:2001223~negative regulation of neuron migration,
**DistUt**	**DOWN**	ARHGAP28	Rho GTPase activating protein 28(ARHGAP28)	GO:0007165~signal transduction,
**DistUt**	**DOWN**	GUCY1B3	guanylate cyclase 1, soluble, beta 3(GUCY1B3)	GO:0007165~signal transduction,GO:0035556~intracellular signal transduction,
**DistUt**	**DOWN**	SPARC	secreted protein, acidic, cysteine-rich (osteonectin)(SPARC)	GO:0007165~signal transduction,GO:0048856~anatomical structure development,
**DistUt**	**DOWN**	TSPAN9	tetraspanin 9(TSPAN9)	GO:0007166~cell surface receptor signaling pathway,
**DistUt**	**DOWN**	LOC100157704	olfactory receptor 1L8-like(LOC100157704)	GO:0007186~G-protein coupled receptor signaling pathway,GO:0050907~detection of chemical stimulus involved in sensory perception,
**DistUt**	**DOWN**	GNAI1	G protein subunit alpha i1(GNAI1)	GO:0007188~adenylate cyclase-modulating G-protein coupled receptor signaling pathway,GO:0051301~cell division,
**DistUt**	**DOWN**	TAC3	tachykinin 3(TAC3)	GO:0007217~tachykinin receptor signaling pathway,GO:0007218~neuropeptide signaling pathway,
**DistUt**	**DOWN**	RASL12	RAS like family 12(RASL12)	GO:0007264~small GTPase mediated signal transduction,
**DistUt**	**DOWN**	RND3	Rho family GTPase 3(RND3)	GO:0007264~small GTPase mediated signal transduction,
**DistUt**	**DOWN**	ABHD2	abhydrolase domain containing 2(ABHD2)	GO:0007340~acrosome reaction,GO:0009611~response to wounding,GO:0030336~negative regulation of cell migration,GO:0032570~response to progesterone,GO:0046464~acylglycerol catabolic process,GO:0048240~sperm capacitation,
**DistUt**	**DOWN**	BICC1	BicC family RNA binding protein 1(BICC1)	GO:0007368~determination of left/right symmetry,GO:0007507~heart development,GO:0090090~negative regulation of canonical Wnt signaling pathway,
**DistUt**	**DOWN**	CFL2	cofilin 2(CFL2)	GO:0007519~skeletal muscle tissue development,GO:0030042~actin filament depolymerization,GO:0030043~actin filament fragmentation,GO:0030836~positive regulation of actin filament depolymerization,GO:0045214~sarcomere organization,GO:0046716~muscle cell cellular homeostasis,
**DistUt**	**DOWN**	MORC3	MORC family CW-type zinc finger 3(MORC3)	GO:0007569~cell aging,GO:0009791~post-embryonic development,GO:0018105~peptidyl-serine phosphorylation,GO:0048147~negative regulation of fibroblast proliferation,GO:0050821~protein stabilization,GO:0051457~maintenance of protein location in nucleus,
**DistUt**	**DOWN**	AFF2	AF4/FMR2 family member 2(AFF2)	GO:0007611~learning or memory,GO:0043484~regulation of RNA splicing,
**DistUt**	**DOWN**	EPHA4	EPH receptor A4(EPHA4)	GO:0007628~adult walking behavior,GO:0008045~motor neuron axon guidance,GO:0018108~peptidyl-tyrosine phosphorylation,GO:0021957~corticospinal tract morphogenesis,GO:0043507~positive regulation of JUN kinase activity,GO:0046777~protein autophosphorylation,GO:0048681~negative regulation of axon regeneration,GO:0048710~regulation of astrocyte differentiation,GO:0050770~regulation of axonogenesis,GO:0061001~regulation of dendritic spine morphogenesis,GO:0072178~nephric duct morphogenesis,GO:0097155~fasciculation of sensory neuron axon,GO:0097156~fasciculation of motor neuron axon,GO:2001108~positive regulation of Rho guanyl-nucleotide exchange factor activity,
**DistUt**	**DOWN**	PELO	pelota homolog (Drosophila)(PELO)	GO:0008283~cell proliferation,GO:0051276~chromosome organization,GO:0070481~nuclear-transcribed mRNA catabolic process, non-stop decay,GO:0070966~nuclear-transcribed mRNA catabolic process, no-go decay,GO:0071025~RNA surveillance,
**DistUt**	**DOWN**	CD248	CD248 molecule(CD248)	GO:0008284~positive regulation of cell proliferation,GO:0016477~cell migration,GO:0048535~lymph node development,GO:0060033~anatomical structure regression,GO:2000353~positive regulation of endothelial cell apoptotic process,

**DistUt**	**DOWN**	FRZB	frizzled-related protein(FRZB)	GO:0008285~negative regulation of cell proliferation,GO:0010721~negative regulation of cell development,GO:0014033~neural crest cell differentiation,GO:0030308~negative regulation of cell growth,GO:0035567~non-canonical Wnt signaling pathway,GO:0043065~positive regulation of apoptotic process,GO:0045600~positive regulation of fat cell differentiation,GO:0060029~convergent extension involved in organogenesis,GO:0060070~canonical Wnt signaling pathway,GO:0061037~negative regulation of cartilage development,GO:0061053~somite development,GO:0070367~negative regulation of hepatocyte differentiation,GO:0090090~negative regulation of canonical Wnt signaling pathway,GO:0090103~cochlea morphogenesis,
**DistUt**	**DOWN**	ADAM22	ADAM metallopeptidase domain 22(ADAM22)	GO:0008344~adult locomotory behavior,GO:0022011~myelination in peripheral nervous system,
**DistUt**	**DOWN**	FGF2	fibroblast growth factor 2(FGF2)	GO:0008543~fibroblast growth factor receptor signaling pathway,
**DistUt**	**DOWN**	TVP23B	trans-golgi network vesicle protein 23 homolog B(TVP23B)	GO:0009306~protein secretion,GO:0016192~vesicle-mediated transport,
**DistUt**	**DOWN**	SLC30A7	solute carrier family 30 member 7(SLC30A7)	GO:0010043~response to zinc ion,GO:0032119~sequestering of zinc ion,GO:0061088~regulation of sequestering of zinc ion,
**DistUt**	**DOWN**	AGR2	anterior gradient 2, protein disulphide isomerase family member(AGR2)	GO:0010628~positive regulation of gene expression,GO:0010811~positive regulation of cell-substrate adhesion,GO:0030154~cell differentiation,GO:0045742~positive regulation of epidermal growth factor receptor signaling pathway,GO:0048546~digestive tract morphogenesis,GO:0048639~positive regulation of developmental growth,GO:0060480~lung goblet cell differentiation,GO:0060548~negative regulation of cell death,GO:0070254~mucus secretion,GO:0090004~positive regulation of establishment of protein localization to plasma membrane,GO:1903896~positive regulation of IRE1-mediated unfolded protein response,GO:1903899~positive regulation of PERK-mediated unfolded protein response,
**DistUt**	**DOWN**	FAP	fibroblast activation protein alpha(FAP)	GO:0010710~regulation of collagen catabolic process,GO:0010716~negative regulation of extracellular matrix disassembly,GO:0043542~endothelial cell migration,GO:0051603~proteolysis involved in cellular protein catabolic process,GO:0060244~negative regulation of cell proliferation involved in contact inhibition,GO:0071158~positive regulation of cell cycle arrest,GO:0071850~mitotic cell cycle arrest,GO:0097325~melanocyte proliferation,GO:1900119~positive regulation of execution phase of apoptosis,GO:1902362~melanocyte apoptotic process,
**DistUt**	**DOWN**	MMP3	matrix metallopeptidase 3 (stromelysin 1, progelatinase)(MMP3)	GO:0010727~negative regulation of hydrogen peroxide metabolic process,GO:0032461~positive regulation of protein oligomerization,
**DistUt**	**DOWN**	IFRD1	interferon related developmental regulator 1(IFRD1)	GO:0014706~striated muscle tissue development,GO:0030517~negative regulation of axon extension,GO:0042692~muscle cell differentiation,GO:0043403~skeletal muscle tissue regeneration,GO:0048671~negative regulation of collateral sprouting,
**DistUt**	**DOWN**	STEAP2	STEAP2 metalloreductase(STEAP2)	GO:0015677~copper ion import,GO:0097461~ferric iron import into cell,
**DistUt**	**DOWN**	CLINT1	clathrin interactor 1(CLINT1)	GO:0016192~vesicle-mediated transport,
**DistUt**	**DOWN**	SDC2	syndecan 2(SDC2)	GO:0016477~cell migration,GO:0048813~dendrite morphogenesis,GO:0048814~regulation of dendrite morphogenesis,
**DistUt**	**DOWN**	FBXL5	F-box and leucine rich repeat protein 5(FBXL5)	GO:0016567~protein ubiquitination,GO:0031146~SCF-dependent proteasomal ubiquitin-dependent protein catabolic process,GO:0055072~iron ion homeostasis,GO:1903364~positive regulation of cellular protein catabolic process,
**DistUt**	**DOWN**	TGM2	transglutaminase 2(TGM2)	GO:0018149~peptide cross-linking,GO:0032471~negative regulation of endoplasmic reticulum calcium ion concentration,GO:0043065~positive regulation of apoptotic process,GO:0043277~apoptotic cell clearance,GO:0045785~positive regulation of cell adhesion,GO:0051561~positive regulation of mitochondrial calcium ion concentration,GO:0060445~branching involved in salivary gland morphogenesis,GO:0060662~salivary gland cavitation,
**DistUt**	**DOWN**	RCAN2	regulator of calcineurin 2(RCAN2)	GO:0019722~calcium-mediated signaling,GO:0070884~regulation of calcineurin-NFAT signaling cascade,
**DistUt**	**DOWN**	CRISPLD2	cysteine rich secretory protein LCCL domain containing 2(CRISPLD2)	GO:0030198~extracellular matrix organization,GO:0060325~face morphogenesis,
**DistUt**	**DOWN**	HSD11B1	hydroxysteroid 11-beta dehydrogenase 1(HSD11B1)	GO:0030324~lung development,
**DistUt**	**DOWN**	DNAJB9	DnaJ heat shock protein family (Hsp40) member B9(DNAJB9)	GO:0030433~ER-associated ubiquitin-dependent protein catabolic process,
**DistUt**	**DOWN**	ERLEC1	endoplasmic reticulum lectin 1(ERLEC1)	GO:0030433~ER-associated ubiquitin-dependent protein catabolic process,GO:0030970~retrograde protein transport, ER to cytosol,GO:1904153~negative regulation of retrograde protein transport, ER to cytosol,
**DistUt**	**DOWN**	OMD	osteomodulin(OMD)	GO:0030500~regulation of bone mineralization,
**DistUt**	**DOWN**	PPP4R4	protein phosphatase 4 regulatory subunit 4(PPP4R4)	GO:0032515~negative regulation of phosphoprotein phosphatase activity,GO:0080163~regulation of protein serine/threonine phosphatase activity,
**DistUt**	**DOWN**	ARMCX3	armadillo repeat containing, X-linked 3(ARMCX3)	GO:0034613~cellular protein localization,GO:0045944~positive regulation of transcription from RNA polymerase II promoter,
**DistUt**	**DOWN**	PLEKHA8	pleckstrin homology domain containing A8(PLEKHA8)	GO:0035621~ER to Golgi ceramide transport,
**DistUt**	**DOWN**	PDP1	pyruvate dehyrogenase phosphatase catalytic subunit 1(PDP1)	GO:0035970~peptidyl-threonine dephosphorylation,
**DistUt**	**DOWN**	ADAMTS5	ADAM metallopeptidase with thrombospondin type 1 motif 5(ADAMTS5)	GO:0042742~defense response to bacterium,
**DistUt**	**DOWN**	RNF11	ring finger protein 11(RNF11)	GO:0042787~protein ubiquitination involved in ubiquitin-dependent protein catabolic process,GO:0051865~protein autoubiquitination,
**DistUt**	**DOWN**	BRINP2	BMP/retinoic acid inducible neural specific 2(BRINP2)	GO:0045666~positive regulation of neuron differentiation,GO:0045930~negative regulation of mitotic cell cycle,GO:0071300~cellular response to retinoic acid,
**DistUt**	**DOWN**	FAM213A	family with sequence similarity 213 member A(FAM213A)	GO:0045670~regulation of osteoclast differentiation,GO:0055114~oxidation-reduction process,
**DistUt**	**DOWN**	MFAP2	microfibrillar associated protein 2(MFAP2)	GO:0048048~embryonic eye morphogenesis,GO:0048050~post-embryonic eye morphogenesis,
**DistUt**	**DOWN**	LOC100738373	epidermal growth factor receptor kinase substrate 8-like(LOC100738373)	GO:0051016~barbed-end actin filament capping,GO:0051017~actin filament bundle assembly,
**DistUt**	**DOWN**	KCTD9	potassium channel tetramerization domain containing 9(KCTD9)	GO:0051260~protein homooligomerization,
**DistUt**	**DOWN**	SLC41A2	solute carrier family 41 member 2(SLC41A2)	GO:0055085~transmembrane transport,GO:0070838~divalent metal ion transport,
**DistUt**	**DOWN**	FKBP7	FK506 binding protein 7(FKBP7)	GO:0061077~chaperone-mediated protein folding,
**DistUt**	**DOWN**	LOC100517015	cAMP-dependent protein kinase inhibitor beta-like(LOC100517015)	GO:2000480~negative regulation of cAMP-dependent protein kinase activity,
**ProxUt**	**UP**	CALM1	calmodulin 1(CALM1)	GO:0000086~G2/M transition of mitotic cell cycle,GO:0002027~regulation of heart rate,GO:0005513~detection of calcium ion,GO:0010880~regulation of release of sequestered calcium ion into cytosol by sarcoplasmic reticulum,GO:0021762~substantia nigra development,GO:0030801~positive regulation of cyclic nucleotide metabolic process,GO:0032465~regulation of cytokinesis,GO:0032516~positive regulation of phosphoprotein phosphatase activity,GO:0043388~positive regulation of DNA binding,GO:0051343~positive regulation of cyclic-nucleotide phosphodiesterase activity,GO:0055117~regulation of cardiac muscle contraction,GO:0060316~positive regulation of ryanodine-sensitive calcium-release channel activity,
**ProxUt**	**UP**	JAZF1	JAZF zinc finger 1(JAZF1)	GO:0000122~negative regulation of transcription from RNA polymerase II promoter,

**ProxUt**	**UP**	RREB1	ras responsive element binding protein 1(RREB1)	GO:0000122~negative regulation of transcription from RNA polymerase II promoter,GO:0006355~regulation of transcription, DNA-templated,GO:0006366~transcription from RNA polymerase II promoter,GO:0007165~signal transduction,GO:0007275~multicellular organism development,GO:0010634~positive regulation of epithelial cell migration,GO:0033601~positive regulation of mammary gland epithelial cell proliferation,GO:1900026~positive regulation of substrate adhesion-dependent cell spreading,GO:1903691~positive regulation of wound healing, spreading of epidermal cells,GO:2000394~positive regulation of lamellipodium morphogenesis,
**ProxUt**	**UP**	MAPT	microtubule associated protein tau(MAPT)	GO:0000226~microtubule cytoskeleton organization,GO:0001764~neuron migration,GO:0007628~adult walking behavior,GO:0008088~axo-dendritic transport,GO:0010506~regulation of autophagy,GO:0031116~positive regulation of microtubule polymerization,GO:0031175~neuron projection development,GO:0032387~negative regulation of intracellular transport,GO:0045773~positive regulation of axon extension,GO:0047497~mitochondrion transport along microtubule,GO:0048675~axon extension,GO:0060632~regulation of microtubule-based movement,
**ProxUt**	**UP**	XAB2	XPA binding protein 2(XAB2)	GO:0000349~generation of catalytic spliceosome for first transesterification step,GO:0001824~blastocyst development,GO:0006283~transcription-coupled nucleotide-excision repair,GO:0006351~transcription, DNA-templated,
**ProxUt**	**UP**	CELF4	CUGBP, Elav-like family member 4(CELF4)	GO:0000380~alternative mRNA splicing, via spliceosome,GO:0000381~regulation of alternative mRNA splicing, via spliceosome,GO:0006376~mRNA splice site selection,GO:0048025~negative regulation of mRNA splicing, via spliceosome,GO:0048026~positive regulation of mRNA splicing, via spliceosome,GO:0090394~negative regulation of excitatory postsynaptic potential,GO:1902866~regulation of retina development in camera-type eye,
**ProxUt**	**UP**	HOXD12	homeobox D12(HOXD12)	GO:0001501~skeletal system development,GO:0006355~regulation of transcription, DNA-templated,GO:0007389~pattern specification process,GO:0042733~embryonic digit morphogenesis,
**ProxUt**	**UP**	SP7	Sp7 transcription factor(SP7)	GO:0001649~osteoblast differentiation,GO:0045944~positive regulation of transcription from RNA polymerase II promoter,GO:0060218~hematopoietic stem cell differentiation,GO:2000738~positive regulation of stem cell differentiation,
**ProxUt**	**UP**	DEAF1	DEAF1, transcription factor(DEAF1)	GO:0001662~behavioral fear response,GO:0001843~neural tube closure,GO:0006357~regulation of transcription from RNA polymerase II promoter,GO:0006366~transcription from RNA polymerase II promoter,GO:0008542~visual learning,GO:0033599~regulation of mammary gland epithelial cell proliferation,GO:0045892~negative regulation of transcription, DNA-templated,GO:0045893~positive regulation of transcription, DNA-templated,GO:0048706~embryonic skeletal system development,GO:2000026~regulation of multicellular organismal development,
**ProxUt**	**UP**	ADRA2A	adrenoceptor alpha 2A(ADRA2A)	GO:0001819~positive regulation of cytokine production,GO:0006940~regulation of smooth muscle contraction,GO:0007194~negative regulation of adenylate cyclase activity,GO:0007267~cell-cell signaling,GO:0019229~regulation of vasoconstriction,GO:0030168~platelet activation,GO:0030335~positive regulation of cell migration,GO:0030818~negative regulation of cAMP biosynthetic process,GO:0032148~activation of protein kinase B activity,GO:0032870~cellular response to hormone stimulus,GO:0035625~epidermal growth factor-activated receptor transactivation by G-protein coupled receptor signaling pathway,GO:0042593~glucose homeostasis,GO:0043268~positive regulation of potassium ion transport,GO:0050995~negative regulation of lipid catabolic process,GO:0051044~positive regulation of membrane protein ectodomain proteolysis,GO:0051926~negative regulation of calcium ion transport,GO:0061179~negative regulation of insulin secretion involved in cellular response to glucose stimulus,GO:0071878~negative regulation of adrenergic receptor signaling pathway,GO:0071880~adenylate cyclase-activating adrenergic receptor signaling pathway,GO:0071881~adenylate cyclase-inhibiting adrenergic receptor signaling pathway,GO:0071882~phospholipase C-activating adrenergic receptor signaling pathway,GO:0071883~activation of MAPK activity by adrenergic receptor signaling pathway,GO:0090303~positive regulation of wound healing,GO:1901020~negative regulation of calcium ion transmembrane transporter activity,
**ProxUt**	**UP**	HSF1	heat shock transcription factor 1(HSF1)	GO:0001892~embryonic placenta development,GO:0006468~protein phosphorylation,GO:0007143~female meiotic division,GO:0007283~spermatogenesis,GO:0008285~negative regulation of cell proliferation,GO:0009299~mRNA transcription,GO:0032496~response to lipopolysaccharide,GO:0032720~negative regulation of tumor necrosis factor production,GO:0034605~cellular response to heat,GO:0040018~positive regulation of multicellular organism growth,GO:0045944~positive regulation of transcription from RNA polymerase II promoter,GO:0060136~embryonic process involved in female pregnancy,
**ProxUt**	**UP**	STK11	serine/threonine kinase 11(STK11)	GO:0001894~tissue homeostasis,GO:0001944~vasculature development,GO:0006468~protein phosphorylation,GO:0006974~cellular response to DNA damage stimulus,GO:0007050~cell cycle arrest,GO:0007286~spermatid development,GO:0007409~axonogenesis,GO:0010212~response to ionizing radiation,GO:0010508~positive regulation of autophagy,GO:0030010~establishment of cell polarity,GO:0030111~regulation of Wnt signaling pathway,GO:0030308~negative regulation of cell growth,GO:0030511~positive regulation of transforming growth factor beta receptor signaling pathway,GO:0036399~TCR signalosome assembly,GO:0042593~glucose homeostasis,GO:0043276~anoikis,GO:0045059~positive thymic T cell selection,GO:0046777~protein autophosphorylation,GO:0048814~regulation of dendrite morphogenesis,GO:0050731~positive regulation of peptidyl-tyrosine phosphorylation,GO:0050772~positive regulation of axonogenesis,GO:0050852~T cell receptor signaling pathway,GO:0051055~negative regulation of lipid biosynthetic process,GO:0051645~Golgi localization,GO:0051896~regulation of protein kinase B signaling,GO:0060070~canonical Wnt signaling pathway,GO:0060770~negative regulation of epithelial cell proliferation involved in prostate gland development,GO:0071493~cellular response to UV-B,GO:0071902~positive regulation of protein serine/threonine kinase activity,GO:0072332~intrinsic apoptotic signaling pathway by p53 class mediator,GO:0097484~dendrite extension,GO:1900182~positive regulation of protein localization to nucleus,GO:1904262~negative regulation of TORC1 signaling,GO:2000774~positive regulation of cellular senescence,
**ProxUt**	**UP**	ADRA1A	adrenoceptor alpha 1A(ADRA1A)	GO:0001985~negative regulation of heart rate involved in baroreceptor response to increased systemic arterial blood pressure,GO:0001994~norepinephrine-epinephrine vasoconstriction involved in regulation of systemic arterial blood pressure,GO:0001996~positive regulation of heart rate by epinephrine-norepinephrine,GO:0001997~positive regulation of the force of heart contraction by epinephrine-norepinephrine,GO:0007200~phospholipase C-activating G-protein coupled receptor signaling pathway,GO:0007204~positive regulation of cytosolic calcium ion concentration,GO:0007267~cell-cell signaling,GO:0007512~adult heart development,GO:0016049~cell growth,GO:0035265~organ growth,GO:0043410~positive regulation of MAPK cascade,GO:0045907~positive regulation of vasoconstriction,GO:0045987~positive regulation of smooth muscle contraction,GO:0055117~regulation of cardiac muscle contraction,GO:0071880~adenylate cyclase-activating adrenergic receptor signaling pathway,GO:0097195~pilomotor reflex,
**ProxUt**	**UP**	LIM2	lens intrinsic membrane protein 2(LIM2)	GO:0002088~lens development in camera-type eye,
**ProxUt**	**UP**	CHST4	carbohydrate sulfotransferase 4(CHST4)	GO:0005975~carbohydrate metabolic process,GO:0006044~N-acetylglucosamine metabolic process,GO:0006477~protein sulfation,
**ProxUt**	**UP**	ALDH2	aldehyde dehydrogenase 2 family (mitochondrial)(ALDH2)	GO:0006068~ethanol catabolic process,GO:0055114~oxidation-reduction process,
**ProxUt**	**UP**	DDB1	damage specific DNA binding protein 1(DDB1)	GO:0006289~nucleotide-excision repair,GO:0016055~Wnt signaling pathway,GO:0035518~histone H2A monoubiquitination,GO:0042787~protein ubiquitination involved in ubiquitin-dependent protein catabolic process,GO:0043066~negative regulation of apoptotic process,GO:0043161~proteasome-mediated ubiquitin-dependent protein catabolic process,GO:0045070~positive regulation of viral genome replication,GO:0046726~positive regulation by virus of viral protein levels in host cell,GO:0051702~interaction with symbiont,GO:0070914~UV-damage excision repair,GO:1901990~regulation of mitotic cell cycle phase transition,GO:1902188~positive regulation of viral release from host cell,
**ProxUt**	**UP**	MLXIP	MLX interacting protein(MLXIP)	GO:0006357~regulation of transcription from RNA polymerase II promoter,GO:1900402~regulation of carbohydrate metabolic process by regulation of transcription from RNA polymerase II promoter,
**ProxUt**	**UP**	RTN4RL1	reticulon 4 receptor like 1(RTN4RL1)	GO:0006469~negative regulation of protein kinase activity,GO:0019221~cytokine-mediated signaling pathway,GO:0046426~negative regulation of JAK-STAT cascade,
**ProxUt**	**UP**	CRYL1	crystallin lambda 1(CRYL1)	GO:0006631~fatty acid metabolic process,GO:0055114~oxidation-reduction process,
**ProxUt**	**UP**	SERINC5	serine incorporator 5(SERINC5)	GO:0006658~phosphatidylserine metabolic process,GO:0006665~sphingolipid metabolic process,GO:0042552~myelination,
**ProxUt**	**UP**	SPR	sepiapterin reductase (7,8-dihydrobiopterin:NADP+ oxidoreductase)(SPR)	GO:0006729~tetrahydrobiopterin biosynthetic process,GO:0006809~nitric oxide biosynthetic process,
**ProxUt**	**UP**	PCBD1	pterin-4 alpha-carbinolamine dehydratase 1(PCBD1)	GO:0006729~tetrahydrobiopterin biosynthetic process,GO:0051289~protein homotetramerization,GO:0051291~protein heterooligomerization,
**ProxUt**	**UP**	SLC35F1	solute carrier family 35 member F1(SLC35F1)	GO:0006810~transport,
**ProxUt**	**UP**	RHCG	Rh family C glycoprotein(RHCG)	GO:0006873~cellular ion homeostasis,GO:0006885~regulation of pH,GO:0015695~organic cation transport,GO:0015696~ammonium transport,GO:0019740~nitrogen utilization,GO:0070634~transepithelial ammonium transport,GO:0072488~ammonium transmembrane transport,

**ProxUt**	**UP**	XK	X-linked Kx blood group(XK)	GO:0006874~cellular calcium ion homeostasis,GO:0008361~regulation of cell size,GO:0010961~cellular magnesium ion homeostasis,GO:0031133~regulation of axon diameter,GO:0042552~myelination,GO:0048741~skeletal muscle fiber development,
**ProxUt**	**UP**	RNF152	ring finger protein 152(RNF152)	GO:0006915~apoptotic process,GO:0010508~positive regulation of autophagy,GO:0034198~cellular response to amino acid starvation,GO:0070534~protein K63-linked ubiquitination,GO:0070936~protein K48-linked ubiquitination,GO:1904262~negative regulation of TORC1 signaling,
**ProxUt**	**UP**	IL17D	interleukin 17D(IL17D)	GO:0006954~inflammatory response,GO:0007166~cell surface receptor signaling pathway,
**ProxUt**	**UP**	LOC100737805	SUN domain-containing protein 1-like(LOC100737805)	GO:0006998~nuclear envelope organization,GO:0090286~cytoskeletal anchoring at nuclear membrane,
**ProxUt**	**UP**	CHCHD10	coiled-coil-helix-coiled-coil-helix domain containing 10(CHCHD10)	GO:0007005~mitochondrion organization,
**ProxUt**	**UP**	TMSB4X	thymosin beta 4, X-linked(TMSB4X)	GO:0007015~actin filament organization,
**ProxUt**	**UP**	TMSB4X	thymosin beta 4, X-linked(TMSB4X)	GO:0007015~actin filament organization,GO:0030036~actin cytoskeleton organization,GO:0030334~regulation of cell migration,GO:0042989~sequestering of actin monomers,
**ProxUt**	**UP**	CNTROB	centrobin, centriole duplication and spindle assembly protein(CNTROB)	GO:0007099~centriole replication,GO:0051299~centrosome separation,GO:1902410~mitotic cytokinetic process,
**ProxUt**	**UP**	GRK1	G protein-coupled receptor kinase 1(GRK1)	GO:0007165~signal transduction,GO:0007601~visual perception,GO:0022400~regulation of rhodopsin mediated signaling pathway,GO:0046777~protein autophosphorylation,
**ProxUt**	**UP**	VIPR1	vasoactive intestinal peptide receptor 1(VIPR1)	GO:0007166~cell surface receptor signaling pathway,GO:0007186~G-protein coupled receptor signaling pathway,
**ProxUt**	**UP**	LOC100516511	olfactory receptor 1A1(LOC100516511)	GO:0007186~G-protein coupled receptor signaling pathway,
**ProxUt**	**UP**	GPR26	G protein-coupled receptor 26(GPR26)	GO:0007189~adenylate cyclase-activating G-protein coupled receptor signaling pathway,GO:0031647~regulation of protein stability,GO:1903955~positive regulation of protein targeting to mitochondrion,
**ProxUt**	**UP**	LOC100524975	olfactory receptor 10H1(LOC100524975)	GO:0007192~adenylate cyclase-activating serotonin receptor signaling pathway,GO:0007268~chemical synaptic transmission,
**ProxUt**	**UP**	RXFP4	relaxin/insulin like family peptide receptor 4(RXFP4)	GO:0007200~phospholipase C-activating G-protein coupled receptor signaling pathway,GO:0007218~neuropeptide signaling pathway,GO:2000253~positive regulation of feeding behavior,
**ProxUt**	**UP**	KCNQ4	potassium voltage-gated channel subfamily Q member 4(KCNQ4)	GO:0007605~sensory perception of sound,GO:0042472~inner ear morphogenesis,
**ProxUt**	**UP**	OTOGL	otogelin like(OTOGL)	GO:0007605~sensory perception of sound,GO:0046373~L-arabinose metabolic process,
**ProxUt**	**UP**	ASGR2	asialoglycoprotein receptor 2(ASGR2)	GO:0009100~glycoprotein metabolic process,GO:0031647~regulation of protein stability,GO:0055088~lipid homeostasis,
**ProxUt**	**UP**	LOC100739011	putative lipoyltransferase 2, mitochondrial(LOC100739011)	GO:0009107~lipoate biosynthetic process,GO:0009249~protein lipoylation,
**ProxUt**	**UP**	PGLYRP1	peptidoglycan recognition protein 1(PGLYRP1)	GO:0009253~peptidoglycan catabolic process,GO:0045087~innate immune response,
**ProxUt**	**UP**	ADHFE1	alcohol dehydrogenase, iron containing 1(ADHFE1)	GO:0015993~molecular hydrogen transport,
**ProxUt**	**UP**	RHBDL1	rhomboid like 1(RHBDL1)	GO:0016485~protein processing,
**ProxUt**	**UP**	GALK1	galactokinase 1(GALK1)	GO:0019402~galactitol metabolic process,GO:0061623~glycolytic process from galactose,
**ProxUt**	**UP**	DGAT1	diacylglycerol O-acyltransferase 1(DGAT1)	GO:0019432~triglyceride biosynthetic process,GO:0019915~lipid storage,GO:0034379~very-low-density lipoprotein particle assembly,GO:0035336~long-chain fatty-acyl-CoA metabolic process,GO:0046339~diacylglycerol metabolic process,GO:0055089~fatty acid homeostasis,
**ProxUt**	**UP**	PACSIN3	protein kinase C and casein kinase substrate in neurons 3(PACSIN3)	GO:0030100~regulation of endocytosis,GO:0097320~membrane tubulation,
**ProxUt**	**UP**	SDR16C5	short chain dehydrogenase/reductase family 16C, member 5(SDR16C5)	GO:0042572~retinol metabolic process,GO:0042574~retinal metabolic process,GO:0043616~keratinocyte proliferation,
**ProxUt**	**UP**	JADE3	jade family PHD finger 3(JADE3)	GO:0043966~histone H3 acetylation,GO:0043981~histone H4-K5 acetylation,GO:0043982~histone H4-K8 acetylation,GO:0043983~histone H4-K12 acetylation,
**ProxUt**	**UP**	MTMR7	myotubularin related protein 7(MTMR7)	GO:0046855~inositol phosphate dephosphorylation,GO:0046856~phosphatidylinositol dephosphorylation,
**ProxUt**	**UP**	CD207	CD207 molecule, langerin(CD207)	GO:0051607~defense response to virus,
**ProxUt**	**DOWN**	TCF7L2	transcription factor 7 like 2(TCF7L2)	GO:0000122~negative regulation of transcription from RNA polymerase II promoter,GO:0001568~blood vessel development,GO:0006357~regulation of transcription from RNA polymerase II promoter,GO:0007050~cell cycle arrest,GO:0008283~cell proliferation,GO:0010909~positive regulation of heparan sulfate proteoglycan biosynthetic process,GO:0032024~positive regulation of insulin secretion,GO:0032092~positive regulation of protein binding,GO:0032350~regulation of hormone metabolic process,GO:0042593~glucose homeostasis,GO:0043433~negative regulation of sequence-specific DNA binding transcription factor activity,GO:0043570~maintenance of DNA repeat elements,GO:0044334~canonical Wnt signaling pathway involved in positive regulation of epithelial to mesenchymal transition,GO:0045444~fat cell differentiation,GO:0045944~positive regulation of transcription from RNA polymerase II promoter,GO:0046827~positive regulation of protein export from nucleus,GO:0048625~myoblast fate commitment,GO:0048660~regulation of smooth muscle cell proliferation,GO:0051897~positive regulation of protein kinase B signaling,GO:0060070~canonical Wnt signaling pathway,GO:0090090~negative regulation of canonical Wnt signaling pathway,GO:2000675~negative regulation of type B pancreatic cell apoptotic process,GO:2001237~negative regulation of extrinsic apoptotic signaling pathway,
**ProxUt**	**DOWN**	RARA	retinoic acid receptor alpha(RARA)	GO:0000122~negative regulation of transcription from RNA polymerase II promoter,GO:0001657~ureteric bud development,GO:0001843~neural tube closure,GO:0002068~glandular epithelial cell development,GO:0003148~outflow tract septum morphogenesis,GO:0003417~growth plate cartilage development,GO:0006351~transcription, DNA-templated,GO:0006468~protein phosphorylation,GO:0007281~germ cell development,GO:0007283~spermatogenesis,GO:0008284~positive regulation of cell proliferation,GO:0030853~negative regulation of granulocyte differentiation,GO:0031076~embryonic camera-type eye development,GO:0032689~negative regulation of interferon-gamma production,GO:0032720~negative regulation of tumor necrosis factor production,GO:0032736~positive regulation of interleukin-13 production,GO:0032753~positive regulation of interleukin-4 production,GO:0032754~positive regulation of interleukin-5 production,GO:0035264~multicellular organism growth,GO:0043066~negative regulation of apoptotic process,GO:0043277~apoptotic cell clearance,GO:0045630~positive regulation of T-helper 2 cell differentiation,GO:0045787~positive regulation of cell cycle,GO:0045944~positive regulation of transcription from RNA polymerase II promoter,GO:0045947~negative regulation of translational initiation,GO:0051099~positive regulation of binding,GO:0055012~ventricular cardiac muscle cell differentiation,GO:0060010~Sertoli cell fate commitment,GO:0060173~limb development,GO:0060324~face development,GO:0060534~trachea cartilage development,GO:0060591~chondroblast differentiation,GO:0061037~negative regulation of cartilage development,GO:0071222~cellular response to lipopolysaccharide,GO:0071300~cellular response to retinoic acid,GO:0071391~cellular response to estrogen stimulus,
**ProxUt**	**DOWN**	ZBTB1	zinc finger and BTB domain containing 1(ZBTB1)	GO:0000122~negative regulation of transcription from RNA polymerase II promoter,GO:0002711~positive regulation of T cell mediated immunity,GO:0006338~chromatin remodeling,GO:0019985~translesion synthesis,GO:0030183~B cell differentiation,GO:0032825~positive regulation of natural killer cell differentiation,GO:0033077~T cell differentiation in thymus,GO:0034644~cellular response to UV,GO:0042789~mRNA transcription from RNA polymerase II promoter,GO:0048538~thymus development,GO:0051260~protein homooligomerization,GO:2000176~positive regulation of pro-T cell differentiation,
**ProxUt**	**DOWN**	TDG	thymine-DNA glycosylase(TDG)	GO:0000122~negative regulation of transcription from RNA polymerase II promoter,GO:0006285~base-excision repair, AP site formation,GO:0006298~mismatch repair,GO:0009790~embryo development,GO:0032091~negative regulation of protein binding,GO:0035562~negative regulation of chromatin binding,GO:0040029~regulation of gene expression, epigenetic,GO:0080111~DNA demethylation,
**ProxUt**	**DOWN**	PRDM1	PR/SET domain 1(PRDM1)	GO:0000122~negative regulation of transcription from RNA polymerase II promoter,GO:0048869~cellular developmental process,
**ProxUt**	**DOWN**	P2RX7	purinergic receptor P2X 7(P2RX7)	GO:0000187~activation of MAPK activity,GO:0000902~cell morphogenesis,GO:0001845~phagolysosome assembly,GO:0001916~positive regulation of T cell mediated cytotoxicity,GO:0006468~protein phosphorylation,GO:0006509~membrane protein ectodomain proteolysis,GO:0006649~phospholipid transfer to membrane,GO:0006900~membrane budding,GO:0006954~inflammatory

				response,GO:0007005~mitochondrion organization,GO:0009612~response to mechanical stimulus,GO:0010043~response to zinc ion,GO:0010524~positive regulation of calcium ion transport into cytosol,GO:0010628~positive regulation of gene expression,GO:0014049~positive regulation of glutamate secretion,GO:0014054~positive regulation of gamma-aminobutyric acid secretion,GO:0016079~synaptic vesicle exocytosis,GO:0016485~protein processing,GO:0017121~phospholipid scrambling,GO:0019233~sensory perception of pain,GO:0019835~cytolysis,GO:0030501~positive regulation of bone mineralization,GO:0031668~cellular response to extracellular stimulus,GO:0032060~bleb assembly,GO:0032308~positive regulation of prostaglandin secretion,GO:0032496~response to lipopolysaccharide,GO:0032755~positive regulation of interleukin-6 production,GO:0032963~collagen metabolic process,GO:0033198~response to ATP,GO:0034405~response to fluid shear stress,GO:0034767~positive regulation of ion transmembrane transport,GO:0042098~T cell proliferation,GO:0042493~response to drug,GO:0043029~T cell homeostasis,GO:0043132~NAD transport,GO:0043409~negative regulation of MAPK cascade,GO:0044254~multicellular organismal protein catabolic process,GO:0045332~phospholipid translocation,GO:0045779~negative regulation of bone resorption,GO:0045794~negative regulation of cell volume,GO:0045821~positive regulation of glycolytic process,GO:0046513~ceramide biosynthetic process,GO:0046931~pore complex assembly,GO:0048705~skeletal system morphogenesis,GO:0048873~homeostasis of number of cells within a tissue,GO:0050717~positive regulation of interleukin-1 alpha secretion,GO:0050718~positive regulation of interleukin-1 beta secretion,GO:0050830~defense response to Gram-positive bacterium,GO:0051209~release of sequestered calcium ion into cytosol,GO:0051259~protein oligomerization,GO:0051592~response to calcium ion,GO:0051602~response to electrical stimulus,GO:0051899~membrane depolarization,GO:0051901~positive regulation of mitochondrial depolarization,GO:0070230~positive regulation of lymphocyte apoptotic process,GO:0071359~cellular response to dsRNA,GO:0072593~reactive oxygen species metabolic process,GO:0097191~extrinsic apoptotic signaling pathway,GO:1904172~positive regulation of bleb assembly,
**ProxUt**	**DOWN**	GOLGA5	golgin A5(GOLGA5)	GO:0000301~retrograde transport, vesicle recycling within Golgi,GO:0007030~Golgi organization,
**ProxUt**	**DOWN**	ERCC6	ERCC excision repair 6, chromatin remodeling factor(ERCC6)	GO:0000303~response to superoxide,GO:0006283~transcription-coupled nucleotide-excision repair,GO:0006284~base-excision repair,GO:0006290~pyrimidine dimer repair,GO:0006362~transcription elongation from RNA polymerase I promoter,GO:0007256~activation of JNKK activity,GO:0007257~activation of JUN kinase activity,GO:0008630~intrinsic apoptotic signaling pathway in response to DNA damage,GO:0009636~response to toxic substance,GO:0010165~response to X-ray,GO:0010224~response to UV-B,GO:0010332~response to gamma radiation,GO:0032786~positive regulation of DNA-templated transcription, elongation,GO:0035264~multicellular organism growth,GO:0045494~photoreceptor cell maintenance,
**ProxUt**	**DOWN**	NOX4	NADPH oxidase 4(NOX4)	GO:0000902~cell morphogenesis,GO:0007569~cell aging,GO:0008285~negative regulation of cell proliferation,GO:0010467~gene expression,GO:0042554~superoxide anion generation,GO:0043406~positive regulation of MAP kinase activity,GO:0045453~bone resorption,GO:0050667~homocysteine metabolic process,GO:0051897~positive regulation of protein kinase B signaling,GO:0055007~cardiac muscle cell differentiation,GO:0070374~positive regulation of ERK1 and ERK2 cascade,GO:0071333~cellular response to glucose stimulus,GO:2000573~positive regulation of DNA biosynthetic process,
**ProxUt**	**DOWN**	VCAN	versican(VCAN)	GO:0001501~skeletal system development,GO:0001649~osteoblast differentiation,GO:0007155~cell adhesion,GO:0007417~central nervous system development,
**ProxUt**	**DOWN**	FBN1	fibrillin 1(FBN1)	GO:0001501~skeletal system development,GO:0006006~glucose metabolic process,GO:0007507~heart development,GO:0009653~anatomical structure morphogenesis,GO:0010737~protein kinase A signaling,GO:0034199~activation of protein kinase A activity,GO:0042593~glucose homeostasis,GO:0043010~camera-type eye development,GO:0048048~embryonic eye morphogenesis,GO:0048050~post-embryonic eye morphogenesis,GO:0090287~regulation of cellular response to growth factor stimulus,
**ProxUt**	**DOWN**	DMP1	dentin matrix acidic phosphoprotein 1(DMP1)	GO:0001503~ossification,GO:0010811~positive regulation of cell-substrate adhesion,GO:0030198~extracellular matrix organization,GO:0070173~regulation of enamel mineralization,
**ProxUt**	**DOWN**	ACKR3	atypical chemokine receptor 3(ACKR3)	GO:0001525~angiogenesis,GO:0001570~vasculogenesis,GO:0002230~positive regulation of defense response to virus by host,GO:0006935~chemotaxis,GO:0031623~receptor internalization,GO:0070374~positive regulation of ERK1 and ERK2 cascade,GO:0098792~xenophagy,GO:1902230~negative regulation of intrinsic apoptotic signaling pathway in response to DNA damage,
**ProxUt**	**DOWN**	EPAS1	endothelial PAS domain protein 1(EPAS1)	GO:0001525~angiogenesis,GO:0001892~embryonic placenta development,GO:0001974~blood vessel remodeling,GO:0002027~regulation of heart rate,GO:0006351~transcription, DNA-templated,GO:0007005~mitochondrion organization,GO:0007601~visual perception,GO:0030218~erythrocyte differentiation,GO:0030324~lung development,GO:0042415~norepinephrine metabolic process,GO:0043129~surfactant homeostasis,GO:0043619~regulation of transcription from RNA polymerase II promoter in response to oxidative stress,GO:0045944~positive regulation of transcription from RNA polymerase II promoter,GO:0048469~cell maturation,GO:0048625~myoblast fate commitment,GO:0055072~iron ion homeostasis,GO:0071456~cellular response to hypoxia,
**ProxUt**	**DOWN**	ENPEP	glutamyl aminopeptidase(ENPEP)	GO:0001525~angiogenesis,GO:0002003~angiotensin maturation,GO:0003081~regulation of systemic arterial blood pressure by renin-angiotensin,GO:0006508~proteolysis,GO:0008217~regulation of blood pressure,GO:0008283~cell proliferation,GO:0016477~cell migration,GO:0032835~glomerulus development,GO:0043171~peptide catabolic process,
**ProxUt**	**DOWN**	MFGE8	milk fat globule-EGF factor 8 protein(MFGE8)	GO:0001525~angiogenesis,GO:0006910~phagocytosis, recognition,GO:0006911~phagocytosis, engulfment,GO:0007155~cell adhesion,GO:0007338~single fertilization,GO:2000427~positive regulation of apoptotic cell clearance,
**ProxUt**	**DOWN**	SRPX2	sushi repeat containing protein, X-linked 2(SRPX2)	GO:0001525~angiogenesis,GO:0016337~single organismal cell-cell adhesion,GO:0042325~regulation of phosphorylation,GO:0048870~cell motility,GO:0051965~positive regulation of synapse assembly,GO:0071625~vocalization behavior,GO:0090050~positive regulation of cell migration involved in sprouting angiogenesis,
**ProxUt**	**DOWN**	LOC100525121	neuron navigator 1(LOC100525121)	GO:0001578~microtubule bundle formation,GO:0001764~neuron migration,
**ProxUt**	**DOWN**	GJA1	gap junction protein alpha 1(GJA1)	GO:0001649~osteoblast differentiation,GO:0001701~in utero embryonic development,GO:0001764~neuron migration,GO:0001947~heart looping,GO:0002070~epithelial cell maturation,GO:0002088~lens development in camera-type eye,GO:0002931~response to ischemia,GO:0003294~atrial ventricular junction remodeling,GO:0007154~cell communication,GO:0007267~cell-cell signaling,GO:0007507~heart development,GO:0007512~adult heart development,GO:0009611~response to wounding,GO:0010628~positive regulation of gene expression,GO:0010629~negative regulation of gene expression,GO:0010643~cell communication by chemical coupling,GO:0030308~negative regulation of cell growth,GO:0030500~regulation of bone mineralization,GO:0036120~cellular response to platelet-derived growth factor stimulus,GO:0042733~embryonic digit morphogenesis,GO:0043123~positive regulation of I-kappaB kinase/NF-kappaB signaling,GO:0043403~skeletal muscle tissue regeneration,GO:0045216~cell-cell junction organization,GO:0045669~positive regulation of osteoblast differentiation,GO:0045844~positive regulation of striated muscle tissue development,GO:0046850~regulation of bone remodeling,GO:0048514~blood vessel morphogenesis,GO:0055085~transmembrane transport,GO:0060156~milk ejection,GO:0060307~regulation of ventricular cardiac muscle cell membrane repolarization,GO:0060371~regulation of atrial cardiac muscle cell membrane depolarization,GO:0060373~regulation of ventricular cardiac muscle cell membrane depolarization,GO:0061337~cardiac conduction,GO:0071673~positive regulation of smooth muscle cell chemotaxis,
**ProxUt**	**DOWN**	MRC2	mannose receptor C type 2(MRC2)	GO:0001649~osteoblast differentiation,GO:0030574~collagen catabolic process,
**ProxUt**	**DOWN**	PLAU	plasminogen activator, urokinase(PLAU)	GO:0001666~response to hypoxia,GO:0006508~proteolysis,GO:0010469~regulation of receptor activity,GO:0014909~smooth muscle cell migration,GO:0014910~regulation of smooth muscle cell migration,GO:0030335~positive regulation of cell migration,GO:0033628~regulation of cell adhesion mediated by integrin,GO:0042127~regulation of cell proliferation,GO:0042730~fibrinolysis,GO:2000097~regulation of smooth muscle cell-matrix adhesion,
**ProxUt**	**DOWN**	PLOD2	procollagen-lysine,2-oxoglutarate 5-dioxygenase 2(PLOD2)	GO:0001666~response to hypoxia,GO:0098779~mitophagy in response to mitochondrial depolarization,
**ProxUt**	**DOWN**	MAN2A1	mannosidase alpha class 2A member 1(MAN2A1)	GO:0001701~in utero embryonic development,GO:0001889~liver development,GO:0006013~mannose metabolic process,GO:0006491~N-glycan processing,GO:0006517~protein deglycosylation,GO:0007005~mitochondrion organization,GO:0007033~vacuole organization,GO:0007585~respiratory gaseous exchange,GO:0048286~lung alveolus development,GO:0050769~positive regulation of neurogenesis,GO:0060042~retina morphogenesis in camera-type eye,
**ProxUt**	**DOWN**	LOC100739844	semaphorin-3A-like(LOC100739844)	GO:0001755~neural crest cell migration,GO:0021772~olfactory bulb development,GO:0048843~negative regulation of axon extension involved in axon guidance,GO:0050919~negative chemotaxis,GO:0071526~semaphorin-plexin signaling pathway,GO:2001224~positive regulation of neuron migration,
**ProxUt**	**DOWN**	ELF4	E74 like ETS transcription factor 4(ELF4)	GO:0001787~natural killer cell proliferation,GO:0001866~NK T cell proliferation,GO:0006357~regulation of transcription from RNA polymerase II promoter,GO:0030154~cell differentiation,GO:0045087~innate immune response,GO:0045944~positive regulation of transcription from RNA polymerase II promoter,
**ProxUt**	**DOWN**	IRAK4	interleukin 1 receptor associated kinase 4(IRAK4)	GO:0001816~cytokine production,GO:0002446~neutrophil mediated immunity,GO:0019221~cytokine-mediated signaling pathway,GO:0035556~intracellular signal transduction,GO:0043123~positive regulation of I-kappaB kinase/NF-kappaB signaling,GO:1990266~neutrophil migration,

**ProxUt**	**DOWN**	AGTR1	angiotensin II receptor type 1(AGTR1)	GO:0001822~kidney development,GO:0007266~Rho protein signal transduction,GO:0010873~positive regulation of cholesterol esterification,GO:0019229~regulation of vasoconstriction,GO:0019722~calcium-mediated signaling,GO:0032270~positive regulation of cellular protein metabolic process,GO:0032430~positive regulation of phospholipase A2 activity,GO:0051482~positive regulation of cytosolic calcium ion concentration involved in phospholipase C-activating G-protein coupled signaling pathway,GO:0060326~cell chemotaxis,GO:0086097~phospholipase C-activating angiotensin-activated signaling pathway,
**ProxUt**	**DOWN**	SDC4	syndecan 4(SDC4)	GO:0001843~neural tube closure,GO:0010762~regulation of fibroblast migration,GO:0016477~cell migration,GO:0042060~wound healing,GO:0060122~inner ear receptor stereocilium organization,GO:1903543~positive regulation of exosomal secretion,GO:1903553~positive regulation of extracellular exosome assembly,
**ProxUt**	**DOWN**	ECE1	endothelin converting enzyme 1(ECE1)	GO:0001921~positive regulation of receptor recycling,GO:0007507~heart development,GO:0010814~substance P catabolic process,GO:0010815~bradykinin catabolic process,GO:0010816~calcitonin catabolic process,GO:0016485~protein processing,GO:0034959~endothelin maturation,GO:0042733~embryonic digit morphogenesis,GO:0043583~ear development,GO:0060037~pharyngeal system development,
**ProxUt**	**DOWN**	STAT2	signal transducer and activator of transcription 2(STAT2)	GO:0001932~regulation of protein phosphorylation,GO:0002230~positive regulation of defense response to virus by host,GO:0006351~transcription, DNA-templated,GO:0006355~regulation of transcription, DNA-templated,GO:0051607~defense response to virus,GO:0060337~type I interferon signaling pathway,GO:0090140~regulation of mitochondrial fission,GO:0098779~mitophagy in response to mitochondrial depolarization,GO:0098792~xenophagy,
**ProxUt**	**DOWN**	PLAUR	plasminogen activator, urokinase receptor(PLAUR)	GO:0001934~positive regulation of protein phosphorylation,GO:0030162~regulation of proteolysis,GO:0038195~urokinase plasminogen activator signaling pathway,GO:0043388~positive regulation of DNA binding,GO:0045742~positive regulation of epidermal growth factor receptor signaling pathway,GO:0090200~positive regulation of release of cytochrome c from mitochondria,GO:2001243~negative regulation of intrinsic apoptotic signaling pathway,GO:2001268~negative regulation of cysteine-type endopeptidase activity involved in apoptotic signaling pathway,
**ProxUt**	**DOWN**	KDR	kinase insert domain receptor(KDR)	GO:0001938~positive regulation of endothelial cell proliferation,GO:0008360~regulation of cell shape,GO:0014068~positive regulation of phosphatidylinositol 3-kinase signaling,GO:0016239~positive regulation of macroautophagy,GO:0035584~calcium-mediated signaling using intracellular calcium source,GO:0038033~positive regulation of endothelial cell chemotaxis by VEGF-activated vascular endothelial growth factor receptor signaling pathway,GO:0045766~positive regulation of angiogenesis,GO:0046777~protein autophosphorylation,GO:0048010~vascular endothelial growth factor receptor signaling pathway,GO:0050927~positive regulation of positive chemotaxis,GO:0051770~positive regulation of nitric-oxide synthase biosynthetic process,GO:0051894~positive regulation of focal adhesion assembly,GO:0051901~positive regulation of mitochondrial depolarization,GO:0070374~positive regulation of ERK1 and ERK2 cascade,GO:0090141~positive regulation of mitochondrial fission,GO:2000352~negative regulation of endothelial cell apoptotic process,
**ProxUt**	**DOWN**	CRYGS	crystallin gamma S(CRYGS)	GO:0002009~morphogenesis of an epithelium,GO:0002088~lens development in camera-type eye,
**ProxUt**	**DOWN**	WNT2	Wnt family member 2(WNT2)	GO:0002053~positive regulation of mesenchymal cell proliferation,GO:0016055~Wnt signaling pathway,GO:0030182~neuron differentiation,GO:0033278~cell proliferation in midbrain,GO:0045165~cell fate commitment,GO:0045944~positive regulation of transcription from RNA polymerase II promoter,GO:0048146~positive regulation of fibroblast proliferation,GO:0050769~positive regulation of neurogenesis,GO:0051091~positive regulation of sequence-specific DNA binding transcription factor activity,GO:0055009~atrial cardiac muscle tissue morphogenesis,GO:0060045~positive regulation of cardiac muscle cell proliferation,GO:0060070~canonical Wnt signaling pathway,GO:0060317~cardiac epithelial to mesenchymal transition,GO:0060492~lung induction,GO:0060501~positive regulation of epithelial cell proliferation involved in lung morphogenesis,GO:0060716~labyrinthine layer blood vessel development,GO:0061180~mammary gland epithelium development,GO:0071560~cellular response to transforming growth factor beta stimulus,GO:0090263~positive regulation of canonical Wnt signaling pathway,GO:1904948~midbrain dopaminergic neuron differentiation,
**ProxUt**	**DOWN**	CREB3L2	cAMP responsive element binding protein 3 like 2(CREB3L2)	GO:0002062~chondrocyte differentiation,GO:0006888~ER to Golgi vesicle-mediated transport,GO:0030968~endoplasmic reticulum unfolded protein response,
**ProxUt**	**DOWN**	B4GALT1	beta-1,4-galactosyltransferase 1(B4GALT1)	GO:0002064~epithelial cell development,GO:0002526~acute inflammatory response,GO:0005989~lactose biosynthetic process,GO:0006012~galactose metabolic process,GO:0006487~protein N-linked glycosylation,GO:0007155~cell adhesion,GO:0007339~binding of sperm to zona pellucida,GO:0007341~penetration of zona pellucida,GO:0008285~negative regulation of cell proliferation,GO:0030198~extracellular matrix organization,GO:0030879~mammary gland development,GO:0045136~development of secondary sexual characteristics,GO:0048754~branching morphogenesis of an epithelial tube,GO:0050900~leukocyte migration,GO:0051270~regulation of cellular component movement,GO:0060046~regulation of acrosome reaction,GO:0060054~positive regulation of epithelial cell proliferation involved in wound healing,GO:0060055~angiogenesis involved in wound healing,GO:0060058~positive regulation of apoptotic process involved in mammary gland involution,
**ProxUt**	**DOWN**	FGFBP1	fibroblast growth factor binding protein 1(FGFBP1)	GO:0002230~positive regulation of defense response to virus by host,GO:0008284~positive regulation of cell proliferation,GO:0045743~positive regulation of fibroblast growth factor receptor signaling pathway,GO:0098779~mitophagy in response to mitochondrial depolarization,GO:0098792~xenophagy,
**ProxUt**	**DOWN**	TMEM39A	transmembrane protein 39A(TMEM39A)	GO:0002230~positive regulation of defense response to virus by host,GO:0098779~mitophagy in response to mitochondrial depolarization,GO:0098792~xenophagy,
**ProxUt**	**DOWN**	GCNT3	glucosaminyl (N-acetyl) transferase 3, mucin type(GCNT3)	GO:0002426~immunoglobulin production in mucosal tissue,GO:0048729~tissue morphogenesis,GO:0050892~intestinal absorption,GO:0060993~kidney morphogenesis,
**ProxUt**	**DOWN**	ABCB1	ATP-binding cassette, sub-family B (MDR/TAP), member 1(ABCB1)	GO:0002481~antigen processing and presentation of exogenous protein antigen via MHC class Ib, TAP-dependent,GO:0002485~antigen processing and presentation of endogenous peptide antigen via MHC class I via ER pathway, TAP-dependent,GO:0002489~antigen processing and presentation of endogenous peptide antigen via MHC class Ib via ER pathway, TAP-dependent,GO:0002591~positive regulation of antigen processing and presentation of peptide antigen via MHC class I,GO:0055085~transmembrane transport,
**ProxUt**	**DOWN**	VNN1	vanin 1(VNN1)	GO:0002526~acute inflammatory response,GO:0002544~chronic inflammatory response,GO:0006768~biotin metabolic process,GO:0006807~nitrogen compound metabolic process,GO:0007417~central nervous system development,GO:0015939~pantothenate metabolic process,GO:0016337~single organismal cell-cell adhesion,GO:0033089~positive regulation of T cell differentiation in thymus,GO:0045087~innate immune response,GO:1902176~negative regulation of oxidative stress-induced intrinsic apoptotic signaling pathway,
**ProxUt**	**DOWN**	GLB1L	galactosidase beta 1 like(GLB1L)	GO:0005975~carbohydrate metabolic process,
**ProxUt**	**DOWN**	GPD2	glycerol-3-phosphate dehydrogenase 2(GPD2)	GO:0006072~glycerol-3-phosphate metabolic process,GO:0006094~gluconeogenesis,GO:0035264~multicellular organism growth,GO:0043010~camera-type eye development,
**ProxUt**	**DOWN**	DCTD	dCMP deaminase(DCTD)	GO:0006220~pyrimidine nucleotide metabolic process,
**ProxUt**	**DOWN**	FAM111A	family with sequence similarity 111 member A(FAM111A)	GO:0006260~DNA replication,GO:0045071~negative regulation of viral genome replication,
**ProxUt**	**DOWN**	DCLRE1A	DNA cross-link repair 1A(DCLRE1A)	GO:0006303~double-strand break repair via nonhomologous end joining,GO:0031848~protection from non-homologous end joining at telomere,GO:0036297~interstrand cross-link repair,
**ProxUt**	**DOWN**	HOXA10	homeobox A10(HOXA10)	GO:0006355~regulation of transcription, DNA-templated,
**ProxUt**	**DOWN**	HOXA10	homeobox A10(HOXA10)	GO:0006355~regulation of transcription, DNA-templated,
**ProxUt**	**DOWN**	TCEAL8	transcription elongation factor A like 8(TCEAL8)	GO:0006357~regulation of transcription from RNA polymerase II promoter,
**ProxUt**	**DOWN**	MAGED1	MAGE family member D1(MAGED1)	GO:0006357~regulation of transcription from RNA polymerase II promoter,GO:0032922~circadian regulation of gene expression,GO:0042752~regulation of circadian rhythm,GO:0042981~regulation of apoptotic process,GO:0043406~positive regulation of MAP kinase activity,GO:0045892~negative regulation of transcription, DNA-templated,GO:0045893~positive regulation of transcription, DNA-templated,GO:0050680~negative regulation of epithelial cell proliferation,GO:0090190~positive regulation of branching involved in ureteric bud morphogenesis,GO:2001235~positive regulation of apoptotic signaling pathway,
**ProxUt**	**DOWN**	ZNF516	zinc finger protein 516(ZNF516)	GO:0006366~transcription from RNA polymerase II promoter,GO:0007165~signal transduction,GO:0007275~multicellular organism development,GO:0009409~response to cold,GO:0045893~positive regulation of transcription, DNA-templated,GO:0050873~brown fat cell differentiation,GO:0060612~adipose tissue development,
**ProxUt**	**DOWN**	ERP44	endoplasmic reticulum protein 44(ERP44)	GO:0006457~protein folding,GO:0006986~response to unfolded protein,GO:0009100~glycoprotein metabolic process,GO:0034976~response to endoplasmic reticulum stress,GO:0045454~cell redox homeostasis,
**ProxUt**	**DOWN**	GMNN	geminin, DNA replication inhibitor(GMNN)	GO:0006461~protein complex assembly,GO:0008156~negative regulation of DNA replication,GO:0009887~organ morphogenesis,GO:0045786~negative regulation of cell cycle,GO:0045892~negative regulation of transcription, DNA-templated,
**ProxUt**	**DOWN**	NCEH1	neutral cholesterol ester hydrolase 1(NCEH1)	GO:0006470~protein dephosphorylation,GO:0006805~xenobiotic metabolic process,GO:0009056~catabolic process,GO:0060395~SMAD protein signal transduction,
**ProxUt**	**DOWN**	B3GNT2	UDP-GlcNAc:betaGal beta-1,3-N-acetylglucosaminyltransferase 2(B3GNT2)	GO:0006486~protein glycosylation,

**ProxUt**	**DOWN**	B3GALT2	beta-1,3-galactosyltransferase 2(B3GALT2)	GO:0006486~protein glycosylation,GO:0009312~oligosaccharide biosynthetic process,
**ProxUt**	**DOWN**	MAN1A1	mannosidase alpha class 1A member 1(MAN1A1)	GO:0006491~N-glycan processing,GO:0008152~metabolic process,
**ProxUt**	**DOWN**	CPXM1	carboxypeptidase X, M14 family member 1(CPXM1)	GO:0006508~proteolysis,
**ProxUt**	**DOWN**	MMP1	matrix metallopeptidase 1(MMP1)	GO:0006508~proteolysis,GO:0030574~collagen catabolic process,GO:0032461~positive regulation of protein oligomerization,
**ProxUt**	**DOWN**	USP15	ubiquitin specific peptidase 15(USP15)	GO:0006511~ubiquitin-dependent protein catabolic process,GO:0016579~protein deubiquitination,
**ProxUt**	**DOWN**	SRP54	signal recognition particle 54(SRP54)	GO:0006614~SRP-dependent cotranslational protein targeting to membrane,GO:0042493~response to drug,
**ProxUt**	**DOWN**	KDELR3	KDEL endoplasmic reticulum protein retention receptor 3(KDELR3)	GO:0006621~protein retention in ER lumen,GO:0015031~protein transport,
**ProxUt**	**DOWN**	SEC23A	Sec23 homolog A, coat complex II component(SEC23A)	GO:0006886~intracellular protein transport,GO:0006888~ER to Golgi vesicle-mediated transport,
**ProxUt**	**DOWN**	TBC1D15	TBC1 domain family member 15(TBC1D15)	GO:0006886~intracellular protein transport,GO:0031338~regulation of vesicle fusion,GO:0090630~activation of GTPase activity,
**ProxUt**	**DOWN**	EXOC5	exocyst complex component 5(EXOC5)	GO:0006887~exocytosis,GO:0042384~cilium assembly,GO:0048278~vesicle docking,
**ProxUt**	**DOWN**	LMAN2	lectin, mannose binding 2(LMAN2)	GO:0006888~ER to Golgi vesicle-mediated transport,GO:0006890~retrograde vesicle-mediated transport, Golgi to ER,GO:0007029~endoplasmic reticulum organization,GO:0007030~Golgi organization,GO:0050766~positive regulation of phagocytosis,
**ProxUt**	**DOWN**	NSF	N-ethylmaleimide sensitive factor, vesicle fusing ATPase(NSF)	GO:0006891~intra-Golgi vesicle-mediated transport,GO:0043001~Golgi to plasma membrane protein transport,GO:0048211~Golgi vesicle docking,
**ProxUt**	**DOWN**	CHIC2	cysteine rich hydrophobic domain 2(CHIC2)	GO:0006893~Golgi to plasma membrane transport,
**ProxUt**	**DOWN**	RAB8B	RAB8B, member RAS oncogene family(RAB8B)	GO:0006904~vesicle docking involved in exocytosis,GO:0007264~small GTPase mediated signal transduction,GO:0009306~protein secretion,GO:0019882~antigen processing and presentation,GO:0045046~protein import into peroxisome membrane,
**ProxUt**	**DOWN**	TMEM74	transmembrane protein 74(TMEM74)	GO:0006914~autophagy,
**ProxUt**	**DOWN**	GPX8	glutathione peroxidase 8 (putative)(GPX8)	GO:0006979~response to oxidative stress,
**ProxUt**	**DOWN**	CLDN11	claudin 11(CLDN11)	GO:0007155~cell adhesion,GO:0007283~spermatogenesis,GO:0008366~axon ensheathment,
**ProxUt**	**DOWN**	SELL	selectin L(SELL)	GO:0007155~cell adhesion,GO:0033198~response to ATP,
**ProxUt**	**DOWN**	LOC100520265	protocadherin-9(LOC100520265)	GO:0007156~homophilic cell adhesion via plasma membrane adhesion molecules,
**ProxUt**	**DOWN**	CDH11	cadherin 11(CDH11)	GO:0007156~homophilic cell adhesion via plasma membrane adhesion molecules,GO:0021957~corticospinal tract morphogenesis,
**ProxUt**	**DOWN**	COL3A1	collagen type III alpha 1 chain(COL3A1)	GO:0007160~cell-matrix adhesion,GO:0007179~transforming growth factor beta receptor signaling pathway,GO:0007229~integrin-mediated signaling pathway,GO:0007507~heart development,GO:0009314~response to radiation,GO:0018149~peptide cross-linking,GO:0021987~cerebral cortex development,GO:0030199~collagen fibril organization,GO:0034097~response to cytokine,GO:0035025~positive regulation of Rho protein signal transduction,GO:0042060~wound healing,GO:0043206~extracellular fibril organization,GO:0043588~skin development,GO:0048565~digestive tract development,GO:0050777~negative regulation of immune response,GO:0060414~aorta smooth muscle tissue morphogenesis,GO:0071230~cellular response to amino acid stimulus,GO:2001223~negative regulation of neuron migration,
**ProxUt**	**DOWN**	PDE7B	phosphodiesterase 7B(PDE7B)	GO:0007165~signal transduction,
**ProxUt**	**DOWN**	SPARC	secreted protein, acidic, cysteine-rich (osteonectin)(SPARC)	GO:0007165~signal transduction,GO:0048856~anatomical structure development,
**ProxUt**	**DOWN**	GNAI1	G protein subunit alpha i1(GNAI1)	GO:0007188~adenylate cyclase-modulating G-protein coupled receptor signaling pathway,GO:0051301~cell division,
**ProxUt**	**DOWN**	TAC3	tachykinin 3(TAC3)	GO:0007217~tachykinin receptor signaling pathway,GO:0007218~neuropeptide signaling pathway,
**ProxUt**	**DOWN**	PTPN1	protein tyrosine phosphatase, non-receptor type 1(PTPN1)	GO:0007257~activation of JUN kinase activity,GO:0008286~insulin receptor signaling pathway,GO:0030100~regulation of endocytosis,GO:0030948~negative regulation of vascular endothelial growth factor receptor signaling pathway,GO:0031532~actin cytoskeleton reorganization,GO:0033157~regulation of intracellular protein transport,GO:0035791~platelet-derived growth factor receptor-beta signaling pathway,GO:0036498~IRE1-mediated unfolded protein response,GO:0043407~negative regulation of MAP kinase activity,GO:0061098~positive regulation of protein tyrosine kinase activity,GO:0070373~negative regulation of ERK1 and ERK2 cascade,GO:1902202~regulation of hepatocyte growth factor receptor signaling pathway,GO:1902236~negative regulation of endoplasmic reticulum stress-induced intrinsic apoptotic signaling pathway,GO:1903898~negative regulation of PERK-mediated unfolded protein response,GO:1990264~peptidyl-tyrosine dephosphorylation involved in inactivation of protein kinase activity,GO:2000646~positive regulation of receptor catabolic process,
**ProxUt**	**DOWN**	RAB32	RAB32, member RAS oncogene family(RAB32)	GO:0007264~small GTPase mediated signal transduction,GO:0019882~antigen processing and presentation,GO:0032438~melanosome organization,GO:0035646~endosome to melanosome transport,GO:0090382~phagosome maturation,GO:1903232~melanosome assembly,
**ProxUt**	**DOWN**	RAP2C	RAP2C, member of RAS oncogene family(RAP2C)	GO:0007264~small GTPase mediated signal transduction,GO:0030336~negative regulation of cell migration,GO:0031954~positive regulation of protein autophosphorylation,GO:0032486~Rap protein signal transduction,GO:0061097~regulation of protein tyrosine kinase activity,GO:0090557~establishment of endothelial intestinal barrier,
**ProxUt**	**DOWN**	ABHD2	abhydrolase domain containing 2(ABHD2)	GO:0007340~acrosome reaction,GO:0009611~response to wounding,GO:0030336~negative regulation of cell migration,GO:0032570~response to progesterone,GO:0046464~acylglycerol catabolic process,GO:0048240~sperm capacitation,
**ProxUt**	**DOWN**	MORC3	MORC family CW-type zinc finger 3(MORC3)	GO:0007569~cell aging,GO:0009791~post-embryonic development,GO:0018105~peptidyl-serine phosphorylation,GO:0048147~negative regulation of fibroblast proliferation,GO:0050821~protein stabilization,GO:0051457~maintenance of protein location in nucleus,
**ProxUt**	**DOWN**	LUM	lumican(LUM)	GO:0007601~visual perception,GO:0030199~collagen fibril organization,GO:0032914~positive regulation of transforming growth factor beta1 production,GO:0045944~positive regulation of transcription from RNA polymerase II promoter,
**ProxUt**	**DOWN**	AFF2	AF4/FMR2 family member 2(AFF2)	GO:0007611~learning or memory,GO:0043484~regulation of RNA splicing,
**ProxUt**	**DOWN**	EPHA4	EPH receptor A4(EPHA4)	GO:0007628~adult walking behavior,GO:0008045~motor neuron axon guidance,GO:0018108~peptidyl-tyrosine phosphorylation,GO:0021957~corticospinal tract morphogenesis,GO:0043507~positive regulation of JUN kinase activity,GO:0046777~protein autophosphorylation,GO:0048681~negative regulation of axon regeneration,GO:0048710~regulation of astrocyte differentiation,GO:0050770~regulation of axonogenesis,GO:0061001~regulation of dendritic spine morphogenesis,GO:0072178~nephric duct morphogenesis,GO:0097155~fasciculation of sensory neuron axon,GO:0097156~fasciculation of motor neuron axon,GO:2001108~positive regulation of Rho guanyl-nucleotide exchange factor activity,
**ProxUt**	**DOWN**	LOC100736973	ras-related protein Rap-1b(LOC100736973)	GO:0008283~cell proliferation,GO:0032486~Rap protein signal transduction,GO:0061028~establishment of endothelial barrier,GO:0070374~positive regulation of ERK1 and ERK2 cascade,GO:0071320~cellular response to cAMP,GO:1901888~regulation of cell junction assembly,GO:2000114~regulation of establishment of cell polarity,GO:2000301~negative regulation of synaptic vesicle exocytosis,
**ProxUt**	**DOWN**	FRZB	frizzled-related protein(FRZB)	GO:0008285~negative regulation of cell proliferation,GO:0010721~negative regulation of cell development,GO:0014033~neural crest cell differentiation,GO:0030308~negative regulation of cell growth,GO:0035567~non-canonical Wnt signaling pathway,GO:0043065~positive regulation of apoptotic process,GO:0045600~positive regulation of fat cell differentiation,GO:0060029~convergent extension involved in organogenesis,GO:0060070~canonical Wnt signaling pathway,GO:0061037~negative regulation of cartilage development,GO:0061053~somite development,GO:0070367~negative regulation of hepatocyte differentiation,GO:0090090~negative regulation of canonical Wnt signaling pathway,GO:0090103~cochlea morphogenesis,

ProxUt	DOWN	LOC100623707	phosphatidylinositol 3-kinase regulatory subunit alpha-like(LOC100623707)	GO:0008286~insulin receptor signaling pathway,GO:0043551~regulation of phosphatidylinositol 3-kinase activity,GO:0046854~phosphatidylinositol phosphorylation,
ProxUt	DOWN	ADAM22	ADAM metallopeptidase domain 22(ADAM22)	GO:0008344~adult locomotory behavior,GO:0022011~myelination in peripheral nervous system,
ProxUt	DOWN	DRAM1	DNA damage regulated autophagy modulator 1(DRAM1)	GO:0010506~regulation of autophagy,
ProxUt	DOWN	AGR2	anterior gradient 2, protein disulphide isomerase family member(AGR2)	GO:0010628~positive regulation of gene expression,GO:0010811~positive regulation of cell-substrate adhesion,GO:0030154~cell differentiation,GO:0045742~positive regulation of epidermal growth factor receptor signaling pathway,GO:0048546~digestive tract morphogenesis,GO:0048639~positive regulation of developmental growth,GO:0060480~lung goblet cell differentiation,GO:0060548~negative regulation of cell death,GO:0070254~mucus secretion,GO:0090004~positive regulation of establishment of protein localization to plasma membrane,GO:1903896~positive regulation of IRE1-mediated unfolded protein response,GO:1903899~positive regulation of PERK-mediated unfolded protein response,
ProxUt	DOWN	FAP	fibroblast activation protein alpha(FAP)	GO:0010710~regulation of collagen catabolic process,GO:0010716~negative regulation of extracellular matrix disassembly,GO:0043542~endothelial cell migration,GO:0051603~proteolysis involved in cellular protein catabolic process,GO:0060244~negative regulation of cell proliferation involved in contact inhibition,GO:0071158~positive regulation of cell cycle arrest,GO:0071850~mitotic cell cycle arrest,GO:0097325~melanocyte proliferation,GO:1900119~positive regulation of execution phase of apoptosis,GO:1902362~melanocyte apoptotic process,
ProxUt	DOWN	BMP1	bone morphogenetic protein 1(BMP1)	GO:0010862~positive regulation of pathway-restricted SMAD protein phosphorylation,GO:0030509~BMP signaling pathway,GO:0042981~regulation of apoptotic process,GO:0043408~regulation of MAPK cascade,GO:0048468~cell development,GO:0060395~SMAD protein signal transduction,GO:0061036~positive regulation of cartilage development,
ProxUt	DOWN	CLIC2	chloride intracellular channel 2(CLIC2)	GO:0010880~regulation of release of sequestered calcium ion into cytosol by sarcoplasmic reticulum,GO:0051099~positive regulation of binding,GO:0060315~negative regulation of ryanodine-sensitive calcium-release channel activity,GO:1902476~chloride transmembrane transport,
ProxUt	DOWN	IER3	immediate early response 3(IER3)	GO:0010941~regulation of cell death,GO:0043066~negative regulation of apoptotic process,GO:2001020~regulation of response to DNA damage stimulus,
ProxUt	DOWN	STEAP2	STEAP2 metalloreductase(STEAP2)	GO:0015677~copper ion import,GO:0097461~ferric iron import into cell,
ProxUt	DOWN	SEC22B	SEC22 homolog B, vesicle trafficking protein(SEC22B)	GO:0016192~vesicle-mediated transport,
ProxUt	DOWN	CLINT1	clathrin interactor 1(CLINT1)	GO:0016192~vesicle-mediated transport,
ProxUt	DOWN	SDC2	syndecan 2(SDC2)	GO:0016477~cell migration,GO:0048813~dendrite morphogenesis,GO:0048814~regulation of dendrite morphogenesis,
ProxUt	DOWN	FBXL5	F-box and leucine rich repeat protein 5(FBXL5)	GO:0016567~protein ubiquitination,GO:0031146~SCF-dependent proteasomal ubiquitin-dependent protein catabolic process,GO:0055072~iron ion homeostasis,GO:1903364~positive regulation of cellular protein catabolic process,
ProxUt	DOWN	FBLN1	fibulin 1(FBLN1)	GO:0030198~extracellular matrix organization,
ProxUt	DOWN	CRISPLD2	cysteine rich secretory protein LCCL domain containing 2(CRISPLD2)	GO:0030198~extracellular matrix organization,GO:0060325~face morphogenesis,
ProxUt	DOWN	SLC12A2	solute carrier family 12 member 2(SLC12A2)	GO:0030321~transepithelial chloride transport,GO:0070634~transepithelial ammonium transport,
ProxUt	DOWN	HSD11B1	hydroxysteroid 11-beta dehydrogenase 1(HSD11B1)	GO:0030324~lung development,
ProxUt	DOWN	ERLEC1	endoplasmic reticulum lectin 1(ERLEC1)	GO:0030433~ER-associated ubiquitin-dependent protein catabolic process,GO:0030970~retrograde protein transport, ER to cytosol,GO:1904153~negative regulation of retrograde protein transport, ER to cytosol,
ProxUt	DOWN	OMD	osteomodulin(OMD)	GO:0030500~regulation of bone mineralization,
ProxUt	DOWN	CREB3L1	cAMP responsive element binding protein 3 like 1(CREB3L1)	GO:0030968~endoplasmic reticulum unfolded protein response,GO:0045944~positive regulation of transcription from RNA polymerase II promoter,
ProxUt	DOWN	SIPA1L1	signal induced proliferation associated 1 like 1(SIPA1L1)	GO:0031532~actin cytoskeleton reorganization,GO:0048013~ephrin receptor signaling pathway,GO:0048814~regulation of dendrite morphogenesis,GO:0050770~regulation of axonogenesis,GO:0051056~regulation of small GTPase mediated signal transduction,
ProxUt	DOWN	PPP4R4	protein phosphatase 4 regulatory subunit 4(PPP4R4)	GO:0032515~negative regulation of phosphoprotein phosphatase activity,GO:0080163~regulation of protein serine/threonine phosphatase activity,
ProxUt	DOWN	PLEKHG2	pleckstrin homology and RhoGEF domain containing G2(PLEKHG2)	GO:0035023~regulation of Rho protein signal transduction,
ProxUt	DOWN	PDP1	pyruvate dehyrogenase phosphatase catalytic subunit 1(PDP1)	GO:0035970~peptidyl-threonine dephosphorylation,
ProxUt	DOWN	COL7A1	collagen type VII alpha 1 chain(COL7A1)	GO:0035987~endodermal cell differentiation,
ProxUt	DOWN	FGFR1OP2	FGFR1 oncogene partner 2(FGFR1OP2)	GO:0042060~wound healing,
ProxUt	DOWN	MDFIC	MyoD family inhibitor domain containing(MDFIC)	GO:0042308~negative regulation of protein import into nucleus,GO:0045892~negative regulation of transcription, DNA-templated,GO:0050434~positive regulation of viral transcription,
ProxUt	DOWN	ADAMTS9	ADAM metallopeptidase with thrombospondin type 1 motif 9(ADAMTS9)	GO:0045636~positive regulation of melanocyte differentiation,
ProxUt	DOWN	BRINP2	BMP/retinoic acid inducible neural specific 2(BRINP2)	GO:0045666~positive regulation of neuron differentiation,GO:0045930~negative regulation of mitotic cell cycle,GO:0071300~cellular response to retinoic acid,
ProxUt	DOWN	MFAP2	microfibrillar associated protein 2(MFAP2)	GO:0048048~embryonic eye morphogenesis,GO:0048050~post-embryonic eye morphogenesis,
ProxUt	DOWN	FKBP7	FK506 binding protein 7(FKBP7)	GO:0061077~chaperone-mediated protein folding,
ProxUt	DOWN	UACA	uveal autoantigen with coiled-coil domains and ankyrin repeats(UACA)	GO:0097190~apoptotic signaling pathway,GO:1901222~regulation of NIK/NF-kappaB signaling,
ProxUt	DOWN	LOC100513074	serine protease inhibitor Kazal-type 9(LOC100513074)	GO:1900004~negative regulation of serine-type endopeptidase activity,
ProxUt	DOWN	LOC100517015	cAMP-dependent protein kinase inhibitor beta-like(LOC100517015)	GO:2000480~negative regulation of cAMP-dependent protein kinase activity,
UTJ	UP	EP300	E1A binding protein p300(EP300)	GO:0000122~negative regulation of transcription from RNA polymerase II promoter,GO:0001666~response to hypoxia,GO:0001756~somitogenesis,GO:0006355~regulation of transcription, DNA-templated,GO:0006990~positive regulation of transcription from RNA polymerase II promoter involved in unfolded protein response,GO:0007507~heart development,GO:0007519~skeletal muscle tissue development,GO:0007623~circadian rhythm,GO:0009887~organ morphogenesis,GO:0018076~N-terminal peptidyl-lysine acetylation,GO:0030183~B cell differentiation,GO:0030220~platelet formation,GO:0030324~lung development,GO:0032092~positive regulation of protein binding,GO:0034644~cellular response to UV,GO:0035855~megakaryocyte development,GO:0042771~intrinsic apoptotic signaling pathway in response to DNA damage by p53 class mediator,GO:0043627~response to estrogen,GO:0043923~positive regulation by host of viral transcription,GO:0043967~histone H4 acetylation,GO:0043969~histone H2B acetylation,GO:0045444~fat cell differentiation,GO:0050821~protein stabilization,GO:0051091~positive regulation of sequence-specific DNA binding transcription factor activity,GO:0060765~regulation of androgen receptor signaling pathway,GO:0090043~regulation of tubulin deacetylation,
UTJ	UP	TSHZ2	teashirt zinc finger homeobox 2(TSHZ2)	GO:0000122~negative regulation of transcription from RNA polymerase II promoter,GO:0006351~transcription, DNA-templated,GO:0006357~regulation of transcription from RNA polymerase II promoter,GO:0007275~multicellular organism development,GO:0010468~regulation of gene expression,
UTJ	UP	LOC100627422	ligand-dependent corepressor(LOC100627422)	GO:0000122~negative regulation of transcription from RNA polymerase II promoter,GO:0006366~transcription from RNA polymerase II promoter,

**UTJ**	**UP**	TTLL6	tubulin tyrosine ligase like 6(TTLL6)	GO:0001578~microtubule bundle formation,GO:0003353~positive regulation of cilium movement,GO:0018095~protein polyglutamylation,GO:0051013~microtubule severing,
**UTJ**	**UP**	BMPER	BMP binding endothelial regulator(BMPER)	GO:0001657~ureteric bud development,GO:0002043~blood vessel endothelial cell proliferation involved in sprouting angiogenesis,GO:0010594~regulation of endothelial cell migration,GO:0030514~negative regulation of BMP signaling pathway,GO:0042118~endothelial cell activation,GO:0045446~endothelial cell differentiation,GO:0048839~inner ear development,GO:0060393~regulation of pathway-restricted SMAD protein phosphorylation,GO:0070374~positive regulation of ERK1 and ERK2 cascade,
**UTJ**	**UP**	CTDSPL	CTD small phosphatase like(CTDSPL)	GO:0001933~negative regulation of protein phosphorylation,GO:2000134~negative regulation of G1/S transition of mitotic cell cycle,
**UTJ**	**UP**	CA9	carbonic anhydrase 9(CA9)	GO:0002009~morphogenesis of an epithelium,GO:0006730~one-carbon metabolic process,GO:0046903~secretion,
**UTJ**	**UP**	ARRB1	arrestin beta 1(ARRB1)	GO:0002031~G-protein coupled receptor internalization,GO:0002092~positive regulation of receptor internalization,GO:0006366~transcription from RNA polymerase II promoter,GO:0007602~phototransduction,GO:0016567~protein ubiquitination,GO:0031397~negative regulation of protein ubiquitination,GO:0032088~negative regulation of NF-kappaB transcription factor activity,GO:0032092~positive regulation of protein binding,GO:0032715~negative regulation of interleukin-6 production,GO:0032717~negative regulation of interleukin-8 production,GO:0033138~positive regulation of peptidyl-serine phosphorylation,GO:0035025~positive regulation of Rho protein signal transduction,GO:0035774~positive regulation of insulin secretion involved in cellular response to glucose stimulus,GO:0043149~stress fiber assembly,GO:0043161~proteasome-mediated ubiquitin-dependent protein catabolic process,GO:0045944~positive regulation of transcription from RNA polymerase II promoter,GO:0070374~positive regulation of ERK1 and ERK2 cascade,GO:0090240~positive regulation of histone H4 acetylation,
**UTJ**	**UP**	RDH10	retinol dehydrogenase 10 (all-trans)(RDH10)	GO:0002138~retinoic acid biosynthetic process,
**UTJ**	**UP**	JAK2	Janus kinase 2(JAK2)	GO:0002250~adaptive immune response,GO:0006954~inflammatory response,GO:0007165~signal transduction,GO:0007169~transmembrane receptor protein tyrosine kinase signaling pathway,GO:0007260~tyrosine phosphorylation of STAT protein,GO:0010811~positive regulation of cell-substrate adhesion,GO:0016477~cell migration,GO:0019221~cytokine-mediated signaling pathway,GO:0030155~regulation of cell adhesion,GO:0030218~erythrocyte differentiation,GO:0033209~tumor necrosis factor-mediated signaling pathway,GO:0035409~histone H3-Y41 phosphorylation,GO:0035556~intracellular signal transduction,GO:0035722~interleukin-12-mediated signaling pathway,GO:0038083~peptidyl-tyrosine autophosphorylation,GO:0042127~regulation of cell proliferation,GO:0042517~positive regulation of tyrosine phosphorylation of Stat3 protein,GO:0042977~activation of JAK2 kinase activity,GO:0042981~regulation of apoptotic process,GO:0045087~innate immune response,GO:0046677~response to antibiotic,GO:0046777~protein autophosphorylation,GO:0050727~regulation of inflammatory response,GO:0060396~growth hormone receptor signaling pathway,GO:0060397~JAK-STAT cascade involved in growth hormone signaling pathway,
**UTJ**	**UP**	HAVCR2	hepatitis A virus cellular receptor 2(HAVCR2)	GO:0002281~macrophage activation involved in immune response,GO:0002519~natural killer cell tolerance induction,GO:0002652~regulation of tolerance induction dependent upon immune response,GO:0002826~negative regulation of T-helper 1 type immune response,GO:0002859~negative regulation of natural killer cell mediated cytotoxicity directed against tumor cell target,GO:0010629~negative regulation of gene expression,GO:0030886~negative regulation of myeloid dendritic cell activation,GO:0032088~negative regulation of NF-kappaB transcription factor activity,GO:0032687~negative regulation of interferon-alpha production,GO:0032689~negative regulation of interferon-gamma production,GO:0032703~negative regulation of interleukin-2 production,GO:0032712~negative regulation of interleukin-3 production,GO:0032715~negative regulation of interleukin-6 production,GO:0032720~negative regulation of tumor necrosis factor production,GO:0032722~positive regulation of chemokine production,GO:0032729~positive regulation of interferon-gamma production,GO:0032732~positive regulation of interleukin-1 production,GO:0032753~positive regulation of interleukin-4 production,GO:0032815~negative regulation of natural killer cell activation,GO:0034138~toll-like receptor 3 signaling pathway,GO:0034154~toll-like receptor 7 signaling pathway,GO:0034162~toll-like receptor 9 signaling pathway,GO:0042102~positive regulation of T cell proliferation,GO:0042130~negative regulation of T cell proliferation,GO:0043032~positive regulation of macrophage activation,GO:0050830~defense response to Gram-positive bacterium,GO:0060135~maternal process involved in female pregnancy,GO:0070374~positive regulation of ERK1 and ERK2 cascade,GO:0071222~cellular response to lipopolysaccharide,GO:0071656~negative regulation of granulocyte colony-stimulating factor production,GO:1900425~negative regulation of defense response to bacterium,GO:1900426~positive regulation of defense response to bacterium,GO:1901224~positive regulation of NIK/NF-kappaB signaling,GO:1904469~positive regulation of tumor necrosis factor secretion,GO:2000521~negative regulation of immunological synapse formation,GO:2001189~negative regulation of T cell activation via T cell receptor contact with antigen bound to MHC molecule on antigen presenting cell,
**UTJ**	**UP**	RENBP	renin binding protein(RENBP)	GO:0006044~N-acetylglucosamine metabolic process,GO:0010951~negative regulation of endopeptidase activity,GO:0019262~N-acetylneuraminate catabolic process,
**UTJ**	**UP**	UAP1	UDP-N-acetylglucosamine pyrophosphorylase 1(UAP1)	GO:0006048~UDP-N-acetylglucosamine biosynthetic process,
**UTJ**	**UP**	ALDH2	aldehyde dehydrogenase 2 family (mitochondrial)(ALDH2)	GO:0006068~ethanol catabolic process,GO:0055114~oxidation-reduction process,
**UTJ**	**UP**	LOC100154396	sister chromatid cohesion protein PDS5 homolog B(LOC100154396)	GO:0006281~DNA repair,GO:0007064~mitotic sister chromatid cohesion,
**UTJ**	**UP**	IRF2	interferon regulatory factor 2(IRF2)	GO:0006351~transcription, DNA-templated,GO:0008283~cell proliferation,
**UTJ**	**UP**	MAFB	MAF bZIP transcription factor B(MAFB)	GO:0006357~regulation of transcription from RNA polymerase II promoter,GO:0007379~segment specification,GO:0007399~nervous system development,GO:0007585~respiratory gaseous exchange,GO:0021571~rhombomere 5 development,GO:0021572~rhombomere 6 development,GO:0033077~T cell differentiation in thymus,GO:0035284~brain segmentation,GO:0042472~inner ear morphogenesis,GO:0045647~negative regulation of erythrocyte differentiation,GO:0045671~negative regulation of osteoclast differentiation,GO:0048538~thymus development,
**UTJ**	**UP**	ZNF217	zinc finger protein 217(ZNF217)	GO:0006366~transcription from RNA polymerase II promoter,GO:0007165~signal transduction,GO:0022008~neurogenesis,
**UTJ**	**UP**	CHKA	choline kinase alpha(CHKA)	GO:0006646~phosphatidylethanolamine biosynthetic process,
**UTJ**	**UP**	HPGD	hydroxyprostaglandin dehydrogenase 15-(NAD)(HPGD)	GO:0006693~prostaglandin metabolic process,GO:0007179~transforming growth factor beta receptor signaling pathway,GO:0007565~female pregnancy,GO:0007567~parturition,GO:0030728~ovulation,GO:0045786~negative regulation of cell cycle,GO:0070493~thrombin receptor signaling pathway,GO:0097070~ductus arteriosus closure,
**UTJ**	**UP**	EPS15	epidermal growth factor receptor pathway substrate 15(EPS15)	GO:0006895~Golgi to endosome transport,GO:0019065~receptor-mediated endocytosis of virus by host cell,GO:0048268~clathrin coat assembly,
**UTJ**	**UP**	ELMO1	engulfment and cell motility 1(ELMO1)	GO:0006911~phagocytosis, engulfment,GO:0016477~cell migration,GO:0016601~Rac protein signal transduction,GO:0030036~actin cytoskeleton organization,
**UTJ**	**UP**	CD27	CD27 molecule(CD27)	GO:0006954~inflammatory response,GO:0006955~immune response,GO:0007420~brain development,GO:0008625~extrinsic apoptotic signaling pathway via death domain receptors,GO:0032496~response to lipopolysaccharide,GO:0042127~regulation of cell proliferation,GO:0043281~regulation of cysteine-type endopeptidase activity involved in apoptotic process,GO:0043410~positive regulation of MAPK cascade,GO:0046330~positive regulation of JNK cascade,GO:0070233~negative regulation of T cell apoptotic process,
**UTJ**	**UP**	CDC14A	cell division cycle 14A(CDC14A)	GO:0007096~regulation of exit from mitosis,GO:0042384~cilium assembly,GO:0051256~mitotic spindle midzone assembly,GO:0071850~mitotic cell cycle arrest,
**UTJ**	**UP**	ARHGAP20	Rho GTPase activating protein 20(ARHGAP20)	GO:0007165~signal transduction,
**UTJ**	**UP**	MYLIP	myosin regulatory light chain interacting protein(MYLIP)	GO:0007399~nervous system development,GO:0010989~negative regulation of low-density lipoprotein particle clearance,GO:0031648~protein destabilization,GO:0032803~regulation of low-density lipoprotein particle receptor catabolic process,GO:0042632~cholesterol homeostasis,GO:0042787~protein ubiquitination involved in ubiquitin-dependent protein catabolic process,GO:0045732~positive regulation of protein catabolic process,
**UTJ**	**UP**	ADAP2	ArfGAP with dual PH domains 2(ADAP2)	GO:0007507~heart development,
**UTJ**	**UP**	DLEC1	deleted in lung and esophageal cancer 1(DLEC1)	GO:0008285~negative regulation of cell proliferation,
**UTJ**	**UP**	MTR	5-methyltetrahydrofolate-homocysteine methyltransferase(MTR)	GO:0009235~cobalamin metabolic process,GO:0042558~pteridine-containing compound metabolic process,

**UTJ**	**UP**	HIPK3	homeodomain interacting protein kinase 3(HIPK3)	GO:0009299~mRNA transcription,GO:0018105~peptidyl-serine phosphorylation,GO:0018107~peptidyl-threonine phosphorylation,GO:0043066~negative regulation of apoptotic process,GO:0043508~negative regulation of JUN kinase activity,
**UTJ**	**UP**	CAMK1D	calcium/calmodulin dependent protein kinase ID(CAMK1D)	GO:0010976~positive regulation of neuron projection development,GO:0032793~positive regulation of CREB transcription factor activity,GO:0043065~positive regulation of apoptotic process,GO:0043066~negative regulation of apoptotic process,GO:0050766~positive regulation of phagocytosis,GO:0050773~regulation of dendrite development,GO:0060267~positive regulation of respiratory burst,GO:0090023~positive regulation of neutrophil chemotaxis,
**UTJ**	**UP**	CHDH	choline dehydrogenase(CHDH)	GO:0019285~glycine betaine biosynthetic process from choline,
**UTJ**	**UP**	TEKT2	tektin 2(TEKT2)	GO:0030317~sperm motility,GO:0036159~inner dynein arm assembly,
**UTJ**	**UP**	ANKZF1	ankyrin repeat and zinc finger domain containing 1(ANKZF1)	GO:0030433~ER-associated ubiquitin-dependent protein catabolic process,GO:0071630~nucleus-associated proteasomal ubiquitin-dependent protein catabolic process,GO:0072671~mitochondria-associated ubiquitin-dependent protein catabolic process,
**UTJ**	**UP**	C1QC	complement C1q C chain(C1QC)	GO:0030853~negative regulation of granulocyte differentiation,GO:0045650~negative regulation of macrophage differentiation,
**UTJ**	**UP**	EPB41L1	erythrocyte membrane protein band 4.1 like 1(EPB41L1)	GO:0030866~cortical actin cytoskeleton organization,GO:0031032~actomyosin structure organization,
**UTJ**	**UP**	STMND1	stathmin domain containing 1(STMND1)	GO:0031110~regulation of microtubule polymerization or depolymerization,
**UTJ**	**UP**	RNF128	ring finger protein 128, E3 ubiquitin protein ligase(RNF128)	GO:0031647~regulation of protein stability,GO:0042036~negative regulation of cytokine biosynthetic process,
**UTJ**	**UP**	ECT2L	epithelial cell transforming 2 like(ECT2L)	GO:0035023~regulation of Rho protein signal transduction,
**UTJ**	**UP**	SLC2A12	solute carrier family 2 member 12(SLC2A12)	GO:0035428~hexose transmembrane transport,GO:0046323~glucose import,
**UTJ**	**UP**	CHN2	chimerin 2(CHN2)	GO:0035556~intracellular signal transduction,GO:0043087~regulation of GTPase activity,GO:0051056~regulation of small GTPase mediated signal transduction,
**UTJ**	**UP**	JADE1	jade family PHD finger 1(JADE1)	GO:0043966~histone H3 acetylation,GO:0043981~histone H4-K5 acetylation,GO:0043982~histone H4-K8 acetylation,GO:0043983~histone H4-K12 acetylation,GO:2000134~negative regulation of G1/S transition of mitotic cell cycle,
**UTJ**	**UP**	DYRK1A	dual specificity tyrosine phosphorylation regulated kinase 1A(DYRK1A)	GO:0046777~protein autophosphorylation,
**UTJ**	**UP**	KCNRG	potassium channel regulator(KCNRG)	GO:0051260~protein homooligomerization,GO:1902260~negative regulation of delayed rectifier potassium channel activity,
**UTJ**	**DOWN**	CALM2	calmodulin 2(CALM2)	GO:0000086~G2/M transition of mitotic cell cycle,GO:0002027~regulation of heart rate,GO:0005513~detection of calcium ion,GO:0010880~regulation of release of sequestered calcium ion into cytosol by sarcoplasmic reticulum,GO:0021762~substantia nigra development,GO:0030801~positive regulation of cyclic nucleotide metabolic process,GO:0032465~regulation of cytokinesis,GO:0032516~positive regulation of phosphoprotein phosphatase activity,GO:0043388~positive regulation of DNA binding,GO:0051343~positive regulation of cyclic-nucleotide phosphodiesterase activity,GO:0055117~regulation of cardiac muscle contraction,GO:0060316~positive regulation of ryanodine-sensitive calcium-release channel activity,
**UTJ**	**DOWN**	GAPDH	glyceraldehyde-3-phosphate dehydrogenase(GAPDH)	GO:0000226~microtubule cytoskeleton organization,GO:0006006~glucose metabolic process,GO:0006096~glycolytic process,GO:0006417~regulation of translation,GO:0035606~peptidyl-cysteine S-trans-nitrosylation,GO:0050821~protein stabilization,GO:0051402~neuron apoptotic process,GO:0055114~oxidation-reduction process,
**UTJ**	**DOWN**	VCAN	versican(VCAN)	GO:0001501~skeletal system development,GO:0001649~osteoblast differentiation,GO:0007155~cell adhesion,GO:0007417~central nervous system development,
**UTJ**	**DOWN**	FBN1	fibrillin 1(FBN1)	GO:0001501~skeletal system development,GO:0006006~glucose metabolic process,GO:0007507~heart development,GO:0009653~anatomical structure morphogenesis,GO:0010737~protein kinase A signaling,GO:0034199~activation of protein kinase A activity,GO:0042593~glucose homeostasis,GO:0043010~camera-type eye development,GO:0048048~embryonic eye morphogenesis,GO:0048050~post-embryonic eye morphogenesis,GO:0090287~regulation of cellular response to growth factor stimulus,
**UTJ**	**DOWN**	ACKR3	atypical chemokine receptor 3(ACKR3)	GO:0001525~angiogenesis,GO:0001570~vasculogenesis,GO:0002230~positive regulation of defense response to virus by host,GO:0006935~chemotaxis,GO:0031623~receptor internalization,GO:0070374~positive regulation of ERK1 and ERK2 cascade,GO:0098792~xenophagy,GO:1902230~negative regulation of intrinsic apoptotic signaling pathway in response to DNA damage,
**UTJ**	**DOWN**	SRPX2	sushi repeat containing protein, X-linked 2(SRPX2)	GO:0001525~angiogenesis,GO:0016337~single organismal cell-cell adhesion,GO:0042325~regulation of phosphorylation,GO:0048870~cell motility,GO:0051965~positive regulation of synapse assembly,GO:0071625~vocalization behavior,GO:0090050~positive regulation of cell migration involved in sprouting angiogenesis,
**UTJ**	**DOWN**	PLAUR	plasminogen activator, urokinase receptor(PLAUR)	GO:0001934~positive regulation of protein phosphorylation,GO:0030162~regulation of proteolysis,GO:0038195~urokinase plasminogen activator signaling pathway,GO:0043388~positive regulation of DNA binding,GO:0045742~positive regulation of epidermal growth factor receptor signaling pathway,GO:0090200~positive regulation of release of cytochrome c from mitochondria,GO:2001243~negative regulation of intrinsic apoptotic signaling pathway,GO:2001268~negative regulation of cysteine-type endopeptidase activity involved in apoptotic signaling pathway,
**UTJ**	**DOWN**	CREB3L2	cAMP responsive element binding protein 3 like 2(CREB3L2)	GO:0002062~chondrocyte differentiation,GO:0006888~ER to Golgi vesicle-mediated transport,GO:0030968~endoplasmic reticulum unfolded protein response,
**UTJ**	**DOWN**	B4GALT1	beta-1,4-galactosyltransferase 1(B4GALT1)	GO:0002064~epithelial cell development,GO:0002526~acute inflammatory response,GO:0005989~lactose biosynthetic process,GO:0006012~galactose metabolic process,GO:0006487~protein N-linked glycosylation,GO:0007155~cell adhesion,GO:0007339~binding of sperm to zona pellucida,GO:0007341~penetration of zona pellucida,GO:0008285~negative regulation of cell proliferation,GO:0030198~extracellular matrix organization,GO:0030879~mammary gland development,GO:0045136~development of secondary sexual characteristics,GO:0048754~branching morphogenesis of an epithelial tube,GO:0050900~leukocyte migration,GO:0051270~regulation of cellular component movement,GO:0060046~regulation of acrosome reaction,GO:0060054~positive regulation of epithelial cell proliferation involved in wound healing,GO:0060055~angiogenesis involved in wound healing,GO:0060058~positive regulation of apoptotic process involved in mammary gland involution,
**UTJ**	**DOWN**	CD63	CD63 molecule(CD63)	GO:0002092~positive regulation of receptor internalization,GO:0007155~cell adhesion,GO:0007160~cell-matrix adhesion,GO:0007166~cell surface receptor signaling pathway,GO:0016477~cell migration,GO:0034613~cellular protein localization,GO:0035646~endosome to melanosome transport,GO:0045807~positive regulation of endocytosis,GO:1900746~regulation of vascular endothelial growth factor signaling pathway,GO:2000680~regulation of rubidium ion transport,GO:2001046~positive regulation of integrin-mediated signaling pathway,
**UTJ**	**DOWN**	PGK1	phosphoglycerate kinase 1(PGK1)	GO:0006096~glycolytic process,GO:0016310~phosphorylation,
**UTJ**	**DOWN**	NPR2	natriuretic peptide receptor B/guanylate cyclase B (atrionatriuretic peptide receptor B)(NPR2)	GO:0006182~cGMP biosynthetic process,GO:0007168~receptor guanylyl cyclase signaling pathway,GO:0035556~intracellular signal transduction,GO:0044702~single organism reproductive process,GO:0051447~negative regulation of meiotic cell cycle,GO:0060348~bone development,GO:0097011~cellular response to granulocyte macrophage colony-stimulating factor stimulus,GO:1900194~negative regulation of oocyte maturation,
**UTJ**	**DOWN**	CCNO	cyclin O(CCNO)	GO:0006284~base-excision repair,GO:0007049~cell cycle,GO:0042384~cilium assembly,GO:0051301~cell division,GO:1903251~multi-ciliated epithelial cell differentiation,
**UTJ**	**DOWN**	MAGED1	MAGE family member D1(MAGED1)	GO:0006357~regulation of transcription from RNA polymerase II promoter,GO:0032922~circadian regulation of gene expression,GO:0042752~regulation of circadian rhythm,GO:0042981~regulation of apoptotic process,GO:0043406~positive regulation of MAP kinase activity,GO:0045892~negative regulation of transcription, DNA-templated,GO:0045893~positive regulation of transcription, DNA-templated,GO:0050680~negative regulation of epithelial cell proliferation,GO:0090190~positive regulation of branching involved in ureteric bud morphogenesis,GO:2001235~positive regulation of apoptotic signaling pathway,
**UTJ**	**DOWN**	EEF1G	eukaryotic translation elongation factor 1 gamma(EEF1G)	GO:0006414~translational elongation,GO:0006749~glutathione metabolic process,GO:0009615~response to virus,
**UTJ**	**DOWN**	CPXM1	carboxypeptidase X, M14 family member 1(CPXM1)	GO:0006508~proteolysis,
**UTJ**	**DOWN**	ALDH18A1	aldehyde dehydrogenase 18 family member A1(ALDH18A1)	GO:0006536~glutamate metabolic process,GO:0006561~proline biosynthetic process,GO:0006592~ornithine biosynthetic process,GO:0019240~citrulline biosynthetic process,
**UTJ**	**DOWN**	SEC61G	Sec61 translocon gamma subunit(SEC61G)	GO:0006605~protein targeting,
**UTJ**	**DOWN**	KDELR3	KDEL endoplasmic reticulum protein retention receptor 3(KDELR3)	GO:0006621~protein retention in ER lumen,GO:0015031~protein transport,

**UTJ**	**DOWN**	SEC23A	Sec23 homolog A, coat complex II component(SEC23A)	GO:0006886~intracellular protein transport,GO:0006888~ER to Golgi vesicle-mediated transport,
**UTJ**	**DOWN**	ATP8B2	ATPase phospholipid transporting 8B2(ATP8B2)	GO:0007030~Golgi organization,
**UTJ**	**DOWN**	SHAS2	hyaluronan synthase 2(SHAS2)	GO:0007155~cell adhesion,GO:0030213~hyaluronan biosynthetic process,GO:0035810~positive regulation of urine volume,GO:0045226~extracellular polysaccharide biosynthetic process,GO:0070295~renal water absorption,GO:0085029~extracellular matrix assembly,
**UTJ**	**DOWN**	GNG2	G protein subunit gamma 2(GNG2)	GO:0007186~G-protein coupled receptor signaling pathway,GO:0008283~cell proliferation,
**UTJ**	**DOWN**	GDI2	GDP dissociation inhibitor 2(GDI2)	GO:0007264~small GTPase mediated signal transduction,GO:0015031~protein transport,GO:0043547~positive regulation of GTPase activity,GO:0055114~oxidation-reduction process,
**UTJ**	**DOWN**	RHOC	ras homolog family member C(RHOC)	GO:0007264~small GTPase mediated signal transduction,GO:0043297~apical junction assembly,GO:0044319~wound healing, spreading of cells,GO:1902766~skeletal muscle satellite cell migration,
**UTJ**	**DOWN**	ABHD2	abhydrolase domain containing 2(ABHD2)	GO:0007340~acrosome reaction,GO:0009611~response to wounding,GO:0030336~negative regulation of cell migration,GO:0032570~response to progesterone,GO:0046464~acylglycerol catabolic process,GO:0048240~sperm capacitation,
**UTJ**	**DOWN**	CD248	CD248 molecule(CD248)	GO:0008284~positive regulation of cell proliferation,GO:0016477~cell migration,GO:0048535~lymph node development,GO:0060033~anatomical structure regression,GO:2000353~positive regulation of endothelial cell apoptotic process,
**UTJ**	**DOWN**	HMGCS1	3-hydroxy-3-methylglutaryl-CoA synthase 1(HMGCS1)	GO:0008299~isoprenoid biosynthetic process,
**UTJ**	**DOWN**	FBXL5	F-box and leucine rich repeat protein 5(FBXL5)	GO:0016567~protein ubiquitination,GO:0031146~SCF-dependent proteasomal ubiquitin-dependent protein catabolic process,GO:0055072~iron ion homeostasis,GO:1903364~positive regulation of cellular protein catabolic process,
**UTJ**	**DOWN**	CYP51	cytochrome P450, family 51, subfamily A, polypeptide 1(CYP51)	GO:0033488~cholesterol biosynthetic process via 24,25-dihydrolanosterol,GO:0055114~oxidation-reduction process,GO:0070988~demethylation,
**UTJ**	**DOWN**	FAM19A4	family with sequence similarity 19 member A4, C-C motif chemokine like(FAM19A4)	GO:0042391~regulation of membrane potential,GO:0051930~regulation of sensory perception of pain,

Table 7. List of biological pathways classified according to **molecular function** (DAVID database**,** P< 0.05) of differentially-expressed genes (DEGs, ID and name), solely related to copulation, in pig uterine mucosae (endocervix (Cvx), endometrium (distal: D-Endom or proximal: P-Endom) and utero-tubal junction (UTJ)), up- or down-regulated.

Tissue	Regulation	ID	Gene Name	GOTERM_MF_DIRECT
Cvx	UP	TSHZ2	teashirt zinc finger homeobox 2(TSHZ2)	GO:0001078~transcriptional repressor activity, RNA polymerase II core promoter proximal region sequence-specific binding,GO:0003677~DNA binding,GO:0003682~chromatin binding,GO:0046872~metal ion binding,
Cvx	UP	LOC100520903	zinc finger protein 300-like(LOC100520903)	GO:0003676~nucleic acid binding,GO:0003700~transcription factor activity, sequence-specific DNA binding,GO:0046872~metal ion binding,
Cvx	UP	DDX17	DEAD-box helicase 17(DDX17)	GO:0003676~nucleic acid binding,GO:0004004~ATP-dependent RNA helicase activity,GO:0005524~ATP binding,
Cvx	UP	ZBTB44	zinc finger and BTB domain containing 44(ZBTB44)	GO:0003676~nucleic acid binding,GO:0046872~metal ion binding,
Cvx	UP	AKAP9	A-kinase anchoring protein 9(AKAP9)	GO:0003677~DNA binding,
Cvx	UP	NCOR2	nuclear receptor corepressor 2(NCOR2)	GO:0003677~DNA binding,GO:0003714~transcription corepressor activity,GO:0008134~transcription factor binding,GO:0016922~ligand-dependent nuclear receptor binding,
Cvx	UP	ENPP5	ectonucleotide pyrophosphatase/phosphodiesterase 5 (putative)(ENPP5)	GO:0003824~catalytic activity,
Cvx	UP	CPD	carboxypeptidase D(CPD)	GO:0004181~metallocarboxypeptidase activity,GO:0004185~serine-type carboxypeptidase activity,GO:0008270~zinc ion binding,
Cvx	UP	LOC100153094	glutathione S-transferase theta-1(LOC100153094)	GO:0004364~glutathione transferase activity,
Cvx	UP	HDAC9	histone deacetylase 9(HDAC9)	GO:0004407~histone deacetylase activity,
Cvx	UP	FMO1	flavin containing monooxygenase 1(FMO1)	GO:0004499~N,N-dimethylaniline monooxygenase activity,GO:0050660~flavin adenine dinucleotide binding,GO:0050661~NADP binding,
Cvx	UP	PCBD1	pterin-4 alpha-carbinolamine dehydratase 1(PCBD1)	GO:0004505~phenylalanine 4-monooxygenase activity,GO:0008124~4-alpha-hydroxytetrahydrobiopterin dehydratase activity,
Cvx	UP	PRKCQ	protein kinase C theta(PRKCQ)	GO:0004674~protein serine/threonine kinase activity,GO:0004697~protein kinase C activity,GO:0005524~ATP binding,GO:0046872~metal ion binding,
Cvx	UP	IGF1R	insulin like growth factor 1 receptor(IGF1R)	GO:0004714~transmembrane receptor protein tyrosine kinase activity,GO:0005520~insulin-like growth factor binding,GO:0005524~ATP binding,
Cvx	UP	MYLIP	myosin regulatory light chain interacting protein(MYLIP)	GO:0004842~ubiquitin-protein transferase activity,GO:0008270~zinc ion binding,GO:0061630~ubiquitin protein ligase activity,
Cvx	UP	KCNQ4	potassium voltage-gated channel subfamily Q member 4(KCNQ4)	GO:0005251~delayed rectifier potassium channel activity,
Cvx	UP	VAMP2	vesicle associated membrane protein 2(VAMP2)	GO:0005484~SNAP receptor activity,GO:0005543~phospholipid binding,GO:0019905~syntaxin binding,
Cvx	UP	THBS3	thrombospondin 3(THBS3)	GO:0005509~calcium ion binding,GO:0008201~heparin binding,
Cvx	UP	PYGO1	pygopus family PHD finger 1(PYGO1)	GO:0008270~zinc ion binding,
Cvx	UP	RNF149	ring finger protein 149(RNF149)	GO:0008270~zinc ion binding,GO:0061630~ubiquitin protein ligase activity,
Cvx	UP	DECR2	2,4-dienoyl-CoA reductase 2, peroxisomal(DECR2)	GO:0008670~2,4-dienoyl-CoA reductase (NADPH) activity,GO:0019166~trans-2-enoyl-CoA reductase (NADPH) activity,
Cvx	UP	LOC102166095	nuclear receptor coactivator 2-like(LOC102166095)	GO:0016922~ligand-dependent nuclear receptor binding,GO:0030374~ligand-dependent nuclear receptor transcription coactivator activity,GO:0035257~nuclear hormone receptor binding,
Cvx	UP	CDKAL1	CDK5 regulatory subunit associated protein 1 like 1(CDKAL1)	GO:0035598~N6-threonylcarbomyladenosine methylthiotransferase activity,GO:0051539~4 iron, 4 sulfur cluster binding,
Cvx	UP	RETSAT	retinol saturase(RETSAT)	GO:0051786~all-trans-retinol 13,14-reductase activity,
Cvx	DOWN	TDG	thymine-DNA glycosylase(TDG)	GO:0001104~RNA polymerase II transcription cofactor activity,GO:0004844~uracil DNA N-glycosylase activity,GO:0008263~pyrimidine-specific mismatch base pair DNA N-glycosylase activity,GO:0030983~mismatched DNA binding,GO:0043566~structure-specific DNA binding,
Cvx	DOWN	NPY2R	neuropeptide Y receptor Y2(NPY2R)	GO:0001601~peptide YY receptor activity,GO:0004930~G-protein coupled receptor activity,GO:0004983~neuropeptide Y receptor activity,
Cvx	DOWN	THBS1	thrombospondin 1(THBS1)	GO:0001786~phosphatidylserine binding,GO:0005509~calcium ion binding,GO:0008201~heparin binding,GO:0030169~low-density lipoprotein particle binding,GO:0050840~extracellular matrix binding,
Cvx	DOWN	ZBP1	Z-DNA binding protein 1(ZBP1)	GO:0003677~DNA binding,GO:0003723~RNA binding,GO:0003726~double-stranded RNA adenosine deaminase activity,
Cvx	DOWN	ABHD2	abhydrolase domain containing 2(ABHD2)	GO:0003707~steroid hormone receptor activity,GO:0042562~hormone binding,GO:0047372~acylglycerol lipase activity,
Cvx	DOWN	SLC25A17	solute carrier family 25 member 17(SLC25A17)	GO:0003735~structural constituent of ribosome,GO:0005347~ATP transmembrane transporter activity,GO:0015217~ADP transmembrane transporter activity,GO:0015228~coenzyme A transmembrane transporter activity,GO:0015230~FAD transmembrane transporter activity,GO:0044610~FMN transmembrane transporter activity,GO:0051724~NAD transporter activity,GO:0080122~AMP transmembrane transporter activity,
Cvx	DOWN	SLA-7	MHC class I antigen 7(SLA-7)	GO:0003823~antigen binding,
Cvx	DOWN	RSAD2	radical S-adenosyl methionine domain containing 2(RSAD2)	GO:0003824~catalytic activity,GO:0043621~protein self-association,GO:0046872~metal ion binding,GO:0051539~4 iron, 4 sulfur cluster binding,
Cvx	DOWN	B4GALT1	beta-1,4-galactosyltransferase 1(B4GALT1)	GO:0003831~beta-N-acetylglucosaminylglycopeptide beta-1,4-galactosyltransferase activity,GO:0003945~N-acetyllactosamine synthase activity,GO:0004461~lactose synthase activity,GO:0030145~manganese ion binding,
Cvx	DOWN	AGPAT2	1-acylglycerol-3-phosphate O-acyltransferase 2(AGPAT2)	GO:0003841~1-acylglycerol-3-phosphate O-acyltransferase activity,
Cvx	DOWN	MX1	myxovirus (influenza virus) resistance 1, interferon-inducible protein p78 (mouse)(MX1)	GO:0003924~GTPase activity,GO:0005525~GTP binding,GO:0008017~microtubule binding,
Cvx	DOWN	PAPSS2	3'-phosphoadenosine 5'-phosphosulfate synthase 2(PAPSS2)	GO:0004020~adenylylsulfate kinase activity,GO:0004781~sulfate adenylyltransferase (ATP) activity,GO:0005524~ATP binding,
Cvx	DOWN	CMPK2	cytidine/uridine monophosphate kinase 2(CMPK2)	GO:0004127~cytidylate kinase activity,GO:0004550~nucleoside diphosphate kinase activity,GO:0004798~thymidylate kinase activity,GO:0009041~uridylate kinase activity,GO:0033862~UMP kinase activity,
Cvx	DOWN	FDPS	farnesyl diphosphate synthase(FDPS)	GO:0004161~dimethylallyltranstransferase activity,GO:0004337~geranyltranstransferase activity,GO:0044822~poly(A) RNA binding,
Cvx	DOWN	MMP3	matrix metallopeptidase 3 (stromelysin 1, progelatinase)(MMP3)	GO:0004222~metalloendopeptidase activity,GO:0005509~calcium ion binding,GO:0008270~zinc ion binding,
Cvx	DOWN	HTRA4	HtrA serine peptidase 4(HTRA4)	GO:0004252~serine-type endopeptidase activity,
Cvx	DOWN	PLAT	plasminogen activator, tissue type(PLAT)	GO:0004252~serine-type endopeptidase activity,
Cvx	DOWN	PRSS23	protease, serine 23(PRSS23)	GO:0004252~serine-type endopeptidase activity,
Cvx	DOWN	NPR2	natriuretic peptide receptor B/guanylate cyclase B (atrionatriuretic peptide receptor B)(NPR2)	GO:0004383~guanylate cyclase activity,GO:0004672~protein kinase activity,GO:0005524~ATP binding,GO:0016941~natriuretic peptide receptor activity,GO:0042562~hormone binding,

**Cvx**	**DOWN**	HMGCR	3-hydroxy-3-methylglutaryl-CoA reductase(HMGCR)	GO:0004420~hydroxymethylglutaryl-CoA reductase (NADPH) activity,GO:0042282~hydroxymethylglutaryl-CoA reductase activity,GO:0050661~NADP binding,GO:0070402~NADPH binding,
**Cvx**	**DOWN**	HMGCS1	3-hydroxy-3-methylglutaryl-CoA synthase 1(HMGCS1)	GO:0004421~hydroxymethylglutaryl-CoA synthase activity,
**Cvx**	**DOWN**	CYP51	cytochrome P450, family 51, subfamily A, polypeptide 1(CYP51)	GO:0004497~monooxygenase activity,GO:0005506~iron ion binding,GO:0008168~methyltransferase activity,GO:0008398~sterol 14-demethylase activity,GO:0016705~oxidoreductase activity, acting on paired donors, with incorporation or reduction of molecular oxygen,GO:0020037~heme binding,
**Cvx**	**DOWN**	SQLE	squalene epoxidase(SQLE)	GO:0004506~squalene monooxygenase activity,GO:0050660~flavin adenine dinucleotide binding,
**Cvx**	**DOWN**	MAN2A1	mannosidase alpha class 2A member 1(MAN2A1)	GO:0004559~alpha-mannosidase activity,GO:0008270~zinc ion binding,GO:0016799~hydrolase activity, hydrolyzing N-glycosyl compounds,GO:0030246~carbohydrate binding,
**Cvx**	**DOWN**	PANK3	pantothenate kinase 3(PANK3)	GO:0004594~pantothenate kinase activity,GO:0005524~ATP binding,
**Cvx**	**DOWN**	PGD	phosphogluconate dehydrogenase(PGD)	GO:0004616~phosphogluconate dehydrogenase (decarboxylating) activity,
**Cvx**	**DOWN**	KSR1	kinase suppressor of ras 1(KSR1)	GO:0004672~protein kinase activity,GO:0005524~ATP binding,GO:0046872~metal ion binding,
**Cvx**	**DOWN**	RIPK3	receptor interacting serine/threonine kinase 3(RIPK3)	GO:0004704~NF-kappaB-inducing kinase activity,GO:0005524~ATP binding,
**Cvx**	**DOWN**	MAPK6	mitogen-activated protein kinase 6(MAPK6)	GO:0004707~MAP kinase activity,GO:0005524~ATP binding,
**Cvx**	**DOWN**	UBASH3B	ubiquitin associated and SH3 domain containing B(UBASH3B)	GO:0004725~protein tyrosine phosphatase activity,
**Cvx**	**DOWN**	SEPHS2	selenophosphate synthetase 2(SEPHS2)	GO:0004756~selenide, water dikinase activity,GO:0005524~ATP binding,
**Cvx**	**DOWN**	GARS	glycyl-tRNA synthetase(GARS)	GO:0004820~glycine-tRNA ligase activity,GO:0005524~ATP binding,
**Cvx**	**DOWN**	UBA7	ubiquitin like modifier activating enzyme 7(UBA7)	GO:0004839~ubiquitin activating enzyme activity,GO:0004842~ubiquitin-protein transferase activity,GO:0005524~ATP binding,GO:0008641~small protein activating enzyme activity,GO:0019782~ISG15 activating enzyme activity,
**Cvx**	**DOWN**	LOC100518083	E3 ISG15--protein ligase HERC5(LOC100518083)	GO:0004842~ubiquitin-protein transferase activity,GO:0016874~ligase activity,
**Cvx**	**DOWN**	GJA1	gap junction protein alpha 1(GJA1)	GO:0004871~signal transducer activity,GO:0015075~ion transmembrane transporter activity,GO:0022857~transmembrane transporter activity,GO:1903763~gap junction channel activity involved in cell communication by electrical coupling,
**Cvx**	**DOWN**	LOC100154959	olfactory receptor 4K3(LOC100154959)	GO:0004888~transmembrane signaling receptor activity,GO:0004930~G-protein coupled receptor activity,GO:0004984~olfactory receptor activity,
**Cvx**	**DOWN**	IL6ST	interleukin 6 signal transducer(IL6ST)	GO:0004896~cytokine receptor activity,GO:0004915~interleukin-6 receptor activity,GO:0004921~interleukin-11 receptor activity,GO:0004923~leukemia inhibitory factor receptor activity,GO:0004924~oncostatin-M receptor activity,
**Cvx**	**DOWN**	VCAN	versican(VCAN)	GO:0005201~extracellular matrix structural constituent,GO:0005509~calcium ion binding,GO:0005540~hyaluronic acid binding,
**Cvx**	**DOWN**	NPC1	NPC intracellular cholesterol transporter 1(NPC1)	GO:0005319~lipid transporter activity,GO:0015485~cholesterol binding,
**Cvx**	**DOWN**	SLC30A7	solute carrier family 30 member 7(SLC30A7)	GO:0005385~zinc ion transmembrane transporter activity,
**Cvx**	**DOWN**	PLOD2	procollagen-lysine,2-oxoglutarate 5-dioxygenase 2(PLOD2)	GO:0005506~iron ion binding,GO:0008475~procollagen-lysine 5-dioxygenase activity,GO:0031418~L-ascorbic acid binding,
**Cvx**	**DOWN**	CDH7	cadherin 7(CDH7)	GO:0005509~calcium ion binding,
**Cvx**	**DOWN**	ANXA5	annexin A5(ANXA5)	GO:0005509~calcium ion binding,GO:0005544~calcium-dependent phospholipid binding,
**Cvx**	**DOWN**	LRP8	LDL receptor related protein 8(LRP8)	GO:0005509~calcium ion binding,GO:0030229~very-low-density lipoprotein particle receptor activity,
**Cvx**	**DOWN**	LOC100152428	multidrug resistance-associated protein 1-like(LOC100152428)	GO:0005524~ATP binding,GO:0042626~ATPase activity, coupled to transmembrane movement of substances,
**Cvx**	**DOWN**	IFI44	interferon induced protein 44(IFI44)	GO:0005525~GTP binding,
**Cvx**	**DOWN**	RASD1	ras related dexamethasone induced 1(RASD1)	GO:0005525~GTP binding,
**Cvx**	**DOWN**	MFSD2A	major facilitator superfamily domain containing 2A(MFSD2A)	GO:0005548~phospholipid transporter activity,GO:0015293~symporter activity,
**Cvx**	**DOWN**	CXCL10	C-X-C motif chemokine ligand 10(CXCL10)	GO:0008009~chemokine activity,GO:0008201~heparin binding,GO:0045236~CXCR chemokine receptor binding,
**Cvx**	**DOWN**	NT5C2	5'-nucleotidase, cytosolic II(NT5C2)	GO:0008253~5'-nucleotidase activity,GO:0046872~metal ion binding,
**Cvx**	**DOWN**	CPXM1	carboxypeptidase X, M14 family member 1(CPXM1)	GO:0008270~zinc ion binding,
**Cvx**	**DOWN**	ENPEP	glutamyl aminopeptidase(ENPEP)	GO:0008270~zinc ion binding,GO:0042277~peptide binding,GO:0070006~metalloaminopeptidase activity,
**Cvx**	**DOWN**	STARD4	StAR related lipid transfer domain containing 4(STARD4)	GO:0008289~lipid binding,GO:0015485~cholesterol binding,
**Cvx**	**DOWN**	B3GALT2	beta-1,3-galactosyltransferase 2(B3GALT2)	GO:0008499~UDP-galactose:beta-N-acetylglucosamine beta-1,3-galactosyltransferase activity,
**Cvx**	**DOWN**	LOC100511841	UDP-glucuronosyltransferase 1-10(LOC100511841)	GO:0016758~transferase activity, transferring hexosyl groups,
**Cvx**	**DOWN**	OLFML3	olfactomedin like 3(OLFML3)	GO:0019164~pyruvate synthase activity,
**Cvx**	**DOWN**	XPNPEP1	X-prolyl aminopeptidase 1(XPNPEP1)	GO:0030145~manganese ion binding,GO:0046872~metal ion binding,GO:0070006~metalloaminopeptidase activity,
**Cvx**	**DOWN**	LOC100739844	semaphorin-3A-like(LOC100739844)	GO:0030215~semaphorin receptor binding,GO:0038191~neuropilin binding,GO:0045499~chemorepellent activity,
**Cvx**	**DOWN**	PLAUR	plasminogen activator, urokinase receptor(PLAUR)	GO:0030377~urokinase plasminogen activator receptor activity,
**Cvx**	**DOWN**	SNX31	sorting nexin 31(SNX31)	GO:0035091~phosphatidylinositol binding,
**Cvx**	**DOWN**	S1PR3	sphingosine-1-phosphate receptor 3(S1PR3)	GO:0038036~sphingosine-1-phosphate receptor activity,
**Cvx**	**DOWN**	SGPP2	sphingosine-1-phosphate phosphatase 2(SGPP2)	GO:0042392~sphingosine-1-phosphate phosphatase activity,
**Cvx**	**DOWN**	ATP6V0C	ATPase H+ transporting V0 subunit c(ATP6V0C)	GO:0046961~proton-transporting ATPase activity, rotational mechanism,
**Cvx**	**DOWN**	GCNT3	glucosaminyl (N-acetyl) transferase 3, mucin type(GCNT3)	GO:0047225~acetylgalactosaminyl-O-glycosyl-glycoprotein beta-1,6-N-acetylglucosaminyltransferase activity,
**Cvx**	**DOWN**	CD47	CD47 molecule(CD47)	GO:0070053~thrombospondin receptor activity,
**Cvx**	**DOWN**	DCBLD1	discoidin, CUB and LCCL domain containing 1(DCBLD1)	GO:0070492~oligosaccharide binding,
**DistUt**	**UP**	STX10	syntaxin 10(STX10)	GO:0000149~SNARE binding,GO:0005484~SNAP receptor activity,
**DistUt**	**UP**	IDH3G	isocitrate dehydrogenase 3 (NAD(+)) gamma(IDH3G)	GO:0000287~magnesium ion binding,GO:0004449~isocitrate dehydrogenase (NAD+) activity,GO:0051287~NAD binding,
**DistUt**	**UP**	PRPSAP1	phosphoribosyl pyrophosphate synthetase associated protein 1(PRPSAP1)	GO:0000287~magnesium ion binding,GO:0004749~ribose phosphate diphosphokinase activity,
**DistUt**	**UP**	RARG	retinoic acid receptor gamma(RARG)	GO:0000977~RNA polymerase II regulatory region sequence-specific DNA binding,GO:0003707~steroid hormone receptor activity,GO:0003708~retinoic acid receptor activity,GO:0008270~zinc ion binding,
**DistUt**	**UP**	EHMT2	euchromatic histone lysine methyltransferase 2(EHMT2)	GO:0000977~RNA polymerase II regulatory region sequence-specific DNA binding,GO:0008168~methyltransferase activity,GO:0008270~zinc ion binding,GO:0018024~histone-lysine N-methyltransferase activity,GO:0046974~histone methyltransferase activity (H3-K9 specific),GO:0046976~histone methyltransferase activity (H3-K27 specific),

**DistUt**	**UP**	RREB1	ras responsive element binding protein 1(RREB1)	GO:0000979~RNA polymerase II core promoter sequence-specific DNA binding,GO:0003676~nucleic acid binding,GO:0003700~transcription factor activity, sequence-specific DNA binding,GO:0043565~sequence-specific DNA binding,GO:0044212~transcription regulatory region DNA binding,GO:0046872~metal ion binding,
**DistUt**	**UP**	NFIX	nuclear factor I X(NFIX)	GO:0000981~RNA polymerase II transcription factor activity, sequence-specific DNA binding,GO:0003677~DNA binding,GO:0003700~transcription factor activity, sequence-specific DNA binding,
**DistUt**	**UP**	LOC100153966	activator of 90 kDa heat shock protein ATPase homolog 1(LOC100153966)	GO:0001671~ATPase activator activity,
**DistUt**	**UP**	ANXA9	annexin A9(ANXA9)	GO:0001786~phosphatidylserine binding,GO:0005509~calcium ion binding,GO:0005544~calcium-dependent phospholipid binding,GO:0015464~acetylcholine receptor activity,
**DistUt**	**UP**	ZCCHC14	zinc finger CCHC-type containing 14(ZCCHC14)	GO:0003676~nucleic acid binding,GO:0008270~zinc ion binding,
**DistUt**	**UP**	ZBTB7C	zinc finger and BTB domain containing 7C(ZBTB7C)	GO:0003676~nucleic acid binding,GO:0046872~metal ion binding,
**DistUt**	**UP**	NCOR2	nuclear receptor corepressor 2(NCOR2)	GO:0003677~DNA binding,GO:0003714~transcription corepressor activity,GO:0008134~transcription factor binding,GO:0016922~ligand-dependent nuclear receptor binding,
**DistUt**	**UP**	POLR1D	RNA polymerase I subunit D(POLR1D)	GO:0003677~DNA binding,GO:0003899~DNA-directed RNA polymerase activity,
**DistUt**	**UP**	POLR1A	RNA polymerase I subunit A(POLR1A)	GO:0003677~DNA binding,GO:0003899~DNA-directed RNA polymerase activity,GO:0008270~zinc ion binding,
**DistUt**	**UP**	TET3	tet methylcytosine dioxygenase 3(TET3)	GO:0003677~DNA binding,GO:0005506~iron ion binding,GO:0008270~zinc ion binding,GO:0070579~methylcytosine dioxygenase activity,
**DistUt**	**UP**	SRCAP	Snf2 related CREBBP activator protein(SRCAP)	GO:0003677~DNA binding,GO:0005524~ATP binding,
**DistUt**	**UP**	TBL1X	transducin beta like 1X-linked(TBL1X)	GO:0003682~chromatin binding,GO:0003714~transcription corepressor activity,GO:0044212~transcription regulatory region DNA binding,
**DistUt**	**UP**	UBP1	upstream binding protein 1 (LBP-1a)(UBP1)	GO:0003700~transcription factor activity, sequence-specific DNA binding,GO:0043565~sequence-specific DNA binding,
**DistUt**	**UP**	KCTD1	potassium channel tetramerization domain containing 1(KCTD1)	GO:0003714~transcription corepressor activity,
**DistUt**	**UP**	HSP90AB1	heat shock protein 90 alpha family class B member 1(HSP90AB1)	GO:0003725~double-stranded RNA binding,GO:0005524~ATP binding,GO:0019887~protein kinase regulator activity,GO:0044822~poly(A) RNA binding,
**DistUt**	**UP**	CBX8	chromobox 8(CBX8)	GO:0003727~single-stranded RNA binding,GO:0097027~ubiquitin-protein transferase activator activity,
**DistUt**	**UP**	CAP2	CAP, adenylate cyclase-associated protein, 2 (yeast)(CAP2)	GO:0003779~actin binding,GO:0008179~adenylate cyclase binding,
**DistUt**	**UP**	LOC100624445	gamma-glutamylcyclotransferase-like(LOC100624445)	GO:0003839~gamma-glutamylcyclotransferase activity,
**DistUt**	**UP**	DGAT1	diacylglycerol O-acyltransferase 1(DGAT1)	GO:0003846~2-acylglycerol O-acyltransferase activity,GO:0004144~diacylglycerol O-acyltransferase activity,GO:0008374~O-acyltransferase activity,GO:0016746~transferase activity, transferring acyl groups,
**DistUt**	**UP**	HADH	hydroxyacyl-CoA dehydrogenase(HADH)	GO:0003857~3-hydroxyacyl-CoA dehydrogenase activity,GO:0070403~NAD+ binding,
**DistUt**	**UP**	GMPR	guanosine monophosphate reductase(GMPR)	GO:0003920~GMP reductase activity,GO:0046872~metal ion binding,
**DistUt**	**UP**	DHX32	DEAH-box helicase 32 (putative)(DHX32)	GO:0004004~ATP-dependent RNA helicase activity,GO:0004386~helicase activity,GO:0044822~poly(A) RNA binding,
**DistUt**	**UP**	ALDH6A1	aldehyde dehydrogenase 6 family member A1(ALDH6A1)	GO:0004029~aldehyde dehydrogenase (NAD) activity,GO:0004491~methylmalonate-semialdehyde dehydrogenase (acylating) activity,GO:0018478~malonate-semialdehyde dehydrogenase (acetylating) activity,GO:0044822~poly(A) RNA binding,
**DistUt**	**UP**	ALDH2	aldehyde dehydrogenase 2 family (mitochondrial)(ALDH2)	GO:0004029~aldehyde dehydrogenase (NAD) activity,GO:0016620~oxidoreductase activity, acting on the aldehyde or oxo group of donors, NAD or NADP as acceptor,
**DistUt**	**UP**	CHKA	choline kinase alpha(CHKA)	GO:0004103~choline kinase activity,GO:0004104~cholinesterase activity,GO:0004305~ethanolamine kinase activity,GO:0008144~drug binding,
**DistUt**	**UP**	DGUOK	deoxyguanosine kinase(DGUOK)	GO:0004138~deoxyguanosine kinase activity,GO:0005524~ATP binding,
**DistUt**	**UP**	CAPN5	calpain 5(CAPN5)	GO:0004198~calcium-dependent cysteine-type endopeptidase activity,
**DistUt**	**UP**	CAPN15	calpain 15(CAPN15)	GO:0004198~calcium-dependent cysteine-type endopeptidase activity,GO:0008270~zinc ion binding,
**DistUt**	**UP**	FAH	fumarylacetoacetate hydrolase(FAH)	GO:0004334~fumarylacetoacetase activity,
**DistUt**	**UP**	GSTK1	glutathione S-transferase kappa 1(GSTK1)	GO:0004364~glutathione transferase activity,GO:0004602~glutathione peroxidase activity,GO:0015035~protein disulfide oxidoreductase activity,
**DistUt**	**UP**	FMO2	flavin containing monooxygenase 2(FMO2)	GO:0004497~monooxygenase activity,GO:0004499~N,N-dimethylaniline monooxygenase activity,GO:0050660~flavin adenine dinucleotide binding,GO:0050661~NADP binding,
**DistUt**	**UP**	PCBD1	pterin-4 alpha-carbinolamine dehydratase 1(PCBD1)	GO:0004505~phenylalanine 4-monooxygenase activity,GO:0008124~4-alpha-hydroxytetrahydrobiopterin dehydratase activity,
**DistUt**	**UP**	CNOT2	CCR4-NOT transcription complex subunit 2(CNOT2)	GO:0004535~poly(A)-specific ribonuclease activity,
**DistUt**	**UP**	CNOT1	CCR4-NOT transcription complex subunit 1(CNOT1)	GO:0004535~poly(A)-specific ribonuclease activity,GO:0032947~protein complex scaffold,GO:0044822~poly(A) RNA binding,
**DistUt**	**UP**	PLD2	phospholipase D2(PLD2)	GO:0004630~phospholipase D activity,
**DistUt**	**UP**	MAP4K2	mitogen-activated protein kinase kinase kinase kinase 2(MAP4K2)	GO:0004702~receptor signaling protein serine/threonine kinase activity,GO:0005524~ATP binding,GO:0008349~MAP kinase kinase kinase kinase activity,
**DistUt**	**UP**	CSF1R	colony stimulating factor 1 receptor(CSF1R)	GO:0004714~transmembrane receptor protein tyrosine kinase activity,GO:0005011~macrophage colony-stimulating factor receptor activity,GO:0005524~ATP binding,
**DistUt**	**UP**	NTRK3	neurotrophic receptor tyrosine kinase 3(NTRK3)	GO:0004714~transmembrane receptor protein tyrosine kinase activity,GO:0005030~neurotrophin receptor activity,GO:0005524~ATP binding,
**DistUt**	**UP**	CDC25B	cell division cycle 25B(CDC25B)	GO:0004725~protein tyrosine phosphatase activity,
**DistUt**	**UP**	CDC25C	cell division cycle 25C(CDC25C)	GO:0004725~protein tyrosine phosphatase activity,GO:0019901~protein kinase binding,GO:0050699~WW domain binding,
**DistUt**	**UP**	MTMR7	myotubularin related protein 7(MTMR7)	GO:0004725~protein tyrosine phosphatase activity,GO:0052866~phosphatidylinositol phosphate phosphatase activity,
**DistUt**	**UP**	DHRS3	dehydrogenase/reductase 3(DHRS3)	GO:0004745~retinol dehydrogenase activity,
**DistUt**	**UP**	SPR	sepiapterin reductase (7,8-dihydrobiopterin:NADP+ oxidoreductase)(SPR)	GO:0004757~sepiapterin reductase activity,
**DistUt**	**UP**	RNF167	ring finger protein 167(RNF167)	GO:0004842~ubiquitin-protein transferase activity,GO:0008270~zinc ion binding,
**DistUt**	**UP**	TRIM9	tripartite motif containing 9(TRIM9)	GO:0004842~ubiquitin-protein transferase activity,GO:0008270~zinc ion binding,
**DistUt**	**UP**	USP2	ubiquitin specific peptidase 2(USP2)	GO:0004843~thiol-dependent ubiquitin-specific protease activity,GO:0036459~thiol-dependent ubiquitinyl hydrolase activity,
**DistUt**	**UP**	GABRB2	gamma-aminobutyric acid type A receptor beta2 subunit(GABRB2)	GO:0004890~GABA-A receptor activity,GO:0005230~extracellular ligand-gated ion channel activity,
**DistUt**	**UP**	IL1RAPL2	interleukin 1 receptor accessory protein like 2(IL1RAPL2)	GO:0004910~interleukin-1, Type II, blocking receptor activity,
**DistUt**	**UP**	LOC100152489	olfactory receptor 13C8(LOC100152489)	GO:0004930~G-protein coupled receptor activity,GO:0004984~olfactory receptor activity,

**DistUt**	**UP**	ADRA2A	adrenoceptor alpha 2A(ADRA2A)	GO:0004938~alpha2-adrenergic receptor activity,GO:0019901~protein kinase binding,GO:0031692~alpha-1B adrenergic receptor binding,GO:0031696~alpha-2C adrenergic receptor binding,GO:0031996~thioesterase binding,GO:0032795~heterotrimeric G-protein binding,GO:0042803~protein homodimerization activity,GO:0046982~protein heterodimerization activity,GO:0051379~epinephrine binding,GO:0051380~norepinephrine binding,
**DistUt**	**UP**	GARNL3	GTPase activating Rap/RanGAP domain like 3(GARNL3)	GO:0005096~GTPase activator activity,
**DistUt**	**UP**	WNT7A	Wnt family member 7A(WNT7A)	GO:0005109~frizzled binding,
**DistUt**	**UP**	AQP9	aquaporin 9(AQP9)	GO:0005215~transporter activity,GO:0005345~purine nucleobase transmembrane transporter activity,GO:0005350~pyrimidine nucleobase transmembrane transporter activity,GO:0015204~urea transmembrane transporter activity,GO:0015250~water channel activity,GO:0015254~glycerol channel activity,GO:0015265~urea channel activity,
**DistUt**	**UP**	STARD10	StAR related lipid transfer domain containing 10(STARD10)	GO:0005215~transporter activity,GO:0008289~lipid binding,
**DistUt**	**UP**	ATP2C2	ATPase secretory pathway Ca2+ transporting 2(ATP2C2)	GO:0005388~calcium-transporting ATPase activity,GO:0005524~ATP binding,GO:0015410~manganese-transporting ATPase activity,GO:0046872~metal ion binding,
**DistUt**	**UP**	ATP2A3	ATPase sarcoplasmic/endoplasmic reticulum Ca2+ transporting 3(ATP2A3)	GO:0005388~calcium-transporting ATPase activity,GO:0005524~ATP binding,GO:0046872~metal ion binding,
**DistUt**	**UP**	SLC15A1	solute carrier family 15 member 1(SLC15A1)	GO:0005427~proton-dependent oligopeptide secondary active transmembrane transporter activity,GO:0015198~oligopeptide transporter activity,
**DistUt**	**UP**	ABCA9	ATP binding cassette subfamily A member 9(ABCA9)	GO:0005524~ATP binding,GO:0042626~ATPase activity, coupled to transmembrane movement of substances,
**DistUt**	**UP**	TRAP1	TNF receptor associated protein 1(TRAP1)	GO:0005524~ATP binding,GO:0044822~poly(A) RNA binding,
**DistUt**	**UP**	HSPE1	heat shock protein family E (Hsp10) member 1(HSPE1)	GO:0005524~ATP binding,GO:0046872~metal ion binding,GO:0051082~unfolded protein binding,GO:0051087~chaperone binding,
**DistUt**	**UP**	GNMT	glycine N-methyltransferase(GNMT)	GO:0005542~folic acid binding,GO:0016594~glycine binding,GO:0017174~glycine N-methyltransferase activity,
**DistUt**	**UP**	TDP1	tyrosyl-DNA phosphodiesterase 1(TDP1)	GO:0008081~phosphoric diester hydrolase activity,
**DistUt**	**UP**	VEGFB	vascular endothelial growth factor B(VEGFB)	GO:0008083~growth factor activity,GO:0043183~vascular endothelial growth factor receptor 1 binding,
**DistUt**	**UP**	RNF4	ring finger protein 4(RNF4)	GO:0008134~transcription factor binding,GO:0008270~zinc ion binding,GO:0032184~SUMO polymer binding,GO:0061630~ubiquitin protein ligase activity,
**DistUt**	**UP**	NDUFA10	NADH:ubiquinone oxidoreductase subunit A10(NDUFA10)	GO:0008137~NADH dehydrogenase (ubiquinone) activity,
**DistUt**	**UP**	NDUFV1	NADH:ubiquinone oxidoreductase core subunit V1(NDUFV1)	GO:0008137~NADH dehydrogenase (ubiquinone) activity,GO:0010181~FMN binding,GO:0051287~NAD binding,GO:0051539~4 iron, 4 sulfur cluster binding,
**DistUt**	**UP**	ZSWIM1	zinc finger SWIM-type containing 1(ZSWIM1)	GO:0008270~zinc ion binding,
**DistUt**	**UP**	RUFY1	RUN and FYVE domain containing 1(RUFY1)	GO:0008270~zinc ion binding,GO:0008565~protein transporter activity,
**DistUt**	**UP**	KRT19	keratin 19(KRT19)	GO:0008307~structural constituent of muscle,
**DistUt**	**UP**	CACNB2	calcium voltage-gated channel auxiliary subunit beta 2(CACNB2)	GO:0008331~high voltage-gated calcium channel activity,GO:0086056~voltage-gated calcium channel activity involved in AV node cell action potential,
**DistUt**	**UP**	POFUT1	protein O-fucosyltransferase 1(POFUT1)	GO:0008417~fucosyltransferase activity,GO:0046922~peptide-O-fucosyltransferase activity,
**DistUt**	**UP**	NUDT14	nudix hydrolase 14(NUDT14)	GO:0008768~UDP-sugar diphosphatase activity,GO:0046872~metal ion binding,GO:0047631~ADP-ribose diphosphatase activity,
**DistUt**	**UP**	SLC7A7	solute carrier family 7 member 7(SLC7A7)	GO:0015171~amino acid transmembrane transporter activity,GO:0015174~basic amino acid transmembrane transporter activity,GO:0015179~L-amino acid transmembrane transporter activity,GO:0015297~antiporter activity,
**DistUt**	**UP**	SLC44A1	solute carrier family 44 member 1(SLC44A1)	GO:0015220~choline transmembrane transporter activity,
**DistUt**	**UP**	KCNMB4	potassium large conductance calcium-activated channel, subfamily M, beta member 4(KCNMB4)	GO:0015269~calcium-activated potassium channel activity,
**DistUt**	**UP**	FCGR1A	Fc fragment of IgG, high affinity Ia, receptor (CD64)(FCGR1A)	GO:0019770~IgG receptor activity,
**DistUt**	**UP**	LOC100521617	myelin and lymphocyte protein(LOC100521617)	GO:0019911~structural constituent of myelin sheath,
**DistUt**	**UP**	CUL4A	cullin 4A(CUL4A)	GO:0031625~ubiquitin protein ligase binding,
**DistUt**	**UP**	KBTBD4	kelch repeat and BTB domain containing 4(KBTBD4)	GO:0031625~ubiquitin protein ligase binding,
**DistUt**	**UP**	NIPSNAP1	nipsnap homolog 1 (C. elegans)(NIPSNAP1)	GO:0042165~neurotransmitter binding,
**DistUt**	**UP**	PDF	peptide deformylase (mitochondrial)(PDF)	GO:0042586~peptide deformylase activity,GO:0046872~metal ion binding,
**DistUt**	**UP**	TENM2	teneurin transmembrane protein 2(TENM2)	GO:0042803~protein homodimerization activity,GO:0046982~protein heterodimerization activity,GO:0050839~cell adhesion molecule binding,
**DistUt**	**UP**	OLFML2A	olfactomedin like 2A(OLFML2A)	GO:0042803~protein homodimerization activity,GO:0050840~extracellular matrix binding,
**DistUt**	**UP**	CCDC124	coiled-coil domain containing 124(CCDC124)	GO:0044822~poly(A) RNA binding,
**DistUt**	**UP**	KCTD12	potassium channel tetramerization domain containing 12(KCTD12)	GO:0044822~poly(A) RNA binding,
**DistUt**	**UP**	PRR3	proline rich 3(PRR3)	GO:0046872~metal ion binding,
**DistUt**	**UP**	ATP5E	ATP synthase, H+ transporting, mitochondrial F1 complex, epsilon subunit(ATP5E)	GO:0046933~proton-transporting ATP synthase activity, rotational mechanism,GO:0046961~proton-transporting ATPase activity, rotational mechanism,
**DistUt**	**UP**	LOC100513474	platelet-activating factor acetylhydrolase IB subunit gamma(LOC100513474)	GO:0047179~platelet-activating factor acetyltransferase activity,
**DistUt**	**DOWN**	GUCY1B3	guanylate cyclase 1, soluble, beta 3(GUCY1B3)	GO:0000166~nucleotide binding,GO:0004383~guanylate cyclase activity,GO:0020037~heme binding,
**DistUt**	**DOWN**	RCAN2	regulator of calcineurin 2(RCAN2)	GO:0000166~nucleotide binding,GO:0008597~calcium-dependent protein serine/threonine phosphatase regulator activity,
**DistUt**	**DOWN**	ATP8B2	ATPase phospholipid transporting 8B2(ATP8B2)	GO:0000287~magnesium ion binding,GO:0004012~phospholipid-translocating ATPase activity,GO:0005524~ATP binding,
**DistUt**	**DOWN**	RPS6KA6	ribosomal protein S6 kinase A6(RPS6KA6)	GO:0000287~magnesium ion binding,GO:0004672~protein kinase activity,GO:0004674~protein serine/threonine kinase activity,GO:0005524~ATP binding,
**DistUt**	**DOWN**	FOXP2	forkhead box P2(FOXP2)	GO:0000978~RNA polymerase II core promoter proximal region sequence-specific DNA binding,GO:0000981~RNA polymerase II transcription factor activity, sequence-specific DNA binding,GO:0001078~transcriptional repressor activity, RNA polymerase II core promoter proximal region sequence-specific binding,GO:0003700~transcription factor activity, sequence-specific DNA binding,GO:0043565~sequence-specific DNA binding,
**DistUt**	**DOWN**	LOC100736857	microphthalmia-associated transcription factor-like(LOC100736857)	GO:0000978~RNA polymerase II core promoter proximal region sequence-specific DNA binding,GO:0001077~transcriptional activator activity, RNA polymerase II core promoter proximal region sequence-specific binding,
**DistUt**	**DOWN**	CREB3L2	cAMP responsive element binding protein 3 like 2(CREB3L2)	GO:0000978~RNA polymerase II core promoter proximal region sequence-specific DNA binding,GO:0001077~transcriptional activator activity, RNA polymerase II core promoter proximal region sequence-specific binding,GO:0035497~cAMP response element binding,

**DistUt**	**DOWN**	TCF7L2	transcription factor 7 like 2(TCF7L2)	GO:0000978~RNA polymerase II core promoter proximal region sequence-specific DNA binding,GO:0003700~transcription factor activity, sequence-specific DNA binding,GO:0008013~beta-catenin binding,GO:0043565~sequence-specific DNA binding,GO:0044212~transcription regulatory region DNA binding,
**DistUt**	**DOWN**	HIF1A	hypoxia inducible factor 1, alpha subunit (basic helix-loop-helix transcription factor)(HIF1A)	GO:0000981~RNA polymerase II transcription factor activity, sequence-specific DNA binding,GO:0001076~transcription factor activity, RNA polymerase II transcription factor binding,GO:0001077~transcriptional activator activity, RNA polymerase II core promoter proximal region sequence-specific binding,GO:0003700~transcription factor activity, sequence-specific DNA binding,GO:0003705~transcription factor activity, RNA polymerase II distal enhancer sequence-specific binding,GO:0043565~sequence-specific DNA binding,
**DistUt**	**DOWN**	ZNF516	zinc finger protein 516(ZNF516)	GO:0000987~core promoter proximal region sequence-specific DNA binding,GO:0003700~transcription factor activity, sequence-specific DNA binding,GO:0033613~activating transcription factor binding,GO:0043565~sequence-specific DNA binding,GO:0044212~transcription regulatory region DNA binding,GO:0046872~metal ion binding,
**DistUt**	**DOWN**	TDG	thymine-DNA glycosylase(TDG)	GO:0001104~RNA polymerase II transcription cofactor activity,GO:0004844~uracil DNA N-glycosylase activity,GO:0008263~pyrimidine-specific mismatch base pair DNA N-glycosylase activity,GO:0030983~mismatched DNA binding,GO:0043566~structure-specific DNA binding,
**DistUt**	**DOWN**	AGTR1	angiotensin II receptor type 1(AGTR1)	GO:0001596~angiotensin type I receptor activity,GO:0004945~angiotensin type II receptor activity,GO:0031711~bradykinin receptor binding,GO:0046982~protein heterodimerization activity,
**DistUt**	**DOWN**	ERLEC1	endoplasmic reticulum lectin 1(ERLEC1)	GO:0001948~glycoprotein binding,
**DistUt**	**DOWN**	AFF2	AF4/FMR2 family member 2(AFF2)	GO:0002151~G-quadruplex RNA binding,
**DistUt**	**DOWN**	AGR2	anterior gradient 2, protein disulphide isomerase family member(AGR2)	GO:0002162~dystroglycan binding,
**DistUt**	**DOWN**	PRDM1	PR/SET domain 1(PRDM1)	GO:0003676~nucleic acid binding,GO:0003700~transcription factor activity, sequence-specific DNA binding,GO:0046872~metal ion binding,
**DistUt**	**DOWN**	ZBTB38	zinc finger and BTB domain containing 38(ZBTB38)	GO:0003676~nucleic acid binding,GO:0046872~metal ion binding,
**DistUt**	**DOWN**	ZFAND5	zinc finger AN1-type containing 5(ZFAND5)	GO:0003677~DNA binding,GO:0008270~zinc ion binding,
**DistUt**	**DOWN**	SVEP1	sushi, von Willebrand factor type A, EGF and pentraxin domain containing 1(SVEP1)	GO:0003682~chromatin binding,GO:0005509~calcium ion binding,
**DistUt**	**DOWN**	ABHD2	abhydrolase domain containing 2(ABHD2)	GO:0003707~steroid hormone receptor activity,GO:0042562~hormone binding,GO:0047372~acylglycerol lipase activity,
**DistUt**	**DOWN**	MAGED1	MAGE family member D1(MAGED1)	GO:0003713~transcription coactivator activity,
**DistUt**	**DOWN**	HSBP1L1	heat shock factor binding protein 1 like 1(HSBP1L1)	GO:0003714~transcription corepressor activity,
**DistUt**	**DOWN**	FKBP7	FK506 binding protein 7(FKBP7)	GO:0003755~peptidyl-prolyl cis-trans isomerase activity,GO:0005509~calcium ion binding,GO:0005528~FK506 binding,
**DistUt**	**DOWN**	ERO1A	endoplasmic reticulum oxidoreductase 1 alpha(ERO1A)	GO:0003756~protein disulfide isomerase activity,GO:0015035~protein disulfide oxidoreductase activity,GO:0016671~oxidoreductase activity, acting on a sulfur group of donors, disulfide as acceptor,
**DistUt**	**DOWN**	PDIA3	protein disulfide isomerase family A member 3(PDIA3)	GO:0003756~protein disulfide isomerase activity,GO:0044822~poly(A) RNA binding,
**DistUt**	**DOWN**	TGM2	transglutaminase 2(TGM2)	GO:0003810~protein-glutamine gamma-glutamyltransferase activity,GO:0046872~metal ion binding,
**DistUt**	**DOWN**	B4GALT1	beta-1,4-galactosyltransferase 1(B4GALT1)	GO:0003831~beta-N-acetylglucosaminylglycopeptide beta-1,4-galactosyltransferase activity,GO:0003945~N-acetyllactosamine synthase activity,GO:0004461~lactose synthase activity,GO:0030145~manganese ion binding,
**DistUt**	**DOWN**	HSD11B1	hydroxysteroid 11-beta dehydrogenase 1(HSD11B1)	GO:0003845~11-beta-hydroxysteroid dehydrogenase [NAD(P)] activity,
**DistUt**	**DOWN**	GNAI1	G protein subunit alpha i1(GNAI1)	GO:0003924~GTPase activity,GO:0004871~signal transducer activity,GO:0005525~GTP binding,GO:0031683~G-protein beta/gamma-subunit complex binding,GO:0031821~G-protein coupled serotonin receptor binding,
**DistUt**	**DOWN**	ADAM22	ADAM metallopeptidase domain 22(ADAM22)	GO:0004222~metalloendopeptidase activity,
**DistUt**	**DOWN**	MMP1	matrix metallopeptidase 1(MMP1)	GO:0004222~metalloendopeptidase activity,GO:0005509~calcium ion binding,GO:0008270~zinc ion binding,
**DistUt**	**DOWN**	MMP3	matrix metallopeptidase 3 (stromelysin 1, progelatinase)(MMP3)	GO:0004222~metalloendopeptidase activity,GO:0005509~calcium ion binding,GO:0008270~zinc ion binding,
**DistUt**	**DOWN**	ADAMTS5	ADAM metallopeptidase with thrombospondin type 1 motif 5(ADAMTS5)	GO:0004222~metalloendopeptidase activity,GO:0008201~heparin binding,GO:0008270~zinc ion binding,GO:0050840~extracellular matrix binding,
**DistUt**	**DOWN**	HTRA4	HtrA serine peptidase 4(HTRA4)	GO:0004252~serine-type endopeptidase activity,
**DistUt**	**DOWN**	PLAU	plasminogen activator, urokinase(PLAU)	GO:0004252~serine-type endopeptidase activity,
**DistUt**	**DOWN**	PRSS23	protease, serine 23(PRSS23)	GO:0004252~serine-type endopeptidase activity,
**DistUt**	**DOWN**	FAP	fibroblast activation protein alpha(FAP)	GO:0004252~serine-type endopeptidase activity,GO:0008239~dipeptidyl-peptidase activity,
**DistUt**	**DOWN**	GCLM	glutamate-cysteine ligase modifier subunit(GCLM)	GO:0004357~glutamate-cysteine ligase activity,GO:0030234~enzyme regulator activity,
**DistUt**	**DOWN**	NPR2	natriuretic peptide receptor B/guanylate cyclase B (atrionatriuretic peptide receptor B)(NPR2)	GO:0004383~guanylate cyclase activity,GO:0004672~protein kinase activity,GO:0005524~ATP binding,GO:0016941~natriuretic peptide receptor activity,GO:0042562~hormone binding,
**DistUt**	**DOWN**	ME1	malic enzyme 1(ME1)	GO:0004470~malic enzyme activity,GO:0004471~malate dehydrogenase (decarboxylating) (NAD+) activity,GO:0004473~malate dehydrogenase (decarboxylating) (NADP+) activity,GO:0008948~oxaloacetate decarboxylase activity,GO:0030145~manganese ion binding,GO:0051287~NAD binding,
**DistUt**	**DOWN**	PELO	pelota homolog (Drosophila)(PELO)	GO:0004519~endonuclease activity,GO:0046872~metal ion binding,
**DistUt**	**DOWN**	MAN2A1	mannosidase alpha class 2A member 1(MAN2A1)	GO:0004559~alpha-mannosidase activity,GO:0008270~zinc ion binding,GO:0016799~hydrolase activity, hydrolyzing N-glycosyl compounds,GO:0030246~carbohydrate binding,
**DistUt**	**DOWN**	EDEM3	ER degradation enhancing alpha-mannosidase like protein 3(EDEM3)	GO:0004569~glycoprotein endo-alpha-1,2-mannosidase activity,GO:0004571~mannosyl-oligosaccharide 1,2-alpha-mannosidase activity,GO:0005509~calcium ion binding,
**DistUt**	**DOWN**	GPX8	glutathione peroxidase 8 (putative)(GPX8)	GO:0004602~glutathione peroxidase activity,
**DistUt**	**DOWN**	EIF2AK3	eukaryotic translation initiation factor 2 alpha kinase 3(EIF2AK3)	GO:0004672~protein kinase activity,GO:0004694~eukaryotic translation initiation factor 2alpha kinase activity,GO:0005524~ATP binding,
**DistUt**	**DOWN**	EPHA4	EPH receptor A4(EPHA4)	GO:0004672~protein kinase activity,GO:0005003~ephrin receptor activity,GO:0005004~GPI-linked ephrin receptor activity,GO:0005005~transmembrane-ephrin receptor activity,GO:0005524~ATP binding,GO:0097161~DH domain binding,
**DistUt**	**DOWN**	PDP1	pyruvate dehyrogenase phosphatase catalytic subunit 1(PDP1)	GO:0004722~protein serine/threonine phosphatase activity,GO:0004741~[pyruvate dehydrogenase (lipoamide)] phosphatase activity,GO:0046872~metal ion binding,
**DistUt**	**DOWN**	RNF11	ring finger protein 11(RNF11)	GO:0004842~ubiquitin-protein transferase activity,GO:0008270~zinc ion binding,
**DistUt**	**DOWN**	DCN	decorin(DCN)	GO:0004860~protein kinase inhibitor activity,GO:0005518~collagen binding,GO:0005539~glycosaminoglycan binding,GO:0044822~poly(A) RNA binding,GO:0050840~extracellular matrix binding,
**DistUt**	**DOWN**	LOC100517015	cAMP-dependent protein kinase inhibitor beta-like(LOC100517015)	GO:0004862~cAMP-dependent protein kinase inhibitor activity,
**DistUt**	**DOWN**	COL6A3	collagen type VI alpha 3 chain(COL6A3)	GO:0004867~serine-type endopeptidase inhibitor activity,

**DistUt**	**DOWN**	GJA1	gap junction protein alpha 1(GJA1)	GO:0004871~signal transducer activity,GO:0015075~ion transmembrane transporter activity,GO:0022857~transmembrane transporter activity,GO:1903763~gap junction channel activity involved in cell communication by electrical coupling,
**DistUt**	**DOWN**	MRC2	mannose receptor C type 2(MRC2)	GO:0004888~transmembrane signaling receptor activity,
**DistUt**	**DOWN**	LOC100157704	olfactory receptor 1L8-like(LOC100157704)	GO:0004888~transmembrane signaling receptor activity,GO:0004930~G-protein coupled receptor activity,GO:0004984~olfactory receptor activity,
**DistUt**	**DOWN**	FRZB	frizzled-related protein(FRZB)	GO:0004930~G-protein coupled receptor activity,GO:0017147~Wnt-protein binding,GO:0042813~Wnt-activated receptor activity,
**DistUt**	**DOWN**	PTGFR	prostaglandin F receptor(PTGFR)	GO:0004958~prostaglandin F receptor activity,
**DistUt**	**DOWN**	HRH1	histamine receptor H1(HRH1)	GO:0004969~histamine receptor activity,
**DistUt**	**DOWN**	HTR2A	5-hydroxytryptamine receptor 2A(HTR2A)	GO:0004993~G-protein coupled serotonin receptor activity,GO:0008144~drug binding,GO:0030594~neurotransmitter receptor activity,GO:0051378~serotonin binding,GO:0071886~1-(4-iodo-2,5-dimethoxyphenyl)propan-2-amine binding,
**DistUt**	**DOWN**	FGFR1	fibroblast growth factor receptor 1(FGFR1)	GO:0005007~fibroblast growth factor-activated receptor activity,GO:0005524~ATP binding,GO:0008201~heparin binding,
**DistUt**	**DOWN**	ACKR3	atypical chemokine receptor 3(ACKR3)	GO:0005044~scavenger receptor activity,GO:0015026~coreceptor activity,GO:0016494~C-X-C chemokine receptor activity,
**DistUt**	**DOWN**	WNT2	Wnt family member 2(WNT2)	GO:0005109~frizzled binding,
**DistUt**	**DOWN**	FBN1	fibrillin 1(FBN1)	GO:0005179~hormone activity,GO:0005201~extracellular matrix structural constituent,GO:0005509~calcium ion binding,GO:0008201~heparin binding,
**DistUt**	**DOWN**	CLDN11	claudin 11(CLDN11)	GO:0005198~structural molecule activity,
**DistUt**	**DOWN**	COL1A2	collagen type I alpha 2 chain(COL1A2)	GO:0005201~extracellular matrix structural constituent,
**DistUt**	**DOWN**	COL3A1	collagen type III alpha 1 chain(COL3A1)	GO:0005201~extracellular matrix structural constituent,
**DistUt**	**DOWN**	COL4A1	collagen type IV alpha 1 chain(COL4A1)	GO:0005201~extracellular matrix structural constituent,
**DistUt**	**DOWN**	COL5A2	collagen type V alpha 2 chain(COL5A2)	GO:0005201~extracellular matrix structural constituent,
**DistUt**	**DOWN**	VCAN	versican(VCAN)	GO:0005201~extracellular matrix structural constituent,GO:0005509~calcium ion binding,GO:0005540~hyaluronic acid binding,
**DistUt**	**DOWN**	CRYGS	crystallin gamma S(CRYGS)	GO:0005212~structural constituent of eye lens,
**DistUt**	**DOWN**	SLCO2A1	solute carrier organic anion transporter family, member 2A1(SLCO2A1)	GO:0005215~transporter activity,
**DistUt**	**DOWN**	SLC38A1	solute carrier family 38 member 1(SLC38A1)	GO:0005290~L-histidine transmembrane transporter activity,GO:0015182~L-asparagine transmembrane transporter activity,GO:0015186~L-glutamine transmembrane transporter activity,
**DistUt**	**DOWN**	SLC30A7	solute carrier family 30 member 7(SLC30A7)	GO:0005385~zinc ion transmembrane transporter activity,
**DistUt**	**DOWN**	FBXL5	F-box and leucine rich repeat protein 5(FBXL5)	GO:0005506~iron ion binding,
**DistUt**	**DOWN**	PLOD2	procollagen-lysine,2-oxoglutarate 5-dioxygenase 2(PLOD2)	GO:0005506~iron ion binding,GO:0008475~procollagen-lysine 5-dioxygenase activity,GO:0031418~L-ascorbic acid binding,
**DistUt**	**DOWN**	PRNP	prion protein(PRNP)	GO:0005507~copper ion binding,GO:0008017~microtubule binding,GO:0015631~tubulin binding,GO:0042802~identical protein binding,
**DistUt**	**DOWN**	CDH11	cadherin 11(CDH11)	GO:0005509~calcium ion binding,
**DistUt**	**DOWN**	CALU	calumenin(CALU)	GO:0005509~calcium ion binding,
**DistUt**	**DOWN**	LOC100520265	protocadherin-9(LOC100520265)	GO:0005509~calcium ion binding,
**DistUt**	**DOWN**	SPARC	secreted protein, acidic, cysteine-rich (osteonectin)(SPARC)	GO:0005509~calcium ion binding,GO:0005518~collagen binding,GO:0050840~extracellular matrix binding,
**DistUt**	**DOWN**	ANXA5	annexin A5(ANXA5)	GO:0005509~calcium ion binding,GO:0005544~calcium-dependent phospholipid binding,
**DistUt**	**DOWN**	CD248	CD248 molecule(CD248)	GO:0005509~calcium ion binding,GO:0050840~extracellular matrix binding,
**DistUt**	**DOWN**	HSPA13	heat shock protein family A (Hsp70) member 13(HSPA13)	GO:0005524~ATP binding,
**DistUt**	**DOWN**	ABCB1	ATP-binding cassette, sub-family B (MDR/TAP), member 1(ABCB1)	GO:0005524~ATP binding,GO:0042626~ATPase activity, coupled to transmembrane movement of substances,
**DistUt**	**DOWN**	LOC100152428	multidrug resistance-associated protein 1-like(LOC100152428)	GO:0005524~ATP binding,GO:0042626~ATPase activity, coupled to transmembrane movement of substances,
**DistUt**	**DOWN**	ARF4	ADP ribosylation factor 4(ARF4)	GO:0005525~GTP binding,
**DistUt**	**DOWN**	RASL12	RAS like family 12(RASL12)	GO:0005525~GTP binding,
**DistUt**	**DOWN**	RND3	Rho family GTPase 3(RND3)	GO:0005525~GTP binding,
**DistUt**	**DOWN**	CRISPLD2	cysteine rich secretory protein LCCL domain containing 2(CRISPLD2)	GO:0008201~heparin binding,
**DistUt**	**DOWN**	MORC3	MORC family CW-type zinc finger 3(MORC3)	GO:0008270~zinc ion binding,
**DistUt**	**DOWN**	SEC23A	Sec23 homolog A, coat complex II component(SEC23A)	GO:0008270~zinc ion binding,
**DistUt**	**DOWN**	SEC23B	Sec23 homolog B, coat complex II component(SEC23B)	GO:0008270~zinc ion binding,
**DistUt**	**DOWN**	ENPEP	glutamyl aminopeptidase(ENPEP)	GO:0008270~zinc ion binding,GO:0042277~peptide binding,GO:0070006~metalloaminopeptidase activity,
**DistUt**	**DOWN**	B3GALT2	beta-1,3-galactosyltransferase 2(B3GALT2)	GO:0008499~UDP-galactose:beta-N-acetylglucosamine beta-1,3-galactosyltransferase activity,
**DistUt**	**DOWN**	STEAP2	STEAP2 metalloreductase(STEAP2)	GO:0008823~cupric reductase activity,GO:0052851~ferric-chelate reductase (NADPH) activity,
**DistUt**	**DOWN**	FAM213A	family with sequence similarity 213 member A(FAM213A)	GO:0016209~antioxidant activity,
**DistUt**	**DOWN**	HSD17B11	hydroxysteroid 17-beta dehydrogenase 11(HSD17B11)	GO:0016229~steroid dehydrogenase activity,
**DistUt**	**DOWN**	PLEKHA8	pleckstrin homology domain containing A8(PLEKHA8)	GO:0017089~glycolipid transporter activity,GO:0051861~glycolipid binding,GO:0070273~phosphatidylinositol-4-phosphate binding,GO:0097001~ceramide binding,
**DistUt**	**DOWN**	NCEH1	neutral cholesterol ester hydrolase 1(NCEH1)	GO:0017171~serine hydrolase activity,GO:0042301~phosphate ion binding,GO:0052689~carboxylic ester hydrolase activity,
**DistUt**	**DOWN**	PPP4R4	protein phosphatase 4 regulatory subunit 4(PPP4R4)	GO:0019888~protein phosphatase regulator activity,
**DistUt**	**DOWN**	LOC100739844	semaphorin-3A-like(LOC100739844)	GO:0030215~semaphorin receptor binding,GO:0038191~neuropilin binding,GO:0045499~chemorepellent activity,
**DistUt**	**DOWN**	PLAUR	plasminogen activator, urokinase receptor(PLAUR)	GO:0030377~urokinase plasminogen activator receptor activity,
**DistUt**	**DOWN**	WIPI1	WD repeat domain, phosphoinositide interacting 1(WIPI1)	GO:0032266~phosphatidylinositol-3-phosphate binding,GO:0080025~phosphatidylinositol-3,5-bisphosphate binding,
**DistUt**	**DOWN**	INPP5F	inositol polyphosphate-5-phosphatase F(INPP5F)	GO:0034595~phosphatidylinositol phosphate 5-phosphatase activity,GO:0034596~phosphatidylinositol phosphate 4-phosphatase activity,GO:0052833~inositol monophosphate 4-phosphatase activity,

**DistUt**	**DOWN**	LOC100738373	epidermal growth factor receptor kinase substrate 8-like(LOC100738373)	GO:0035591~signaling adaptor activity,
**DistUt**	**DOWN**	S1PR3	sphingosine-1-phosphate receptor 3(S1PR3)	GO:0038036~sphingosine-1-phosphate receptor activity,
**DistUt**	**DOWN**	HOXA10	homeobox A10(HOXA10)	GO:0043565~sequence-specific DNA binding,
**DistUt**	**DOWN**	HOXA10	homeobox A10(HOXA10)	GO:0043565~sequence-specific DNA binding,
**DistUt**	**DOWN**	BICC1	BicC family RNA binding protein 1(BICC1)	GO:0044822~poly(A) RNA binding,
**DistUt**	**DOWN**	SEC63	SEC63 homolog, protein translocation regulator(SEC63)	GO:0044822~poly(A) RNA binding,
**DistUt**	**DOWN**	HELZ	helicase with zinc finger(HELZ)	GO:0044822~poly(A) RNA binding,GO:0046872~metal ion binding,
**DistUt**	**DOWN**	SLC39A7	solute carrier family 39 member 7(SLC39A7)	GO:0046873~metal ion transmembrane transporter activity,
**DistUt**	**DOWN**	KDELR3	KDEL endoplasmic reticulum protein retention receptor 3(KDELR3)	GO:0046923~ER retention sequence binding,
**DistUt**	**DOWN**	GCNT3	glucosaminyl (N-acetyl) transferase 3, mucin type(GCNT3)	GO:0047225~acetylgalactosaminyl-O-glycosyl-glycoprotein beta-1,6-N-acetylglucosaminyltransferase activity,
**DistUt**	**DOWN**	DMP1	dentin matrix acidic phosphoprotein 1(DMP1)	GO:0050840~extracellular matrix binding,
**DistUt**	**DOWN**	CFL2	cofilin 2(CFL2)	GO:0051015~actin filament binding,
**DistUt**	**DOWN**	SLC41A2	solute carrier family 41 member 2(SLC41A2)	GO:0072509~divalent inorganic cation transmembrane transporter activity,
**ProxUt**	**UP**	CELF4	CUGBP, Elav-like family member 4(CELF4)	GO:0000166~nucleotide binding,GO:0003729~mRNA binding,
**ProxUt**	**UP**	STK11	serine/threonine kinase 11(STK11)	GO:0000287~magnesium ion binding,GO:0004674~protein serine/threonine kinase activity,GO:0005524~ATP binding,GO:0030295~protein kinase activator activity,
**ProxUt**	**UP**	HSF1	heat shock transcription factor 1(HSF1)	GO:0000978~RNA polymerase II core promoter proximal region sequence-specific DNA binding,GO:0000979~RNA polymerase II core promoter sequence-specific DNA binding,GO:0001078~transcriptional repressor activity, RNA polymerase II core promoter proximal region sequence-specific binding,GO:0001162~RNA polymerase II intronic transcription regulatory region sequence-specific DNA binding,GO:0003682~chromatin binding,
**ProxUt**	**UP**	RREB1	ras responsive element binding protein 1(RREB1)	GO:0000979~RNA polymerase II core promoter sequence-specific DNA binding,GO:0003676~nucleic acid binding,GO:0003700~transcription factor activity, sequence-specific DNA binding,GO:0043565~sequence-specific DNA binding,GO:0044212~transcription regulatory region DNA binding,GO:0046872~metal ion binding,
**ProxUt**	**UP**	MLXIP	MLX interacting protein(MLXIP)	GO:0000989~transcription factor activity, transcription factor binding,GO:0003700~transcription factor activity, sequence-specific DNA binding,
**ProxUt**	**UP**	CHST4	carbohydrate sulfotransferase 4(CHST4)	GO:0001517~N-acetylglucosamine 6-O-sulfotransferase activity,
**ProxUt**	**UP**	JAZF1	JAZF zinc finger 1(JAZF1)	GO:0003676~nucleic acid binding,GO:0003714~transcription corepressor activity,GO:0046872~metal ion binding,
**ProxUt**	**UP**	LOC100152736	zinc finger protein 142(LOC100152736)	GO:0003676~nucleic acid binding,GO:0046872~metal ion binding,
**ProxUt**	**UP**	ZNF576	zinc finger protein 576(ZNF576)	GO:0003676~nucleic acid binding,GO:0046872~metal ion binding,
**ProxUt**	**UP**	ZNF697	zinc finger protein 697(ZNF697)	GO:0003676~nucleic acid binding,GO:0046872~metal ion binding,
**ProxUt**	**UP**	DEAF1	DEAF1, transcription factor(DEAF1)	GO:0003677~DNA binding,GO:0003700~transcription factor activity, sequence-specific DNA binding,GO:0046872~metal ion binding,
**ProxUt**	**UP**	THAP11	THAP domain containing 11(THAP11)	GO:0003677~DNA binding,GO:0008270~zinc ion binding,
**ProxUt**	**UP**	SP7	Sp7 transcription factor(SP7)	GO:0003677~DNA binding,GO:0046872~metal ion binding,
**ProxUt**	**UP**	DDB1	damage specific DNA binding protein 1(DDB1)	GO:0003684~damaged DNA binding,
**ProxUt**	**UP**	TMSB4X	thymosin beta 4, X-linked(TMSB4X)	GO:0003785~actin monomer binding,
**ProxUt**	**UP**	DGAT1	diacylglycerol O-acyltransferase 1(DGAT1)	GO:0003846~2-acylglycerol O-acyltransferase activity,GO:0004144~diacylglycerol O-acyltransferase activity,GO:0008374~O-acyltransferase activity,GO:0016746~transferase activity, transferring acyl groups,
**ProxUt**	**UP**	CRYL1	crystallin lambda 1(CRYL1)	GO:0003857~3-hydroxyacyl-CoA dehydrogenase activity,GO:0050104~L-gulonate 3-dehydrogenase activity,GO:0070403~NAD+ binding,
**ProxUt**	**UP**	ALDH2	aldehyde dehydrogenase 2 family (mitochondrial)(ALDH2)	GO:0004029~aldehyde dehydrogenase (NAD) activity,GO:0016620~oxidoreductase activity, acting on the aldehyde or oxo group of donors, NAD or NADP as acceptor,
**ProxUt**	**UP**	RHBDL1	rhomboid like 1(RHBDL1)	GO:0004252~serine-type endopeptidase activity,
**ProxUt**	**UP**	MST1	macrophage stimulating 1(MST1)	GO:0004252~serine-type endopeptidase activity,
**ProxUt**	**UP**	GALK1	galactokinase 1(GALK1)	GO:0004335~galactokinase activity,GO:0005524~ATP binding,GO:0005534~galactose binding,
**ProxUt**	**UP**	KHK	ketohexokinase(KHK)	GO:0004454~ketohexokinase activity,
**ProxUt**	**UP**	PCBD1	pterin-4 alpha-carbinolamine dehydratase 1(PCBD1)	GO:0004505~phenylalanine 4-monooxygenase activity,GO:0008124~4-alpha-hydroxytetrahydrobiopterin dehydratase activity,
**ProxUt**	**UP**	GRK1	G protein-coupled receptor kinase 1(GRK1)	GO:0004703~G-protein coupled receptor kinase activity,GO:0005524~ATP binding,GO:0050254~rhodopsin kinase activity,
**ProxUt**	**UP**	MTMR7	myotubularin related protein 7(MTMR7)	GO:0004725~protein tyrosine phosphatase activity,GO:0052866~phosphatidylinositol phosphate phosphatase activity,
**ProxUt**	**UP**	SDR16C5	short chain dehydrogenase/reductase family 16C, member 5(SDR16C5)	GO:0004745~retinol dehydrogenase activity,
**ProxUt**	**UP**	SPR	sepiapterin reductase (7,8-dihydrobiopterin:NADP+ oxidoreductase)(SPR)	GO:0004757~sepiapterin reductase activity,
**ProxUt**	**UP**	RTN4RL1	reticulon 4 receptor like 1(RTN4RL1)	GO:0004860~protein kinase inhibitor activity,
**ProxUt**	**UP**	GPR26	G protein-coupled receptor 26(GPR26)	GO:0004930~G-protein coupled receptor activity,
**ProxUt**	**UP**	LOC100516511	olfactory receptor 1A1(LOC100516511)	GO:0004930~G-protein coupled receptor activity,GO:0004984~olfactory receptor activity,
**ProxUt**	**UP**	ADRA1A	adrenoceptor alpha 1A(ADRA1A)	GO:0004937~alpha1-adrenergic receptor activity,
**ProxUt**	**UP**	ADRA2A	adrenoceptor alpha 2A(ADRA2A)	GO:0004938~alpha2-adrenergic receptor activity,GO:0019901~protein kinase binding,GO:0031692~alpha-1B adrenergic receptor binding,GO:0031696~alpha-2C adrenergic receptor binding,GO:0031996~thioesterase binding,GO:0032795~heterotrimeric G-protein binding,GO:0042803~protein homodimerization activity,GO:0046982~protein heterodimerization activity,GO:0051379~epinephrine binding,GO:0051380~norepinephrine binding,
**ProxUt**	**UP**	RXFP4	relaxin/insulin like family peptide receptor 4(RXFP4)	GO:0004945~angiotensin type II receptor activity,GO:0008528~G-protein coupled peptide receptor activity,
**ProxUt**	**UP**	LOC100524975	olfactory receptor 10H1(LOC100524975)	GO:0004984~olfactory receptor activity,GO:0004993~G-protein coupled serotonin receptor activity,GO:0030594~neurotransmitter receptor activity,
**ProxUt**	**UP**	VIPR1	vasoactive intestinal peptide receptor 1(VIPR1)	GO:0004999~vasoactive intestinal polypeptide receptor activity,
**ProxUt**	**UP**	IL17D	interleukin 17D(IL17D)	GO:0005125~cytokine activity,GO:0005126~cytokine receptor binding,
**ProxUt**	**UP**	LIM2	lens intrinsic membrane protein 2(LIM2)	GO:0005212~structural constituent of eye lens,

**ProxUt**	**UP**	KCNQ4	potassium voltage-gated channel subfamily Q member 4(KCNQ4)	GO:0005251~delayed rectifier potassium channel activity,
**ProxUt**	**UP**	LOC100515312	C2 calcium-dependent domain-containing protein 4D-like(LOC100515312)	GO:0005509~calcium ion binding,GO:0005544~calcium-dependent phospholipid binding,
**ProxUt**	**UP**	CALM1	calmodulin 1(CALM1)	GO:0005509~calcium ion binding,GO:0072542~protein phosphatase activator activity,
**ProxUt**	**UP**	MAPT	microtubule associated protein tau(MAPT)	GO:0008017~microtubule binding,GO:0071813~lipoprotein particle binding,
**ProxUt**	**UP**	JADE3	jade family PHD finger 3(JADE3)	GO:0008270~zinc ion binding,
**ProxUt**	**UP**	ZSWIM1	zinc finger SWIM-type containing 1(ZSWIM1)	GO:0008270~zinc ion binding,
**ProxUt**	**UP**	PGLYRP1	peptidoglycan recognition protein 1(PGLYRP1)	GO:0008270~zinc ion binding,GO:0008745~N-acetylmuramoyl-L-alanine amidase activity,
**ProxUt**	**UP**	RNF152	ring finger protein 152(RNF152)	GO:0008270~zinc ion binding,GO:0061630~ubiquitin protein ligase activity,
**ProxUt**	**UP**	RHCG	Rh family C glycoprotein(RHCG)	GO:0008519~ammonium transmembrane transporter activity,GO:0030506~ankyrin binding,
**ProxUt**	**UP**	SERINC5	serine incorporator 5(SERINC5)	GO:0015194~L-serine transmembrane transporter activity,
**ProxUt**	**UP**	LOC100739011	putative lipoyltransferase 2, mitochondrial(LOC100739011)	GO:0016415~octanoyltransferase activity,GO:0033819~lipoyl(octanoyl) transferase activity,
**ProxUt**	**UP**	ADHFE1	alcohol dehydrogenase, iron containing 1(ADHFE1)	GO:0016491~oxidoreductase activity,GO:0016616~oxidoreductase activity, acting on the CH-OH group of donors, NAD or NADP as acceptor,GO:0046872~metal ion binding,GO:0047988~hydroxyacid-oxoacid transhydrogenase activity,
**ProxUt**	**UP**	CD207	CD207 molecule, langerin(CD207)	GO:0030246~carbohydrate binding,
**ProxUt**	**UP**	HOXD12	homeobox D12(HOXD12)	GO:0043565~sequence-specific DNA binding,
**ProxUt**	**UP**	OTOGL	otogelin like(OTOGL)	GO:0046556~alpha-L-arabinofuranosidase activity,
**ProxUt**	**DOWN**	IRAK4	interleukin 1 receptor associated kinase 4(IRAK4)	GO:0000287~magnesium ion binding,GO:0004672~protein kinase activity,GO:0004674~protein serine/threonine kinase activity,GO:0005524~ATP binding,
**ProxUt**	**DOWN**	RARA	retinoic acid receptor alpha(RARA)	GO:0000977~RNA polymerase II regulatory region sequence-specific DNA binding,GO:0001972~retinoic acid binding,GO:0003707~steroid hormone receptor activity,GO:0003708~retinoic acid receptor activity,GO:0003713~transcription coactivator activity,GO:0003714~transcription corepressor activity,GO:0008270~zinc ion binding,GO:0031490~chromatin DNA binding,GO:0044323~retinoic acid-responsive element binding,
**ProxUt**	**DOWN**	ELF4	E74 like ETS transcription factor 4(ELF4)	GO:0000978~RNA polymerase II core promoter proximal region sequence-specific DNA binding,GO:0000981~RNA polymerase II transcription factor activity, sequence-specific DNA binding,GO:0001077~transcriptional activator activity, RNA polymerase II core promoter proximal region sequence-specific binding,GO:0003700~transcription factor activity, sequence-specific DNA binding,GO:0043565~sequence-specific DNA binding,
**ProxUt**	**DOWN**	CREB3L2	cAMP responsive element binding protein 3 like 2(CREB3L2)	GO:0000978~RNA polymerase II core promoter proximal region sequence-specific DNA binding,GO:0001077~transcriptional activator activity, RNA polymerase II core promoter proximal region sequence-specific binding,GO:0035497~cAMP response element binding,
**ProxUt**	**DOWN**	TCF7L2	transcription factor 7 like 2(TCF7L2)	GO:0000978~RNA polymerase II core promoter proximal region sequence-specific DNA binding,GO:0003700~transcription factor activity, sequence-specific DNA binding,GO:0008013~beta-catenin binding,GO:0043565~sequence-specific DNA binding,GO:0044212~transcription regulatory region DNA binding,
**ProxUt**	**DOWN**	EPAS1	endothelial PAS domain protein 1(EPAS1)	GO:0000981~RNA polymerase II transcription factor activity, sequence-specific DNA binding,GO:0001077~transcriptional activator activity, RNA polymerase II core promoter proximal region sequence-specific binding,GO:0003700~transcription factor activity, sequence-specific DNA binding,GO:0043565~sequence-specific DNA binding,
**ProxUt**	**DOWN**	ZNF516	zinc finger protein 516(ZNF516)	GO:0000987~core promoter proximal region sequence-specific DNA binding,GO:0003700~transcription factor activity, sequence-specific DNA binding,GO:0033613~activating transcription factor binding,GO:0043565~sequence-specific DNA binding,GO:0044212~transcription regulatory region DNA binding,GO:0046872~metal ion binding,
**ProxUt**	**DOWN**	TDG	thymine-DNA glycosylase(TDG)	GO:0001104~RNA polymerase II transcription cofactor activity,GO:0004844~uracil DNA N-glycosylase activity,GO:0008263~pyrimidine-specific mismatch base pair DNA N-glycosylase activity,GO:0030983~mismatched DNA binding,GO:0043566~structure-specific DNA binding,
**ProxUt**	**DOWN**	P2RX7	purinergic receptor P2X 7(P2RX7)	GO:0001530~lipopolysaccharide binding,GO:0001614~purinergic nucleotide receptor activity,GO:0004931~extracellular ATP-gated cation channel activity,GO:0005524~ATP binding,
**ProxUt**	**DOWN**	AGTR1	angiotensin II receptor type 1(AGTR1)	GO:0001596~angiotensin type I receptor activity,GO:0004945~angiotensin type II receptor activity,GO:0031711~bradykinin receptor binding,GO:0046982~protein heterodimerization activity,
**ProxUt**	**DOWN**	MFGE8	milk fat globule-EGF factor 8 protein(MFGE8)	GO:0001786~phosphatidylserine binding,GO:0008429~phosphatidylethanolamine binding,
**ProxUt**	**DOWN**	ERLEC1	endoplasmic reticulum lectin 1(ERLEC1)	GO:0001948~glycoprotein binding,
**ProxUt**	**DOWN**	LMAN2	lectin, mannose binding 2(LMAN2)	GO:0001948~glycoprotein binding,GO:0005537~mannose binding,
**ProxUt**	**DOWN**	AFF2	AF4/FMR2 family member 2(AFF2)	GO:0002151~G-quadruplex RNA binding,
**ProxUt**	**DOWN**	AGR2	anterior gradient 2, protein disulphide isomerase family member(AGR2)	GO:0002162~dystroglycan binding,
**ProxUt**	**DOWN**	PRDM1	PR/SET domain 1(PRDM1)	GO:0003676~nucleic acid binding,GO:0003700~transcription factor activity, sequence-specific DNA binding,GO:0046872~metal ion binding,
**ProxUt**	**DOWN**	ZBTB1	zinc finger and BTB domain containing 1(ZBTB1)	GO:0003676~nucleic acid binding,GO:0046872~metal ion binding,
**ProxUt**	**DOWN**	SNAI2	snail family transcriptional repressor 2(SNAI2)	GO:0003676~nucleic acid binding,GO:0046872~metal ion binding,
**ProxUt**	**DOWN**	SNAI2	snail homolog 2 (Drosophila)(SNAI2)	GO:0003676~nucleic acid binding,GO:0046872~metal ion binding,
**ProxUt**	**DOWN**	ERCC6	ERCC excision repair 6, chromatin remodeling factor(ERCC6)	GO:0003677~DNA binding,GO:0003682~chromatin binding,GO:0005524~ATP binding,GO:0008094~DNA-dependent ATPase activity,GO:0030296~protein tyrosine kinase activator activity,
**ProxUt**	**DOWN**	STAT2	signal transducer and activator of transcription 2(STAT2)	GO:0003677~DNA binding,GO:0003700~transcription factor activity, sequence-specific DNA binding,GO:0004871~signal transducer activity,
**ProxUt**	**DOWN**	DCLRE1A	DNA cross-link repair 1A(DCLRE1A)	GO:0003684~damaged DNA binding,GO:0035312~5'-3' exodeoxyribonuclease activity,
**ProxUt**	**DOWN**	CREB3L1	cAMP responsive element binding protein 3 like 1(CREB3L1)	GO:0003700~transcription factor activity, sequence-specific DNA binding,GO:0035497~cAMP response element binding,GO:0043565~sequence-specific DNA binding,
**ProxUt**	**DOWN**	XBP1	X-box binding protein 1(XBP1)	GO:0003700~transcription factor activity, sequence-specific DNA binding,GO:0043565~sequence-specific DNA binding,
**ProxUt**	**DOWN**	ABHD2	abhydrolase domain containing 2(ABHD2)	GO:0003707~steroid hormone receptor activity,GO:0042562~hormone binding,GO:0047372~acylglycerol lipase activity,
**ProxUt**	**DOWN**	MAGED1	MAGE family member D1(MAGED1)	GO:0003713~transcription coactivator activity,
**ProxUt**	**DOWN**	RAP2C	RAP2C, member of RAS oncogene family(RAP2C)	GO:0003713~transcription coactivator activity,GO:0005525~GTP binding,GO:0019003~GDP binding,
**ProxUt**	**DOWN**	GMNN	geminin, DNA replication inhibitor(GMNN)	GO:0003714~transcription corepressor activity,
**ProxUt**	**DOWN**	FKBP7	FK506 binding protein 7(FKBP7)	GO:0003755~peptidyl-prolyl cis-trans isomerase activity,GO:0005509~calcium ion binding,GO:0005528~FK506 binding,
**ProxUt**	**DOWN**	ERP44	endoplasmic reticulum protein 44(ERP44)	GO:0003756~protein disulfide isomerase activity,
**ProxUt**	**DOWN**	B4GALT1	beta-1,4-galactosyltransferase 1(B4GALT1)	GO:0003831~beta-N-acetylglucosaminylglycopeptide beta-1,4-galactosyltransferase activity,GO:0003945~N-acetyllactosamine synthase activity,GO:0004461~lactose synthase activity,GO:0030145~manganese ion binding,
**ProxUt**	**DOWN**	HSD11B1	hydroxysteroid 11-beta dehydrogenase 1(HSD11B1)	GO:0003845~11-beta-hydroxysteroid dehydrogenase [NAD(P)] activity,

**ProxUt**	**DOWN**	GNAI1	G protein subunit alpha i1(GNAI1)	GO:0003924~GTPase activity,GO:0004871~signal transducer activity,GO:0005525~GTP binding,GO:0031683~G-protein beta/gamma-subunit complex binding,GO:0031821~G-protein coupled serotonin receptor binding,
**ProxUt**	**DOWN**	RAB32	RAB32, member RAS oncogene family(RAB32)	GO:0003924~GTPase activity,GO:0005525~GTP binding,
**ProxUt**	**DOWN**	SRP54	signal recognition particle 54(SRP54)	GO:0003924~GTPase activity,GO:0005525~GTP binding,GO:0008144~drug binding,GO:0008312~7S RNA binding,GO:0019003~GDP binding,GO:0030942~endoplasmic reticulum signal peptide binding,GO:0043021~ribonucleoprotein complex binding,GO:0044822~poly(A) RNA binding,
**ProxUt**	**DOWN**	LOC100736973	ras-related protein Rap-1b(LOC100736973)	GO:0003924~GTPase activity,GO:0005525~GTP binding,GO:0019003~GDP binding,
**ProxUt**	**DOWN**	PDE7B	phosphodiesterase 7B(PDE7B)	GO:0004115~3',5'-cyclic-AMP phosphodiesterase activity,GO:0046872~metal ion binding,
**ProxUt**	**DOWN**	DCTD	dCMP deaminase(DCTD)	GO:0004132~dCMP deaminase activity,GO:0008270~zinc ion binding,
**ProxUt**	**DOWN**	ECE1	endothelin converting enzyme 1(ECE1)	GO:0004222~metalloendopeptidase activity,
**ProxUt**	**DOWN**	ADAM22	ADAM metallopeptidase domain 22(ADAM22)	GO:0004222~metalloendopeptidase activity,
**ProxUt**	**DOWN**	ADAM12	ADAM metallopeptidase domain 12(ADAM12)	GO:0004222~metalloendopeptidase activity,
**ProxUt**	**DOWN**	BMP1	bone morphogenetic protein 1(BMP1)	GO:0004222~metalloendopeptidase activity,GO:0005125~cytokine activity,GO:0005160~transforming growth factor beta receptor binding,GO:0005509~calcium ion binding,GO:0008270~zinc ion binding,
**ProxUt**	**DOWN**	MMP1	matrix metallopeptidase 1(MMP1)	GO:0004222~metalloendopeptidase activity,GO:0005509~calcium ion binding,GO:0008270~zinc ion binding,
**ProxUt**	**DOWN**	ADAMTS9	ADAM metallopeptidase with thrombospondin type 1 motif 9(ADAMTS9)	GO:0004222~metalloendopeptidase activity,GO:0008270~zinc ion binding,
**ProxUt**	**DOWN**	PLAU	plasminogen activator, urokinase(PLAU)	GO:0004252~serine-type endopeptidase activity,
**ProxUt**	**DOWN**	HTRA4	HtrA serine peptidase 4(HTRA4)	GO:0004252~serine-type endopeptidase activity,
**ProxUt**	**DOWN**	PRSS23	protease, serine 23(PRSS23)	GO:0004252~serine-type endopeptidase activity,
**ProxUt**	**DOWN**	FAP	fibroblast activation protein alpha(FAP)	GO:0004252~serine-type endopeptidase activity,GO:0008239~dipeptidyl-peptidase activity,
**ProxUt**	**DOWN**	MAN2A1	mannosidase alpha class 2A member 1(MAN2A1)	GO:0004559~alpha-mannosidase activity,GO:0008270~zinc ion binding,GO:0016799~hydrolase activity, hydrolyzing N-glycosyl compounds,GO:0030246~carbohydrate binding,
**ProxUt**	**DOWN**	GLB1L	galactosidase beta 1 like(GLB1L)	GO:0004565~beta-galactosidase activity,
**ProxUt**	**DOWN**	MAN1A1	mannosidase alpha class 1A member 1(MAN1A1)	GO:0004571~mannosyl-oligosaccharide 1,2-alpha-mannosidase activity,GO:0005509~calcium ion binding,
**ProxUt**	**DOWN**	GPX8	glutathione peroxidase 8 (putative)(GPX8)	GO:0004602~glutathione peroxidase activity,
**ProxUt**	**DOWN**	CLIC2	chloride intracellular channel 2(CLIC2)	GO:0004602~glutathione peroxidase activity,GO:0005244~voltage-gated ion channel activity,GO:0005254~chloride channel activity,
**ProxUt**	**DOWN**	EPHA4	EPH receptor A4(EPHA4)	GO:0004672~protein kinase activity,GO:0005003~ephrin receptor activity,GO:0005004~GPI-linked ephrin receptor activity,GO:0005005~transmembrane-ephrin receptor activity,GO:0005524~ATP binding,GO:0097161~DH domain binding,
**ProxUt**	**DOWN**	PDP1	pyruvate dehyrogenase phosphatase catalytic subunit 1(PDP1)	GO:0004722~protein serine/threonine phosphatase activity,GO:0004741~[pyruvate dehydrogenase (lipoamide)] phosphatase activity,GO:0046872~metal ion binding,
**ProxUt**	**DOWN**	PTPN1	protein tyrosine phosphatase, non-receptor type 1(PTPN1)	GO:0004725~protein tyrosine phosphatase activity,GO:0008270~zinc ion binding,GO:0044822~poly(A) RNA binding,
**ProxUt**	**DOWN**	USP15	ubiquitin specific peptidase 15(USP15)	GO:0004843~thiol-dependent ubiquitin-specific protease activity,
**ProxUt**	**DOWN**	LOC100517015	cAMP-dependent protein kinase inhibitor beta-like(LOC100517015)	GO:0004862~cAMP-dependent protein kinase inhibitor activity,
**ProxUt**	**DOWN**	COL7A1	collagen type VII alpha 1 chain(COL7A1)	GO:0004867~serine-type endopeptidase inhibitor activity,
**ProxUt**	**DOWN**	LOC100513074	serine protease inhibitor Kazal-type 9(LOC100513074)	GO:0004867~serine-type endopeptidase inhibitor activity,
**ProxUt**	**DOWN**	GJA1	gap junction protein alpha 1(GJA1)	GO:0004871~signal transducer activity,GO:0015075~ion transmembrane transporter activity,GO:0022857~transmembrane transporter activity,GO:1903763~gap junction channel activity involved in cell communication by electrical coupling,
**ProxUt**	**DOWN**	MRC2	mannose receptor C type 2(MRC2)	GO:0004888~transmembrane signaling receptor activity,
**ProxUt**	**DOWN**	FRZB	frizzled-related protein(FRZB)	GO:0004930~G-protein coupled receptor activity,GO:0017147~Wnt-protein binding,GO:0042813~Wnt-activated receptor activity,
**ProxUt**	**DOWN**	KDR	kinase insert domain receptor(KDR)	GO:0005021~vascular endothelial growth factor-activated receptor activity,GO:0005524~ATP binding,
**ProxUt**	**DOWN**	ACKR3	atypical chemokine receptor 3(ACKR3)	GO:0005044~scavenger receptor activity,GO:0015026~coreceptor activity,GO:0016494~C-X-C chemokine receptor activity,
**ProxUt**	**DOWN**	PLEKHG2	pleckstrin homology and RhoGEF domain containing G2(PLEKHG2)	GO:0005089~Rho guanyl-nucleotide exchange factor activity,
**ProxUt**	**DOWN**	SIPA1L1	signal induced proliferation associated 1 like 1(SIPA1L1)	GO:0005096~GTPase activator activity,
**ProxUt**	**DOWN**	TBC1D15	TBC1 domain family member 15(TBC1D15)	GO:0005096~GTPase activator activity,GO:0017137~Rab GTPase binding,
**ProxUt**	**DOWN**	WNT2	Wnt family member 2(WNT2)	GO:0005109~frizzled binding,
**ProxUt**	**DOWN**	FBN1	fibrillin 1(FBN1)	GO:0005179~hormone activity,GO:0005201~extracellular matrix structural constituent,GO:0005509~calcium ion binding,GO:0008201~heparin binding,
**ProxUt**	**DOWN**	CLDN11	claudin 11(CLDN11)	GO:0005198~structural molecule activity,
**ProxUt**	**DOWN**	ADD3	adducin 3(ADD3)	GO:0005198~structural molecule activity,
**ProxUt**	**DOWN**	COL3A1	collagen type III alpha 1 chain(COL3A1)	GO:0005201~extracellular matrix structural constituent,
**ProxUt**	**DOWN**	COL1A2	collagen type I alpha 2 chain(COL1A2)	GO:0005201~extracellular matrix structural constituent,
**ProxUt**	**DOWN**	VCAN	versican(VCAN)	GO:0005201~extracellular matrix structural constituent,GO:0005509~calcium ion binding,GO:0005540~hyaluronic acid binding,
**ProxUt**	**DOWN**	CRYGS	crystallin gamma S(CRYGS)	GO:0005212~structural constituent of eye lens,
**ProxUt**	**DOWN**	FBXL5	F-box and leucine rich repeat protein 5(FBXL5)	GO:0005506~iron ion binding,
**ProxUt**	**DOWN**	PLOD2	procollagen-lysine,2-oxoglutarate 5-dioxygenase 2(PLOD2)	GO:0005506~iron ion binding,GO:0008475~procollagen-lysine 5-dioxygenase activity,GO:0031418~L-ascorbic acid binding,
**ProxUt**	**DOWN**	LOC100520265	protocadherin-9(LOC100520265)	GO:0005509~calcium ion binding,
**ProxUt**	**DOWN**	CDH11	cadherin 11(CDH11)	GO:0005509~calcium ion binding,
**ProxUt**	**DOWN**	SPARC	secreted protein, acidic, cysteine-rich (osteonectin)(SPARC)	GO:0005509~calcium ion binding,GO:0005518~collagen binding,GO:0050840~extracellular matrix binding,
**ProxUt**	**DOWN**	FBLN1	fibulin 1(FBLN1)	GO:0005509~calcium ion binding,GO:0016504~peptidase activator activity,
**ProxUt**	**DOWN**	GPD2	glycerol-3-phosphate dehydrogenase 2(GPD2)	GO:0005509~calcium ion binding,GO:0052591~sn-glycerol-3-phosphate:ubiquinone-8 oxidoreductase activity,
**ProxUt**	**DOWN**	LUM	lumican(LUM)	GO:0005518~collagen binding,

**ProxUt**	**DOWN**	HSPA13	heat shock protein family A (Hsp70) member 13(HSPA13)	GO:0005524~ATP binding,
**ProxUt**	**DOWN**	NSF	N-ethylmaleimide sensitive factor, vesicle fusing ATPase(NSF)	GO:0005524~ATP binding,GO:0016887~ATPase activity,
**ProxUt**	**DOWN**	ABCB1	ATP-binding cassette, sub-family B (MDR/TAP), member 1(ABCB1)	GO:0005524~ATP binding,GO:0042626~ATPase activity, coupled to transmembrane movement of substances,
**ProxUt**	**DOWN**	LOC100152428	multidrug resistance-associated protein 1-like(LOC100152428)	GO:0005524~ATP binding,GO:0042626~ATPase activity, coupled to transmembrane movement of substances,
**ProxUt**	**DOWN**	RAB8B	RAB8B, member RAS oncogene family(RAB8B)	GO:0005525~GTP binding,GO:0019003~GDP binding,
**ProxUt**	**DOWN**	MDFIC	MyoD family inhibitor domain containing(MDFIC)	GO:0008134~transcription factor binding,
**ProxUt**	**DOWN**	CRISPLD2	cysteine rich secretory protein LCCL domain containing 2(CRISPLD2)	GO:0008201~heparin binding,
**ProxUt**	**DOWN**	CPXM1	carboxypeptidase X, M14 family member 1(CPXM1)	GO:0008270~zinc ion binding,
**ProxUt**	**DOWN**	SEC23A	Sec23 homolog A, coat complex II component(SEC23A)	GO:0008270~zinc ion binding,
**ProxUt**	**DOWN**	MORC3	MORC family CW-type zinc finger 3(MORC3)	GO:0008270~zinc ion binding,
**ProxUt**	**DOWN**	ENPEP	glutamyl aminopeptidase(ENPEP)	GO:0008270~zinc ion binding,GO:0042277~peptide binding,GO:0070006~metalloaminopeptidase activity,
**ProxUt**	**DOWN**	B3GNT2	UDP-GlcNAc:betaGal beta-1,3-N-acetylglucosaminyltransferase 2(B3GNT2)	GO:0008378~galactosyltransferase activity,
**ProxUt**	**DOWN**	B3GALT2	beta-1,3-galactosyltransferase 2(B3GALT2)	GO:0008499~UDP-galactose:beta-N-acetylglucosamine beta-1,3-galactosyltransferase activity,
**ProxUt**	**DOWN**	SLC12A2	solute carrier family 12 member 2(SLC12A2)	GO:0008519~ammonium transmembrane transporter activity,GO:0015377~cation:chloride symporter activity,
**ProxUt**	**DOWN**	STEAP2	STEAP2 metalloreductase(STEAP2)	GO:0008823~cupric reductase activity,GO:0052851~ferric-chelate reductase (NADPH) activity,
**ProxUt**	**DOWN**	SLC9B1	solute carrier family 9 member B1(SLC9B1)	GO:0015299~solute:proton antiporter activity,
**ProxUt**	**DOWN**	NOX4	NADPH oxidase 4(NOX4)	GO:0016175~superoxide-generating NADPH oxidase activity,GO:0016491~oxidoreductase activity,GO:0072341~modified amino acid binding,
**ProxUt**	**DOWN**	ENTPD5	ectonucleoside triphosphate diphosphohydrolase 5(ENTPD5)	GO:0016787~hydrolase activity,
**ProxUt**	**DOWN**	VNN1	vanin 1(VNN1)	GO:0017159~pantetheine hydrolase activity,
**ProxUt**	**DOWN**	NCEH1	neutral cholesterol ester hydrolase 1(NCEH1)	GO:0017171~serine hydrolase activity,GO:0042301~phosphate ion binding,GO:0052689~carboxylic ester hydrolase activity,
**ProxUt**	**DOWN**	OLFML3	olfactomedin like 3(OLFML3)	GO:0019164~pyruvate synthase activity,
**ProxUt**	**DOWN**	PPP4R4	protein phosphatase 4 regulatory subunit 4(PPP4R4)	GO:0019888~protein phosphatase regulator activity,
**ProxUt**	**DOWN**	LOC100739844	semaphorin-3A-like(LOC100739844)	GO:0030215~semaphorin receptor binding,GO:0038191~neuropilin binding,GO:0045499~chemorepellent activity,
**ProxUt**	**DOWN**	SELL	selectin L(SELL)	GO:0030246~carbohydrate binding,
**ProxUt**	**DOWN**	PLAUR	plasminogen activator, urokinase receptor(PLAUR)	GO:0030377~urokinase plasminogen activator receptor activity,
**ProxUt**	**DOWN**	LOC100512873	antileukoproteinase(LOC100512873)	GO:0030414~peptidase inhibitor activity,
**ProxUt**	**DOWN**	LOC100623707	phosphatidylinositol 3-kinase regulatory subunit alpha-like(LOC100623707)	GO:0035014~phosphatidylinositol 3-kinase regulator activity,GO:0046935~1-phosphatidylinositol-3-kinase regulator activity,
**ProxUt**	**DOWN**	HOXA10	homeobox A10(HOXA10)	GO:0043565~sequence-specific DNA binding,
**ProxUt**	**DOWN**	HOXA10	homeobox A10(HOXA10)	GO:0043565~sequence-specific DNA binding,
**ProxUt**	**DOWN**	KDELR3	KDEL endoplasmic reticulum protein retention receptor 3(KDELR3)	GO:0046923~ER retention sequence binding,
**ProxUt**	**DOWN**	GCNT3	glucosaminyl (N-acetyl) transferase 3, mucin type(GCNT3)	GO:0047225~acetylgalactosaminyl-O-glycosyl-glycoprotein beta-1,6-N-acetylglucosaminyltransferase activity,
**ProxUt**	**DOWN**	TCEAL8	transcription elongation factor A like 8(TCEAL8)	GO:0050699~WW domain binding,
**ProxUt**	**DOWN**	DMP1	dentin matrix acidic phosphoprotein 1(DMP1)	GO:0050840~extracellular matrix binding,
**ProxUt**	**DOWN**	SDC4	syndecan 4(SDC4)	GO:0070053~thrombospondin receptor activity,
**UTJ**	**UP**	IRF2	interferon regulatory factor 2(IRF2)	GO:0000975~regulatory region DNA binding,GO:0000977~RNA polymerase II regulatory region sequence-specific DNA binding,GO:0001228~transcriptional activator activity, RNA polymerase II transcription regulatory region sequence-specific binding,GO:0003700~transcription factor activity, sequence-specific DNA binding,
**UTJ**	**UP**	MAFB	MAF bZIP transcription factor B(MAFB)	GO:0000978~RNA polymerase II core promoter proximal region sequence-specific DNA binding,GO:0001077~transcriptional activator activity, RNA polymerase II core promoter proximal region sequence-specific binding,GO:0001228~transcriptional activator activity, RNA polymerase II transcription regulatory region sequence-specific binding,
**UTJ**	**UP**	ZNF217	zinc finger protein 217(ZNF217)	GO:0000978~RNA polymerase II core promoter proximal region sequence-specific DNA binding,GO:0001078~transcriptional repressor activity, RNA polymerase II core promoter proximal region sequence-specific binding,GO:0003676~nucleic acid binding,GO:0043565~sequence-specific DNA binding,GO:0044212~transcription regulatory region DNA binding,GO:0046872~metal ion binding,
**UTJ**	**UP**	EP300	E1A binding protein p300(EP300)	GO:0000979~RNA polymerase II core promoter sequence-specific DNA binding,GO:0001228~transcriptional activator activity, RNA polymerase II transcription regulatory region sequence-specific binding,GO:0003684~damaged DNA binding,GO:0003713~transcription coactivator activity,GO:0004402~histone acetyltransferase activity,GO:0004468~lysine N-acetyltransferase activity, acting on acetyl phosphate as donor,GO:0008270~zinc ion binding,GO:0031490~chromatin DNA binding,GO:0097157~pre-mRNA intronic binding,
**UTJ**	**UP**	TSHZ2	teashirt zinc finger homeobox 2(TSHZ2)	GO:0001078~transcriptional repressor activity, RNA polymerase II core promoter proximal region sequence-specific binding,GO:0003677~DNA binding,GO:0003682~chromatin binding,GO:0046872~metal ion binding,
**UTJ**	**UP**	JADE1	jade family PHD finger 1(JADE1)	GO:0001105~RNA polymerase II transcription coactivator activity,GO:0008270~zinc ion binding,
**UTJ**	**UP**	ZXDB	zinc finger, X-linked, duplicated B(ZXDB)	GO:0003676~nucleic acid binding,GO:0046872~metal ion binding,
**UTJ**	**UP**	LOC100627422	ligand-dependent corepressor(LOC100627422)	GO:0003677~DNA binding,GO:0003700~transcription factor activity, sequence-specific DNA binding,
**UTJ**	**UP**	RENBP	renin binding protein(RENBP)	GO:0003824~catalytic activity,GO:0004866~endopeptidase inhibitor activity,GO:0030414~peptidase inhibitor activity,GO:0050121~N-acylglucosamine 2-epimerase activity,
**UTJ**	**UP**	HPGD	hydroxyprostaglandin dehydrogenase 15-(NAD)(HPGD)	GO:0003824~catalytic activity,GO:0004957~prostaglandin E receptor activity,GO:0016404~15-hydroxyprostaglandin dehydrogenase (NAD+) activity,GO:0051287~NAD binding,GO:0070403~NAD+ binding,
**UTJ**	**UP**	UAP1	UDP-N-acetylglucosamine pyrophosphorylase 1(UAP1)	GO:0003977~UDP-N-acetylglucosamine diphosphorylase activity,
**UTJ**	**UP**	ALDH2	aldehyde dehydrogenase 2 family (mitochondrial)(ALDH2)	GO:0004029~aldehyde dehydrogenase (NAD) activity,GO:0016620~oxidoreductase activity, acting on the aldehyde or oxo group of donors, NAD or NADP as acceptor,
**UTJ**	**UP**	CA9	carbonic anhydrase 9(CA9)	GO:0004089~carbonate dehydratase activity,GO:0008270~zinc ion binding,
**UTJ**	**UP**	CHKA	choline kinase alpha(CHKA)	GO:0004103~choline kinase activity,GO:0004104~cholinesterase activity,GO:0004305~ethanolamine kinase activity,GO:0008144~drug binding,

**UTJ**	**UP**	JAK2	Janus kinase 2(JAK2)	GO:0004672~protein kinase activity,GO:0004713~protein tyrosine kinase activity,GO:0004715~non-membrane spanning protein tyrosine kinase activity,GO:0005131~growth hormone receptor binding,GO:0005524~ATP binding,GO:0016301~kinase activity,GO:0020037~heme binding,GO:0035401~histone kinase activity (H3-Y41 specific),GO:0042393~histone binding,GO:0046872~metal ion binding,
**UTJ**	**UP**	HIPK3	homeodomain interacting protein kinase 3(HIPK3)	GO:0004674~protein serine/threonine kinase activity,GO:0005524~ATP binding,
**UTJ**	**UP**	CAMK1D	calcium/calmodulin dependent protein kinase ID(CAMK1D)	GO:0004674~protein serine/threonine kinase activity,GO:0005524~ATP binding,
**UTJ**	**UP**	MAPK10	mitogen-activated protein kinase 10(MAPK10)	GO:0004707~MAP kinase activity,GO:0005524~ATP binding,
**UTJ**	**UP**	DYRK1A	dual specificity tyrosine phosphorylation regulated kinase 1A(DYRK1A)	GO:0004712~protein serine/threonine/tyrosine kinase activity,GO:0005524~ATP binding,
**UTJ**	**UP**	CTDSPL	CTD small phosphatase like(CTDSPL)	GO:0004721~phosphoprotein phosphatase activity,
**UTJ**	**UP**	CDC14A	cell division cycle 14A(CDC14A)	GO:0004722~protein serine/threonine phosphatase activity,GO:0004725~protein tyrosine phosphatase activity,GO:0008138~protein tyrosine/serine/threonine phosphatase activity,
**UTJ**	**UP**	MYLIP	myosin regulatory light chain interacting protein(MYLIP)	GO:0004842~ubiquitin-protein transferase activity,GO:0008270~zinc ion binding,GO:0061630~ubiquitin protein ligase activity,
**UTJ**	**UP**	CD27	CD27 molecule(CD27)	GO:0005031~tumor necrosis factor-activated receptor activity,GO:0043027~cysteine-type endopeptidase inhibitor activity involved in apoptotic process,
**UTJ**	**UP**	ELMO1	engulfment and cell motility 1(ELMO1)	GO:0005085~guanyl-nucleotide exchange factor activity,
**UTJ**	**UP**	ECT2L	epithelial cell transforming 2 like(ECT2L)	GO:0005089~Rho guanyl-nucleotide exchange factor activity,
**UTJ**	**UP**	ARHGAP20	Rho GTPase activating protein 20(ARHGAP20)	GO:0005096~GTPase activator activity,
**UTJ**	**UP**	ADAP2	ArfGAP with dual PH domains 2(ADAP2)	GO:0005096~GTPase activator activity,GO:0005547~phosphatidylinositol-3,4,5-trisphosphate binding,GO:0043533~inositol 1,3,4,5 tetrakisphosphate binding,
**UTJ**	**UP**	ARRB1	arrestin beta 1(ARRB1)	GO:0005096~GTPase activator activity,GO:0043027~cysteine-type endopeptidase inhibitor activity involved in apoptotic process,GO:0044212~transcription regulatory region DNA binding,
**UTJ**	**UP**	CHN2	chimerin 2(CHN2)	GO:0005096~GTPase activator activity,GO:0046872~metal ion binding,
**UTJ**	**UP**	EPB41L1	erythrocyte membrane protein band 4.1 like 1(EPB41L1)	GO:0005200~structural constituent of cytoskeleton,
**UTJ**	**UP**	SLC2A12	solute carrier family 2 member 12(SLC2A12)	GO:0005351~sugar:proton symporter activity,GO:0005355~glucose transmembrane transporter activity,
**UTJ**	**UP**	EPS15	epidermal growth factor receptor pathway substrate 15(EPS15)	GO:0005509~calcium ion binding,
**UTJ**	**UP**	EFCAB6	EF-hand calcium binding domain 6(EFCAB6)	GO:0005509~calcium ion binding,
**UTJ**	**UP**	MTR	5-methyltetrahydrofolate-homocysteine methyltransferase(MTR)	GO:0008270~zinc ion binding,GO:0008705~methionine synthase activity,GO:0008898~S-adenosylmethionine-homocysteine S-methyltransferase activity,GO:0031419~cobalamin binding,
**UTJ**	**UP**	RNF128	ring finger protein 128, E3 ubiquitin protein ligase(RNF128)	GO:0008270~zinc ion binding,GO:0061630~ubiquitin protein ligase activity,
**UTJ**	**UP**	CHDH	choline dehydrogenase(CHDH)	GO:0008812~choline dehydrogenase activity,GO:0050660~flavin adenine dinucleotide binding,
**UTJ**	**UP**	ANKZF1	ankyrin repeat and zinc finger domain containing 1(ANKZF1)	GO:0046872~metal ion binding,
**UTJ**	**UP**	RDH10	retinol dehydrogenase 10 (all-trans)(RDH10)	GO:0052650~NADP-retinol dehydrogenase activity,
**UTJ**	**UP**	TTLL6	tubulin tyrosine ligase like 6(TTLL6)	GO:0070739~protein-glutamic acid ligase activity,
**UTJ**	**DOWN**	ATP8B2	ATPase phospholipid transporting 8B2(ATP8B2)	GO:0000287~magnesium ion binding,GO:0004012~phospholipid-translocating ATPase activity,GO:0005524~ATP binding,
**UTJ**	**DOWN**	CREB3L2	cAMP responsive element binding protein 3 like 2(CREB3L2)	GO:0000978~RNA polymerase II core promoter proximal region sequence-specific DNA binding,GO:0001077~transcriptional activator activity, RNA polymerase II core promoter proximal region sequence-specific binding,GO:0035497~cAMP response element binding,
**UTJ**	**DOWN**	DPF2	double PHD fingers 2(DPF2)	GO:0000978~RNA polymerase II core promoter proximal region sequence-specific DNA binding,GO:0008270~zinc ion binding,
**UTJ**	**DOWN**	ABHD2	abhydrolase domain containing 2(ABHD2)	GO:0003707~steroid hormone receptor activity,GO:0042562~hormone binding,GO:0047372~acylglycerol lipase activity,
**UTJ**	**DOWN**	MAGED1	MAGE family member D1(MAGED1)	GO:0003713~transcription coactivator activity,
**UTJ**	**DOWN**	EEF1G	eukaryotic translation elongation factor 1 gamma(EEF1G)	GO:0003746~translation elongation factor activity,GO:0004364~glutathione transferase activity,
**UTJ**	**DOWN**	B4GALT1	beta-1,4-galactosyltransferase 1(B4GALT1)	GO:0003831~beta-N-acetylglucosaminylglycopeptide beta-1,4-galactosyltransferase activity,GO:0003945~N-acetyllactosamine synthase activity,GO:0004461~lactose synthase activity,GO:0030145~manganese ion binding,
**UTJ**	**DOWN**	MMP19	matrix metallopeptidase 19(MMP19)	GO:0004222~metalloendopeptidase activity,GO:0004252~serine-type endopeptidase activity,GO:0005509~calcium ion binding,GO:0008270~zinc ion binding,
**UTJ**	**DOWN**	HTRA4	HtrA serine peptidase 4(HTRA4)	GO:0004252~serine-type endopeptidase activity,
**UTJ**	**DOWN**	PRSS23	protease, serine 23(PRSS23)	GO:0004252~serine-type endopeptidase activity,
**UTJ**	**DOWN**	ALDH18A1	aldehyde dehydrogenase 18 family member A1(ALDH18A1)	GO:0004349~glutamate 5-kinase activity,GO:0004350~glutamate-5-semialdehyde dehydrogenase activity,GO:0017084~delta1-pyrroline-5-carboxylate synthetase activity,GO:0044822~poly(A) RNA binding,
**UTJ**	**DOWN**	GAPDH	glyceraldehyde-3-phosphate dehydrogenase(GAPDH)	GO:0004365~glyceraldehyde-3-phosphate dehydrogenase (NAD+) (phosphorylating) activity,GO:0008017~microtubule binding,GO:0016620~oxidoreductase activity, acting on the aldehyde or oxo group of donors, NAD or NADP as acceptor,GO:0035605~peptidyl-cysteine S-nitrosylase activity,GO:0050661~NADP binding,GO:0051287~NAD binding,
**UTJ**	**DOWN**	NPR2	natriuretic peptide receptor B/guanylate cyclase B (atrionatriuretic peptide receptor B)(NPR2)	GO:0004383~guanylate cyclase activity,GO:0004672~protein kinase activity,GO:0005524~ATP binding,GO:0016941~natriuretic peptide receptor activity,GO:0042562~hormone binding,
**UTJ**	**DOWN**	HMGCS1	3-hydroxy-3-methylglutaryl-CoA synthase 1(HMGCS1)	GO:0004421~hydroxymethylglutaryl-CoA synthase activity,
**UTJ**	**DOWN**	CYP51	cytochrome P450, family 51, subfamily A, polypeptide 1(CYP51)	GO:0004497~monooxygenase activity,GO:0005506~iron ion binding,GO:0008168~methyltransferase activity,GO:0008398~sterol 14-demethylase activity,GO:0016705~oxidoreductase activity, acting on paired donors, with incorporation or reduction of molecular oxygen,GO:0020037~heme binding,
**UTJ**	**DOWN**	SQLE	squalene epoxidase(SQLE)	GO:0004506~squalene monooxygenase activity,GO:0050660~flavin adenine dinucleotide binding,
**UTJ**	**DOWN**	PGK1	phosphoglycerate kinase 1(PGK1)	GO:0004618~phosphoglycerate kinase activity,GO:0005524~ATP binding,
**UTJ**	**DOWN**	CCNO	cyclin O(CCNO)	GO:0004844~uracil DNA N-glycosylase activity,
**UTJ**	**DOWN**	GNG2	G protein subunit gamma 2(GNG2)	GO:0004871~signal transducer activity,
**UTJ**	**DOWN**	ACKR3	atypical chemokine receptor 3(ACKR3)	GO:0005044~scavenger receptor activity,GO:0015026~coreceptor activity,GO:0016494~C-X-C chemokine receptor activity,
**UTJ**	**DOWN**	GDI2	GDP dissociation inhibitor 2(GDI2)	GO:0005093~Rab GDP-dissociation inhibitor activity,GO:0005096~GTPase activator activity,GO:0016491~oxidoreductase activity,GO:0044822~poly(A) RNA binding,
**UTJ**	**DOWN**	TP23	basic proline-rich protein(TP23)	GO:0005179~hormone activity,
**UTJ**	**DOWN**	FBN1	fibrillin 1(FBN1)	GO:0005179~hormone activity,GO:0005201~extracellular matrix structural constituent,GO:0005509~calcium ion binding,GO:0008201~heparin binding,
**UTJ**	**DOWN**	COL1A2	collagen type I alpha 2 chain(COL1A2)	GO:0005201~extracellular matrix structural constituent,
**UTJ**	**DOWN**	VCAN	versican(VCAN)	GO:0005201~extracellular matrix structural constituent,GO:0005509~calcium ion binding,GO:0005540~hyaluronic acid binding,
**UTJ**	**DOWN**	FBXL5	F-box and leucine rich repeat protein 5(FBXL5)	GO:0005506~iron ion binding,
**UTJ**	**DOWN**	CD248	CD248 molecule(CD248)	GO:0005509~calcium ion binding,GO:0050840~extracellular matrix binding,

**UTJ**	**DOWN**	CALM2	calmodulin 2(CALM2)	GO:0005509~calcium ion binding,GO:0072542~protein phosphatase activator activity,
**UTJ**	**DOWN**	HSPA13	heat shock protein family A (Hsp70) member 13(HSPA13)	GO:0005524~ATP binding,
**UTJ**	**DOWN**	RHOC	ras homolog family member C(RHOC)	GO:0005525~GTP binding,
**UTJ**	**DOWN**	CPXM1	carboxypeptidase X, M14 family member 1(CPXM1)	GO:0008270~zinc ion binding,
**UTJ**	**DOWN**	SEC23A	Sec23 homolog A, coat complex II component(SEC23A)	GO:0008270~zinc ion binding,
**UTJ**	**DOWN**	SLC9B1	solute carrier family 9 member B1(SLC9B1)	GO:0015299~solute:proton antiporter activity,
**UTJ**	**DOWN**	SEC61G	Sec61 translocon gamma subunit(SEC61G)	GO:0015450~P-P-bond-hydrolysis-driven protein transmembrane transporter activity,
**UTJ**	**DOWN**	PLAUR	plasminogen activator, urokinase receptor(PLAUR)	GO:0030377~urokinase plasminogen activator receptor activity,
**UTJ**	**DOWN**	FAM98A	family with sequence similarity 98 member A(FAM98A)	GO:0044822~poly(A) RNA binding,
**UTJ**	**DOWN**	KDELR3	KDEL endoplasmic reticulum protein retention receptor 3(KDELR3)	GO:0046923~ER retention sequence binding,
**UTJ**	**DOWN**	SHAS2	hyaluronan synthase 2(SHAS2)	GO:0050501~hyaluronan synthase activity,
